# Academic Outcomes in Primary and Secondary School Students Prescribed Long-Acting Stimulants for ADHD Management

**DOI:** 10.1177/10870547251378169

**Published:** 2025-10-07

**Authors:** Chris Folkins, Chandy Somayaji, Simerpal K. Gill, James Ted McDonald

**Affiliations:** 1 University of New Brunswick, Fredericton, NB, Canada; 2 Takeda Pharmaceutical Company Limited, Toronto, ON, Canada

**Keywords:** academic performance, ADHD, population-based study

## Abstract

**Objective:**

This study examines the impact of long-acting stimulant (LAS) pharmacotherapy for ADHD on academic outcomes among students in grades K-12 using retrospective analysis of administrative data.

**Methods:**

ADHD diagnosis was identified based on ADHD management plans in school records, physician notes in billing records, and/or prescription records. Prescription records identified LAS-treated students (*n* = 15,544), excluding those treated with immediate/intermediate-acting stimulants or atomoxetine. A control group without ADHD (*n* = 204,681), and another with untreated ADHD (*n* = 27,880) were also identified. The following outcomes were examined using multivariate regression: report card scores, standardized assessment exam performance, graduation from high school, school attendance, and transition to post-secondary education.

**Results:**

ADHD was associated with lower average report card scores and provincial assessment exam scores and increased frequency of school absence among grades K-12, and decreased likelihood of high school graduation and transition to post-secondary education. LAS treatment was associated with improved report card (score estimate −4.93 Treated, −6.19 Untreated) and provincial assessment exam scores (percentile rank estimate −9.20 Treated, −11.50 Untreated) among grades 9 to 12, reduced absences among grades K-12 (absence rate estimate −3.33 Treated, 7.96 Untreated), and increased likelihood of graduation (OR of failure to graduate 1.39 Treated, 2.22 Untreated) and transition to post-secondary education (OR of no transition 0.77 Treated, 1.42 Untreated; reference = No ADHD group).

**Conclusion:**

LAS pharmacotherapy is associated with improved academic performance, attendance, and likelihood of graduation and transition to post-secondary education.

## Introduction

ADHD is a common neurodevelopmental disorder in school-aged individuals, with an estimated worldwide prevalence of 3.4% among children and adolescents (Polanczyk et al., 2015). Quality of life is significantly impaired among those with ADHD as the impacts on self-esteem and functionality span across multiple domains of life (Faraone et al., 2015). Data consistently demonstrate the negative consequences of ADHD on individuals, particularly during the formative years, including increased risks for mental health disorders, motor vehicle accidents, injuries, suicidality, criminality, and adverse educational outcomes (Chang et al., 2019; Faraone et al., 2015). ADHD negatively impacts academic achievement as measured by a variety of metrics including teacher/parent rating, grade point averages, grade retention, high school graduation, and transition to post-secondary education (Frazier et al., 2007; Polderman et al., 2010). Stimulant medications, particularly long-acting stimulants (LAS), are considered the gold standard for medical treatment of ADHD (Almagor et al., 2020). They have been shown to improve the core symptoms of ADHD (Van der Oord et al., 2008) such as inattention and hyperactivity, however their effects on functional academic outcomes are less well-defined.

A 2013 review and meta-analysis of 43 randomized controlled trials of stimulant medications (Prasad et al., 2013) reported that most of the reviewed studies found that stimulants were associated with significant improvements in academic productivity and on-task behavior in students with ADHD, although some studies reported no such benefit. Substantially fewer reviewed studies showed improvement in accuracy of academic work associated with stimulant medications. A more recent 2019 review and meta-analysis of 34 placebo-controlled studies (Kortekaas-Rijlaarsdam et al., 2019) found that methylphenidate treatment of students with ADHD significantly improved math productivity and to a lesser extent accuracy, significantly improved reading productivity but not accuracy, and did not conclusively improve outcomes related to spelling.

Most studies on academic outcomes associated with stimulant drug therapy have examined relatively small sample sizes in controlled experiments, however a smaller number of studies have used administrative data to examine drug effects on a much larger scale in a real-world setting. A review and meta-analysis conducted in 2020 (Boland et al., 2020) examined eight studies that used large databases to investigate the impact of stimulant medications on academic outcomes. Five of these eight studies reported that stimulant medications were associated with significantly higher test scores and grade point averages and reduced school absence; however, one study (Currie et al., 2014) showed no relationship between stimulants and academic outcomes, and two studies (van der Schans et al., 2017; Zoëga et al., 2012) showed that stimulants were associated with a decline in academic performance.

Administrative databases are a valuable tool for studying functional outcomes associated with ADHD drug therapy. These databases generally include a broad sample universe and are linkable at the individual level across a variety of administrative data resources, permitting longitudinal, often population-level studies that are capable of assessing relationships with, and adjusting for, a broad spectrum of covariates that include health, demographic, and socioeconomic data. A key advantage of this approach is its potential for efficient generation of real-world evidence, which can provide a novel perspective that may help to improve our understanding of the impact of stimulant medications on academic outcomes. Since relatively few large-scale administrative data studies have addressed this topic, and those that have offer seemingly conflicting evidence (Boland et al., 2020), further studies using this approach are warranted.

This study uses administrative data to examine the relationship between LAS pharmacotherapy and academic outcomes in New Brunswick grade school students with ADHD. By examining relevant measures of academic achievement including report card and standardized test scores, high school graduation and transition to post-secondary education, as well as school attendance, in a natural setting at the population level, this study aims to provide novel insights into the real-world effects of LAS pharmacotherapy for ADHD.

## Methods

### General Methods

This study used linkable, individual-level administrative health, education, and demographic data accessed via DataNB. DataNB data are pseudonymized and accessed according to established protocols to protect individual privacy. Study protocol was approved by the University of New Brunswick’s Research Ethics Board.

#### Definition of ADHD Diagnosis

Given the absence of a validated algorithm for ADHD diagnosis using NB administrative data, our method integrated multiple data sources to ensure robust case identification, drawing from physician billing records, prescription drug data, and academic records. A diagnosis of ADHD was assumed if one or more of the following indicators were present in administrative data records: (i) free-text notes in physician service billing records referencing ADHD (between Apr 1, 2008 and Nov 17, 2021). This approach was used as an alternative to the more commonly used International Classification of Disease (ICD) diagnostic codes, as diagnoses were not reported in NB physician claims data using ICD codes during the years examined in this study; (ii) history of dispensed LAS prescriptions (Jan 1, 2015–Dec 31, 2021), based on the rationale that LAS are predominantly used for the treatment of ADHD. Previous studies using administrative data have also inferred ADHD diagnosis based on prescribed ADHD medications (Jangmo et al., 2019; Larsson et al., 2013); (iii) academic records indicating a medical plan for ADHD management has been implemented (July 1, 2019–June 30, 2021). Such management plans are only implemented for students with ADHD, and their presence in academic records is therefore indicative of an ADHD diagnosis. Individuals were considered to have ADHD as of 180 days before their earliest diagnostic indicator, or as of their 12th birthday if their earliest indicator occurred after their 12th birthday (because diagnostic criteria require that symptoms are present for at least 6 months before a diagnosis is rendered, and are present since before age 12 years for diagnosis in older individuals; American Psychiatric Association, 2022).

#### Definition of Diagnosis/Treatment Groups

In each academic year (AY; Sept 1–June 30), study subjects were allocated to one of three diagnosis/treatment groups: Treated, Untreated, or No ADHD.

Individuals were included in the Treated group during a given AY if they had at least 150 days of continuous LAS treatment within that AY. The rationale for this definition was that treatment for at least half of an AY (which has a duration of approximately 300 days) was assumed to be sufficient to have the potential to influence academic outcomes during that AY. Continuous treatment was defined as beginning with the fourth consecutive lifetime LAS prescription (considering all available prescription records) and continuing for as long as prescriptions were dispensed without gaps exceeding 180 days. The definition of continuous treatment as consecutive prescriptions without gaps greater than 180 days has been used in previous ADHD studies (Chen et al., 2014; Lichtenstein et al., 2012; Lu et al., 2017; Zetterqvist et al., 2013), and is meant to capture individuals receiving ongoing LAS drug therapy while making reasonable allowances for delays in prescription refills due to logistical or financial reasons, or imperfect adherence. The initial washout period (i.e., beginning the definition of continuous treatment with the fourth LAS prescription) was added to exclude individuals during the initial drug trials and dose titration that is common when initiating LAS therapy, with the intention of limiting inclusion in the Treated group to individuals on a stable, consistent LAS treatment regimen.

Individuals were included in the Untreated group during a given AY if they met the ADHD diagnosis criteria but had no dispensed LAS prescriptions during the AY or in the 180 days preceding it. This definition was intended to capture individuals with ADHD who were not receiving LAS treatment during the AY of interest. The rationale for the definition was that if an individual was not treated with LAS during or in the 180 days preceding the AY (a threshold reflecting the definition of continuous treatment, as described above), it would be unlikely that LAS would influence their academic outcomes during that AY.

Individuals were included in the No ADHD group during a given AY if they had no administrative data records (across physician billing, prescription, or academic records) at any time (before or after that AY) indicating a diagnosis of ADHD. The rationale for this definition was that the precise timing that ADHD symptoms began for an individual cannot be determined using administrative data, so limiting inclusion in the No ADHD group to individuals with no indicators of ADHD diagnosis at any time minimizes the risk of misclassification.

For all outcomes besides attendance, individuals with ADHD were classified as one of either Treated or Untreated for the entirety of each AY. The rationale for this was that these outcomes (e.g., report card scores) reflect academic performance over an entire AY, so performance was attributed to whichever condition (Treated or Untreated) was predominant during that AY. For the attendance outcome, individuals with ADHD were categorized as Treated during periods of continuous treatment, and as Untreated outside of periods of continuous treatment, irrespective of AY. The rationale for this distinction was that, unlike the other outcomes, attendance is not a reflection of academic performance over the entirety of an AY and can therefore be assessed during varying observation windows without regard to the bounds of an AY.

#### Exclusions

To ensure that any observed impacts on outcomes were attributable to LAS therapy and not other common ADHD medications, individuals being treated with short- or intermediate-acting stimulants or atomoxetine were excluded. This approach was intended to minimize potential confounding due to non-LAS medications and allow for a focused evaluation of LAS treatment effects.

For outcomes other than attendance, individuals with prescriptions for short- or intermediate-acting stimulants or atomoxetine during or within 180 days before a given AY were excluded from that AY. For the attendance outcome, individuals with prescriptions for these medications during a given observation window were excluded from that observation window.

Additionally, for outcomes other than attendance, individuals who died or left NB during an AY were excluded from that AY (to ensure that assessments of academic outcomes associated with a given AY would include only those individuals who completed that AY). For the attendance outcome, if an individual died or left NB during an observation window, they were included for the portion of the observation window during which they could be observed and excluded following their death or departure (irrespective of AY).

#### Independent Variables in Regression Models

Using linked administrative data, a range of independent variables were included in the regression models to account for potential confounders and sources of variation in educational outcomes among children and adolescents. The variables (listed below) were selected based on their theoretical and empirical relevance to the outcomes under investigation and to ensure comparability with previous studies in ADHD research. The rationale for each variable is outlined below:

- Age and sex: Included to adjust for variations in age and sex distribution between diagnosis/treatment groups, as age and sex may have impacted the academic outcomes observed.
- NB health zone of residence: Included to adjust for regional differences in healthcare access and service delivery between New Brunswick’s seven health zones, which may impact ADHD management and therefore may have influenced academic outcomes according to zone of residence.
- School district; program of study: As a bilingual province, NB includes an anglophone and francophone school district and offers English, French, and French Immersion-based academic programs. These variables were included to adjust for potential district and program-associated differences in curricula, educational experiences, academic expectations, evaluation criteria and availability of support services, and accommodations for ADHD that may have influenced the observed academic outcomes.
- Comorbid conditions: Conditions that are commonly comorbid with ADHD, including asthma, diabetes, epilepsy, mood and anxiety disorders, and schizophrenia, may independently impact school attendance and academic performance. These comorbidities were included in regression models to adjust for their influence on the observed outcomes.
- Select medications: Several non-LAS medications commonly used in the treatment of ADHD, including second generation antipsychotics, clonidine, modafinil, and guanfacine, may independently influence academic outcomes among students with ADHD. These medications were therefore included collectively as a variable in the regression models.
- Household composition: This dimension reflects the number of adults (0, 1, or 2+ individuals age 22+ years) and other children (0 or 1+ individuals age 21 years or under) residing with the student and is meant to account for differences in family dynamics and availability of parental/adult support which may influence academic outcomes.
- Recent immigration: Recent immigrant status (immigrated to Canada within past 5 years) was included to account for challenges faced by families new to the country, such as language barriers, cultural differences, and systemic challenges in accessing educational resources, which may independently influence academic outcomes.
- Household income quintile, Canadian Index of Multiple Deprivation (CIMD), and history of social assistance use: Included to account for socioeconomic disparities which may affect access to resources, stability of home life, and other factors which may influence academic outcomes. CIMD is a composite index that includes several indicators of material deprivation across the following dimensions (each presented as quintiles): residential instability, economic dependency, ethnocultural composition, and situational vulnerability. The social assistance variable classifies individuals based on whether they did or did not receive social assistance payments within the past 5 years.

Models for attendance additionally included duration of each observation window as an independent variable to adjust for differences in observation time. In all models, values of time-varying covariates (e.g., age and area of residence) were taken at the midpoint of the observation window. For income and CIMD, individual-level data were not available, so Statistics Canada data were used to estimate each variable’s average value for each census dissemination area (DA). Each DA’s average value was then assigned to each resident of that DA.

#### Statistical Analysis

Analysis was conducted using SAS 9.4. Generalized linear models (GLM) and logistic regression models were employed to evaluate continuous and binary outcomes, respectively. The proc_glm function was used for continuous outcomes (report card scores, absences per person per observation window, and standardized exam percentiles), while the proc_logistic function was applied to binary outcomes (high school graduation and transition to post-secondary education). Regression estimates were deemed statistically significant if *p* ≤ .05.

Regression analysis was selected as the primary method because it offers a robust and widely used approach for examining relationships between multiple predictors and outcomes in observational datasets. This approach allows for the simultaneous adjustment for a broad range of covariates including demographic and socioeconomic factors (e.g., age, sex, and household income), household composition, comorbid conditions, and program of study, which provides a useful means of controlling for potential confounding and heterogeneity in the data. The complexity of the dataset and the need to account for varying individual, household, and contextual factors made regression analysis the most suitable choice for isolating the effects of LAS on educational outcomes.

The use of regression analysis is consistent with standard practices in the literature examining the impact of ADHD treatments on academic outcomes, as seen in previous studies (Barbaresi et al., 2007; Baweja et al., 2015; Fleming et al., 2017; Jangmo et al., 2019; Keilow et al., 2018; Marcus & Durkin, 2011) These studies similarly leveraged regression models to account for confounders and heterogeneity inherent in large observational datasets.

To address the specific objectives of this study, regression models were designed to compare outcomes across diagnosis/treatment groups. Estimates for the Treated and Untreated groups used the No ADHD group as the reference category, and additional models directly compared Treated and Untreated groups to assess the differential impact of LAS. Reported n values in the results represent the total number of observations included in each model. For most outcomes, one observation was counted per student per AY. For the attendance outcome, each Treated/Untreated period for a student within an AY was counted as a separate observation to reflect changes in treatment status during the year.

### Outcome-Specific Methods

The outcomes examined and associated cohort definitions and statistical models used for each are summarized in Table 1 below. Data for all analyses were sourced from linked administrative databases. The inclusion and exclusion criteria described under General Methods above were applied to each cohort.

**Table 1.** Outcome-specific Methods.

Outcome	Cohort description	Outcome description	Statistical model
Report card scores	Students enrolled in grades K-12 for at least one full AY from AY2017 to 2020	Overall and subject-specific (math, STEM, and language) mean of all reported competency/course scores for each AY. Grades K-8 scored using 1–4 scale; grades 9–12 using percentage (scores of 0 excluded).	Multivariate GLM regression
Performance on standardized provincial assessment exams	Students who completed percentage-based standardized exams for grades 2, 3, 4, 6, 8, 9, 10, and 11 from AY2017 to 2019.	Overall and subject-specific (math, STEM, and language) scores were converted to percentile ranks among students who wrote the same exam in the same year. Individual ranks were assigned to diagnosis/treatment groups based on the AY in which the exam was written, except for exams written in September, which were assigned based on the AY prior. Mean percentile ranks for each diagnosis/treatment group were computed.	Multivariate GLM regression
Graduation from high school	Students enrolled in grade 12 for the first time from AY2017 to 2019.	Likelihood of failing grade 12 on first attempt. Students were assigned to diagnosis/treatment groups based on their grade 12 year.	Multivariate logistic regression
School attendance	Students enrolled in grades K-12 for any portion of AY2018–2020 (excluding COVID-19 closure periods, Mar–June 2020)	Absences per student per observation window, overall, and by reason for absence (illness, medical appointment, suspension, and unknown; absences for sports or bereavement were excluded)	Multivariate GLM regression
Transition to post-secondary education	Students graduating from grade 12 in AY2018 who maintained active NB Medicare status through first 6 months of AY2019	Likelihood of not enrolling in NB post-secondary institutions within first 6 months of AY2019. Students were assigned to diagnosis/treatment groups based on their grade 12 year. Enrollment in NB’s four public universities and three of its four public colleges was examined.	Multivariate logistic regression

## Results

### Report Card Scores

Table 2 shows background characteristics of the report card score cohorts (records with scores of 0 were excluded from regression analyses). Among grade K-8 students, regression estimates for mean overall report card score were significantly lower among Treated and Untreated students with ADHD compared to those without ADHD (Table 3A). Estimates for the Treated and Untreated groups were not significantly different from one another (Supplemental Table S3a). The same pattern was evident across all subject-specific score estimates (Supplemental Tables S3b–S3d). Similar results were noted (Supplemental Tables S4a–S4h) when academic performance was modeled as the proportion of report card scores meeting minimum acceptable standards (3 or higher on 4-point scale).

**Table 2.** Background Characteristics for Grades K-8 and 9 to 12 Report Card Score Cohorts (AY 2017–2020).

Variable	Diagnosis/treatment group	Grades K-8 Cohort (*n* = ,778)	Grades 9–12 Cohort (*n* = 88,327)
N in each ADHD Treatment Group (% of total cohort)	Treated ADHD	9,800 (6.1%)	5,744 (6.5%)
	Untreated ADHD	15,847 (9.9%)	12,033 (13.6%)
	No ADHD	134,131 (83.9%)	70,550 (79.9%)
Mean age	Treated ADHD	10.4	16.0
	Untreated ADHD	10.1	16.3
	No ADHD	9.4	16.1
Sex (% Male)	Treated ADHD	73.5%	70.3%
	Untreated ADHD	68.2%	68.6%
	No ADHD	47.6%	46.9%
Comorbid conditions (% with mood and anxiety disorders)	Treated ADHD	4.5%	9.6%
	Untreated ADHD	3.9%	9.8%
	No ADHD	1.1%	5.3%
Select medications (% with prescriptions for any among second generation antipsychotics, clonidine, modafinil, and guanfacine)	Treated ADHD	19.3%	37.4%
	Untreated ADHD	7.7%	7.2%
	No ADHD	0.4%	3.0%
Household income quintile (% in lowest quintile)	Treated ADHD	21.4%	15.4%
	Untreated ADHD	24.2%	21.3%
	No ADHD	17.5%	16.5%
History of social assistance use (% receiving any in past 5 years)	Treated ADHD	24.9%	11.9%
	Untreated ADHD	32.0%	19.6%
	No ADHD	14.8%	9.6%

*Note*. Full breakdowns for additional variables including NB health zone of residence, school district, program of study, household composition, recent immigration status, other comorbid conditions, and CIMD quintiles for each treatment group are available in Supplemental Tables S1a and S1b.

**Table 3.** GLM Regression Estimates—Mean Report Card Score (AY 2017–2020).

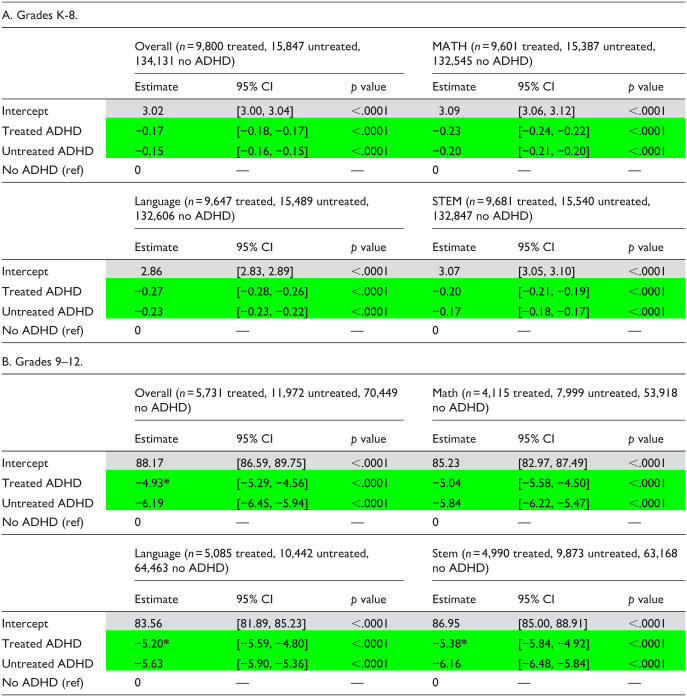

*Note*: These results tables present only the multivariate regression estimates for the diagnosis/treatment group assignment variable (adjusting for all covariates). Full regression results showing estimates associated with each covariate are available in Supplemental Tables S2a to S2h.

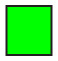
 Significant difference versus No ADHD group.

* Significant difference between Treated and Untreated groups.

Among grade 9 to 12 students, mean overall report card score estimates were significantly lower in the Treated and Untreated groups compared to the No ADHD group (Table 3B). The same pattern was observed across all subject-specific estimates. Direct comparison of Treated and Untreated estimates showed that mean score estimates were significantly higher for the Treated group for overall, STEM, and language scores (Supplemental Tables S5a, S5b, and S5d). Math score estimates also trended toward higher in the Treated group compared to the Untreated group, though this difference was not statistically significant (Supplemental Table S5c). Similar results were noted when academic performance was modeled as proportion of courses failed (score <60%), with significantly higher proportion of courses failed among Treated and Untreated students compared to those without ADHD (Supplemental Tables S6a–S6d), and significantly lower proportion of courses failed for Treated compared to Untreated students overall and across all subject areas (Supplemental Tables S6e–S6h).

### Performance on Standardized Provincial Assessment Exams

Among grade K-8 students (Table 4A), estimates of percentile rank were significantly lower in Treated and Untreated groups compared to the No ADHD group, overall, and across all subject areas. Treated and Untreated estimates did not differ significantly from one another on direct comparison (Supplemental Tables S8a–S8d).

**Table 4.** GLM Regression Estimates—Provincial Assessment Exams Percentile Rank (AY 2017–2019).

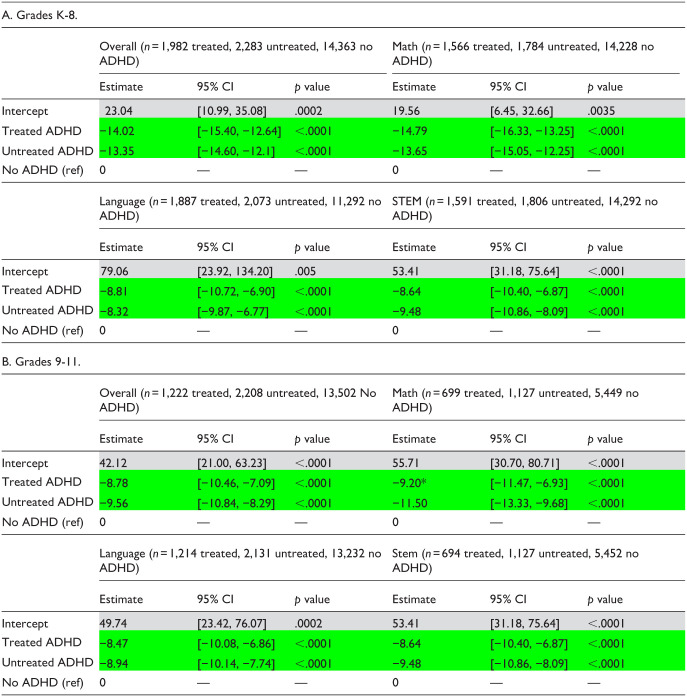

*Note*. These results tables present only the multivariate regression estimates for the diagnosis/treatment group assignment variable (adjusting for all covariates). Full regression results showing estimates associated with each covariate are available in Supplemental Tables 7a to 7h.

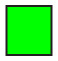
 Significant difference versus No ADHD group.

* Significant difference between Treated and Untreated groups.

Among grade 9 to 11 students (Table 4B), estimates for the Treated and Untreated groups were significantly lower compared to the No ADHD group, overall, and across all subject areas. Direct comparison of Treated and Untreated estimates showed that the estimate for math assessments was significantly higher for the Treated group (Supplemental Tables S8e–S8h).

### Graduation From High School

Regression estimates showed that the likelihood of not graduating from high school on the first attempt was significantly higher in the Untreated group compared to the No ADHD group, while the likelihood in the Treated group did not differ significantly from that in the No ADHD group (Table 5). On direct comparison of the Treated and Untreated estimates, the Untreated group had a significantly higher likelihood of failure to graduate (Supplementary Table S10).

**Table 5.** Logistic Regression Estimates—Likelihood of not graduating from high school on First Attempt (AY 2017–2019; *n* = 771 Treated, 2,059 Untreated, 11,880 No ADHD).

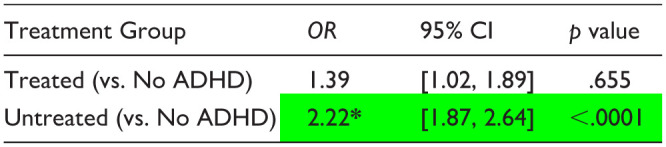

*Note*. These results tables present only the multivariate regression odds ratio estimates for the diagnosis/treatment group assignment variable (adjusting for all covariates). Full regression results showing estimates associated with each covariate are available in Supplemental Table S9.

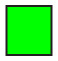
 Significant difference versus No ADHD group.

* Significant difference between Treated and Untreated groups.

### School Attendance

Regression estimates showed that the frequency of any absence among students in grades K-12 was significantly higher in the Untreated group, and significantly lower in the Treated group, compared to the No ADHD group (Table 6A). On direct comparison of Treated and Untreated groups, the Untreated group had significantly higher frequency of any absence (Supplemental Table S12a).

**Table 6.** GLM regression Estimates—School Attendance—Grades K-12 (AY 2018–2020; *n* = 15,264 Treated, 31,912 Untreated, 230814 No ADHD).

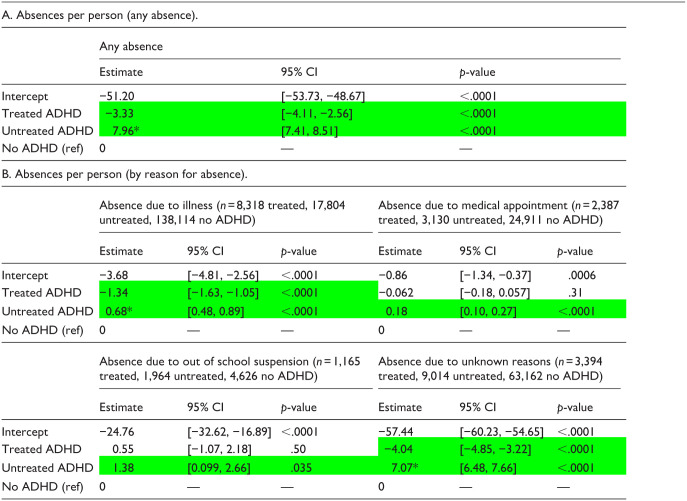

*Note*. These results tables present only the multivariate regression estimates for the diagnosis/treatment group assignment variable (adjusting for all covariates). Full regression results showing estimates associated with each covariate are available in Supplemental Tables S11a to S11e.

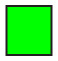
 Significant difference versus No ADHD group.

* Significant difference between Treated and Untreated groups.

Regression estimates for absences by reason for absence showed that frequency of absence for all reasons (illness, medical appointment, out-of-school suspension, and unknown reasons) was significantly higher in the Untreated group compared to the No ADHD group (Table 6B). Frequencies of absence due to illness or unknown reasons in the Treated group were significantly lower compared to the No ADHD group, while frequencies of absence due to medical appointment or suspension in the Treated group did not differ significantly from those in the No ADHD group. On direct comparison of Treated and Untreated groups, the Untreated group had significantly higher frequencies of absence due to illness and due to unknown reasons (Supplemental Tables S12b and S12e).

### Transition to Post-Secondary Education

Regression estimates showed that the Untreated group had significantly higher likelihood of not transitioning to postsecondary education compared to both the No ADHD (Table 7) and Treated (Supplemental Table S14) groups, and the Treated group had significantly lower likelihood of not transitioning compared to the No ADHD group (Table 7).

**Table 7.** Logistic Regression Estimates—Likelihood of Not Transitioning to Post-secondary Education in NB (AY 2018; *n* = 248 Treated, 541 Untreated, 510 No ADHD).

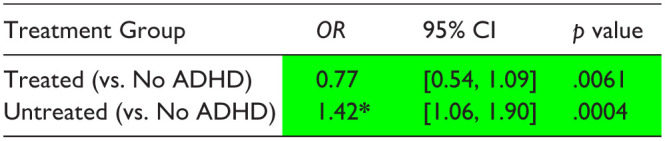

*Note*. These results tables present only the multivariate regression odds ratio estimates for the diagnosis/treatment group assignment variable (adjusting for all covariates). Full regression results showing estimates associated with each covariate are available in Supplemental Table S13.

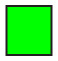
 Significant difference versus No ADHD group.

* Significant difference between Treated and Untreated groups.

## Discussion

To our knowledge, this research represents one of the only population-level studies in Canada to examine the relationship between LAS pharmacotherapy and academic outcomes among students with ADHD using administrative data. The findings demonstrate that ADHD is associated with poorer academic performance and attendance, reduced likelihood of high school graduation and transition to post-secondary education, and that treatment with long-stimulants may mitigate these outcomes, primarily in high school students.

Our findings are consistent with those of several other studies showing improvements in functional academic outcomes associated with stimulant pharmacotherapy (Baweja et al., 2015; Boland et al., 2020; Chang et al., 2019; Jangmo et al., 2019; Kortekaas-Rijlaarsdam et al., 2019; Prasad et al., 2013). For example, a large administrative data study (Jangmo et al., 2019) found that ADHD medication was associated with improved grades and decreased odds of being ineligible to transition to upper school levels. The potential for impact of pharmacotherapy was also illustrated in a recent number needed to treat analysis, which demonstrated that treating as few as three individuals with ADHD with stimulants can prevent one occurrence of grade repetition (Biederman et al., 2019). Previous work has described the benefits of ADHD medication across the dimensions of academic performance (e.g., grade point average), academic skills (e.g., scores on standardized achievement tests), and academic enablers (e.g., classroom behaviors; Baweja et al., 2015). The benefits of LAS demonstrated by our findings similarly span these dimensions: academic performance—report card scores and graduation; academic skills—standardized assessment exam performance; academic enablers—attendance.

Our results show that mean report card scores and provincial assessment exam percentile ranks were significantly lower among students with ADHD compared to students without ADHD across all grade levels. Students in grades 9 to 12 showed improvement in academic performance associated with LAS pharmacotherapy, but this was not evident at lower grades. Several previous studies *have* reported improvements in academic performance associated with stimulant therapy in primary school children, though their outcome measures were different from ours and improvements were small in magnitude (Kortekaas-Rijlaarsdam et al., 2019). Another study using an outcome measure similar to ours (GPA; grade point average) found that stimulant adherence was associated with increased GPA in both elementary and middle school students, though the association was stronger in the middle school group (Marcus & Durkin, 2011). The apparent lack of improvement at lower grades in our study may be related to our choice of outcome measures, that is, stimulant-associated differences in performance may be less detectable within the grading framework for grades K-8 (although we did note significant differences in this metric between untreated students with ADHD and those without ADHD at the K-8 level). Another possibility is that, due to longer time on treatment, older individuals may experience increased functional benefits and increased opportunity for optimization of therapy. Notably, the observed academic benefit of LAS among older students differed by subject area—a finding reported in previous studies (Kortekaas-Rijlaarsdam et al., 2019; Scheffler et al., 2009).

We found an increased likelihood of school absence associated with ADHD, consistent with previous studies (Fleming et al., 2017; Niemi et al., 2022). Reasons for increased absence could include illness symptoms associated with ADHD or comorbidities, increased frequency of medical appointments, and suspension associated with ADHD-related behavioral issues. Unexplained absences were also more likely among students with ADHD. Consistent with previous research (Barbaresi et al., 2007), our results also suggest an association between stimulant treatment and reduced absenteeism. Our analysis shows that LAS pharmacotherapy appeared to reduce absence rates to levels similar to or in some cases below that in students without ADHD, although another study found that absenteeism remained elevated in students treated with ADHD medication compared to their peers without ADHD (Fleming et al., 2017). Differences in the degree of impact of ADHD medications on absenteeism noted between our results and those of other studies may be attributable to differences in study design. Excessive absenteeism may negatively impact academic performance and trajectory, so it is possible that the effects of LAS therapy in reducing absenteeism may also mediate other functional academic improvements.

Our findings highlight the potential benefits of early and optimized intervention. A review by Faraone et al. (2015) illustrates the implications of ADHD for mental and physical health, academics and occupation, social disability, and risky behaviors. The psychological dysfunction experienced by those with ADHD, particularly when untreated or sub-optimally treated, is associated with a wide variety of adverse outcomes that negatively impact quality of life, including accidents and injuries, poor relationships, low self esteem, unemployment, and criminality. The benefits of pharmacotherapy in school-aged children suggested by our findings, including improved academic performance, attendance, graduation rates, and post-secondary enrolment, may have a role in mitigating some of the downstream consequences of ADHD observed later in life. For example, we found that Untreated students were significantly more likely to fail to graduate on the first attempt compared to their peers without ADHD, while the likelihood of failure to graduate among Treated students did not differ significantly from that among those without ADHD. These findings have significant public health and economic implications as gold standard treatment of ADHD with LAS appears to mitigate against the consequences of academic failure. Similarly, we found that Untreated students had a significantly higher likelihood of not transitioning to postsecondary education in NB compared with both the Treated and No ADHD groups, while the Treated group had significantly lower likelihood of not transitioning compared to the No ADHD group. These findings suggest that ADHD may negatively impact academic trajectory beyond grade school, and that LAS pharmacotherapy may mitigate this effect. There are limitations to this portion of the analysis however (discussed below), and additional long-term studies focused on implications for employment and socioeconomic status will be critical to solidify our understanding of the downstream impacts of stimulant pharmacotherapy on the lives of individuals with ADHD.

### Study Limitations

Although administrative datasets are useful for providing real world evidence, they are not without limitations. ADHD diagnosis was identified through LAS prescriptions, physician notes in claims data, and academic records indicating a management plan for ADHD. Although our approach was based on rational inference and methods used in previous studies, (Jangmo et al., 2019; Larsson et al., 2013) it has not been validated and may have led to some misclassification. For instance, individuals exclusively treated with non-LAS medications could have been classified as Untreated or No ADHD, though this is unlikely given that LAS are first-line therapy. Additionally, the use of free-text physician billing notes rather than standardized diagnostic codes may have introduced some uncertainty in identifying ADHD cases.

Some potentially important individual and treatment-related factors, such as symptom severity, adherence, and use of academic accommodations, were not accounted for. Similarly, grouping all LAS together without differentiating specific medications or dosages may have limited the ability to capture nuances in treatment effects. Additionally, while short-acting stimulants and other medications have an important role in the treatment of ADHD, they were not the focus of this study. This study was intended and designed to examine academic impacts associated with long-acting stimulants, and therefore common non-LAS ADHD medications were excluded or adjusted for in regression models to avoid confounding effects, such that any observed impacts could be attributed to LAS. Future work examining the academic impacts of other ADHD medications, and of LAS in the context of these medications, is warranted.

The COVID-19 pandemic occurred during the study period, with school closures and disruptions affecting data availability during certain periods and likely impacting patterns of attendance and academic performance. An example of this impact is that attendance could not be evaluated from March to June 2020 when schools were closed. While it was beyond the scope of this study to account for the breadth of possible impacts of the various waves of the pandemic on the outcomes examined, their potential implications should be considered when interpreting the results.

The analysis of postsecondary transitions faced additional limitations. Only NB’s public universities and most of its public colleges were captured, meaning enrollments at private institutions in NB or at institutions outside the province (among students who maintained an NB residential address in administrative data) were misclassified as non-enrollments. A possible mitigating factor, however, is that the majority of NB residents pursuing postsecondary education are expected to do so within NB (Boco et al., 2023).

Despite the aforementioned limitations, the robust analyses and inclusion of extensive variables from a variety of data sources in the regression models provide reassurance on the validity of the findings.

## Conclusion

This study used administrative data to examine the impact of LAS pharmacotherapy on a variety of academic and related outcomes among a large sample of grade school students with ADHD drawn from the entire New Brunswick population.

Our results suggest that treatment with LAS positively impacts several measures of academic success, a finding that is consistent with many but not all previous studies on the topic (Boland et al., 2020). Notably, the observed academic benefits of LAS were mostly limited to high school students. Our findings provide reassurance that gold standard treatment of ADHD may mitigate against the downstream consequences faced by those with untreated ADHD.

Taken together, these findings contribute to our understanding of the impact of LAS pharmacotherapy on a range of functional outcomes associated with ADHD. Future work aimed at assessing the robustness of these findings would be beneficial and may include sensitivity analyses trialing different approaches to defining ADHD diagnosis and treatment groups in administrative data, measures to address the known methodological limitations of the study, such as accounting for non-drug therapy and academic accommodations, and larger sample sizes and longer observation windows as data availability improves.

## Supplemental Material

sj-docx-1-jad-10.1177_10870547251378169 – Supplemental material for Academic Outcomes in Primary and Secondary School Students Prescribed Long-Acting Stimulants for ADHD Management Supplemental material, sj-docx-1-jad-10.1177_10870547251378169 for Academic Outcomes in Primary and Secondary School Students Prescribed Long-Acting Stimulants for ADHD Management by Chris Folkins, Chandy Somayaji, Simerpal K. Gill and James Ted McDonald in Journal of Attention Disorders

sj-docx-10-jad-10.1177_10870547251378169 – Supplemental material for Academic Outcomes in Primary and Secondary School Students Prescribed Long-Acting Stimulants for ADHD Management Supplemental material, sj-docx-10-jad-10.1177_10870547251378169 for Academic Outcomes in Primary and Secondary School Students Prescribed Long-Acting Stimulants for ADHD Management by Chris Folkins, Chandy Somayaji, Simerpal K. Gill and James Ted McDonald in Journal of Attention Disorders

sj-docx-11-jad-10.1177_10870547251378169 – Supplemental material for Academic Outcomes in Primary and Secondary School Students Prescribed Long-Acting Stimulants for ADHD Management Supplemental material, sj-docx-11-jad-10.1177_10870547251378169 for Academic Outcomes in Primary and Secondary School Students Prescribed Long-Acting Stimulants for ADHD Management by Chris Folkins, Chandy Somayaji, Simerpal K. Gill and James Ted McDonald in Journal of Attention Disorders

sj-docx-12-jad-10.1177_10870547251378169 – Supplemental material for Academic Outcomes in Primary and Secondary School Students Prescribed Long-Acting Stimulants for ADHD Management Supplemental material, sj-docx-12-jad-10.1177_10870547251378169 for Academic Outcomes in Primary and Secondary School Students Prescribed Long-Acting Stimulants for ADHD Management by Chris Folkins, Chandy Somayaji, Simerpal K. Gill and James Ted McDonald in Journal of Attention Disorders

sj-docx-13-jad-10.1177_10870547251378169 – Supplemental material for Academic Outcomes in Primary and Secondary School Students Prescribed Long-Acting Stimulants for ADHD Management Supplemental material, sj-docx-13-jad-10.1177_10870547251378169 for Academic Outcomes in Primary and Secondary School Students Prescribed Long-Acting Stimulants for ADHD Management by Chris Folkins, Chandy Somayaji, Simerpal K. Gill and James Ted McDonald in Journal of Attention Disorders

sj-docx-14-jad-10.1177_10870547251378169 – Supplemental material for Academic Outcomes in Primary and Secondary School Students Prescribed Long-Acting Stimulants for ADHD Management Supplemental material, sj-docx-14-jad-10.1177_10870547251378169 for Academic Outcomes in Primary and Secondary School Students Prescribed Long-Acting Stimulants for ADHD Management by Chris Folkins, Chandy Somayaji, Simerpal K. Gill and James Ted McDonald in Journal of Attention Disorders

sj-docx-15-jad-10.1177_10870547251378169 – Supplemental material for Academic Outcomes in Primary and Secondary School Students Prescribed Long-Acting Stimulants for ADHD Management Supplemental material, sj-docx-15-jad-10.1177_10870547251378169 for Academic Outcomes in Primary and Secondary School Students Prescribed Long-Acting Stimulants for ADHD Management by Chris Folkins, Chandy Somayaji, Simerpal K. Gill and James Ted McDonald in Journal of Attention Disorders

sj-docx-2-jad-10.1177_10870547251378169 – Supplemental material for Academic Outcomes in Primary and Secondary School Students Prescribed Long-Acting Stimulants for ADHD Management Supplemental material, sj-docx-2-jad-10.1177_10870547251378169 for Academic Outcomes in Primary and Secondary School Students Prescribed Long-Acting Stimulants for ADHD Management by Chris Folkins, Chandy Somayaji, Simerpal K. Gill and James Ted McDonald in Journal of Attention Disorders

sj-docx-3-jad-10.1177_10870547251378169 – Supplemental material for Academic Outcomes in Primary and Secondary School Students Prescribed Long-Acting Stimulants for ADHD Management Supplemental material, sj-docx-3-jad-10.1177_10870547251378169 for Academic Outcomes in Primary and Secondary School Students Prescribed Long-Acting Stimulants for ADHD Management by Chris Folkins, Chandy Somayaji, Simerpal K. Gill and James Ted McDonald in Journal of Attention Disorders

sj-docx-4-jad-10.1177_10870547251378169 – Supplemental material for Academic Outcomes in Primary and Secondary School Students Prescribed Long-Acting Stimulants for ADHD Management Supplemental material, sj-docx-4-jad-10.1177_10870547251378169 for Academic Outcomes in Primary and Secondary School Students Prescribed Long-Acting Stimulants for ADHD Management by Chris Folkins, Chandy Somayaji, Simerpal K. Gill and James Ted McDonald in Journal of Attention Disorders

sj-docx-5-jad-10.1177_10870547251378169 – Supplemental material for Academic Outcomes in Primary and Secondary School Students Prescribed Long-Acting Stimulants for ADHD Management Supplemental material, sj-docx-5-jad-10.1177_10870547251378169 for Academic Outcomes in Primary and Secondary School Students Prescribed Long-Acting Stimulants for ADHD Management by Chris Folkins, Chandy Somayaji, Simerpal K. Gill and James Ted McDonald in Journal of Attention Disorders

sj-docx-6-jad-10.1177_10870547251378169 – Supplemental material for Academic Outcomes in Primary and Secondary School Students Prescribed Long-Acting Stimulants for ADHD Management Supplemental material, sj-docx-6-jad-10.1177_10870547251378169 for Academic Outcomes in Primary and Secondary School Students Prescribed Long-Acting Stimulants for ADHD Management by Chris Folkins, Chandy Somayaji, Simerpal K. Gill and James Ted McDonald in Journal of Attention Disorders

sj-docx-7-jad-10.1177_10870547251378169 – Supplemental material for Academic Outcomes in Primary and Secondary School Students Prescribed Long-Acting Stimulants for ADHD Management Supplemental material, sj-docx-7-jad-10.1177_10870547251378169 for Academic Outcomes in Primary and Secondary School Students Prescribed Long-Acting Stimulants for ADHD Management by Chris Folkins, Chandy Somayaji, Simerpal K. Gill and James Ted McDonald in Journal of Attention Disorders

sj-docx-8-jad-10.1177_10870547251378169 – Supplemental material for Academic Outcomes in Primary and Secondary School Students Prescribed Long-Acting Stimulants for ADHD Management Supplemental material, sj-docx-8-jad-10.1177_10870547251378169 for Academic Outcomes in Primary and Secondary School Students Prescribed Long-Acting Stimulants for ADHD Management by Chris Folkins, Chandy Somayaji, Simerpal K. Gill and James Ted McDonald in Journal of Attention Disorders

sj-docx-9-jad-10.1177_10870547251378169 – Supplemental material for Academic Outcomes in Primary and Secondary School Students Prescribed Long-Acting Stimulants for ADHD Management Supplemental material, sj-docx-9-jad-10.1177_10870547251378169 for Academic Outcomes in Primary and Secondary School Students Prescribed Long-Acting Stimulants for ADHD Management by Chris Folkins, Chandy Somayaji, Simerpal K. Gill and James Ted McDonald in Journal of Attention Disorders
