# Supplementary material for: Academic Outcomes in Primary and Secondary School Students Prescribed Long-Acting Stimulants for ADHD Management
Source: J Atten Disord. 2025 Oct 7;30(4):493–505. doi: 10.1177/10870547251378169 (PMC12953683; doi:10.1177/10870547251378169)
Supplement: sj-docx-1-jad-10.1177_10870547251378169 – Supplemental material for Academic Outcomes in Primary and Secondary School Students Prescribed Long-Acting Stimulants for ADHD Management [file sj-docx-1-jad-10.1177_10870547251378169.docx]

**Supplementary Table S1a. Full background characteristics for grades K-8 report card score cohorts (AY 2017 – 2020)**

|  | **Overall cohort** | **Treated ADHD** | **Untreated ADHD** | **No ADHD** |
| --- | --- | --- | --- | --- |
| **n** | **159778** | **9800** | **15847** | **134131** |
| **Age** |  |  |  |  |
| Age (mean, median) | 9.6,9.5 | 10.4,10.5 | 10.1,10.3 | 9.4,9.4 |
| **Sex** |  |  |  |  |
| M | 81806(51.2%) | 7199(73.5%) | 10802(68.2%) | 63805(47.6%) |
| F | 77972(48.8%) | 2601(26.5%) | 5045(31.8%) | 70326(52.4%) |
| **NB Health Zone of residence** |  |  |  |  |
| Zone 1 | 43732(27.4%) | 3275(33.4%) | 3734(23.6%) | 36723(27.4%) |
| Zone 2 | 45673(28.6%) | 2613(26.7%) | 4777(30.1%) | 38283(28.5%) |
| Zone 3 | 44753(28%) | 2101(21.4%) | 4879(30.8%) | 37773(28.2%) |
| Zone 4 | 5132(3.2%) | 279(2.9%) | 419(2.6%) | 4434(3.3%) |
| Zone 5 | 3349(2.1%) | 229(2.3%) | 407(2.6%) | 2713(2%) |
| Zone 6 | 8951(5.6%) | 888(9.1%) | 927(5.9%) | 7136(5.3%) |
| Zone 7 | 8188(5.1%) | 415(4.2%) | 704(4.4%) | 7069(5.3%) |
| **School District** |  |  |  |  |
| Anglophone | 130814(81.9%) | 7751(79.1%) | 13789(87%) | 109274(81.5%) |
| Francophone | 28964(18.1%) | 2049(20.9%) | 2058(13%) | 24857(18.5%) |
| **Program of study** |  |  |  |  |
| English | 110967(69.5%) | 6547(66.8%) | 12175(76.8%) | 92245(68.8%) |
| French | 29350(18.4%) | 2107(21.5%) | 2142(13.5%) | 25101(18.7%) |
| French Immersion/Other | 19461(12.2%) | 1146(11.7%) | 1530(9.7%) | 16785(12.5%) |
| **Household composition – Adults (age 22+)** |  |  |  |  |
| No adults in household | 824(0.5%) | 95(1%) | 141(0.9%) | 588(0.4%) |
| One adult in household | 42189(26.4%) | 3159(32.2) | 5589(35.3%) | 33441(24.9%) |
| More than 1 adult in household | 116765(73.1%) | 6546(66.8%) | 10117(63.8%) | 100102(74.6%) |
| **Household composition – Children (age 21 or under)** |  |  |  |  |
| Student is only child in household | 26116(16.4%) | 2110(21.5%) | 3076(19.4%) | 20930(15.6%) |
| Other children in household | 133662(83.6%) | 7690(78.5%) | 12771(80.6%) | 113201(84.4%) |
| **Immigration status** |  |  |  |  |
| Recent immigrant | 6724(4.2%) | 34(0.4%) | 123(0.8%) | 6567(4.9%) |
| Not a recent immigrant | 153054(95.8%) | 9766(99.6%) | 15724(99.2%) | 127564(95.1%) |
| **Comorbid conditions** |  |  |  |  |
| None | 155645(97.4%) | 9270(94.6%) | 15012(94.7%) | 131363(97.9%) |
| Mood & anxiety disorders | 2551(1.6%) | 444(4.5%) | 619(3.9%) | 1488(1.1%) |
| One or more of: asthma, diabetes, epilepsy, schizophrenia | 1608(1%) | 90(0.9%) | 224(1.4%) | 1294(1%) |
| **Select medications** |  |  |  |  |
| None | 156167(97.7%) | 7907(80.7%) | 14625(92.3%) | 133635(99.6%) |
| One or more | 3611(2.3%) | 1893(19.3%) | 1222(7.7%) | 496(0.4%) |
| **Household income quintile** |  |  |  |  |
| 1 (lowest income) | 29416(18.4%) | 2101(21.4%) | 3835(24.2%) | 23480(17.5%) |
| 2 | 29128(18.2%) | 1746(17.8%) | 3276(20.7%) | 24106(18%) |
| 3 | 31228(19.5%) | 1960(20%) | 3038(19.2%) | 26230(19.6%) |
| 4 | 33824(21.2%) | 2047(20.9%) | 2988(18.9%) | 28789(21.5%) |
| 5 (highest income) | 36182(22.7%) | 1946(19.9%) | 2710(17.1%) | 31526(23.5%) |
| **Canadian Index of Multiple Deprivation**  **– Residential Instability (quintiles)** |  |  |  |  |
| 1 (least deprived) | 40053(25.1%) | 2099(21.4%) | 3267(20.6%) | 34687(25.9%) |
| 2 | 40122(25.1%) | 2290(23.4%) | 3636(22.9%) | 34196(25.5%) |
| 3 | 34052(21.3%) | 2196(22.4%) | 3258(20.6%) | 28598(21.3%) |
| 4 | 25909(16.2%) | 1873(19.1%) | 3005(19%) | 21031(15.7%) |
| 5 (most deprived) | 19642(12.3%) | 1342(13.7%) | 2681(16.9%) | 15619(11.6%) |
| **CIMD – Economic Dependency (quintiles)** |  |  |  |  |
| 1 (least deprived) | 27055(16.9%) | 1710(17.5%) | 2363(14.9%) | 22982(17.1%) |
| 2 | 26335(16.5%) | 1563(16%) | 2470(15.6%) | 22302(16.6%) |
| 3 | 31993(20%) | 1893(19.3%) | 3228(20.4%) | 26872(20%) |
| 4 | 36284(22.7%) | 2221(22.7%) | 3670(23.2%) | 30393(22.7%) |
| 5 (most deprived) | 38111(23.9%) | 2413(24.6%) | 4116(26%) | 31582(23.6%) |
| **CIMD – Ethno-Cultural Composition (quintiles)** |  |  |  |  |
| 1 (least deprived) | 66225(41.5%) | 3997(40.8%) | 6411(40.5%) | 55817(41.6%) |
| 2 | 56587(35.4%) | 3606(36.8%) | 5530(34.9%) | 47451(35.4%) |
| 3 | 23359(14.6%) | 1394(14.2%) | 2309(14.6%) | 19656(14.7%) |
| 4 | 9017(5.6%) | 574(5.9%) | 1036(6.5%) | 7407(5.5%) |
| 5 (most deprived) | 4590(2.9%) | 229(2.3%) | 561(3.5%) | 3800(2.8%) |
| **CIMD – Situational Vulnerability (quintiles)** |  |  |  |  |
| 1 (least deprived) | 33332(20.9%) | 1883(19.2%) | 2592(16.4%) | 28857(21.5%) |
| 2 | 30668(19.2%) | 1812(18.5%) | 2918(18.4%) | 25938(19.3%) |
| 3 | 23411(14.7%) | 1418(14.5%) | 2153(13.6%) | 19840(14.8%) |
| 4 | 35448(22.2%) | 2061(21%) | 3716(23.5%) | 29671(22.1%) |
| 5 (most deprived) | 36919(23.1%) | 2626(26.8%) | 4468(28.2%) | 29825(22.2%) |
| **Social Assistance** |  |  |  |  |
| None in past 5 years | 132394(82.9%) | 7363(75.1%) | 10782(68%) | 114249(85.2%) |
| Any in past 5 years | 27384(17.1%) | 2437(24.9%) | 5065(32%) | 19882(14.8%) |

**Supplementary Table S1b. Full background characteristics for grades 9-12 report card score cohorts (AY 2017 – 2020)**

|  | **Overall cohort** | **Treated ADHD** | **Untreated ADHD** | **No ADHD** |
| --- | --- | --- | --- | --- |
| **n** | **88327** | **5744** | **12033** | **70550** |
| **Age** |  |  |  |  |
| Age (mean, median) | 16.14,16.13 | 16.01,15.96 | 16.26,16.21 | 16.13,16.13 |
| **Sex** |  |  |  |  |
| M | 45390(51.4%) | 4038(70.3%) | 8261(68.6%) | 33091(46.9%) |
| F | 42937(48.6%) | 1706(29.7%) | 3772(31.4%) | 37459(53.1%) |
| **NB Health Zone of residence** |  |  |  |  |
| Zone 1 | 24700(28%) | 1756(30.6%) | 3108(25.8%) | 19836(28.1%) |
| Zone 2 | 21935(24.8%) | 1137(19.8%) | 3078(25.6%) | 17720(25.1%) |
| Zone 3 | 21466(24.3%) | 1269(22.1%) | 2781(23.1%) | 17416(24.7%) |
| Zone 4 | 5177(5.9%) | 290(5.1%) | 708(5.9%) | 4179(5.9%) |
| Zone 5 | 2611(3%) | 224(3.9%) | 381(3.2%) | 2006(2.8%) |
| Zone 6 | 7404(8.4%) | 823(14.3%) | 1370(11.4%) | 5211(7.4%) |
| Zone 7 | 5034(5.7%) | 245(4.3%) | 607(5%) | 4182(5.9%) |
| **School District** |  |  |  |  |
| Anglophone | 63751(72.2%) | 3539(61.6%) | 8620(71.6%) | 51592(73.1%) |
| Francophone | 24576(27.8%) | 2205(38.4%) | 3413(28.4%) | 18958(26.9%) |
| **Program of study** |  |  |  |  |
| English | 38365(43.4%) | 2592(45.1%) | 6805(56.6%) | 28968(41.1%) |
| French | 24356(27.6%) | 2173(37.8%) | 3186(26.5%) | 18997(26.9%) |
| French Immersion | 24921(28.2%) | 910(15.8%) | 1746(14.5%) | 22265(31.6%) |
| Other | 685(0.8%) | 69(1.2%) | 296(2.5%) | 320(0.5%) |
| **Household composition – Adults (age 22+)** |  |  |  |  |
| No adults in household | 1356(1.5%) | 100(1.7%) | 373(3.1%) | 883(1.3%) |
| One adult in household | 17168(19.4%) | 1319(23%) | 3159(26.3%) | 12690(18%) |
| More than 1 adult in household | 69803(79%) | 4325(75.3%) | 8501(70.7%) | 56977(80.8%) |
| **Household composition – Children (age 21 or under)** |  |  |  |  |
| Student is only child in household | 21702(24.6%) | 1738(30.3%) | 3498(29.1%) | 16466(23.3%) |
| Other children in household | 66625(75.4%) | 4006(69.7%) | 8535(70.9%) | 54084(76.7%) |
| **Immigration status** |  |  |  |  |
| Recent immigrant | 2525(2.9%) | 16(0.3%) | 36(0.3%) | 2473(3.5%) |
| Not a recent immigrant | 85802(97.1%) | 5728(99.7%) | 11997(99.7%) | 68077(96.5%) |
| **Comorbid conditions** |  |  |  |  |
| None | 82264(93.1%) | 5153(89.7%) | 10730(89.2%) | 66381(94.1%) |
| Mood & anxiety disorders | 5487(6.2%) | 553(9.6%) | 1180(9.8%) | 3754(5.3%) |
| One or more of: asthma, diabetes, epilepsy, schizophrenia | 629(0.7%) | 43(0.8%) | 146(1.2%) | 440(0.6%) |
| **Select medications** |  |  |  |  |
| None | 83206(94.2%) | 3597(62.6%) | 11163(92.3%) | 68446(97%) |
| One or more | 5121(5.8%) | 2147(37.4%) | 870(7.2%) | 2104(3%) |
| **Household income quintile** |  |  |  |  |
| 1 (lowest income) | 15104(17.1%) | 885(15.4%) | 2561(21.3%) | 11658(16.5%) |
| 2 | 16079(18.2%) | 975(17%) | 2381(19.8%) | 12723(18%) |
| 3 | 17151(19.4%) | 1090(19%) | 2425(20.2%) | 13636(19.3%) |
| 4 | 18448(20.9%) | 1263(22%) | 2290(19%) | 14895(21.1%) |
| 5 (highest income) | 21545(24.4%) | 1531(26.6%) | 2376(19.8%) | 17638(25%) |
| **Canadian Index of Multiple Deprivation – Residential Instability (quintiles)** |  |  |  |  |
| 1 (least deprived) | 22077(25%) | 1343(23.4%) | 2581(21.5%) | 18153(25.7%) |
| 2 | 22966(26%) | 1431(24.9%) | 2968(24.7%) | 18567(26.3%) |
| 3 | 19929(22.6%) | 1467(25.5%) | 2757(22.9%) | 15705(22.3%) |
| 4 | 14669(16.6%) | 969(16.9%) | 2345(19.5%) | 11355(16.1%) |
| 5 (most deprived) | 8686(9.8%) | 534(9.3%) | 1382(11.5%) | 6770(9.6%) |
| **CIMD – Economic Dependency (quintiles)** |  |  |  |  |
| 1 (least deprived) | 13856(15.7%) | 1033(18%) | 1620(13.5%) | 11203(15.9%) |
| 2 | 13813(15.6%) | 923(16.1%) | 1698(14.1%) | 11192(15.9%) |
| 3 | 17075(19.3%) | 1028(17.9%) | 2288(19%) | 13759(19.5%) |
| 4 | 19958(22.6%) | 1202(20.9%) | 2872(23.9%) | 15884(22.5%) |
| 5 (most deprived) | 23625(26.8%) | 1558(27.1%) | 3555(29.5%) | 18512(26.2%) |
| **CIMD – Ethno-Cultural Composition (quintiles)** |  |  |  |  |
| 1 (least deprived) | 37812(42.8%) | 2502(43.6%) | 5263(43.7%) | 30047(42.6%) |
| 2 | 32295(36.6%) | 2115(36.8%) | 4379(36.4%) | 25801(36.6%) |
| 3 | 11990(13.6%) | 758(13.2%) | 1606(13.4%) | 9626(13.6%) |
| 4 | 4427(5%) | 277(4.8%) | 522(4.3%) | 3628(5.1%) |
| 5 (most deprived) | 1803(2%) | 92(1.6%) | 263(2.2%) | 1448(2.1) |
| **CIMD – Situational Vulnerability (quintiles)** |  |  |  |  |
| 1 (least deprived) | 18590(21.1%) | 1268(22.1%) | 1988(16.5%) | 15334(21.7%) |
| 2 | 15615(17.7%) | 1051(18.3%) | 1926(16%) | 12638(17.9%) |
| 3 | 12215(13.8%) | 718(12.5%) | 1632(13.6%) | 9865(14%) |
| 4 | 19482(22.1%) | 1250(21.8%) | 2729(22.7%) | 15503(22%) |
| 5 (most deprived) | 22425(25.4) | 1457(25.4%) | 3758(31.2%) | 17210(24.4%) |
| **Social Assistance** |  |  |  |  |
| None in past 5 years | 78486(88.9%) | 5059(88.1%) | 9676(80.4%) | 63751(90.4%) |
| Any in past 5 years | 9841(11.1%) | 685(11.9%) | 2357(19.6%) | 6799(9.6%) |
