# Supplementary material for: Academic Outcomes in Primary and Secondary School Students Prescribed Long-Acting Stimulants for ADHD Management
Source: J Atten Disord. 2025 Oct 7;30(4):493–505. doi: 10.1177/10870547251378169 (PMC12953683; doi:10.1177/10870547251378169)
Supplement: sj-docx-10-jad-10.1177_10870547251378169 – Supplemental material for Academic Outcomes in Primary and Secondary School Students Prescribed Long-Acting Stimulants for ADHD Management [file sj-docx-10-jad-10.1177_10870547251378169.docx]

**Supplementary Table S10. Logistic regression estimates - Likelihood of not graduating from high school on first attempt (AY 2017-2019) (Untreated group as reference)**

| **Odds Ratio Estimates** | | | |
| --- | --- | --- | --- |
| **Effect** | **Point Estimate** | **95% Wald**  **Confidence Limits** | |
| **Treated ADHD vs Untreated ADHD** | 0.595 | 0.429 | 0.825 |
| **Age** | 1.255 | 1.055 | 1.493 |
| **Male vs Female** | 0.958 | 0.722 | 1.272 |
| **Household income quintile Q2 vs Q1 (lowest income)** | 1.18 | 0.757 | 1.841 |
| **Household income quintile Q3 vs Q1 (lowest income)** | 1.002 | 0.593 | 1.693 |
| **Household income quintile Q4 vs Q1 (lowest income)** | 1.184 | 0.687 | 2.04 |
| **Household income quintile Q5 (highest income) vs Q1 (lowest income)** | 1.063 | 0.58 | 1.949 |
| **NB Health Zone 2 vs Zone 1** | 0.669 | 0.46 | 0.973 |
| **NB Health Zone 3 vs Zone 1** | 0.854 | 0.588 | 1.241 |
| **NB Health Zone 4 vs Zone 1** | 0.781 | 0.352 | 1.732 |
| **NB Health Zone 5 vs Zone 1** | 0.726 | 0.325 | 1.62 |
| **NB Health Zone 6 vs Zone 1** | 0.542 | 0.304 | 0.967 |
| **NB Health Zone 7 vs Zone 1** | 1.202 | 0.667 | 2.168 |
| **Comorbid conditions – Mood & anxiety disorders - yes vs no** | 1.269 | 0.694 | 2.32 |
| **Comorbid conditions – One or more of: asthma, diabetes, epilepsy, schizophrenia - yes vs no** | 0.956 | 0.4 | 2.285 |
| **Select medications - yes vs no** | 3.256 | 2.293 | 4.624 |
| **School District - Anglophone vs Francophone** | 0.855 | 0.366 | 1.996 |
| **CIMD - Residential Instability Q2 vs Q1 (least deprived)** | 0.781 | 0.525 | 1.163 |
| **CIMD - Residential Instability Q3 vs Q1 (least deprived)** | 1.027 | 0.679 | 1.555 |
| **CIMD - Residential Instability Q4 vs Q1 (least deprived)** | 1.126 | 0.7 | 1.811 |
| **CIMD - Residential Instability Q5 (most deprived) vs Q1 (least deprived)** | 1.915 | 1.059 | 3.463 |
| **CIMD - Economic Dependency Q2 vs Q1 (least deprived)** | 0.682 | 0.406 | 1.146 |
| **CIMD - Economic Dependency Q3 vs Q1 (least deprived)** | 0.849 | 0.512 | 1.411 |
| **CIMD - Economic Dependency Q4 vs Q1 (least deprived)** | 0.812 | 0.485 | 1.36 |
| **CIMD - Economic Dependency Q5 (most deprived) vs Q1 (least deprived)** | 0.798 | 0.472 | 1.351 |
| **CIMD - Ethnocultural Composition Q2 vs Q1 (least deprived)** | 1.207 | 0.904 | 1.612 |
| **CIMD - Ethnocultural Composition Q3 vs Q1 (least deprived)** | 0.695 | 0.434 | 1.114 |
| **CIMD - Ethnocultural Composition Q4 vs Q1 (least deprived)** | 0.534 | 0.252 | 1.131 |
| **CIMD - Ethnocultural Composition Q5 (most deprived) vs Q1 (least deprived)** | 1.281 | 0.519 | 3.162 |
| **CIMD - Situational Vulnerability Q2 vs Q1 (least deprived)** | 0.949 | 0.578 | 1.558 |
| **CIMD - Situational Vulnerability Q3 vs Q1 (least deprived)** | 1.384 | 0.801 | 2.392 |
| **CIMD - Situational Vulnerability Q4 vs Q1 (least deprived)** | 1.192 | 0.713 | 1.994 |
| **CIMD - Situational Vulnerability Q5 (most deprived) vs Q1 (least deprived)** | 0.961 | 0.549 | 1.682 |
| **Social Assistance – any received in past 5 years - yes vs no** | 2.012 | 1.459 | 2.777 |
| **Program of Study - French Immersion/Other vs English** | 0.241 | 0.133 | 0.44 |
| **Program of Study - French vs English** | 0.231 | 0.094 | 0.572 |
| **Household composition – Adults (age 22+) – No adults in household vs More than one adult in household** | 2.448 | 1.551 | 3.864 |
| **Household composition – Adults (age 22+) – One adult in household vs More than one adult in household** | 1.456 | 1.062 | 1.996 |
| **Household composition - Children (age 21 or under) – Student is only child in household vs Other children in household** | 1.304 | 0.984 | 1.726 |
| **Recent immigrant vs Not a recent immigrant** | <0.001 | <0.001 | >999.999 |
