# Supplementary material for: Academic Outcomes in Primary and Secondary School Students Prescribed Long-Acting Stimulants for ADHD Management
Source: J Atten Disord. 2025 Oct 7;30(4):493–505. doi: 10.1177/10870547251378169 (PMC12953683; doi:10.1177/10870547251378169)
Supplement: sj-docx-11-jad-10.1177_10870547251378169 – Supplemental material for Academic Outcomes in Primary and Secondary School Students Prescribed Long-Acting Stimulants for ADHD Management [file sj-docx-11-jad-10.1177_10870547251378169.docx]

**Supplementary Table S11a. GLM regression estimates – Absences per person (any absence) – Grades K-12 (AY 2018-2020)**

| **Parameter** | **Estimate** | **Standard**  **Error** | **t Value** | **Pr > \|t\|** | **95% Confidence Limits** | |
| --- | --- | --- | --- | --- | --- | --- |
| **Intercept** | -51.2014 | 1.2925 | -39.6100 | <.0001 | -53.7347 | -48.6681 |
| **Treated ADHD** | -3.3339 | 0.3961 | -8.4200 | <.0001 | -4.1102 | -2.5577 |
| **Untreated ADHD** | 7.9617 | 0.2813 | 28.3000 | <.0001 | 7.4103 | 8.5131 |
| **No ADHD (REF)** | 0.0000 | . | . | . | . | . |
| **Age** | 4.4549 | 0.0218 | 204.6600 | <.0001 | 4.4123 | 4.4976 |
| **Observation Time** | 0.0819 | 0.0014 | 59.6600 | <.0001 | 0.0792 | 0.0846 |
| **Male** | -0.1531 | 0.1738 | -0.8800 | 0.3784 | -0.4937 | 0.1875 |
| **Female (REF)** | 0.0000 | . | . | . | . | . |
| **Household income quintile Q2** | -2.5137 | 0.3312 | -7.5900 | <.0001 | -3.1629 | -1.8645 |
| **Household income quintile Q3** | -2.5283 | 0.3609 | -7.0100 | <.0001 | -3.2357 | -1.8210 |
| **Household income quintile Q4** | -3.0788 | 0.3871 | -7.9500 | <.0001 | -3.8374 | -2.3201 |
| **Household income quintile Q5 (highest income)** | -3.4054 | 0.4189 | -8.1300 | <.0001 | -4.2264 | -2.5843 |
| **Household income quintile Q1 (lowest income) (REF)** | 0.0000 | . | . | . | . | . |
| **NB Health Zone 2** | 11.4434 | 0.2572 | 44.4900 | <.0001 | 10.9393 | 11.9475 |
| **NB Health Zone 3** | 2.8089 | 0.2522 | 11.1400 | <.0001 | 2.3146 | 3.3032 |
| **NB Health Zone 4** | 0.1688 | 0.4195 | 0.4000 | 0.6875 | -0.6535 | 0.9910 |
| **NB Health Zone 5** | 0.1590 | 0.5639 | 0.2800 | 0.7779 | -0.9462 | 1.2643 |
| **NB Health Zone 6** | -3.2355 | 0.3848 | -8.4100 | <.0001 | -3.9898 | -2.4812 |
| **NB Health Zone 7** | 0.3280 | 0.4247 | 0.7700 | 0.4399 | -0.5044 | 1.1604 |
| **NB Health Zone 1 (REF)** | 0.0000 | . | . | . | . | . |
| **Comorbid conditions - Mood & anxiety disorders (yes)** | 29.6807 | 0.8321 | 35.6700 | <.0001 | 28.0497 | 31.3116 |
| **Comorbid conditions - Mood & anxiety disorders (no) (REF)** | 0.0000 | . | . | . | . | . |
| **Comorbid conditions – One or more of: asthma, diabetes, epilepsy, schizophrenia (yes)** | 3.0438 | 1.1668 | 2.6100 | 0.0091 | 0.7570 | 5.3307 |
| **Comorbid conditions – One or more of: asthma, diabetes, epilepsy, schizophrenia (no) (REF)** | 0.0000 | . | . | . | . | . |
| **Select medications (one or more)** | 18.8242 | 0.5474 | 34.3900 | <.0001 | 17.7512 | 19.8971 |
| **Select medications (none) (REF)** | 0.0000 | . | . | . | . | . |
| **School District - Anglophone** | 4.8786 | 1.0606 | 4.6000 | <.0001 | 2.7999 | 6.9572 |
| **School District – Francophone (REF)** | 0.0000 | . | . | . | . | . |
| **CIMD - Residential Instability Q2** | 0.7120 | 0.2567 | 2.7700 | 0.0055 | 0.2088 | 1.2151 |
| **CIMD - Residential Instability Q3** | 1.5977 | 0.2750 | 5.8100 | <.0001 | 1.0586 | 2.1367 |
| **CIMD - Residential Instability Q4** | 2.9032 | 0.3207 | 9.0500 | <.0001 | 2.2747 | 3.5317 |
| **CIMD – Residential Instability Q5 (most deprived)** | 2.1702 | 0.4294 | 5.0500 | <.0001 | 1.3285 | 3.0118 |
| **CIMD - Residential Instability Q1 (least deprived) (REF)** | 0.0000 | . | . | . | . | . |
| **CIMD - Economic Dependency Q2** | -0.5955 | 0.3217 | -1.8500 | 0.0642 | -1.2260 | 0.0350 |
| **CIMD - Economic Dependency Q3** | -0.5537 | 0.3238 | -1.7100 | 0.0873 | -1.1884 | 0.0809 |
| **CIMD - Economic Dependency Q4** | -0.3936 | 0.3348 | -1.1800 | 0.2397 | -1.0498 | 0.2625 |
| **CIMD - Economic Dependency Q5 (most deprived)** | -1.8042 | 0.3446 | -5.2400 | <.0001 | -2.4796 | -1.1287 |
| **CIMD - Economic Dependency Q1 (least deprived) (REF)** | 0.0000 | . | . | . | . | . |
| **CIMD - Ethnocultural Composition Q2** | 0.0255 | 0.2000 | 0.1300 | 0.8987 | -0.3665 | 0.4174 |
| **CIMD - Ethnocultural Composition Q3** | -1.0439 | 0.2873 | -3.6300 | 0.0003 | -1.6070 | -0.4808 |
| **CIMD - Ethnocultural Composition Q4** | -2.1516 | 0.4243 | -5.0700 | <.0001 | -2.9832 | -1.3200 |
| **CIMD - Ethnocultural Composition Q5 (most deprived)** | -2.8909 | 0.6283 | -4.6000 | <.0001 | -4.1224 | -1.6593 |
| **CIMD - Ethnocultural Composition Q1 (least deprived) (REF)** | 0.0000 | . | . | . | . | . |
| **CIMD -Situational Vulnerability Q2** | 1.6702 | 0.3095 | 5.4000 | <.0001 | 1.0636 | 2.2768 |
| **CIMD - Situational Vulnerability Q3** | 1.8263 | 0.3451 | 5.2900 | <.0001 | 1.1499 | 2.5028 |
| **CIMD -Situational Vulnerability Q4** | 2.7260 | 0.3387 | 8.0500 | <.0001 | 2.0621 | 3.3899 |
| **CIMD -Situational Vulnerability Q5 (most deprived)** | 5.8088 | 0.3714 | 15.6400 | <.0001 | 5.0810 | 6.5367 |
| **CIMD - Situational Vulnerability Q1 (least deprived) (REF)** | 0.0000 | . | . | . | . | . |
| **Social Assistance (any in past 5 years)** | 12.0601 | 0.2676 | 45.0600 | <.0001 | 11.5355 | 12.5847 |
| **Social Assistance (none in past 5 years) (REF)** | 0.0000 | . | . | . | . | . |
| **Program of Study - French Immersion/Other** | -12.0306 | 0.2220 | -54.1900 | <.0001 | -12.4657 | -11.5955 |
| **Program of Study - French** | -9.4732 | 1.0716 | -8.8400 | <.0001 | -11.5734 | -7.3730 |
| **Program of Study - English (REF)** | 0.0000 | . | . | . | . | . |
| **Household composition – Adults (age 22+) – No adults in household** | -10.0425 | 0.7951 | -12.6300 | <.0001 | -11.6009 | -8.4841 |
| **Household composition – Adults (age 22+) – One adult in household** | 2.9525 | 0.2110 | 13.9900 | <.0001 | 2.5390 | 3.3660 |
| **Household composition – Adults (age 22+) – More than one adult in household (REF)** | 0.0000 | . | . | . | . | . |
| **Household composition – Children (age 21 or under) – Student is only child in household** | 0.8196 | 0.2213 | 3.7000 | 0.0002 | 0.3859 | 1.2533 |
| **Household composition – Children (age 21 or under) – Other children in household (REF)** | 0.0000 | . | . | . | . | . |
| **Recent immigrant** | -8.0340 | 0.4874 | -16.4800 | <.0001 | -8.9893 | -7.0787 |
| **Not a recent immigrant (REF)** | 0.0000 | . | . | . | . | . |

**Supplementary Table S11b. GLM regression estimates – Absences per person due to illness – Grades K-12 (AY 2018-2020)**

| **Parameter** | **Estimate** | **Standard**  **Error** | **t Value** | **Pr > \|t\|** | **95% Confidence Limits** | |
| --- | --- | --- | --- | --- | --- | --- |
| **Intercept** | -3.6844 | 0.5729 | -6.4300 | <.0001 | -4.8072 | -2.5615 |
| **Treated ADHD** | -1.3411 | 0.1492 | -8.9900 | <.0001 | -1.6336 | -1.0487 |
| **Untreated ADHD** | 0.6835 | 0.1033 | 6.6200 | <.0001 | 0.4811 | 0.8859 |
| **No ADHD (REF)** | 0.0000 | . | . | . | . | . |
| **Age** | 0.8363 | 0.0081 | 102.7600 | <.0001 | 0.8203 | 0.8522 |
| **Observation Time** | 0.0058 | 0.0005 | 11.1000 | <.0001 | 0.0047 | 0.0068 |
| **Male** | 0.0247 | 0.0630 | 0.3900 | 0.6944 | -0.0987 | 0.1481 |
| **Female (REF)** | 0.0000 | . | . | . | . | . |
| **Household income quintile Q2** | -0.1681 | 0.1193 | -1.4100 | 0.1586 | -0.4019 | 0.0656 |
| **Household income quintile Q3** | -0.1664 | 0.1310 | -1.2700 | 0.2039 | -0.4232 | 0.0903 |
| **Household income quintile Q4** | -0.1125 | 0.1403 | -0.8000 | 0.4227 | -0.3875 | 0.1625 |
| **Household income quintile Q5 (highest income)** | -0.2625 | 0.1537 | -1.7100 | 0.0876 | -0.5638 | 0.0387 |
| **Household income quintile Q1 (lowest income) (REF)** | 0.0000 | . | . | . | . | . |
| **NB Health Zone 2** | 1.7418 | 0.0905 | 19.2400 | <.0001 | 1.5643 | 1.9192 |
| **NB Health Zone 3** | -0.8482 | 0.0902 | -9.4100 | <.0001 | -1.0249 | -0.6714 |
| **NB Health Zone 4** | -0.2644 | 0.1708 | -1.5500 | 0.1216 | -0.5993 | 0.0704 |
| **NB Health Zone 5** | -0.8270 | 0.2165 | -3.8200 | 0.0001 | -1.2514 | -0.4027 |
| **NB Health Zone 6** | -0.2966 | 0.1468 | -2.0200 | 0.0433 | -0.5843 | -0.0089 |
| **NB Health Zone 7** | -2.3166 | 0.1660 | -13.9600 | <.0001 | -2.6418 | -1.9913 |
| **NB Health Zone 1 (REF)** | 0.0000 | . | . | . | . | . |
| **Comorbid conditions - Mood & anxiety disorders (yes)** | 5.4006 | 0.2804 | 19.2600 | <.0001 | 4.8510 | 5.9503 |
| **Comorbid conditions - Mood & anxiety disorders (no) (REF)** | 0.0000 | . | . | . | . | . |
| **Comorbid conditions – One or more of: asthma, diabetes, epilepsy, schizophrenia (yes)** | 1.6626 | 0.3946 | 4.2100 | <.0001 | 0.8893 | 2.4360 |
| **Comorbid conditions – One or more of: asthma, diabetes, epilepsy, schizophrenia (no) (REF)** | 0.0000 | . | . | . | . | . |
| **Select medications (one or more)** | 3.2015 | 0.2009 | 15.9400 | <.0001 | 2.8077 | 3.5952 |
| **Select medications (none) (REF)** | 0.0000 | . | . | . | . | . |
| **School District - Anglophone** | 0.4576 | 0.5027 | 0.9100 | 0.3627 | -0.5277 | 1.4429 |
| **School District – Francophone (REF)** | 0.0000 | . | . | . | . | . |
| **CIMD - Residential Instability Q2** | -0.0579 | 0.0932 | -0.6200 | 0.5345 | -0.2406 | 0.1248 |
| **CIMD - Residential Instability Q3** | -0.0851 | 0.1005 | -0.8500 | 0.3973 | -0.2821 | 0.1119 |
| **CIMD - Residential Instability Q4** | 0.0650 | 0.1164 | 0.5600 | 0.5764 | -0.1631 | 0.2931 |
| **CIMD – Residential Instability Q5 (most deprived)** | -0.3135 | 0.1560 | -2.0100 | 0.0445 | -0.6193 | -0.0077 |
| **CIMD - Residential Instability Q1 (least deprived) (REF)** | 0.0000 | . | . | . | . | . |
| **CIMD - Economic Dependency Q2** | 0.0898 | 0.1173 | 0.7700 | 0.4438 | -0.1400 | 0.3197 |
| **CIMD - Economic Dependency Q3** | 0.2165 | 0.1173 | 1.8500 | 0.0650 | -0.0135 | 0.4465 |
| **CIMD - Economic Dependency Q4** | 0.1665 | 0.1210 | 1.3800 | 0.1688 | -0.0707 | 0.4037 |
| **CIMD - Economic Dependency Q5 (most deprived)** | 0.3373 | 0.1257 | 2.6800 | 0.0073 | 0.0909 | 0.5836 |
| **CIMD - Economic Dependency Q1 (least deprived) (REF)** | 0.0000 | . | . | . | . | . |
| **CIMD - Ethnocultural Composition Q2** | 0.0410 | 0.0724 | 0.5700 | 0.5714 | -0.1010 | 0.1830 |
| **CIMD - Ethnocultural Composition Q3** | -0.0912 | 0.1043 | -0.8700 | 0.3818 | -0.2957 | 0.1132 |
| **CIMD - Ethnocultural Composition Q4** | -0.2205 | 0.1532 | -1.4400 | 0.1501 | -0.5209 | 0.0798 |
| **CIMD - Ethnocultural Composition Q5 (most deprived)** | -0.3225 | 0.2284 | -1.4100 | 0.1579 | -0.7702 | 0.1252 |
| **CIMD - Ethnocultural Composition Q1 (least deprived) (REF)** | 0.0000 | . | . | . | . | . |
| **CIMD -Situational Vulnerability Q2** | 0.2794 | 0.1105 | 2.5300 | 0.0115 | 0.0628 | 0.4960 |
| **CIMD - Situational Vulnerability Q3** | 0.3458 | 0.1251 | 2.7600 | 0.0057 | 0.1007 | 0.5910 |
| **CIMD -Situational Vulnerability Q4** | 0.1487 | 0.1224 | 1.2100 | 0.2246 | -0.0913 | 0.3887 |
| **CIMD -Situational Vulnerability Q5 (most deprived)** | 0.1113 | 0.1350 | 0.8200 | 0.4099 | -0.1534 | 0.3759 |
| **CIMD - Situational Vulnerability Q1 (least deprived) (REF)** | 0.0000 | . | . | . | . | . |
| **Social Assistance (any in past 5 years)** | 0.7420 | 0.0941 | 7.8800 | <.0001 | 0.5575 | 0.9265 |
| **Social Assistance (none in past 5 years) (REF)** | 0.0000 | . | . | . | . | . |
| **Program of Study - French Immersion/Other** | -0.9939 | 0.0763 | -13.0300 | <.0001 | -1.1435 | -0.8444 |
| **Program of Study - French** | -1.4422 | 0.5050 | -2.8600 | 0.0043 | -2.4320 | -0.4523 |
| **Program of Study - English (REF)** | 0.0000 | . | . | . | . | . |
| **Household composition – Adults (age 22+) – No adults in household** | -1.3845 | 0.3143 | -4.4100 | <.0001 | -2.0004 | -0.7685 |
| **Household composition – Adults (age 22+) – One adult in household** | -0.0948 | 0.0760 | -1.2500 | 0.2120 | -0.2438 | 0.0541 |
| **Household composition – Adults (age 22+) – More than one adult in household (REF)** | 0.0000 | . | . | . | . | . |
| **Household composition – Children (age 21 or under) – Student is only child in household** | 1.2947 | 0.0798 | 16.2200 | <.0001 | 1.1383 | 1.4512 |
| **Household composition – Children (age 21 or under) – Other children in household (REF)** | 0.0000 | . | . | . | . | . |
| **Recent immigrant** | -1.5936 | 0.1878 | -8.4900 | <.0001 | -1.9616 | -1.2256 |
| **Not a recent immigrant (REF)** | 0.0000 | . | . | . | . | . |

**Supplementary Table S11c. GLM regression estimates – Absences per person due to medical appointment – Grades K-12 (AY 2018-2020)**

| **Parameter** | **Estimate** | **Standard**  **Error** | **t Value** | **Pr > \|t\|** | **95% Confidence Limits** | |
| --- | --- | --- | --- | --- | --- | --- |
| **Intercept** | -0.8554 | 0.2484 | -3.4400 | 0.0006 | -1.3423 | -0.3685 |
| **Treated ADHD** | -0.0617 | 0.0605 | -1.0200 | 0.3081 | -0.1804 | 0.0570 |
| **Untreated ADHD** | 0.1845 | 0.0455 | 4.0500 | <.0001 | 0.0953 | 0.2737 |
| **No ADHD (REF)** | 0.0000 | . | . | . | . | . |
| **Age** | 0.2780 | 0.0037 | 74.6600 | <.0001 | 0.2707 | 0.2852 |
| **Observation Time** | 0.0025 | 0.0002 | 10.5800 | <.0001 | 0.0021 | 0.0030 |
| **Male** | -0.2274 | 0.0288 | -7.8800 | <.0001 | -0.2839 | -0.1708 |
| **Female (REF)** | 0.0000 | . | . | . | . | . |
| **Household income quintile Q2** | -0.1247 | 0.0553 | -2.2500 | 0.0242 | -0.2331 | -0.0163 |
| **Household income quintile Q3** | -0.0416 | 0.0605 | -0.6900 | 0.4912 | -0.1601 | 0.0769 |
| **Household income quintile Q4** | 0.1469 | 0.0642 | 2.2900 | 0.0222 | 0.0210 | 0.2728 |
| **Household income quintile Q5 (highest income)** | 0.1946 | 0.0700 | 2.7800 | 0.0055 | 0.0573 | 0.3318 |
| **Household income quintile Q1 (lowest income) (REF)** | 0.0000 | . | . | . | . | . |
| **NB Health Zone 2** | 0.2307 | 0.0417 | 5.5300 | <.0001 | 0.1489 | 0.3125 |
| **NB Health Zone 3** | -0.1331 | 0.0407 | -3.2700 | 0.0011 | -0.2128 | -0.0534 |
| **NB Health Zone 4** | -0.2213 | 0.0791 | -2.8000 | 0.0051 | -0.3763 | -0.0664 |
| **NB Health Zone 5** | -0.8485 | 0.1050 | -8.0800 | <.0001 | -1.0543 | -0.6426 |
| **NB Health Zone 6** | -0.2119 | 0.0655 | -3.2400 | 0.0012 | -0.3402 | -0.0836 |
| **NB Health Zone 7** | -0.9477 | 0.0766 | -12.3800 | <.0001 | -1.0977 | -0.7976 |
| **NB Health Zone 1 (REF)** | 0.0000 | . | . | . | . | . |
| **Comorbid conditions - Mood & anxiety disorders (yes)** | 1.5770 | 0.1133 | 13.9100 | <.0001 | 1.3549 | 1.7992 |
| **Comorbid conditions - Mood & anxiety disorders (no) (REF)** | 0.0000 | . | . | . | . | . |
| **Comorbid conditions – One or more of: asthma, diabetes, epilepsy, schizophrenia (yes)** | 0.8431 | 0.1582 | 5.3300 | <.0001 | 0.5330 | 1.1531 |
| **Comorbid conditions – One or more of: asthma, diabetes, epilepsy, schizophrenia (no) (REF)** | 0.0000 | . | . | . | . | . |
| **Select medications (one or more)** | 1.0083 | 0.0832 | 12.1200 | <.0001 | 0.8452 | 1.1713 |
| **Select medications (none) (REF)** | 0.0000 | . | . | . | . | . |
| **School District - Anglophone** | -0.4750 | 0.2130 | -2.2300 | 0.0257 | -0.8924 | -0.0576 |
| **School District – Francophone (REF)** | 0.0000 | . | . | . | . | . |
| **CIMD - Residential Instability Q2** | 0.1806 | 0.0420 | 4.3000 | <.0001 | 0.0982 | 0.2629 |
| **CIMD - Residential Instability Q3** | 0.2013 | 0.0453 | 4.4400 | <.0001 | 0.1125 | 0.2901 |
| **CIMD - Residential Instability Q4** | 0.0987 | 0.0527 | 1.8700 | 0.0609 | -0.0045 | 0.2019 |
| **CIMD – Residential Instability Q5 (most deprived)** | -0.1036 | 0.0711 | -1.4600 | 0.1451 | -0.2430 | 0.0358 |
| **CIMD - Residential Instability Q1 (least deprived) (REF)** | 0.0000 | . | . | . | . | . |
| **CIMD - Economic Dependency Q2** | 0.0247 | 0.0525 | 0.4700 | 0.6386 | -0.0783 | 0.1277 |
| **CIMD - Economic Dependency Q3** | 0.1756 | 0.0530 | 3.3100 | 0.0009 | 0.0717 | 0.2796 |
| **CIMD - Economic Dependency Q4** | 0.2138 | 0.0546 | 3.9200 | <.0001 | 0.1069 | 0.3208 |
| **CIMD - Economic Dependency Q5 (most deprived)** | 0.4898 | 0.0568 | 8.6300 | <.0001 | 0.3786 | 0.6011 |
| **CIMD - Economic Dependency Q1 (least deprived) (REF)** | 0.0000 | . | . | . | . | . |
| **CIMD - Ethnocultural Composition Q2** | -0.0350 | 0.0330 | -1.0600 | 0.2891 | -0.0998 | 0.0297 |
| **CIMD - Ethnocultural Composition Q3** | -0.0609 | 0.0469 | -1.3000 | 0.1942 | -0.1528 | 0.0310 |
| **CIMD - Ethnocultural Composition Q4** | -0.2319 | 0.0693 | -3.3500 | 0.0008 | -0.3677 | -0.0961 |
| **CIMD - Ethnocultural Composition Q5 (most deprived)** | -0.2839 | 0.1077 | -2.6400 | 0.0084 | -0.4950 | -0.0728 |
| **CIMD - Ethnocultural Composition Q1 (least deprived) (REF)** | 0.0000 | . | . | . | . | . |
| **CIMD -Situational Vulnerability Q2** | 0.1760 | 0.0498 | 3.5300 | 0.0004 | 0.0784 | 0.2737 |
| **CIMD - Situational Vulnerability Q3** | 0.3672 | 0.0566 | 6.4900 | <.0001 | 0.2562 | 0.4782 |
| **CIMD -Situational Vulnerability Q4** | 0.5701 | 0.0551 | 10.3400 | <.0001 | 0.4620 | 0.6782 |
| **CIMD -Situational Vulnerability Q5 (most deprived)** | 0.6121 | 0.0610 | 10.0400 | <.0001 | 0.4926 | 0.7316 |
| **CIMD - Situational Vulnerability Q1 (least deprived) (REF)** | 0.0000 | . | . | . | . | . |
| **Social Assistance (any in past 5 years)** | 0.1551 | 0.0450 | 3.4500 | 0.0006 | 0.0670 | 0.2432 |
| **Social Assistance (none in past 5 years) (REF)** | 0.0000 | . | . | . | . | . |
| **Program of Study - French Immersion/Other** | -0.3102 | 0.0351 | -8.8400 | <.0001 | -0.3789 | -0.2414 |
| **Program of Study - French** | -0.7435 | 0.2146 | -3.4700 | 0.0005 | -1.1640 | -0.3230 |
| **Program of Study - English (REF)** | 0.0000 | . | . | . | . | . |
| **Household composition – Adults (age 22+) – No adults in household** | -0.1187 | 0.1515 | -0.7800 | 0.4332 | -0.4156 | 0.1782 |
| **Household composition – Adults (age 22+) – One adult in household** | -0.0918 | 0.0353 | -2.6000 | 0.0092 | -0.1609 | -0.0227 |
| **Household composition – Adults (age 22+) – More than one adult in household (REF)** | 0.0000 | . | . | . | . | . |
| **Household composition – Children (age 21 or under) – Student is only child in household** | 0.1906 | 0.0364 | 5.2400 | <.0001 | 0.1193 | 0.2618 |
| **Household composition – Children (age 21 or under) – Other children in household (REF)** | 0.0000 | . | . | . | . | . |
| **Recent immigrant** | -0.0595 | 0.0867 | -0.6900 | 0.4928 | -0.2295 | 0.1105 |
| **Not a recent immigrant (REF)** | 0.0000 | . | . | . | . | . |

**Supplementary Table S11d. GLM regression estimates – Absences per person due to out of school suspension – Grades K-12 (AY 2018-2020)**

| **Parameter** | **Estimate** | **Standard**  **Error** | **t Value** | **Pr > \|t\|** | **95% Confidence Limits** | |
| --- | --- | --- | --- | --- | --- | --- |
| **Intercept** | -24.7568 | 4.0114 | -6.1700 | <.0001 | -32.6201 | -16.8934 |
| **Treated ADHD** | 0.5549 | 0.8305 | 0.6700 | 0.5041 | -1.0731 | 2.1829 |
| **Untreated ADHD** | 1.3773 | 0.6519 | 2.1100 | 0.0346 | 0.0995 | 2.6552 |
| **No ADHD (REF)** | 0.0000 | . | . | . | . | . |
| **Age** | 2.0084 | 0.0998 | 20.1200 | <.0001 | 1.8127 | 2.2041 |
| **Observation Time** | 0.0106 | 0.0042 | 2.5400 | 0.0112 | 0.0024 | 0.0187 |
| **Male** | 1.8780 | 0.6274 | 2.9900 | 0.0028 | 0.6481 | 3.1079 |
| **Female (REF)** | 0.0000 | . | . | . | . | . |
| **Household income quintile Q2** | -0.4873 | 0.9159 | -0.5300 | 0.5947 | -2.2828 | 1.3081 |
| **Household income quintile Q3** | -0.7143 | 1.0589 | -0.6700 | 0.5000 | -2.7900 | 1.3614 |
| **Household income quintile Q4** | -0.8533 | 1.1390 | -0.7500 | 0.4538 | -3.0862 | 1.3795 |
| **Household income quintile Q5 (highest income)** | -0.4877 | 1.3121 | -0.3700 | 0.7101 | -3.0598 | 2.0844 |
| **Household income quintile Q1 (lowest income) (REF)** | 0.0000 | . | . | . | . | . |
| **NB Health Zone 2** | 0.4402 | 0.7392 | 0.6000 | 0.5516 | -1.0089 | 1.8892 |
| **NB Health Zone 3** | -0.7795 | 0.7717 | -1.0100 | 0.3125 | -2.2923 | 0.7333 |
| **NB Health Zone 4** | -1.6989 | 1.6609 | -1.0200 | 0.3064 | -4.9546 | 1.5568 |
| **NB Health Zone 5** | -1.5203 | 1.7477 | -0.8700 | 0.3844 | -4.9462 | 1.9055 |
| **NB Health Zone 6** | -0.6827 | 1.4716 | -0.4600 | 0.6427 | -3.5674 | 2.2019 |
| **NB Health Zone 7** | 3.1804 | 1.2708 | 2.5000 | 0.0123 | 0.6893 | 5.6716 |
| **NB Health Zone 1 (REF)** | 0.0000 | . | . | . | . | . |
| **Comorbid conditions - Mood & anxiety disorders (yes)** | 3.7521 | 1.4369 | 2.6100 | 0.0090 | 0.9355 | 6.5687 |
| **Comorbid conditions - Mood & anxiety disorders (no) (REF)** | 0.0000 | . | . | . | . | . |
| **Comorbid conditions – One or more of: asthma, diabetes, epilepsy, schizophrenia (yes)** | -4.5175 | 3.5660 | -1.2700 | 0.2053 | -11.5078 | 2.4728 |
| **Comorbid conditions – One or more of: asthma, diabetes, epilepsy, schizophrenia (no) (REF)** | 0.0000 | . | . | . | . | . |
| **Select medications (one or more)** | 2.5356 | 0.8956 | 2.8300 | 0.0047 | 0.7800 | 4.2913 |
| **Select medications (none) (REF)** | 0.0000 | . | . | . | . | . |
| **School District - Anglophone** | 6.7253 | 2.7660 | 2.4300 | 0.0151 | 1.3033 | 12.1473 |
| **School District – Francophone (REF)** | 0.0000 | . | . | . | . | . |
| **CIMD - Residential Instability Q2** | 1.3926 | 0.8290 | 1.6800 | 0.0930 | -0.2325 | 3.0177 |
| **CIMD - Residential Instability Q3** | -0.3686 | 0.9139 | -0.4000 | 0.6867 | -2.1601 | 1.4228 |
| **CIMD - Residential Instability Q4** | 0.3726 | 1.0138 | 0.3700 | 0.7132 | -1.6148 | 2.3600 |
| **CIMD – Residential Instability Q5 (most deprived)** | 0.4314 | 1.3062 | 0.3300 | 0.7412 | -2.1291 | 2.9918 |
| **CIMD - Residential Instability Q1 (least deprived) (REF)** | 0.0000 | . | . | . | . | . |
| **CIMD - Economic Dependency Q2** | -1.5622 | 1.1315 | -1.3800 | 0.1674 | -3.7802 | 0.6558 |
| **CIMD - Economic Dependency Q3** | -1.4785 | 1.0906 | -1.3600 | 0.1753 | -3.6164 | 0.6594 |
| **CIMD - Economic Dependency Q4** | -1.5382 | 1.1214 | -1.3700 | 0.1702 | -3.7364 | 0.6601 |
| **CIMD - Economic Dependency Q5 (most deprived)** | -3.5210 | 1.1443 | -3.0800 | 0.0021 | -5.7640 | -1.2779 |
| **CIMD - Economic Dependency Q1 (least deprived) (REF)** | 0.0000 | . | . | . | . | . |
| **CIMD - Ethnocultural Composition Q2** | -0.9706 | 0.6052 | -1.6000 | 0.1088 | -2.1570 | 0.2157 |
| **CIMD - Ethnocultural Composition Q3** | -1.7023 | 0.9496 | -1.7900 | 0.0731 | -3.5638 | 0.1591 |
| **CIMD - Ethnocultural Composition Q4** | -3.6349 | 1.4035 | -2.5900 | 0.0096 | -6.3862 | -0.8836 |
| **CIMD - Ethnocultural Composition Q5 (most deprived)** | -4.2511 | 1.8365 | -2.3100 | 0.0206 | -7.8510 | -0.6511 |
| **CIMD - Ethnocultural Composition Q1 (least deprived) (REF)** | 0.0000 | . | . | . | . | . |
| **CIMD -Situational Vulnerability Q2** | 1.3246 | 1.0757 | 1.2300 | 0.2182 | -0.7841 | 3.4333 |
| **CIMD - Situational Vulnerability Q3** | -0.2073 | 1.1983 | -0.1700 | 0.8627 | -2.5564 | 2.1418 |
| **CIMD -Situational Vulnerability Q4** | 1.1605 | 1.1403 | 1.0200 | 0.3088 | -1.0747 | 3.3958 |
| **CIMD -Situational Vulnerability Q5 (most deprived)** | 1.8084 | 1.1950 | 1.5100 | 0.1303 | -0.5342 | 4.1509 |
| **CIMD - Situational Vulnerability Q1 (least deprived) (REF)** | 0.0000 | . | . | . | . | . |
| **Social Assistance (any in past 5 years)** | 1.8451 | 0.6261 | 2.9500 | 0.0032 | 0.6178 | 3.0724 |
| **Social Assistance (none in past 5 years) (REF)** | 0.0000 | . | . | . | . | . |
| **Program of Study - French Immersion/Other** | -1.5861 | 0.7649 | -2.0700 | 0.0382 | -3.0855 | -0.0866 |
| **Program of Study - French** | 3.4489 | 2.8238 | 1.2200 | 0.2220 | -2.0865 | 8.9843 |
| **Program of Study - English (REF)** | 0.0000 | . | . | . | . | . |
| **Household composition – Adults (age 22+) – No adults in household** | 0.9895 | 2.1586 | 0.4600 | 0.6467 | -3.2420 | 5.2210 |
| **Household composition – Adults (age 22+) – One adult in household** | 0.2192 | 0.5798 | 0.3800 | 0.7054 | -0.9173 | 1.3557 |
| **Household composition – Adults (age 22+) – More than one adult in household (REF)** | 0.0000 | . | . | . | . | . |
| **Household composition – Children (age 21 or under) – Student is only child in household** | 0.1981 | 0.6497 | 0.3000 | 0.7605 | -1.0756 | 1.4717 |
| **Household composition – Children (age 21 or under) – Other children in household (REF)** | 0.0000 | . | . | . | . | . |
| **Recent immigrant** | -2.0843 | 2.1079 | -0.9900 | 0.3228 | -6.2165 | 2.0478 |
| **Not a recent immigrant (REF)** | 0.0000 | . | . | . | . | . |

**Supplementary Table S11e. GLM regression estimates – Absences per person due to unknown reasons – Grades K-12 (AY 2018-2020)**

| **Parameter** | **Estimate** | **Standard**  **Error** | **t Value** | **Pr > \|t\|** | **95% Confidence Limits** | |
| --- | --- | --- | --- | --- | --- | --- |
| **Intercept** | -57.4409 | 1.4220 | -40.3900 | <.0001 | -60.2280 | -54.6539 |
| **Treated ADHD** | -4.0355 | 0.4180 | -9.6500 | <.0001 | -4.8548 | -3.2162 |
| **Untreated ADHD** | 7.0688 | 0.3008 | 23.5000 | <.0001 | 6.4793 | 7.6584 |
| **No ADHD (REF)** | 0.0000 | . | . | . | . | . |
| **Age** | 4.4208 | 0.0233 | 189.5200 | <.0001 | 4.3751 | 4.4665 |
| **Observation Time** | 0.0859 | 0.0015 | 56.5100 | <.0001 | 0.0829 | 0.0889 |
| **Male** | -0.0904 | 0.1842 | -0.4900 | 0.6237 | -0.4515 | 0.2707 |
| **Female (REF)** | 0.0000 | . | . | . | . | . |
| **Household income quintile Q2** | -2.9014 | 0.3483 | -8.3300 | <.0001 | -3.5841 | -2.2188 |
| **Household income quintile Q3** | -2.7626 | 0.3806 | -7.2600 | <.0001 | -3.5086 | -2.0166 |
| **Household income quintile Q4** | -3.8319 | 0.4080 | -9.3900 | <.0001 | -4.6315 | -3.0323 |
| **Household income quintile Q5 (highest income)** | -3.7836 | 0.4411 | -8.5800 | <.0001 | -4.6481 | -2.9191 |
| **Household income quintile Q1 (lowest income) (REF)** | 0.0000 | . | . | . | . | . |
| **NB Health Zone 2** | 11.5381 | 0.2725 | 42.3500 | <.0001 | 11.0041 | 12.0722 |
| **NB Health Zone 3** | 3.9201 | 0.2667 | 14.7000 | <.0001 | 3.3974 | 4.4427 |
| **NB Health Zone 4** | 0.8734 | 0.4495 | 1.9400 | 0.0520 | -0.0076 | 1.7544 |
| **NB Health Zone 5** | 1.0394 | 0.5831 | 1.7800 | 0.0746 | -0.1034 | 2.1822 |
| **NB Health Zone 6** | -3.4970 | 0.4220 | -8.2900 | <.0001 | -4.3241 | -2.6698 |
| **NB Health Zone 7** | 3.4848 | 0.4411 | 7.9000 | <.0001 | 2.6202 | 4.3493 |
| **NB Health Zone 1 (REF)** | 0.0000 | . | . | . | . | . |
| **Comorbid conditions - Mood & anxiety disorders (yes)** | 22.2881 | 0.8394 | 26.5500 | <.0001 | 20.6429 | 23.9333 |
| **Comorbid conditions - Mood & anxiety disorders (no) (REF)** | 0.0000 | . | . | . | . | . |
| **Comorbid conditions – One or more of: asthma, diabetes, epilepsy, schizophrenia (yes)** | 1.2357 | 1.2109 | 1.0200 | 0.3075 | -1.1376 | 3.6090 |
| **Comorbid conditions – One or more of: asthma, diabetes, epilepsy, schizophrenia (no) (REF)** | 0.0000 | . | . | . | . | . |
| **Select medications (one or more)** | 19.1571 | 0.5815 | 32.9400 | <.0001 | 18.0173 | 20.2969 |
| **Select medications (none) (REF)** | 0.0000 | . | . | . | . | . |
| **School District - Anglophone** | 5.8657 | 1.1669 | 5.0300 | <.0001 | 3.5786 | 8.1528 |
| **School District – Francophone (REF)** | 0.0000 | . | . | . | . | . |
| **CIMD - Residential Instability Q2** | 0.5082 | 0.2725 | 1.8600 | 0.0622 | -0.0260 | 1.0423 |
| **CIMD - Residential Instability Q3** | 1.5740 | 0.2920 | 5.3900 | <.0001 | 1.0018 | 2.1462 |
| **CIMD - Residential Instability Q4** | 3.3872 | 0.3395 | 9.9800 | <.0001 | 2.7217 | 4.0527 |
| **CIMD – Residential Instability Q5 (most deprived)** | 3.0290 | 0.4528 | 6.6900 | <.0001 | 2.1415 | 3.9165 |
| **CIMD - Residential Instability Q1 (least deprived) (REF)** | 0.0000 | . | . | . | . | . |
| **CIMD - Economic Dependency Q2** | -0.8575 | 0.3422 | -2.5100 | 0.0122 | -1.5283 | -0.1867 |
| **CIMD - Economic Dependency Q3** | -1.0575 | 0.3445 | -3.0700 | 0.0021 | -1.7327 | -0.3822 |
| **CIMD - Economic Dependency Q4** | -1.0042 | 0.3552 | -2.8300 | 0.0047 | -1.7005 | -0.3080 |
| **CIMD - Economic Dependency Q5 (most deprived)** | -2.6663 | 0.3648 | -7.3100 | <.0001 | -3.3813 | -1.9513 |
| **CIMD - Economic Dependency Q1 (least deprived) (REF)** | 0.0000 | . | . | . | . | . |
| **CIMD - Ethnocultural Composition Q2** | -0.0189 | 0.2122 | -0.0900 | 0.9289 | -0.4348 | 0.3969 |
| **CIMD - Ethnocultural Composition Q3** | -1.1893 | 0.3052 | -3.9000 | <.0001 | -1.7875 | -0.5912 |
| **CIMD - Ethnocultural Composition Q4** | -2.1844 | 0.4501 | -4.8500 | <.0001 | -3.0665 | -1.3022 |
| **CIMD - Ethnocultural Composition Q5 (most deprived)** | -2.7489 | 0.6553 | -4.1900 | <.0001 | -4.0333 | -1.4646 |
| **CIMD - Ethnocultural Composition Q1 (least deprived) (REF)** | 0.0000 | . | . | . | . | . |
| **CIMD -Situational Vulnerability Q2** | 1.5539 | 0.3302 | 4.7100 | <.0001 | 0.9068 | 2.2010 |
| **CIMD - Situational Vulnerability Q3** | 1.5570 | 0.3671 | 4.2400 | <.0001 | 0.8374 | 2.2766 |
| **CIMD -Situational Vulnerability Q4** | 2.4385 | 0.3598 | 6.7800 | <.0001 | 1.7333 | 3.1438 |
| **CIMD -Situational Vulnerability Q5 (most deprived)** | 5.7942 | 0.3936 | 14.7200 | <.0001 | 5.0228 | 6.5656 |
| **CIMD - Situational Vulnerability Q1 (least deprived) (REF)** | 0.0000 | . | . | . | . | . |
| **Social Assistance (any in past 5 years)** | 12.3750 | 0.2788 | 44.3800 | <.0001 | 11.8285 | 12.9215 |
| **Social Assistance (none in past 5 years) (REF)** | 0.0000 | . | . | . | . | . |
| **Program of Study - French Immersion/Other** | -12.6284 | 0.2346 | -53.8300 | <.0001 | -13.0882 | -12.1685 |
| **Program of Study - French** | -5.6738 | 1.1788 | -4.8100 | <.0001 | -7.9843 | -3.3633 |
| **Program of Study - English (REF)** | 0.0000 | . | . | . | . | . |
| **Household composition – Adults (age 22+) – No adults in household** | 3.1183 | 0.8813 | 3.5400 | 0.0004 | 1.3910 | 4.8457 |
| **Household composition – Adults (age 22+) – One adult in household** | 3.3367 | 0.2227 | 14.9800 | <.0001 | 2.9002 | 3.7732 |
| **Household composition – Adults (age 22+) – More than one adult in household (REF)** | 0.0000 | . | . | . | . | . |
| **Household composition – Children (age 21 or under) – Student is only child in household** | -0.0143 | 0.2346 | -0.0600 | 0.9513 | -0.4741 | 0.4454 |
| **Household composition – Children (age 21 or under) – Other children in household (REF)** | 0.0000 | . | . | . | . | . |
| **Recent immigrant** | -6.6946 | 0.5184 | -12.9100 | <.0001 | -7.7107 | -5.6785 |
| **Not a recent immigrant (REF)** | 0.0000 | . | . | . | . | . |
