# Supplementary material for: Academic Outcomes in Primary and Secondary School Students Prescribed Long-Acting Stimulants for ADHD Management
Source: J Atten Disord. 2025 Oct 7;30(4):493–505. doi: 10.1177/10870547251378169 (PMC12953683; doi:10.1177/10870547251378169)
Supplement: sj-docx-12-jad-10.1177_10870547251378169 – Supplemental material for Academic Outcomes in Primary and Secondary School Students Prescribed Long-Acting Stimulants for ADHD Management [file sj-docx-12-jad-10.1177_10870547251378169.docx]

**Supplementary Table S12a. GLM regression estimates – Absences per person (any absence) – Grades K-12 (AY 2018-2020) (Untreated group as reference)**

| **Parameter** | **Estimate** | **Standard**  **Error** | **t Value** | **Pr > \|t\|** | **95% Confidence Limits** | |
| --- | --- | --- | --- | --- | --- | --- |
| **Intercept** | -63.0368 | 2.8763 | -21.9200 | <.0001 | -68.6743 | -57.3993 |
| **Treated ADHD** | -10.1155 | 0.5611 | -18.0300 | <.0001 | -11.2153 | -9.0158 |
| **Untreated ADHD (REF)** | 0.0000 | . | . | . | . | . |
| **Age** | 6.0980 | 0.0720 | 84.7500 | <.0001 | 5.9570 | 6.2390 |
| **Observation Time** | 0.0826 | 0.0031 | 26.3200 | <.0001 | 0.0764 | 0.0887 |
| **Male** | -1.7294 | 0.5557 | -3.1100 | 0.0019 | -2.8186 | -0.6402 |
| **Female (REF)** | 0.0000 | . | . | . | . | . |
| **Household income quintile Q2** | -2.2806 | 0.9440 | -2.4200 | 0.0157 | -4.1308 | -0.4304 |
| **Household income quintile Q3** | -1.3838 | 1.0501 | -1.3200 | 0.1876 | -3.4420 | 0.6744 |
| **Household income quintile Q4** | -3.1149 | 1.1243 | -2.7700 | 0.0056 | -5.3184 | -0.9113 |
| **Household income quintile Q5 (highest income)** | -2.5271 | 1.2310 | -2.0500 | 0.0401 | -4.9398 | -0.1144 |
| **Household income quintile Q1 (lowest income) (REF)** | 0.0000 | . | . | . | . | . |
| **NB Health Zone 2** | 18.7056 | 0.7905 | 23.6600 | <.0001 | 17.1562 | 20.2551 |
| **NB Health Zone 3** | 3.0044 | 0.7676 | 3.9100 | <.0001 | 1.4999 | 4.5089 |
| **NB Health Zone 4** | -2.7452 | 1.2881 | -2.1300 | 0.0331 | -5.2698 | -0.2205 |
| **NB Health Zone 5** | -1.6822 | 1.6016 | -1.0500 | 0.2936 | -4.8214 | 1.4570 |
| **NB Health Zone 6** | -3.4995 | 1.0663 | -3.2800 | 0.0010 | -5.5895 | -1.4096 |
| **NB Health Zone 7** | -1.1189 | 1.3466 | -0.8300 | 0.4060 | -3.7582 | 1.5205 |
| **NB Health Zone 1 (REF)** | 0.0000 | . | . | . | . | . |
| **Comorbid conditions - Mood & anxiety disorders (yes)** | 23.0166 | 1.8727 | 12.2900 | <.0001 | 19.3460 | 26.6871 |
| **Comorbid conditions - Mood & anxiety disorders (no) (REF)** | 0.0000 | . | . | . | . | . |
| **Comorbid conditions – One or more of: asthma, diabetes, epilepsy, schizophrenia (yes)** | -0.6707 | 3.1798 | -0.2100 | 0.8329 | -6.9031 | 5.5618 |
| **Comorbid conditions – One or more of: asthma, diabetes, epilepsy, schizophrenia (no) (REF)** | 0.0000 | . | . | . | . | . |
| **Select medications (one or more)** | 11.0309 | 0.8045 | 13.7100 | <.0001 | 9.4542 | 12.6077 |
| **Select medications (none) (REF)** | 0.0000 | . | . | . | . | . |
| **School District - Anglophone** | 4.7953 | 1.9005 | 2.5200 | 0.0116 | 1.0703 | 8.5203 |
| **School District – Francophone (REF)** | 0.0000 | . | . | . | . | . |
| **CIMD - Residential Instability Q2** | 1.4288 | 0.8094 | 1.7700 | 0.0775 | -0.1576 | 3.0151 |
| **CIMD - Residential Instability Q3** | 3.3348 | 0.8454 | 3.9400 | <.0001 | 1.6778 | 4.9917 |
| **CIMD - Residential Instability Q4** | 3.0155 | 0.9624 | 3.1300 | 0.0017 | 1.1291 | 4.9018 |
| **CIMD – Residential Instability Q5 (most deprived)** | 4.1941 | 1.2404 | 3.3800 | 0.0007 | 1.7628 | 6.6254 |
| **CIMD - Residential Instability Q1 (least deprived) (REF)** | 0.0000 | . | . | . | . | . |
| **CIMD - Economic Dependency Q2** | 0.1514 | 0.9919 | 0.1500 | 0.8787 | -1.7927 | 2.0954 |
| **CIMD - Economic Dependency Q3** | 0.8825 | 0.9978 | 0.8800 | 0.3765 | -1.0733 | 2.8383 |
| **CIMD - Economic Dependency Q4** | -0.3292 | 1.0223 | -0.3200 | 0.7474 | -2.3330 | 1.6746 |
| **CIMD - Economic Dependency Q5 (most deprived)** | -0.3619 | 1.0543 | -0.3400 | 0.7314 | -2.4284 | 1.7046 |
| **CIMD - Economic Dependency Q1 (least deprived) (REF)** | 0.0000 | . | . | . | . | . |
| **CIMD - Ethnocultural Composition Q2** | -0.7912 | 0.5987 | -1.3200 | 0.1863 | -1.9647 | 0.3823 |
| **CIMD - Ethnocultural Composition Q3** | -2.5539 | 0.8645 | -2.9500 | 0.0031 | -4.2482 | -0.8595 |
| **CIMD - Ethnocultural Composition Q4** | -3.6147 | 1.2706 | -2.8400 | 0.0044 | -6.1051 | -1.1244 |
| **CIMD - Ethnocultural Composition Q5 (most deprived)** | -3.3718 | 1.8255 | -1.8500 | 0.0647 | -6.9498 | 0.2061 |
| **CIMD - Ethnocultural Composition Q1 (least deprived) (REF)** | 0.0000 | . | . | . | . | . |
| **CIMD -Situational Vulnerability Q2** | 1.5119 | 0.9698 | 1.5600 | 0.1190 | -0.3889 | 3.4128 |
| **CIMD - Situational Vulnerability Q3** | -0.1300 | 1.0743 | -0.1200 | 0.9037 | -2.2355 | 1.9756 |
| **CIMD -Situational Vulnerability Q4** | 3.0220 | 1.0531 | 2.8700 | 0.0041 | 0.9578 | 5.0861 |
| **CIMD -Situational Vulnerability Q5 (most deprived)** | 4.1398 | 1.1280 | 3.6700 | 0.0002 | 1.9289 | 6.3507 |
| **CIMD - Situational Vulnerability Q1 (least deprived) (REF)** | 0.0000 | . | . | . | . | . |
| **Social Assistance (any in past 5 years)** | 10.5038 | 0.6888 | 15.2500 | <.0001 | 9.1538 | 11.8539 |
| **Social Assistance (none in past 5 years) (REF)** | 0.0000 | . | . | . | . | . |
| **Program of Study - French Immersion/Other** | -12.1573 | 0.7633 | -15.9300 | <.0001 | -13.6535 | -10.6612 |
| **Program of Study - French** | -10.6643 | 1.9439 | -5.4900 | <.0001 | -14.4743 | -6.8543 |
| **Program of Study - English (REF)** | 0.0000 | . | . | . | . | . |
| **Household composition – Adults (age 22+) – No adults in household** | -20.2551 | 1.8854 | -10.7400 | <.0001 | -23.9506 | -16.5597 |
| **Household composition – Adults (age 22+) – One adult in household** | 2.6891 | 0.5845 | 4.6000 | <.0001 | 1.5434 | 3.8347 |
| **Household composition – Adults (age 22+) – More than one adult in household (REF)** | 0.0000 | . | . | . | . | . |
| **Household composition – Children (age 21 or under) – Student is only child in household** | -0.1616 | 0.6168 | -0.2600 | 0.7934 | -1.3705 | 1.0474 |
| **Household composition – Children (age 21 or under) – Other children in household (REF)** | 0.0000 | . | . | . | . | . |
| **Recent immigrant** | -6.1496 | 3.4279 | -1.7900 | 0.0728 | -12.8684 | 0.5691 |
| **Not a recent immigrant (REF)** | 0.0000 | . | . | . | . | . |

**Supplementary Table S12b. GLM regression estimates – Absences per person due to illness – Grades K-12 (AY 2018-2020) (Untreated group as reference)**

| **Parameter** | **Estimate** | **Standard**  **Error** | **t Value** | **Pr > \|t\|** | **95% Confidence Limits** | |
| --- | --- | --- | --- | --- | --- | --- |
| **Intercept** | -7.1807 | 1.1622 | -6.1800 | <.0001 | -9.4587 | -4.9028 |
| **Treated ADHD** | -1.8332 | 0.1979 | -9.2600 | <.0001 | -2.2211 | -1.4453 |
| **Untreated ADHD (REF)** | 0.0000 | . | . | . | . | . |
| **Age** | 1.1006 | 0.0260 | 42.3200 | <.0001 | 1.0496 | 1.1516 |
| **Observation Time** | 0.0084 | 0.0012 | 6.7500 | <.0001 | 0.0060 | 0.0109 |
| **Male** | -0.6188 | 0.1929 | -3.2100 | 0.0013 | -0.9969 | -0.2406 |
| **Female (REF)** | 0.0000 | . | . | . | . | . |
| **Household income quintile Q2** | -0.0743 | 0.3260 | -0.2300 | 0.8197 | -0.7134 | 0.5647 |
| **Household income quintile Q3** | -0.3275 | 0.3659 | -0.9000 | 0.3708 | -1.0448 | 0.3897 |
| **Household income quintile Q4** | -0.6162 | 0.3931 | -1.5700 | 0.1170 | -1.3868 | 0.1543 |
| **Household income quintile Q5 (highest income)** | -0.6193 | 0.4368 | -1.4200 | 0.1562 | -1.4754 | 0.2368 |
| **Household income quintile Q1 (lowest income) (REF)** | 0.0000 | . | . | . | . | . |
| **NB Health Zone 2** | 1.7502 | 0.2656 | 6.5900 | <.0001 | 1.2296 | 2.2707 |
| **NB Health Zone 3** | -0.6384 | 0.2629 | -2.4300 | 0.0152 | -1.1537 | -0.1230 |
| **NB Health Zone 4** | -0.4965 | 0.4981 | -1.0000 | 0.3189 | -1.4729 | 0.4799 |
| **NB Health Zone 5** | -1.4236 | 0.5784 | -2.4600 | 0.0139 | -2.5574 | -0.2898 |
| **NB Health Zone 6** | -0.6178 | 0.3933 | -1.5700 | 0.1162 | -1.3888 | 0.1531 |
| **NB Health Zone 7** | -3.4153 | 0.5016 | -6.8100 | <.0001 | -4.3984 | -2.4323 |
| **NB Health Zone 1 (REF)** | 0.0000 | . | . | . | . | . |
| **Comorbid conditions - Mood & anxiety disorders (yes)** | 4.5821 | 0.5960 | 7.6900 | <.0001 | 3.4140 | 5.7502 |
| **Comorbid conditions - Mood & anxiety disorders (no) (REF)** | 0.0000 | . | . | . | . | . |
| **Comorbid conditions – One or more of: asthma, diabetes, epilepsy, schizophrenia (yes)** | 0.9096 | 0.9989 | 0.9100 | 0.3625 | -1.0483 | 2.8675 |
| **Comorbid conditions – One or more of: asthma, diabetes, epilepsy, schizophrenia (no) (REF)** | 0.0000 | . | . | . | . | . |
| **Select medications (one or more)** | 1.5101 | 0.2789 | 5.4100 | <.0001 | 0.9634 | 2.0568 |
| **Select medications (none) (REF)** | 0.0000 | . | . | . | . | . |
| **School District - Anglophone** | 1.4569 | 0.8493 | 1.7200 | 0.0863 | -0.2078 | 3.1215 |
| **School District – Francophone (REF)** | 0.0000 | . | . | . | . | . |
| **CIMD - Residential Instability Q2** | 0.0113 | 0.2844 | 0.0400 | 0.9683 | -0.5461 | 0.5687 |
| **CIMD - Residential Instability Q3** | 0.4136 | 0.2970 | 1.3900 | 0.1637 | -0.1685 | 0.9956 |
| **CIMD - Residential Instability Q4** | 0.0439 | 0.3357 | 0.1300 | 0.8959 | -0.6140 | 0.7018 |
| **CIMD – Residential Instability Q5 (most deprived)** | -0.1538 | 0.4322 | -0.3600 | 0.7220 | -1.0008 | 0.6933 |
| **CIMD - Residential Instability Q1 (least deprived) (REF)** | 0.0000 | . | . | . | . | . |
| **CIMD - Economic Dependency Q2** | 0.3872 | 0.3477 | 1.1100 | 0.2655 | -0.2944 | 1.0687 |
| **CIMD - Economic Dependency Q3** | 0.3386 | 0.3475 | 0.9700 | 0.3299 | -0.3425 | 1.0198 |
| **CIMD - Economic Dependency Q4** | 0.0735 | 0.3554 | 0.2100 | 0.8362 | -0.6232 | 0.7702 |
| **CIMD - Economic Dependency Q5 (most deprived)** | 0.5476 | 0.3682 | 1.4900 | 0.1370 | -0.1741 | 1.2693 |
| **CIMD - Economic Dependency Q1 (least deprived) (REF)** | 0.0000 | . | . | . | . | . |
| **CIMD - Ethnocultural Composition Q2** | 0.1877 | 0.2099 | 0.8900 | 0.3712 | -0.2237 | 0.5991 |
| **CIMD - Ethnocultural Composition Q3** | -0.7086 | 0.2997 | -2.3600 | 0.0180 | -1.2960 | -0.1213 |
| **CIMD - Ethnocultural Composition Q4** | -0.0298 | 0.4291 | -0.0700 | 0.9446 | -0.8709 | 0.8112 |
| **CIMD - Ethnocultural Composition Q5 (most deprived)** | -0.7042 | 0.6249 | -1.1300 | 0.2598 | -1.9290 | 0.5206 |
| **CIMD - Ethnocultural Composition Q1 (least deprived) (REF)** | 0.0000 | . | . | . | . | . |
| **CIMD -Situational Vulnerability Q2** | 0.2302 | 0.3331 | 0.6900 | 0.4895 | -0.4226 | 0.8830 |
| **CIMD - Situational Vulnerability Q3** | 0.0420 | 0.3744 | 0.1100 | 0.9108 | -0.6919 | 0.7758 |
| **CIMD -Situational Vulnerability Q4** | 0.0614 | 0.3652 | 0.1700 | 0.8664 | -0.6544 | 0.7773 |
| **CIMD -Situational Vulnerability Q5 (most deprived)** | -0.1126 | 0.3944 | -0.2900 | 0.7752 | -0.8856 | 0.6604 |
| **CIMD - Situational Vulnerability Q1 (least deprived) (REF)** | 0.0000 | . | . | . | . | . |
| **Social Assistance (any in past 5 years)** | 0.8160 | 0.2334 | 3.5000 | 0.0005 | 0.3585 | 1.2734 |
| **Social Assistance (none in past 5 years) (REF)** | 0.0000 | . | . | . | . | . |
| **Program of Study - French Immersion/Other** | -0.4871 | 0.2515 | -1.9400 | 0.0528 | -0.9800 | 0.0058 |
| **Program of Study - French** | -0.0429 | 0.8566 | -0.0500 | 0.9601 | -1.7218 | 1.6360 |
| **Program of Study - English (REF)** | 0.0000 | . | . | . | . | . |
| **Household composition – Adults (age 22+) – No adults in household** | -2.7208 | 0.7762 | -3.5100 | 0.0005 | -4.2421 | -1.1995 |
| **Household composition – Adults (age 22+) – One adult in household** | -0.3702 | 0.2032 | -1.8200 | 0.0685 | -0.7685 | 0.0281 |
| **Household composition – Adults (age 22+) – More than one adult in household (REF)** | 0.0000 | . | . | . | . | . |
| **Household composition – Children (age 21 or under) – Student is only child in household** | 1.4543 | 0.2146 | 6.7800 | <.0001 | 1.0336 | 1.8749 |
| **Household composition – Children (age 21 or under) – Other children in household (REF)** | 0.0000 | . | . | . | . | . |
| **Recent immigrant** | 0.3119 | 1.1943 | 0.2600 | 0.7940 | -2.0290 | 2.6529 |
| **Not a recent immigrant (REF)** | 0.0000 | . | . | . | . | . |

**Supplementary Table S12c. GLM regression estimates – Absences per person due to medical appointment – Grades K-12 (AY 2018-2020) (Untreated group as reference)**

| **Parameter** | **Estimate** | **Standard**  **Error** | **t Value** | **Pr > \|t\|** | **95% Confidence Limits** | |
| --- | --- | --- | --- | --- | --- | --- |
| **Intercept** | -1.3898 | 0.4275 | -3.2500 | 0.0012 | -2.2277 | -0.5519 |
| **Treated ADHD** | -0.1802 | 0.0707 | -2.5500 | 0.0911 | -0.3188 | 0.0416 |
| **Untreated ADHD (REF)** | 0.0000 | . | . | . | . | . |
| **Age** | 0.3007 | 0.0096 | 31.2200 | <.0001 | 0.2818 | 0.3196 |
| **Observation Time** | 0.0042 | 0.0005 | 9.1900 | <.0001 | 0.0033 | 0.0051 |
| **Male** | -0.2718 | 0.0706 | -3.8500 | 0.0001 | -0.4102 | -0.1333 |
| **Female (REF)** | 0.0000 | . | . | . | . | . |
| **Household income quintile Q2** | -0.2213 | 0.1231 | -1.8000 | 0.0724 | -0.4626 | 0.0201 |
| **Household income quintile Q3** | 0.0287 | 0.1371 | 0.2100 | 0.8342 | -0.2400 | 0.2974 |
| **Household income quintile Q4** | 0.0286 | 0.1457 | 0.2000 | 0.8442 | -0.2569 | 0.3142 |
| **Household income quintile Q5 (highest income)** | -0.0171 | 0.1615 | -0.1100 | 0.9158 | -0.3337 | 0.2996 |
| **Household income quintile Q1 (lowest income) (REF)** | 0.0000 | . | . | . | . | . |
| **NB Health Zone 2** | 0.3856 | 0.0989 | 3.9000 | <.0001 | 0.1918 | 0.5794 |
| **NB Health Zone 3** | -0.0824 | 0.0955 | -0.8600 | 0.3881 | -0.2696 | 0.1048 |
| **NB Health Zone 4** | -0.2447 | 0.1850 | -1.3200 | 0.1859 | -0.6074 | 0.1179 |
| **NB Health Zone 5** | -0.5669 | 0.2276 | -2.4900 | 0.0127 | -1.0130 | -0.1209 |
| **NB Health Zone 6** | -0.0185 | 0.1424 | -0.1300 | 0.8965 | -0.2976 | 0.2605 |
| **NB Health Zone 7** | -1.1036 | 0.1940 | -5.6900 | <.0001 | -1.4839 | -0.7233 |
| **NB Health Zone 1 (REF)** | 0.0000 | . | . | . | . | . |
| **Comorbid conditions - Mood & anxiety disorders (yes)** | 1.1309 | 0.2050 | 5.5200 | <.0001 | 0.7291 | 1.5328 |
| **Comorbid conditions - Mood & anxiety disorders (no) (REF)** | 0.0000 | . | . | . | . | . |
| **Comorbid conditions – One or more of: asthma, diabetes, epilepsy, schizophrenia (yes)** | 1.6598 | 0.3474 | 4.7800 | <.0001 | 0.9789 | 2.3408 |
| **Comorbid conditions – One or more of: asthma, diabetes, epilepsy, schizophrenia (no) (REF)** | 0.0000 | . | . | . | . | . |
| **Select medications (one or more)** | 0.5361 | 0.0995 | 5.3900 | <.0001 | 0.3411 | 0.7311 |
| **Select medications (none) (REF)** | 0.0000 | . | . | . | . | . |
| **School District - Anglophone** | -0.1401 | 0.3126 | -0.4500 | 0.6540 | -0.7527 | 0.4725 |
| **School District – Francophone (REF)** | 0.0000 | . | . | . | . | . |
| **CIMD - Residential Instability Q2** | 0.1148 | 0.1029 | 1.1200 | 0.2647 | -0.0869 | 0.3165 |
| **CIMD - Residential Instability Q3** | 0.1642 | 0.1082 | 1.5200 | 0.1290 | -0.0478 | 0.3762 |
| **CIMD - Residential Instability Q4** | 0.0072 | 0.1237 | 0.0600 | 0.9533 | -0.2351 | 0.2496 |
| **CIMD – Residential Instability Q5 (most deprived)** | -0.2448 | 0.1595 | -1.5300 | 0.1250 | -0.5575 | 0.0679 |
| **CIMD - Residential Instability Q1 (least deprived) (REF)** | 0.0000 | . | . | . | . | . |
| **CIMD - Economic Dependency Q2** | 0.2494 | 0.1250 | 2.0000 | 0.0460 | 0.0044 | 0.4943 |
| **CIMD - Economic Dependency Q3** | 0.2964 | 0.1259 | 2.3500 | 0.0186 | 0.0496 | 0.5433 |
| **CIMD - Economic Dependency Q4** | 0.3897 | 0.1289 | 3.0200 | 0.0025 | 0.1371 | 0.6424 |
| **CIMD - Economic Dependency Q5 (most deprived)** | 0.5855 | 0.1336 | 4.3800 | <.0001 | 0.3236 | 0.8474 |
| **CIMD - Economic Dependency Q1 (least deprived) (REF)** | 0.0000 | . | . | . | . | . |
| **CIMD - Ethnocultural Composition Q2** | 0.0679 | 0.0776 | 0.8800 | 0.3815 | -0.0842 | 0.2200 |
| **CIMD - Ethnocultural Composition Q3** | 0.0072 | 0.1088 | 0.0700 | 0.9475 | -0.2060 | 0.2204 |
| **CIMD - Ethnocultural Composition Q4** | -0.2237 | 0.1572 | -1.4200 | 0.1546 | -0.5318 | 0.0843 |
| **CIMD - Ethnocultural Composition Q5 (most deprived)** | -0.3752 | 0.2363 | -1.5900 | 0.1124 | -0.8384 | 0.0880 |
| **CIMD - Ethnocultural Composition Q1 (least deprived) (REF)** | 0.0000 | . | . | . | . | . |
| **CIMD -Situational Vulnerability Q2** | -0.0324 | 0.1209 | -0.2700 | 0.7887 | -0.2695 | 0.2046 |
| **CIMD - Situational Vulnerability Q3** | 0.0184 | 0.1360 | 0.1400 | 0.8926 | -0.2483 | 0.2850 |
| **CIMD -Situational Vulnerability Q4** | 0.2028 | 0.1331 | 1.5200 | 0.1275 | -0.0580 | 0.4637 |
| **CIMD -Situational Vulnerability Q5 (most deprived)** | 0.1898 | 0.1442 | 1.3200 | 0.1883 | -0.0929 | 0.4724 |
| **CIMD - Situational Vulnerability Q1 (least deprived) (REF)** | 0.0000 | . | . | . | . | . |
| **Social Assistance (any in past 5 years)** | 0.2446 | 0.0895 | 2.7300 | 0.0063 | 0.0692 | 0.4201 |
| **Social Assistance (none in past 5 years) (REF)** | 0.0000 | . | . | . | . | . |
| **Program of Study - French Immersion/Other** | -0.2215 | 0.0921 | -2.4100 | 0.0162 | -0.4020 | -0.0410 |
| **Program of Study - French** | -0.5123 | 0.3162 | -1.6200 | 0.1052 | -1.1321 | 0.1075 |
| **Program of Study - English (REF)** | 0.0000 | . | . | . | . | . |
| **Household composition – Adults (age 22+) – No adults in household** | -0.1664 | 0.2847 | -0.5800 | 0.5590 | -0.7244 | 0.3917 |
| **Household composition – Adults (age 22+) – One adult in household** | -0.2543 | 0.0762 | -3.3400 | 0.0009 | -0.4037 | -0.1049 |
| **Household composition – Adults (age 22+) – More than one adult in household (REF)** | 0.0000 | . | . | . | . | . |
| **Household composition – Children (age 21 or under) – Student is only child in household** | 0.1672 | 0.0795 | 2.1000 | 0.0355 | 0.0113 | 0.3231 |
| **Household composition – Children (age 21 or under) – Other children in household (REF)** | 0.0000 | . | . | . | . | . |
| **Recent immigrant** | -0.0190 | 0.4127 | -0.0500 | 0.9633 | -0.8278 | 0.7899 |
| **Not a recent immigrant (REF)** | 0.0000 | . | . | . | . | . |

**Supplementary Table S12d. GLM regression estimates – Absences per person due to out of school suspension – Grades K-12 (AY 2018-2020) (Untreated group as reference)**

| **Parameter** | **Estimate** | **Standard**  **Error** | **t Value** | **Pr > \|t\|** | **95% Confidence Limits** | |
| --- | --- | --- | --- | --- | --- | --- |
| **Intercept** | -34.4944 | 6.2409 | -5.5300 | <.0001 | -46.7311 | -22.2577 |
| **Treated ADHD** | -0.5286 | 0.9504 | -0.5600 | 0.5781 | -2.3920 | 1.3349 |
| **Untreated ADHD (REF)** | 0.0000 | . | . | . | . | . |
| **Age** | 2.6882 | 0.1824 | 14.7400 | <.0001 | 2.3307 | 3.0458 |
| **Observation Time** | 0.0176 | 0.0062 | 2.8500 | 0.0045 | 0.0055 | 0.0297 |
| **Male** | 2.4412 | 1.2219 | 2.0000 | 0.0458 | 0.0454 | 4.8369 |
| **Female (REF)** | 0.0000 | . | . | . | . | . |
| **Household income quintile Q2** | -2.9353 | 1.5201 | -1.9300 | 0.0536 | -5.9158 | 0.0451 |
| **Household income quintile Q3** | -3.0406 | 1.7800 | -1.7100 | 0.0877 | -6.5308 | 0.4496 |
| **Household income quintile Q4** | -4.9512 | 1.8929 | -2.6200 | 0.0089 | -8.6627 | -1.2397 |
| **Household income quintile Q5 (highest income)** | -4.2597 | 2.1365 | -1.9900 | 0.0463 | -8.4487 | -0.0707 |
| **Household income quintile Q1 (lowest income) (REF)** | 0.0000 | . | . | . | . | . |
| **NB Health Zone 2** | 1.9106 | 1.2647 | 1.5100 | 0.1310 | -0.5692 | 4.3904 |
| **NB Health Zone 3** | -0.4196 | 1.3269 | -0.3200 | 0.7518 | -3.0212 | 2.1820 |
| **NB Health Zone 4** | -0.5006 | 2.7635 | -0.1800 | 0.8562 | -5.9190 | 4.9177 |
| **NB Health Zone 5** | -0.6270 | 2.8048 | -0.2200 | 0.8231 | -6.1265 | 4.8725 |
| **NB Health Zone 6** | 0.6087 | 2.3193 | 0.2600 | 0.7930 | -3.9387 | 5.1561 |
| **NB Health Zone 7** | 3.4755 | 2.1430 | 1.6200 | 0.1050 | -0.7264 | 7.6774 |
| **NB Health Zone 1 (REF)** | 0.0000 | . | . | . | . | . |
| **Comorbid conditions - Mood & anxiety disorders (yes)** | 5.2023 | 2.2281 | 2.3300 | 0.0196 | 0.8336 | 9.5709 |
| **Comorbid conditions - Mood & anxiety disorders (no) (REF)** | 0.0000 | . | . | . | . | . |
| **Comorbid conditions – One or more of: asthma, diabetes, epilepsy, schizophrenia (yes)** | -3.7591 | 4.8544 | -0.7700 | 0.4388 | -13.2771 | 5.7589 |
| **Comorbid conditions – One or more of: asthma, diabetes, epilepsy, schizophrenia (no) (REF)** | 0.0000 | . | . | . | . | . |
| **Select medications (one or more)** | 1.8925 | 1.1040 | 1.7100 | 0.0866 | -0.2720 | 4.0571 |
| **Select medications (none) (REF)** | 0.0000 | . | . | . | . | . |
| **School District - Anglophone** | 8.9821 | 3.8429 | 2.3400 | 0.0195 | 1.4473 | 16.5169 |
| **School District – Francophone (REF)** | 0.0000 | . | . | . | . | . |
| **CIMD - Residential Instability Q2** | 0.9726 | 1.4272 | 0.6800 | 0.4956 | -1.8257 | 3.7709 |
| **CIMD - Residential Instability Q3** | -0.0924 | 1.5175 | -0.0600 | 0.9514 | -3.0678 | 2.8830 |
| **CIMD - Residential Instability Q4** | 0.2364 | 1.6732 | 0.1400 | 0.8876 | -3.0443 | 3.5171 |
| **CIMD – Residential Instability Q5 (most deprived)** | -0.3021 | 2.1261 | -0.1400 | 0.8870 | -4.4708 | 3.8665 |
| **CIMD - Residential Instability Q1 (least deprived) (REF)** | 0.0000 | . | . | . | . | . |
| **CIMD - Economic Dependency Q2** | -1.7204 | 1.8553 | -0.9300 | 0.3538 | -5.3581 | 1.9172 |
| **CIMD - Economic Dependency Q3** | -2.2367 | 1.7876 | -1.2500 | 0.2109 | -5.7418 | 1.2683 |
| **CIMD - Economic Dependency Q4** | -2.3440 | 1.8627 | -1.2600 | 0.2083 | -5.9962 | 1.3081 |
| **CIMD - Economic Dependency Q5 (most deprived)** | -5.3607 | 1.8844 | -2.8400 | 0.0045 | -9.0555 | -1.6659 |
| **CIMD - Economic Dependency Q1 (least deprived) (REF)** | 0.0000 | . | . | . | . | . |
| **CIMD - Ethnocultural Composition Q2** | -0.6512 | 1.0265 | -0.6300 | 0.5258 | -2.6638 | 1.3614 |
| **CIMD - Ethnocultural Composition Q3** | -1.5192 | 1.5442 | -0.9800 | 0.3253 | -4.5469 | 1.5085 |
| **CIMD - Ethnocultural Composition Q4** | -4.4405 | 2.2903 | -1.9400 | 0.0526 | -8.9312 | 0.0502 |
| **CIMD - Ethnocultural Composition Q5 (most deprived)** | -6.0498 | 2.9843 | -2.0300 | 0.0427 | -11.9012 | -0.1984 |
| **CIMD - Ethnocultural Composition Q1 (least deprived) (REF)** | 0.0000 | . | . | . | . | . |
| **CIMD -Situational Vulnerability Q2** | 2.0695 | 1.8002 | 1.1500 | 0.2504 | -1.4601 | 5.5992 |
| **CIMD - Situational Vulnerability Q3** | -1.1488 | 2.0164 | -0.5700 | 0.5689 | -5.1023 | 2.8048 |
| **CIMD -Situational Vulnerability Q4** | 2.1045 | 1.9191 | 1.1000 | 0.2729 | -1.6583 | 5.8674 |
| **CIMD -Situational Vulnerability Q5 (most deprived)** | 0.8274 | 2.0088 | 0.4100 | 0.6805 | -3.1113 | 4.7660 |
| **CIMD - Situational Vulnerability Q1 (least deprived) (REF)** | 0.0000 | . | . | . | . | . |
| **Social Assistance (any in past 5 years)** | 0.7198 | 1.0491 | 0.6900 | 0.4927 | -1.3372 | 2.7769 |
| **Social Assistance (none in past 5 years) (REF)** | 0.0000 | . | . | . | . | . |
| **Program of Study - French Immersion/Other** | -0.6706 | 1.5079 | -0.4400 | 0.6566 | -3.6272 | 2.2861 |
| **Program of Study - French** | 5.7115 | 3.8774 | 1.4700 | 0.1408 | -1.8910 | 13.3139 |
| **Program of Study - English (REF)** | 0.0000 | . | . | . | . | . |
| **Household composition – Adults (age 22+) – No adults in household** | 3.1319 | 3.0586 | 1.0200 | 0.3059 | -2.8650 | 9.1289 |
| **Household composition – Adults (age 22+) – One adult in household** | 0.1239 | 0.9584 | 0.1300 | 0.8971 | -1.7553 | 2.0031 |
| **Household composition – Adults (age 22+) – More than one adult in household (REF)** | 0.0000 | . | . | . | . | . |
| **Household composition – Children (age 21 or under) – Student is only child in household** | 0.0023 | 1.0536 | 0.0000 | 0.9983 | -2.0635 | 2.0680 |
| **Household composition – Children (age 21 or under) – Other children in household (REF)** | 0.0000 | . | . | . | . | . |
| **Recent immigrant** | 11.0965 | 11.2392 | 0.9900 | 0.3236 | -10.9404 | 33.1333 |
| **Not a recent immigrant (REF)** | 0.0000 | . | . | . | . | . |

**Supplementary Table S12e. GLM regression estimates – Absences per person due to unknown reasons – Grades K-12 (AY 2018-2020) (Untreated group as reference)**

| **Parameter** | **Estimate** | **Standard**  **Error** | **t Value** | **Pr > \|t\|** | **95% Confidence Limits** | |
| --- | --- | --- | --- | --- | --- | --- |
| **Intercept** | -86.6445 | 3.2976 | -26.2700 | <.0001 | -93.1079 | -80.1811 |
| **Treated ADHD** | -9.8160 | 0.5933 | -16.5500 | <.0001 | -10.9788 | -8.6531 |
| **Untreated ADHD (REF)** | 0.0000 | . | . | . | . | . |
| **Age** | 6.3866 | 0.0785 | 81.3700 | <.0001 | 6.2328 | 6.5405 |
| **Observation Time** | 0.0983 | 0.0037 | 26.5000 | <.0001 | 0.0910 | 0.1055 |
| **Male** | -1.6700 | 0.5880 | -2.8400 | 0.0045 | -2.8225 | -0.5175 |
| **Female (REF)** | 0.0000 | . | . | . | . | . |
| **Household income quintile Q2** | -2.3102 | 0.9912 | -2.3300 | 0.0198 | -4.2530 | -0.3673 |
| **Household income quintile Q3** | -1.1651 | 1.1096 | -1.0500 | 0.2937 | -3.3399 | 1.0098 |
| **Household income quintile Q4** | -2.8392 | 1.1879 | -2.3900 | 0.0169 | -5.1676 | -0.5109 |
| **Household income quintile Q5 (highest income)** | -1.8127 | 1.2968 | -1.4000 | 0.1622 | -4.3545 | 0.7291 |
| **Household income quintile Q1 (lowest income) (REF)** | 0.0000 | . | . | . | . | . |
| **NB Health Zone 2** | 18.6291 | 0.8316 | 22.4000 | <.0001 | 16.9992 | 20.2590 |
| **NB Health Zone 3** | 4.4963 | 0.8105 | 5.5500 | <.0001 | 2.9077 | 6.0850 |
| **NB Health Zone 4** | -1.1541 | 1.3825 | -0.8300 | 0.4038 | -3.8637 | 1.5556 |
| **NB Health Zone 5** | 1.2269 | 1.6738 | 0.7300 | 0.4636 | -2.0538 | 4.5075 |
| **NB Health Zone 6** | -3.1479 | 1.1526 | -2.7300 | 0.0063 | -5.4070 | -0.8888 |
| **NB Health Zone 7** | 2.8943 | 1.4114 | 2.0500 | 0.0403 | 0.1279 | 5.6607 |
| **NB Health Zone 1 (REF)** | 0.0000 | . | . | . | . | . |
| **Comorbid conditions - Mood & anxiety disorders (yes)** | 15.2247 | 1.8727 | 8.1300 | <.0001 | 11.5541 | 18.8953 |
| **Comorbid conditions - Mood & anxiety disorders (no) (REF)** | 0.0000 | . | . | . | . | . |
| **Comorbid conditions – One or more of: asthma, diabetes, epilepsy, schizophrenia (yes)** | -1.6624 | 3.3055 | -0.5000 | 0.6150 | -8.1413 | 4.8165 |
| **Comorbid conditions – One or more of: asthma, diabetes, epilepsy, schizophrenia (no) (REF)** | 0.0000 | . | . | . | . | . |
| **Select medications (one or more)** | 10.9176 | 0.8512 | 12.8300 | <.0001 | 9.2492 | 12.5861 |
| **Select medications (none) (REF)** | 0.0000 | . | . | . | . | . |
| **School District - Anglophone** | 12.8814 | 2.2245 | 5.7900 | <.0001 | 8.5213 | 17.2414 |
| **School District – Francophone (REF)** | 0.0000 | . | . | . | . | . |
| **CIMD - Residential Instability Q2** | 1.9379 | 0.8607 | 2.2500 | 0.0243 | 0.2510 | 3.6249 |
| **CIMD - Residential Instability Q3** | 3.1111 | 0.8976 | 3.4700 | 0.0005 | 1.3518 | 4.8704 |
| **CIMD - Residential Instability Q4** | 3.5620 | 1.0201 | 3.4900 | 0.0005 | 1.5625 | 5.5615 |
| **CIMD – Residential Instability Q5 (most deprived)** | 5.6246 | 1.3074 | 4.3000 | <.0001 | 3.0620 | 8.1872 |
| **CIMD - Residential Instability Q1 (least deprived) (REF)** | 0.0000 | . | . | . | . | . |
| **CIMD - Economic Dependency Q2** | -0.6137 | 1.0528 | -0.5800 | 0.5599 | -2.6773 | 1.4498 |
| **CIMD - Economic Dependency Q3** | 0.4427 | 1.0607 | 0.4200 | 0.6764 | -1.6362 | 2.5216 |
| **CIMD - Economic Dependency Q4** | -0.9865 | 1.0803 | -0.9100 | 0.3611 | -3.1039 | 1.1308 |
| **CIMD - Economic Dependency Q5 (most deprived)** | -0.9289 | 1.1117 | -0.8400 | 0.4034 | -3.1079 | 1.2501 |
| **CIMD - Economic Dependency Q1 (least deprived) (REF)** | 0.0000 | . | . | . | . | . |
| **CIMD - Ethnocultural Composition Q2** | -0.7215 | 0.6351 | -1.1400 | 0.2559 | -1.9663 | 0.5232 |
| **CIMD - Ethnocultural Composition Q3** | -2.2795 | 0.9147 | -2.4900 | 0.0127 | -4.0722 | -0.4867 |
| **CIMD - Ethnocultural Composition Q4** | -3.0014 | 1.3381 | -2.2400 | 0.0249 | -5.6241 | -0.3786 |
| **CIMD - Ethnocultural Composition Q5 (most deprived)** | -1.1751 | 1.8996 | -0.6200 | 0.5362 | -4.8984 | 2.5482 |
| **CIMD - Ethnocultural Composition Q1 (least deprived) (REF)** | 0.0000 | . | . | . | . | . |
| **CIMD -Situational Vulnerability Q2** | 1.7606 | 1.0343 | 1.7000 | 0.0887 | -0.2666 | 3.7878 |
| **CIMD - Situational Vulnerability Q3** | 0.3660 | 1.1430 | 0.3200 | 0.7488 | -1.8742 | 2.6062 |
| **CIMD -Situational Vulnerability Q4** | 2.9443 | 1.1176 | 2.6300 | 0.0084 | 0.7538 | 5.1348 |
| **CIMD -Situational Vulnerability Q5 (most deprived)** | 4.1998 | 1.1973 | 3.5100 | 0.0005 | 1.8530 | 6.5466 |
| **CIMD - Situational Vulnerability Q1 (least deprived) (REF)** | 0.0000 | . | . | . | . | . |
| **Social Assistance (any in past 5 years)** | 11.4990 | 0.7201 | 15.9700 | <.0001 | 10.0876 | 12.9104 |
| **Social Assistance (none in past 5 years) (REF)** | 0.0000 | . | . | . | . | . |
| **Program of Study - French Immersion/Other** | -11.5799 | 0.8136 | -14.2300 | <.0001 | -13.1746 | -9.9851 |
| **Program of Study - French** | 0.5228 | 2.2644 | 0.2300 | 0.8174 | -3.9155 | 4.9610 |
| **Program of Study - English (REF)** | 0.0000 | . | . | . | . | . |
| **Household composition – Adults (age 22+) – No adults in household** | -3.5908 | 2.1214 | -1.6900 | 0.0905 | -7.7487 | 0.5672 |
| **Household composition – Adults (age 22+) – One adult in household** | 3.7288 | 0.6166 | 6.0500 | <.0001 | 2.5202 | 4.9373 |
| **Household composition – Adults (age 22+) – More than one adult in household (REF)** | 0.0000 | . | . | . | . | . |
| **Household composition – Children (age 21 or under) – Student is only child in household** | -1.0172 | 0.6533 | -1.5600 | 0.1195 | -2.2976 | 0.2633 |
| **Household composition – Children (age 21 or under) – Other children in household (REF)** | 0.0000 | . | . | . | . | . |
| **Recent immigrant** | -4.5017 | 3.6383 | -1.2400 | 0.2160 | -11.6328 | 2.6294 |
| **Not a recent immigrant (REF)** | 0.0000 | . | . | . | . | . |
