# Supplementary material for: Academic Outcomes in Primary and Secondary School Students Prescribed Long-Acting Stimulants for ADHD Management
Source: J Atten Disord. 2025 Oct 7;30(4):493–505. doi: 10.1177/10870547251378169 (PMC12953683; doi:10.1177/10870547251378169)
Supplement: sj-docx-13-jad-10.1177_10870547251378169 – Supplemental material for Academic Outcomes in Primary and Secondary School Students Prescribed Long-Acting Stimulants for ADHD Management [file sj-docx-13-jad-10.1177_10870547251378169.docx]

**Supplementary Table S13. Logistic regression estimates - Likelihood of not transitioning to post-secondary education in NB (AY 2018)**

| **Odds Ratio Estimates** | | | |
| --- | --- | --- | --- |
| **Effect** | **Point Estimate** | **95% Wald**  **Confidence Limits** | |
| **Treated ADHD vs No ADHD** | 0.768 | 0.542 | 1.088 |
| **Untreated ADHD vs No ADHD** | 1.418 | 1.057 | 1.902 |
| **Age** | 1.008 | 0.74 | 1.374 |
| **Male vs Female** | 0.932 | 0.702 | 1.237 |
| **Household income quintile Q2 vs Q1 (lowest income)** | 1.095 | 0.674 | 1.781 |
| **Household income quintile Q3 vs Q1 (lowest income)** | 1.681 | 0.98 | 2.885 |
| **Household income quintile Q4 vs Q1 (lowest income)** | 1.298 | 0.724 | 2.325 |
| **Household income quintile Q5 (highest income) vs Q1 (lowest income)** | 0.935 | 0.501 | 1.745 |
| **NB Health Zone 2 vs Zone 1** | 0.769 | 0.521 | 1.134 |
| **NB Health Zone 3 vs Zone 1** | 0.732 | 0.502 | 1.066 |
| **NB Health Zone 4 vs Zone 1** | 0.893 | 0.47 | 1.695 |
| **NB Health Zone 5 vs Zone 1** | 0.223 | 0.099 | 0.502 |
| **NB Health Zone 6 vs Zone 1** | 0.642 | 0.38 | 1.083 |
| **NB Health Zone 7 vs Zone 1** | 0.438 | 0.218 | 0.88 |
| **Comorbid conditions – Mood & anxiety disorders - yes vs no** | 0.81 | 0.601 | 1.091 |
| **Comorbid conditions – One or more of: asthma, diabetes, epilepsy, schizophrenia - yes vs no** | 1.02 | 0.486 | 2.14 |
| **Select medications - yes vs no** | 1.999 | 1.281 | 3.117 |
| **School District - Anglophone vs Francophone** | 0.264 | 0.108 | 0.642 |
| **CIMD - Residential Instability Q2 vs Q1 (least deprived)** | 0.847 | 0.59 | 1.217 |
| **CIMD - Residential Instability Q3 vs Q1 (least deprived)** | 0.81 | 0.544 | 1.204 |
| **CIMD - Residential Instability Q4 vs Q1 (least deprived)** | 0.908 | 0.559 | 1.475 |
| **CIMD - Residential Instability Q5 (most deprived) vs Q1 (least deprived)** | 1.105 | 0.559 | 2.185 |
| **CIMD - Economic Dependency Q2 vs Q1 (least deprived)** | 0.955 | 0.6 | 1.521 |
| **CIMD - Economic Dependency Q3 vs Q1 (least deprived)** | 1.287 | 0.796 | 2.082 |
| **CIMD - Economic Dependency Q4 vs Q1 (least deprived)** | 1.25 | 0.755 | 2.068 |
| **CIMD - Economic Dependency Q5 (most deprived) vs Q1 (least deprived)** | 1.099 | 0.658 | 1.837 |
| **CIMD - Ethnocultural Composition Q2 vs Q1 (least deprived)** | 1.072 | 0.808 | 1.422 |
| **CIMD - Ethnocultural Composition Q3 vs Q1 (least deprived)** | 0.725 | 0.476 | 1.103 |
| **CIMD - Ethnocultural Composition Q4 vs Q1 (least deprived)** | 0.982 | 0.495 | 1.948 |
| **CIMD - Ethnocultural Composition Q5 (most deprived) vs Q1 (least deprived)** | 1.161 | 0.366 | 3.678 |
| **CIMD - Situational Vulnerability Q2 vs Q1 (least deprived)** | 0.979 | 0.624 | 1.537 |
| **CIMD - Situational Vulnerability Q3 vs Q1 (least deprived)** | 0.677 | 0.412 | 1.112 |
| **CIMD - Situational Vulnerability Q4 vs Q1 (least deprived)** | 0.94 | 0.569 | 1.552 |
| **CIMD - Situational Vulnerability Q5 (most deprived) vs Q1 (least deprived)** | 1.145 | 0.662 | 1.981 |
| **Social Assistance – any received in past 5 years - yes vs no** | 2.75 | 1.744 | 4.338 |
| **Program of Study - French Immersion/Other vs English** | 0.61 | 0.431 | 0.864 |
| **Program of Study - French vs English** | 0.108 | 0.044 | 0.262 |
| **Household composition – Adults (age 22+) – No adults in household vs More than one adult in household** | 1.923 | 0.974 | 3.795 |
| **Household composition – Adults (age 22+) – One adult in household vs More than one adult in household** | 0.86 | 0.613 | 1.207 |
| **Household composition - Children (age 21 or under) – Student is only child in household vs Other children in household** | 1.031 | 0.787 | 1.352 |
| **Recent immigrant vs Not a recent immigrant** | 0.568 | 0.141 | 2.284 |
