# Supplementary material for: Academic Outcomes in Primary and Secondary School Students Prescribed Long-Acting Stimulants for ADHD Management
Source: J Atten Disord. 2025 Oct 7;30(4):493–505. doi: 10.1177/10870547251378169 (PMC12953683; doi:10.1177/10870547251378169)
Supplement: sj-docx-14-jad-10.1177_10870547251378169 – Supplemental material for Academic Outcomes in Primary and Secondary School Students Prescribed Long-Acting Stimulants for ADHD Management [file sj-docx-14-jad-10.1177_10870547251378169.docx]

**Supplementary Table S14. Logistic regression estimates - Likelihood of not transitioning to post-secondary education in NB (AY 2018) (Untreated group as reference)**

| **Odds Ratio Estimates** | | | |
| --- | --- | --- | --- |
| **Effect** | **Point Estimate** | **95% Wald**  **Confidence Limits** | |
| **Treated ADHD vs Untreated ADHD** | 0.544 | 0.381 | 0.776 |
| **Age** | 1.489 | 0.978 | 2.265 |
| **Male vs Female** | 1.052 | 0.736 | 1.503 |
| **Household income quintile Q2 vs Q1 (lowest income)** | 0.727 | 0.386 | 1.37 |
| **Household income quintile Q3 vs Q1 (lowest income)** | 1.651 | 0.832 | 3.279 |
| **Household income quintile Q4 vs Q1 (lowest income)** | 1.131 | 0.534 | 2.395 |
| **Household income quintile Q5 (highest income) vs Q1 (lowest income)** | 0.882 | 0.395 | 1.968 |
| **NB Health Zone 2 vs Zone 1** | 1.089 | 0.646 | 1.835 |
| **NB Health Zone 3 vs Zone 1** | 0.787 | 0.476 | 1.299 |
| **NB Health Zone 4 vs Zone 1** | 0.589 | 0.258 | 1.344 |
| **NB Health Zone 5 vs Zone 1** | 0.185 | 0.072 | 0.476 |
| **NB Health Zone 6 vs Zone 1** | 0.513 | 0.269 | 0.978 |
| **NB Health Zone 7 vs Zone 1** | 0.503 | 0.194 | 1.301 |
| **Comorbid conditions – Mood & anxiety disorders - yes vs no** | 0.684 | 0.436 | 1.073 |
| **Comorbid conditions – One or more of: asthma, diabetes, epilepsy, schizophrenia - yes vs no** | 0.799 | 0.249 | 2.56 |
| **Select medications - yes vs no** | 1.784 | 0.962 | 3.309 |
| **School District - Anglophone vs Francophone** | 0.193 | 0.071 | 0.522 |
| **CIMD - Residential Instability Q2 vs Q1 (least deprived)** | 0.746 | 0.456 | 1.222 |
| **CIMD - Residential Instability Q3 vs Q1 (least deprived)** | 0.75 | 0.44 | 1.279 |
| **CIMD - Residential Instability Q4 vs Q1 (least deprived)** | 0.884 | 0.458 | 1.705 |
| **CIMD - Residential Instability Q5 (most deprived) vs Q1 (least deprived)** | 0.817 | 0.329 | 2.029 |
| **CIMD - Economic Dependency Q2 vs Q1 (least deprived)** | 0.961 | 0.526 | 1.756 |
| **CIMD - Economic Dependency Q3 vs Q1 (least deprived)** | 1.234 | 0.657 | 2.319 |
| **CIMD - Economic Dependency Q4 vs Q1 (least deprived)** | 1.396 | 0.723 | 2.695 |
| **CIMD - Economic Dependency Q5 (most deprived) vs Q1 (least deprived)** | 1.158 | 0.595 | 2.254 |
| **CIMD - Ethnocultural Composition Q2 vs Q1 (least deprived)** | 1.128 | 0.777 | 1.636 |
| **CIMD - Ethnocultural Composition Q3 vs Q1 (least deprived)** | 0.847 | 0.476 | 1.508 |
| **CIMD - Ethnocultural Composition Q4 vs Q1 (least deprived)** | 1.471 | 0.537 | 4.027 |
| **CIMD - Ethnocultural Composition Q5 (most deprived) vs Q1 (least deprived)** | 1.094 | 0.223 | 5.366 |
| **CIMD - Situational Vulnerability Q2 vs Q1 (least deprived)** | 1.011 | 0.55 | 1.856 |
| **CIMD - Situational Vulnerability Q3 vs Q1 (least deprived)** | 0.944 | 0.493 | 1.81 |
| **CIMD - Situational Vulnerability Q4 vs Q1 (least deprived)** | 1.015 | 0.52 | 1.981 |
| **CIMD - Situational Vulnerability Q5 (most deprived) vs Q1 (least deprived)** | 1.185 | 0.57 | 2.464 |
| **Social Assistance – any received in past 5 years - yes vs no** | 3.084 | 1.606 | 5.922 |
| **Program of Study - French Immersion/Other vs English** | 0.509 | 0.299 | 0.868 |
| **Program of Study - French vs English** | 0.096 | 0.035 | 0.264 |
| **Household composition – Adults (age 22+) – No adults in household vs More than one adult in household** | 0.963 | 0.406 | 2.285 |
| **Household composition – Adults (age 22+) – One adult in household vs More than one adult in household** | 0.939 | 0.606 | 1.453 |
| **Household composition - Children (age 21 or under) – Student is only child in household vs Other children in household** | 0.868 | 0.603 | 1.248 |
| **Recent immigrant vs Not a recent immigrant** | 0.549 | 0.077 | 3.901 |
