# Supplementary material for: Academic Outcomes in Primary and Secondary School Students Prescribed Long-Acting Stimulants for ADHD Management
Source: J Atten Disord. 2025 Oct 7;30(4):493–505. doi: 10.1177/10870547251378169 (PMC12953683; doi:10.1177/10870547251378169)
Supplement: sj-docx-15-jad-10.1177_10870547251378169 – Supplemental material for Academic Outcomes in Primary and Secondary School Students Prescribed Long-Acting Stimulants for ADHD Management [file sj-docx-15-jad-10.1177_10870547251378169.docx]

**Index of Supplementary Tables**

**S1a-b:** Full background characteristics for grades K-8 (**a**) and 9-12 (**b**) report card score cohorts

**S2a-h:** Full regression results for mean report card scores, Grades K-12 (No ADHD group as reference)

**a:** Grades K-8, overall scores (No ADHD group as reference)

**b:** Grades K-8, STEM subject scores (No ADHD group as reference)

**c:** Grades K-8, math scores (No ADHD group as reference)

**d:** Grades K-8, language scores (No ADHD group as reference)

**e:** Grades 9-12, overall scores (No ADHD group as reference)

**f:** Grades 9-12, STEM subject scores (No ADHD group as reference)

**g:** Grades 9-12, math scores (No ADHD group as reference)

**h:** Grades 9-12, language scores (No ADHD group as reference)

**S3a-d:** Full regression results for mean report card scores, Grades K-8 (Untreated group as reference)

**a:** Grades K-8, overall scores (Untreated group as reference)

**b:** Grades K-8, STEM subject scores (Untreated group as reference)

**c:** Grades K-8, math scores (Untreated group as reference)

**d:** Grades K-8, language scores (Untreated group as reference)

**S4a-h:** Full regression results for proportion of report card scores meeting minimum acceptable standard, Grades K-8

**a:** Grades K-8, overall scores (No ADHD group as reference)

**b:** Grades K-8, STEM subject scores (No ADHD group as reference)

**c:** Grades K-8, math scores (No ADHD group as reference)

**d:** Grades K-8, language scores (No ADHD group as reference)

**e:** Grades K-8, overall scores (Untreated group as reference)

**f:** Grades K-8, STEM subject scores (Untreated group as reference)

**g:** Grades K-8, math scores (Untreated group as reference)

**h:** Grades K-8, language scores (Untreated group as reference)

**S5a-d:** Full regression results for mean report card scores, Grades 9-12 (Untreated group as reference)

**a:** Grades 9-12, overall scores (Untreated group as reference)

**b:** Grades 9-12, STEM subject scores (Untreated group as reference)

**c:** Grades 9-12, math scores (Untreated group as reference)

**d:** Grades 9-12, language scores (Untreated group as reference)

**S6a-h:** Full regression results for proportion of courses failed, Grades 9-12

**a:** Grades 9-12, overall scores (No ADHD group as reference)

**b:** Grades 9-12, STEM subject scores (No ADHD group as reference)

**c:** Grades 9-12, math scores (No ADHD group as reference)

**d:** Grades 9-12, language scores (No ADHD group as reference)

**e:** Grades 9-12, overall scores (Untreated group as reference)

**f:** Grades 9-12, STEM subject scores (Untreated group as reference)

**g:** Grades 9-12, math scores (Untreated group as reference)

**h:** Grades 9-12, language scores (Untreated group as reference)

**S7a-h:** Full regression results for provincial assessment exam scores, Grades K-11 (No ADHD group as reference)

**a:** Grades K-8, overall scores (No ADHD group as reference)

**b:** Grades K-8, STEM subject scores (No ADHD group as reference)

**c:** Grades K-8, math scores (No ADHD group as reference)

**d:** Grades K-8, language scores (No ADHD group as reference)

**e:** Grades 9-11, overall scores (No ADHD group as reference)

**f:** Grades 9-11, STEM subject scores (No ADHD group as reference)

**g:** Grades 9-11, math scores (No ADHD group as reference)

**h:** Grades 9-11, language scores (No ADHD group as reference)

**S8a-h:** Full regression results for provincial assessment exam scores, Grades K-11 (Untreated group as reference)

**a:** Grades K-8, overall scores (Untreated group as reference)

**b:** Grades K-8, STEM subject scores (Untreated group as reference)

**c:** Grades K-8, math scores (Untreated group as reference)

**d:** Grades K-8, language scores (Untreated group as reference)

**e:** Grades 9-11, overall scores (Untreated group as reference)

**f:** Grades 9-11, STEM subject scores (Untreated group as reference)

**g:** Grades 9-11, math scores (Untreated group as reference)

**h:** Grades 9-11, language scores (Untreated group as reference)

**S9:** Full regression results for likelihood of not graduating from high school on first attempt (No ADHD group as reference)

**S10:** Full regression results for likelihood of not graduating from high school on first attempt (Untreated group as reference)

**S11a-e:** Full regression results for absences, Grades K-12 (No ADHD group as reference)

**a:** Grades K-12, any absence (No ADHD group as reference)

**b:** Grades K-12, absence due to illness (No ADHD group as reference)

**c:** Grades K-12, absence due to medical appointment (No ADHD group as reference)

**d:** Grades K-12, absence due to suspension (No ADHD group as reference)

**e:** Grades K-12, absence due to unknown reasons (No ADHD group as reference)

**S12a-e:** Full regression results for absences, Grades K-12 (Untreated group as reference)

**a:** Grades K-12, any absence (Untreated group as reference)

**b:** Grades K-12, absence due to illness (Untreated group as reference)

**c:** Grades K-12, absence due to medical appoint. (Untreated group as reference)

**d:** Grades K-12, absence due to suspension (Untreated group as reference)

**e:** Grades K-12, absence due to unknown reasons (Untreated group as reference)

**S13:** Full regression results for likelihood of not transitioning to post-secondary education in NB (No ADHD group as reference)

**S14:** Full regression results for likelihood of not transitioning to post-secondary education in NB (Untreated group as reference)
