# Supplementary material for: Academic Outcomes in Primary and Secondary School Students Prescribed Long-Acting Stimulants for ADHD Management
Source: J Atten Disord. 2025 Oct 7;30(4):493–505. doi: 10.1177/10870547251378169 (PMC12953683; doi:10.1177/10870547251378169)
Supplement: sj-docx-2-jad-10.1177_10870547251378169 – Supplemental material for Academic Outcomes in Primary and Secondary School Students Prescribed Long-Acting Stimulants for ADHD Management [file sj-docx-2-jad-10.1177_10870547251378169.docx]

**Supplementary Table S2a. GLM regression estimates – Mean overall report card score for grades K-8 (AY 2017 – 2020)**

| **Parameter** | **Estimate** | **Standard**  **Error** | **t Value** | **Pr > \|t\|** | **95% Confidence Limits** | |
| --- | --- | --- | --- | --- | --- | --- |
| **Intercept** | 3.0215 | 0.0095 | 319.1300 | <.0001 | 3.0029 | 3.0401 |
| **Treated ADHD** | -0.1746 | 0.0029 | -60.1500 | <.0001 | -0.1803 | -0.1689 |
| **Untreated ADHD** | -0.1516 | 0.0023 | -66.8700 | <.0001 | -0.1560 | -0.1472 |
| **No ADHD (REF)** | 0.0000 | . | . | . | . | . |
| **Age** | 0.0048 | 0.0003 | 17.1800 | <.0001 | 0.0042 | 0.0053 |
| **Male** | -0.0462 | 0.0013 | -34.9300 | <.0001 | -0.0488 | -0.0436 |
| **Female (REF)** | 0.0000 | . | . | . | . | . |
| **Household income quintile Q2** | 0.0140 | 0.0025 | 5.5700 | <.0001 | 0.0091 | 0.0190 |
| **Household income quintile Q3** | 0.0207 | 0.0028 | 7.4500 | <.0001 | 0.0152 | 0.0261 |
| **Household income quintile Q4** | 0.0252 | 0.0030 | 8.4700 | <.0001 | 0.0193 | 0.0310 |
| **Household income quintile Q5 (highest income)** | 0.0305 | 0.0032 | 9.5100 | <.0001 | 0.0243 | 0.0368 |
| **Household income quintile Q1 (lowest income) (REF)** | 0.0000 | . | . | . | . | . |
| **NB Health Zone 2** | 0.0011 | 0.0019 | 0.6000 | 0.5456 | -0.0025 | 0.0048 |
| **NB Health Zone 3** | 0.1008 | 0.0018 | 54.5400 | <.0001 | 0.0972 | 0.1044 |
| **NB Health Zone 4** | 0.0459 | 0.0041 | 11.2800 | <.0001 | 0.0379 | 0.0539 |
| **NB Health Zone 5** | 0.0792 | 0.0048 | 16.3800 | <.0001 | 0.0697 | 0.0886 |
| **NB Health Zone 6** | 0.0960 | 0.0034 | 28.3000 | <.0001 | 0.0893 | 0.1026 |
| **NB Health Zone 7** | 0.0492 | 0.0033 | 15.0200 | <.0001 | 0.0428 | 0.0556 |
| **NB Health Zone 1 (REF)** | 0.0000 | . | . | . | . | . |
| **Comorbid conditions - Mood & anxiety disorders (yes)** | -0.0162 | 0.0052 | -3.1000 | 0.0020 | -0.0265 | -0.0060 |
| **Comorbid conditions - Mood & anxiety disorders (no) (REF)** | 0.0000 | . | . | . | . | . |
| **Comorbid conditions – One or more of: asthma, diabetes, epilepsy, schizophrenia (yes)** | 0.0116 | 0.0065 | 1.7800 | 0.0758 | -0.0012 | 0.0244 |
| **Comorbid conditions – One or more of: asthma, diabetes, epilepsy, schizophrenia (no) (REF)** | 0.0000 | . | . | . | . | . |
| **Select medications (one or more)** | -0.0544 | 0.0047 | -11.6400 | <.0001 | -0.0635 | -0.0452 |
| **Select medications (none) (REF)** | 0.0000 | . | . | . | . | . |
| **School District - Anglophone** | -0.1323 | 0.0082 | -16.1700 | <.0001 | -0.1483 | -0.1163 |
| **School District – Francophone (REF)** | 0.0000 | . | . | . | . | . |
| **CIMD - Residential Instability Q2** | -0.0010 | 0.0020 | -0.5300 | 0.5956 | -0.0049 | 0.0028 |
| **CIMD - Residential Instability Q3** | -0.0050 | 0.0021 | -2.3600 | 0.0181 | -0.0091 | -0.0009 |
| **CIMD - Residential Instability Q4** | -0.0141 | 0.0024 | -5.7900 | <.0001 | -0.0189 | -0.0093 |
| **CIMD – Residential Instability Q5 (most deprived)** | 0.0034 | 0.0032 | 1.0500 | 0.2919 | -0.0029 | 0.0096 |
| **CIMD - Residential Instability Q1 (least deprived) (REF)** | 0.0000 | . | . | . | . | . |
| **CIMD - Economic Dependency Q2** | 0.0039 | 0.0024 | 1.6300 | 0.1036 | -0.0008 | 0.0086 |
| **CIMD - Economic Dependency Q3** | 0.0049 | 0.0024 | 2.0500 | 0.0404 | 0.0002 | 0.0097 |
| **CIMD - Economic Dependency Q4** | 0.0039 | 0.0025 | 1.5800 | 0.1139 | -0.0009 | 0.0088 |
| **CIMD - Economic Dependency Q5 (most deprived)** | 0.0059 | 0.0026 | 2.2900 | 0.0218 | 0.0009 | 0.0110 |
| **CIMD - Economic Dependency Q1 (least deprived) (REF)** | 0.0000 | . | . | . | . | . |
| **CIMD - Ethnocultural Composition Q2** | -0.0005 | 0.0015 | -0.3300 | 0.7450 | -0.0035 | 0.0025 |
| **CIMD - Ethnocultural Composition Q3** | 0.0037 | 0.0022 | 1.6900 | 0.0917 | -0.0006 | 0.0079 |
| **CIMD - Ethnocultural Composition Q4** | 0.0086 | 0.0032 | 2.7200 | 0.0066 | 0.0024 | 0.0148 |
| **CIMD - Ethnocultural Composition Q5 (most deprived)** | -0.0264 | 0.0045 | -5.8800 | <.0001 | -0.0353 | -0.0176 |
| **CIMD - Ethnocultural Composition Q1 (least deprived) (REF)** | 0.0000 | . | . | . | . | . |
| **CIMD -Situational Vulnerability Q2** | -0.0229 | 0.0023 | -9.9800 | <.0001 | -0.0274 | -0.0184 |
| **CIMD - Situational Vulnerability Q3** | -0.0338 | 0.0026 | -13.0400 | <.0001 | -0.0389 | -0.0287 |
| **CIMD -Situational Vulnerability Q4** | -0.0447 | 0.0025 | -17.6000 | <.0001 | -0.0497 | -0.0397 |
| **CIMD -Situational Vulnerability Q5 (most deprived)** | -0.0743 | 0.0028 | -26.4300 | <.0001 | -0.0798 | -0.0688 |
| **CIMD - Situational Vulnerability Q1 (least deprived) (REF)** | 0.0000 | . | . | . | . | . |
| **Social Assistance (any in past 5 years)** | -0.1317 | 0.0019 | -68.0300 | <.0001 | -0.1355 | -0.1279 |
| **Social Assistance (none in past 5 years) (REF)** | 0.0000 | . | . | . | . | . |
| **Program of Study - French Immersion/Other** | 0.0629 | 0.0022 | 28.4600 | <.0001 | 0.0586 | 0.0672 |
| **Program of Study - French** | 0.0169 | 0.0082 | 2.0700 | 0.0389 | 0.0009 | 0.0329 |
| **Program of Study - English (REF)** | 0.0000 | . | . | . | . | . |
| **Household composition – Adults (age 22+) – No adults in household** | -0.0729 | 0.0092 | -7.9500 | <.0001 | -0.0909 | -0.0549 |
| **Household composition – Adults (age 22+) – One adult in household** | -0.0489 | 0.0016 | -30.5900 | <.0001 | -0.0520 | -0.0457 |
| **Household composition – Adults (age 22+) – More than one adult in household (REF)** | 0.0000 | . | . | . | . | . |
| **Household composition – Children (age 21 or under) – Student is only child in household** | -0.0039 | 0.0018 | -2.1900 | 0.0286 | -0.0074 | -0.0004 |
| **Household composition – Children (age 21 or under) – Other children in household (REF)** | 0.0000 | . | . | . | . | . |
| **Recent immigrant** | -0.0039 | 0.0035 | -1.1300 | 0.2590 | -0.0107 | 0.0029 |
| **Not a recent immigrant (REF)** | 0.0000 | . | . | . | . | . |

**Supplementary Table S2b. GLM regression estimates – Mean report card score for STEM subjects for grades K-8 (AY 2017 – 2020)**

| **Parameter** | **Estimate** | **Standard**  **Error** | | **t Value** | **Pr > \|t\|** | **95% Confidence Limits** | |
| --- | --- | --- | --- | --- | --- | --- | --- |
| **Intercept** | 3.0745 | | 0.0129 | 238.3400 | <.0001 | 3.0492 | 3.0998 |
| **Treated ADHD** | -0.2023 | | 0.0039 | -51.7800 | <.0001 | -0.2099 | -0.1946 |
| **Untreated ADHD** | -0.1746 | | 0.0031 | -57.1100 | <.0001 | -0.1806 | -0.1686 |
| **No ADHD (REF)** | 0.0000 | | . | . | . | . | . |
| **Age** | -0.0005 | | 0.0004 | -1.2300 | 0.2177 | -0.0012 | 0.0003 |
| **Male** | 0.0046 | | 0.0018 | 2.6100 | 0.0091 | 0.0012 | 0.0081 |
| **Female (REF)** | 0.0000 | | . | . | . | . | . |
| **Household income quintile Q2** | 0.0171 | | 0.0034 | 5.0400 | <.0001 | 0.0104 | 0.0237 |
| **Household income quintile Q3** | 0.0240 | | 0.0037 | 6.4400 | <.0001 | 0.0167 | 0.0313 |
| **Household income quintile Q4** | 0.0273 | | 0.0040 | 6.8200 | <.0001 | 0.0194 | 0.0351 |
| **Household income quintile Q5 (highest income)** | 0.0341 | | 0.0043 | 7.8900 | <.0001 | 0.0256 | 0.0425 |
| **Household income quintile Q1 (lowest income) (REF)** | 0.0000 | | . | . | . | . | . |
| **NB Health Zone 2** | 0.0170 | | 0.0025 | 6.7400 | <.0001 | 0.0121 | 0.0220 |
| **NB Health Zone 3** | 0.1283 | | 0.0025 | 51.6000 | <.0001 | 0.1234 | 0.1331 |
| **NB Health Zone 4** | 0.0523 | | 0.0055 | 9.5600 | <.0001 | 0.0416 | 0.0631 |
| **NB Health Zone 5** | 0.1050 | | 0.0065 | 16.2000 | <.0001 | 0.0923 | 0.1177 |
| **NB Health Zone 6** | 0.0804 | | 0.0046 | 17.5700 | <.0001 | 0.0714 | 0.0894 |
| **NB Health Zone 7** | 0.0805 | | 0.0044 | 18.2700 | <.0001 | 0.0719 | 0.0891 |
| **NB Health Zone 1 (REF)** | 0.0000 | | . | . | . | . | . |
| **Comorbid conditions - Mood & anxiety disorders (yes)** | -0.0220 | | 0.0071 | -3.1200 | 0.0018 | -0.0359 | -0.0082 |
| **Comorbid conditions - Mood & anxiety disorders (no) (REF)** | 0.0000 | | . | . | . | . | . |
| **Comorbid conditions – One or more of: asthma, diabetes, epilepsy, schizophrenia (yes)** | 0.0063 | | 0.0088 | 0.7200 | 0.4744 | -0.0110 | 0.0235 |
| **Comorbid conditions – One or more of: asthma, diabetes, epilepsy, schizophrenia (no) (REF)** | 0.0000 | | . | . | . | . | . |
| **Select medications (one or more)** | -0.0470 | | 0.0064 | -7.3600 | <.0001 | -0.0595 | -0.0345 |
| **Select medications (none) (REF)** | 0.0000 | | . | . | . | . | . |
| **School District - Anglophone** | -0.1588 | | 0.0112 | -14.1800 | <.0001 | -0.1808 | -0.1369 |
| **School District – Francophone (REF)** | 0.0000 | | . | . | . | . | . |
| **CIMD - Residential Instability Q2** | -0.0028 | | 0.0026 | -1.0800 | 0.2820 | -0.0080 | 0.0023 |
| **CIMD - Residential Instability Q3** | -0.0041 | | 0.0028 | -1.4500 | 0.1481 | -0.0097 | 0.0015 |
| **CIMD - Residential Instability Q4** | -0.0162 | | 0.0033 | -4.9500 | <.0001 | -0.0226 | -0.0098 |
| **CIMD – Residential Instability Q5 (most deprived)** | 0.0065 | | 0.0043 | 1.5200 | 0.1279 | -0.0019 | 0.0150 |
| **CIMD - Residential Instability Q1 (least deprived) (REF)** | 0.0000 | | . | . | . | . | . |
| **CIMD - Economic Dependency Q2** | 0.0063 | | 0.0032 | 1.9400 | 0.0519 | -0.0001 | 0.0126 |
| **CIMD - Economic Dependency Q3** | 0.0050 | | 0.0032 | 1.5400 | 0.1229 | -0.0014 | 0.0113 |
| **CIMD - Economic Dependency Q4** | 0.0025 | | 0.0033 | 0.7400 | 0.4585 | -0.0041 | 0.0090 |
| **CIMD - Economic Dependency Q5 (most deprived)** | 0.0027 | | 0.0035 | 0.7900 | 0.4324 | -0.0041 | 0.0095 |
| **CIMD - Economic Dependency Q1 (least deprived) (REF)** | 0.0000 | | . | . | . | . | . |
| **CIMD - Ethnocultural Composition Q2** | -0.0013 | | 0.0021 | -0.6200 | 0.5357 | -0.0053 | 0.0028 |
| **CIMD - Ethnocultural Composition Q3** | 0.0024 | | 0.0029 | 0.8200 | 0.4098 | -0.0033 | 0.0081 |
| **CIMD - Ethnocultural Composition Q4** | 0.0062 | | 0.0043 | 1.4500 | 0.1470 | -0.0022 | 0.0145 |
| **CIMD - Ethnocultural Composition Q5 (most deprived)** | -0.0429 | | 0.0061 | -7.0500 | <.0001 | -0.0549 | -0.0310 |
| **CIMD - Ethnocultural Composition Q1 (least deprived) (REF)** | 0.0000 | | . | . | . | . | . |
| **CIMD -Situational Vulnerability Q2** | -0.0273 | | 0.0031 | -8.8700 | <.0001 | -0.0334 | -0.0213 |
| **CIMD - Situational Vulnerability Q3** | -0.0417 | | 0.0035 | -11.9600 | <.0001 | -0.0485 | -0.0348 |
| **CIMD -Situational Vulnerability Q4** | -0.0538 | | 0.0034 | -15.7700 | <.0001 | -0.0604 | -0.0471 |
| **CIMD -Situational Vulnerability Q5 (most deprived)** | -0.0882 | | 0.0038 | -23.3200 | <.0001 | -0.0956 | -0.0808 |
| **CIMD - Situational Vulnerability Q1 (least deprived) (REF)** | 0.0000 | | . | . | . | . | . |
| **Social Assistance (any in past 5 years)** | -0.1632 | | 0.0026 | -62.5700 | <.0001 | -0.1683 | -0.1581 |
| **Social Assistance (none in past 5 years) (REF)** | 0.0000 | | . | . | . | . | . |
| **Program of Study - French Immersion/Other** | 0.0541 | | 0.0030 | 18.2500 | <.0001 | 0.0483 | 0.0599 |
| **Program of Study - French** | -0.0333 | | 0.0112 | -2.9700 | 0.0029 | -0.0552 | -0.0113 |
| **Program of Study - English (REF)** | 0.0000 | | . | . | . | . | . |
| **Household composition – Adults (age 22+) – No adults in household** | -0.0686 | | 0.0123 | -5.5600 | <.0001 | -0.0928 | -0.0444 |
| **Household composition – Adults (age 22+) – One adult in household** | -0.0547 | | 0.0021 | -25.4800 | <.0001 | -0.0589 | -0.0505 |
| **Household composition – Adults (age 22+) – More than one adult in household (REF)** | 0.0000 | | . | . | . | . | . |
| **Household composition – Children (age 21 or under) – Student is only child in household** | -0.0021 | | 0.0024 | -0.8600 | 0.3900 | -0.0068 | 0.0026 |
| **Household composition – Children (age 21 or under) – Other children in household (REF)** | 0.0000 | | . | . | . | . | . |
| **Recent immigrant** | -0.0028 | | 0.0047 | -0.5900 | 0.5575 | -0.0120 | 0.0065 |
| **Not a recent immigrant (REF)** | 0.0000 | | . | . | . | . | . |

**Supplementary Table S2c. GLM regression estimates – Mean report card score for math for grades K-8 (AY 2017 – 2020)**

| **Parameter** | **Estimate** | **Standard**  **Error** | | **t Value** | **Pr > \|t\|** | **95% Confidence Limits** | |
| --- | --- | --- | --- | --- | --- | --- | --- |
| **Intercept** | 3.0948 | | 0.0154 | 201.5400 | <.0001 | 3.0647 | 3.1249 |
| **Treated ADHD** | -0.2320 | | 0.0046 | -49.9800 | <.0001 | -0.2411 | -0.2229 |
| **Untreated ADHD** | -0.2024 | | 0.0036 | -55.7400 | <.0001 | -0.2095 | -0.1953 |
| **No ADHD (REF)** | 0.0000 | | . | . | . | . | . |
| **Age** | -0.0058 | | 0.0004 | -13.0400 | <.0001 | -0.0067 | -0.0049 |
| **Male** | 0.0218 | | 0.0021 | 10.3600 | <.0001 | 0.0177 | 0.0260 |
| **Female (REF)** | 0.0000 | | . | . | . | . | . |
| **Household income quintile Q2** | 0.0194 | | 0.0040 | 4.8300 | <.0001 | 0.0115 | 0.0273 |
| **Household income quintile Q3** | 0.0303 | | 0.0044 | 6.8500 | <.0001 | 0.0216 | 0.0389 |
| **Household income quintile Q4** | 0.0332 | | 0.0047 | 7.0200 | <.0001 | 0.0239 | 0.0425 |
| **Household income quintile Q5 (highest income)** | 0.0414 | | 0.0051 | 8.0800 | <.0001 | 0.0313 | 0.0514 |
| **Household income quintile Q1 (lowest income) (REF)** | 0.0000 | | . | . | . | . | . |
| **NB Health Zone 2** | 0.0376 | | 0.0030 | 12.5800 | <.0001 | 0.0318 | 0.0435 |
| **NB Health Zone 3** | 0.1618 | | 0.0029 | 54.9000 | <.0001 | 0.1560 | 0.1676 |
| **NB Health Zone 4** | 0.0572 | | 0.0065 | 8.7900 | <.0001 | 0.0444 | 0.0699 |
| **NB Health Zone 5** | 0.1304 | | 0.0077 | 16.9700 | <.0001 | 0.1153 | 0.1454 |
| **NB Health Zone 6** | 0.0986 | | 0.0054 | 18.1700 | <.0001 | 0.0880 | 0.1093 |
| **NB Health Zone 7** | 0.1057 | | 0.0052 | 20.2600 | <.0001 | 0.0955 | 0.1159 |
| **NB Health Zone 1 (REF)** | 0.0000 | | . | . | . | . | . |
| **Comorbid conditions - Mood & anxiety disorders (yes)** | -0.0282 | | 0.0084 | -3.3500 | 0.0008 | -0.0446 | -0.0117 |
| **Comorbid conditions - Mood & anxiety disorders (no) (REF)** | 0.0000 | | . | . | . | . | . |
| **Comorbid conditions – One or more of: asthma, diabetes, epilepsy, schizophrenia (yes)** | 0.0117 | | 0.0105 | 1.1100 | 0.2652 | -0.0089 | 0.0322 |
| **Comorbid conditions – One or more of: asthma, diabetes, epilepsy, schizophrenia (no) (REF)** | 0.0000 | | . | . | . | . | . |
| **Select medications (one or more)** | -0.0368 | | 0.0076 | -4.8300 | <.0001 | -0.0518 | -0.0219 |
| **Select medications (none) (REF)** | 0.0000 | | . | . | . | . | . |
| **School District - Anglophone** | -0.1666 | | 0.0134 | -12.4800 | <.0001 | -0.1928 | -0.1405 |
| **School District – Francophone (REF)** | 0.0000 | | . | . | . | . | . |
| **CIMD - Residential Instability Q2** | -0.0060 | | 0.0031 | -1.9400 | 0.0519 | -0.0121 | 0.0001 |
| **CIMD - Residential Instability Q3** | -0.0078 | | 0.0034 | -2.3200 | 0.0203 | -0.0144 | -0.0012 |
| **CIMD - Residential Instability Q4** | -0.0204 | | 0.0039 | -5.2600 | <.0001 | -0.0280 | -0.0128 |
| **CIMD – Residential Instability Q5 (most deprived)** | 0.0026 | | 0.0051 | 0.5100 | 0.6100 | -0.0074 | 0.0126 |
| **CIMD - Residential Instability Q1 (least deprived) (REF)** | 0.0000 | | . | . | . | . | . |
| **CIMD - Economic Dependency Q2** | 0.0054 | | 0.0038 | 1.4100 | 0.1588 | -0.0021 | 0.0129 |
| **CIMD - Economic Dependency Q3** | 0.0036 | | 0.0038 | 0.9400 | 0.3485 | -0.0039 | 0.0111 |
| **CIMD - Economic Dependency Q4** | 0.0032 | | 0.0040 | 0.8000 | 0.4222 | -0.0046 | 0.0110 |
| **CIMD - Economic Dependency Q5 (most deprived)** | 0.0042 | | 0.0041 | 1.0300 | 0.3046 | -0.0038 | 0.0123 |
| **CIMD - Economic Dependency Q1 (least deprived) (REF)** | 0.0000 | | . | . | . | . | . |
| **CIMD - Ethnocultural Composition Q2** | -0.0022 | | 0.0025 | -0.8800 | 0.3787 | -0.0070 | 0.0026 |
| **CIMD - Ethnocultural Composition Q3** | 0.0023 | | 0.0035 | 0.6700 | 0.5020 | -0.0045 | 0.0091 |
| **CIMD - Ethnocultural Composition Q4** | 0.0049 | | 0.0050 | 0.9800 | 0.3292 | -0.0050 | 0.0148 |
| **CIMD - Ethnocultural Composition Q5 (most deprived)** | -0.0403 | | 0.0072 | -5.5600 | <.0001 | -0.0545 | -0.0261 |
| **CIMD - Ethnocultural Composition Q1 (least deprived) (REF)** | 0.0000 | | . | . | . | . | . |
| **CIMD -Situational Vulnerability Q2** | -0.0335 | | 0.0037 | -9.1900 | <.0001 | -0.0407 | -0.0264 |
| **CIMD - Situational Vulnerability Q3** | -0.0481 | | 0.0041 | -11.6300 | <.0001 | -0.0562 | -0.0400 |
| **CIMD -Situational Vulnerability Q4** | -0.0640 | | 0.0040 | -15.8400 | <.0001 | -0.0719 | -0.0561 |
| **CIMD -Situational Vulnerability Q5 (most deprived)** | -0.1020 | | 0.0045 | -22.7600 | <.0001 | -0.1108 | -0.0932 |
| **CIMD - Situational Vulnerability Q1 (least deprived) (REF)** | 0.0000 | | . | . | . | . | . |
| **Social Assistance (any in past 5 years)** | -0.1825 | | 0.0031 | -58.9200 | <.0001 | -0.1886 | -0.1764 |
| **Social Assistance (none in past 5 years) (REF)** | 0.0000 | | . | . | . | . | . |
| **Program of Study - French Immersion/Other** | 0.0620 | | 0.0035 | 17.6500 | <.0001 | 0.0551 | 0.0688 |
| **Program of Study - French** | -0.0213 | | 0.0133 | -1.6000 | 0.1106 | -0.0474 | 0.0049 |
| **Program of Study - English (REF)** | 0.0000 | | . | . | . | . | . |
| **Household composition – Adults (age 22+) – No adults in household** | -0.0521 | | 0.0147 | -3.5600 | 0.0004 | -0.0809 | -0.0234 |
| **Household composition – Adults (age 22+) – One adult in household** | -0.0602 | | 0.0025 | -23.6300 | <.0001 | -0.0652 | -0.0552 |
| **Household composition – Adults (age 22+) – More than one adult in household (REF)** | 0.0000 | | . | . | . | . | . |
| **Household composition – Children (age 21 or under) – Student is only child in household** | -0.0064 | | 0.0028 | -2.2300 | 0.0255 | -0.0119 | -0.0008 |
| **Household composition – Children (age 21 or under) – Other children in household (REF)** | 0.0000 | | . | . | . | . | . |
| **Recent immigrant** | 0.0103 | | 0.0056 | 1.8400 | 0.0656 | -0.0007 | 0.0213 |
| **Not a recent immigrant (REF)** | 0.0000 | | . | . | . | . | . |

**Supplementary Table S2d. GLM regression estimates – Mean report card score for language for grades K-8 (AY 2017 – 2020)**

| **Parameter** | **Estimate** | **Standard**  **Error** | **t Value** | **Pr > \|t\|** | **95% Confidence Limits** | |
| --- | --- | --- | --- | --- | --- | --- |
| **Intercept** | 2.8621 | 0.0149 | 192.1400 | <.0001 | 2.8329 | 2.8913 |
| **Treated ADHD** | -0.2694 | 0.0045 | -59.8900 | <.0001 | -0.2782 | -0.2606 |
| **Untreated ADHD** | -0.2269 | 0.0035 | -64.4900 | <.0001 | -0.2338 | -0.2200 |
| **No ADHD (REF)** | 0.0000 | . | . | . | . | . |
| **Age** | 0.0144 | 0.0004 | 33.4000 | <.0001 | 0.0136 | 0.0153 |
| **Male** | -0.1169 | 0.0020 | -57.1900 | <.0001 | -0.1209 | -0.1129 |
| **Female (REF)** | 0.0000 | . | . | . | . | . |
| **Household income quintile Q2** | 0.0110 | 0.0039 | 2.8100 | 0.0049 | 0.0033 | 0.0186 |
| **Household income quintile Q3** | 0.0179 | 0.0043 | 4.1800 | <.0001 | 0.0095 | 0.0263 |
| **Household income quintile Q4** | 0.0282 | 0.0046 | 6.1300 | <.0001 | 0.0192 | 0.0372 |
| **Household income quintile Q5 (highest income)** | 0.0390 | 0.0050 | 7.8600 | <.0001 | 0.0293 | 0.0487 |
| **Household income quintile Q1 (lowest income) (REF)** | 0.0000 | . | . | . | . | . |
| **NB Health Zone 2** | 0.0048 | 0.0029 | 1.6500 | 0.0992 | -0.0009 | 0.0105 |
| **NB Health Zone 3** | 0.1147 | 0.0029 | 40.0900 | <.0001 | 0.1091 | 0.1203 |
| **NB Health Zone 4** | 0.0418 | 0.0063 | 6.6200 | <.0001 | 0.0294 | 0.0541 |
| **NB Health Zone 5** | 0.0589 | 0.0075 | 7.9000 | <.0001 | 0.0443 | 0.0736 |
| **NB Health Zone 6** | 0.1098 | 0.0053 | 20.8700 | <.0001 | 0.0995 | 0.1202 |
| **NB Health Zone 7** | 0.0776 | 0.0051 | 15.3100 | <.0001 | 0.0676 | 0.0875 |
| **NB Health Zone 1 (REF)** | 0.0000 | . | . | . | . | . |
| **Comorbid conditions - Mood & anxiety disorders (yes)** | -0.0081 | 0.0081 | -0.9900 | 0.3215 | -0.0240 | 0.0079 |
| **Comorbid conditions - Mood & anxiety disorders (no) (REF)** | 0.0000 | . | . | . | . | . |
| **Comorbid conditions – One or more of: asthma, diabetes, epilepsy, schizophrenia (yes)** | 0.0235 | 0.0101 | 2.3200 | 0.0205 | 0.0036 | 0.0434 |
| **Comorbid conditions – One or more of: asthma, diabetes, epilepsy, schizophrenia (no) (REF)** | 0.0000 | . | . | . | . | . |
| **Select medications (one or more)** | -0.0472 | 0.0074 | -6.4000 | <.0001 | -0.0617 | -0.0328 |
| **Select medications (none) (REF)** | 0.0000 | . | . | . | . | . |
| **School District - Anglophone** | -0.0951 | 0.0129 | -7.3400 | <.0001 | -0.1205 | -0.0697 |
| **School District – Francophone (REF)** | 0.0000 | . | . | . | . | . |
| **CIMD - Residential Instability Q2** | -0.0018 | 0.0030 | -0.5900 | 0.5569 | -0.0077 | 0.0041 |
| **CIMD - Residential Instability Q3** | 0.0012 | 0.0033 | 0.3800 | 0.7046 | -0.0052 | 0.0076 |
| **CIMD - Residential Instability Q4** | -0.0107 | 0.0038 | -2.8400 | 0.0046 | -0.0181 | -0.0033 |
| **CIMD – Residential Instability Q5 (most deprived)** | 0.0047 | 0.0049 | 0.9500 | 0.3403 | -0.0050 | 0.0144 |
| **CIMD - Residential Instability Q1 (least deprived) (REF)** | 0.0000 | . | . | . | . | . |
| **CIMD - Economic Dependency Q2** | -0.0022 | 0.0037 | -0.6000 | 0.5464 | -0.0095 | 0.0050 |
| **CIMD - Economic Dependency Q3** | -0.0025 | 0.0037 | -0.6800 | 0.4979 | -0.0098 | 0.0048 |
| **CIMD - Economic Dependency Q4** | -0.0053 | 0.0039 | -1.3700 | 0.1711 | -0.0128 | 0.0023 |
| **CIMD - Economic Dependency Q5 (most deprived)** | -0.0038 | 0.0040 | -0.9400 | 0.3480 | -0.0116 | 0.0041 |
| **CIMD - Economic Dependency Q1 (least deprived) (REF)** | 0.0000 | . | . | . | . | . |
| **CIMD - Ethnocultural Composition Q2** | 0.0019 | 0.0024 | 0.8200 | 0.4147 | -0.0027 | 0.0066 |
| **CIMD - Ethnocultural Composition Q3** | 0.0042 | 0.0034 | 1.2600 | 0.2080 | -0.0024 | 0.0108 |
| **CIMD - Ethnocultural Composition Q4** | 0.0036 | 0.0049 | 0.7400 | 0.4615 | -0.0060 | 0.0132 |
| **CIMD - Ethnocultural Composition Q5 (most deprived)** | -0.0645 | 0.0070 | -9.1600 | <.0001 | -0.0783 | -0.0507 |
| **CIMD - Ethnocultural Composition Q1 (least deprived) (REF)** | 0.0000 | . | . | . | . | . |
| **CIMD -Situational Vulnerability Q2** | -0.0336 | 0.0035 | -9.4900 | <.0001 | -0.0406 | -0.0267 |
| **CIMD - Situational Vulnerability Q3** | -0.0466 | 0.0040 | -11.6300 | <.0001 | -0.0545 | -0.0388 |
| **CIMD -Situational Vulnerability Q4** | -0.0646 | 0.0039 | -16.4600 | <.0001 | -0.0723 | -0.0569 |
| **CIMD -Situational Vulnerability Q5 (most deprived)** | -0.1009 | 0.0043 | -23.2100 | <.0001 | -0.1094 | -0.0924 |
| **CIMD - Situational Vulnerability Q1 (least deprived) (REF)** | 0.0000 | . | . | . | . | . |
| **Social Assistance (any in past 5 years)** | -0.2070 | 0.0030 | -68.9100 | <.0001 | -0.2129 | -0.2011 |
| **Social Assistance (none in past 5 years) (REF)** | 0.0000 | . | . | . | . | . |
| **Program of Study - French Immersion/Other** | 0.0634 | 0.0034 | 18.5900 | <.0001 | 0.0567 | 0.0700 |
| **Program of Study - French** | 0.0745 | 0.0129 | 5.7600 | <.0001 | 0.0492 | 0.0999 |
| **Program of Study - English (REF)** | 0.0000 | . | . | . | . | . |
| **Household composition – Adults (age 22+) – No adults in household** | -0.1208 | 0.0142 | -8.5100 | <.0001 | -0.1487 | -0.0930 |
| **Household composition – Adults (age 22+) – One adult in household** | -0.0703 | 0.0025 | -28.4700 | <.0001 | -0.0752 | -0.0655 |
| **Household composition – Adults (age 22+) – More than one adult in household (REF)** | 0.0000 | . | . | . | . | . |
| **Household composition – Children (age 21 or under) – Student is only child in household** | 0.0094 | 0.0028 | 3.4000 | 0.0007 | 0.0040 | 0.0148 |
| **Household composition – Children (age 21 or under) – Other children in household (REF)** | 0.0000 | . | . | . | . | . |
| **Recent immigrant** | -0.0306 | 0.0055 | -5.5900 | <.0001 | -0.0414 | -0.0199 |
| **Not a recent immigrant (REF)** | 0.0000 | . | . | . | . | . |

**Supplementary Table S2e. GLM regression estimates – Mean overall report card score for grades 9-12 (AY 2017 – 2020)**

| **Parameter** | **Estimate** | **Standard**  **Error** | **t Value** | **Pr > \|t\|** | **95% Confidence Limits** | |
| --- | --- | --- | --- | --- | --- | --- |
| **Intercept** | 88.1710 | 0.8053 | 109.4900 | <.0001 | 86.5926 | 89.7494 |
| **Treated ADHD** | -4.9256 | 0.1879 | -26.2200 | <.0001 | -5.2938 | -4.5574 |
| **Untreated ADHD** | -6.1929 | 0.1288 | -48.0700 | <.0001 | -6.4454 | -5.9404 |
| **No ADHD (REF)** | 0.0000 | . | . | . | . | . |
| **Age** | -0.4410 | 0.0353 | -12.4800 | <.0001 | -0.5103 | -0.3717 |
| **Male** | -4.1666 | 0.0870 | -47.8700 | <.0001 | -4.3372 | -3.9960 |
| **Female (REF)** | 0.0000 | . | . | . | . | . |
| **Household income quintile Q2** | 1.3064 | 0.1643 | 7.9500 | <.0001 | 0.9843 | 1.6285 |
| **Household income quintile Q3** | 1.5018 | 0.1779 | 8.4400 | <.0001 | 1.1531 | 1.8505 |
| **Household income quintile Q4** | 2.1268 | 0.1914 | 11.1100 | <.0001 | 1.7516 | 2.5020 |
| **Household income quintile Q5 (highest income)** | 3.2256 | 0.2082 | 15.4900 | <.0001 | 2.8176 | 3.6337 |
| **Household income quintile Q1 (lowest income) (REF)** | 0.0000 | . | . | . | . | . |
| **NB Health Zone 2** | 1.7213 | 0.1269 | 13.5700 | <.0001 | 1.4726 | 1.9700 |
| **NB Health Zone 3** | 2.1878 | 0.1257 | 17.4000 | <.0001 | 1.9413 | 2.4342 |
| **NB Health Zone 4** | 2.9563 | 0.2090 | 14.1400 | <.0001 | 2.5465 | 3.3660 |
| **NB Health Zone 5** | 2.1176 | 0.2699 | 7.8500 | <.0001 | 1.5887 | 2.6465 |
| **NB Health Zone 6** | 3.1029 | 0.1900 | 16.3300 | <.0001 | 2.7306 | 3.4752 |
| **NB Health Zone 7** | 2.0283 | 0.2052 | 9.8800 | <.0001 | 1.6261 | 2.4304 |
| **NB Health Zone 1 (REF)** | 0.0000 | . | . | . | . | . |
| **Comorbid conditions - Mood & anxiety disorders (yes)** | -3.5446 | 0.1797 | -19.7200 | <.0001 | -3.8969 | -3.1924 |
| **Comorbid conditions - Mood & anxiety disorders (no) (REF)** | 0.0000 | . | . | . | . | . |
| **Comorbid conditions – One or more of: asthma, diabetes, epilepsy, schizophrenia (yes)** | 0.4209 | 0.5041 | 0.8300 | 0.4037 | -0.5671 | 1.4089 |
| **Comorbid conditions – One or more of: asthma, diabetes, epilepsy, schizophrenia (no) (REF)** | 0.0000 | . | . | . | . | . |
| **Select medications (one or more)** | -2.8842 | 0.1985 | -14.5300 | <.0001 | -3.2732 | -2.4953 |
| **Select medications (none) (REF)** | 0.0000 | . | . | . | . | . |
| **School District - Anglophone** | -3.0225 | 0.4965 | -6.0900 | <.0001 | -3.9956 | -2.0495 |
| **School District – Francophone (REF)** | 0.0000 | . | . | . | . | . |
| **CIMD - Residential Instability Q2** | -0.0143 | 0.1269 | -0.1100 | 0.9104 | -0.2630 | 0.2344 |
| **CIMD - Residential Instability Q3** | 0.1689 | 0.1365 | 1.2400 | 0.2160 | -0.0987 | 0.4364 |
| **CIMD - Residential Instability Q4** | 0.1282 | 0.1592 | 0.8100 | 0.4206 | -0.1838 | 0.4403 |
| **CIMD – Residential Instability Q5 (most deprived)** | -0.1489 | 0.2180 | -0.6800 | 0.4946 | -0.5762 | 0.2784 |
| **CIMD - Residential Instability Q1 (least deprived) (REF)** | 0.0000 | . | . | . | . | . |
| **CIMD - Economic Dependency Q2** | -0.2783 | 0.0984 | -2.8300 | 0.0047 | -0.4712 | -0.0854 |
| **CIMD - Economic Dependency Q3** | 0.2281 | 0.1429 | 1.6000 | 0.1105 | -0.0520 | 0.5082 |
| **CIMD - Economic Dependency Q4** | 1.5025 | 0.2132 | 7.0500 | <.0001 | 1.0846 | 1.9203 |
| **CIMD - Economic Dependency Q5 (most deprived)** | 1.2537 | 0.3331 | 3.7600 | 0.0002 | 0.6008 | 1.9067 |
| **CIMD - Economic Dependency Q1 (least deprived) (REF)** | 0.0000 | . | . | . | . | . |
| **CIMD - Ethnocultural Composition Q2** | -0.3307 | 0.1614 | -2.0500 | 0.0405 | -0.6471 | -0.0143 |
| **CIMD - Ethnocultural Composition Q3** | 0.0784 | 0.1619 | 0.4800 | 0.6281 | -0.2389 | 0.3957 |
| **CIMD - Ethnocultural Composition Q4** | -0.0520 | 0.1681 | -0.3100 | 0.7572 | -0.3814 | 0.2775 |
| **CIMD - Ethnocultural Composition Q5 (most deprived)** | 0.8492 | 0.1713 | 4.9600 | <.0001 | 0.5135 | 1.1849 |
| **CIMD - Ethnocultural Composition Q1 (least deprived) (REF)** | 0.0000 | . | . | . | . | . |
| **CIMD -Situational Vulnerability Q2** | -1.1025 | 0.1529 | -7.2100 | <.0001 | -1.4021 | -0.8029 |
| **CIMD - Situational Vulnerability Q3** | -1.3132 | 0.1718 | -7.6500 | <.0001 | -1.6499 | -0.9765 |
| **CIMD -Situational Vulnerability Q4** | -1.8107 | 0.1677 | -10.8000 | <.0001 | -2.1393 | -1.4821 |
| **CIMD -Situational Vulnerability Q5 (most deprived)** | -2.7168 | 0.1831 | -14.8400 | <.0001 | -3.0757 | -2.3579 |
| **CIMD - Situational Vulnerability Q1 (least deprived) (REF)** | 0.0000 | . | . | . | . | . |
| **Social Assistance (any in past 5 years)** | -7.3167 | 0.1447 | -50.5600 | <.0001 | -7.6004 | -7.0331 |
| **Social Assistance (none in past 5 years) (REF)** | 0.0000 | . | . | . | . | . |
| **Program of Study - French Immersion** | 4.9482 | 0.1087 | 45.5100 | <.0001 | 4.7351 | 5.1613 |
| **Program of Study -Other** | 2.8817 | 0.6517 | 4.4200 | <.0001 | 1.6044 | 4.1589 |
| **Program of Study - French** | 2.1175 | 0.4943 | 4.2800 | <.0001 | 1.1486 | 3.0864 |
| **Program of Study - English (REF)** | 0.0000 | . | . | . | . | . |
| **Household composition – Adults (age 22+) – No adults in household** | -3.2306 | 0.3653 | -8.8400 | <.0001 | -3.9466 | -2.5145 |
| **Household composition – Adults (age 22+) – One adult in household** | -2.9519 | 0.1112 | -26.5600 | <.0001 | -3.1697 | -2.7340 |
| **Household composition – Adults (age 22+) – More than one adult in household (REF)** | 0.0000 | . | . | . | . | . |
| **Household composition – Children (age 21 or under) – Student is only child in household** | -0.4172 | 0.1013 | -4.1200 | <.0001 | -0.6157 | -0.2187 |
| **Household composition – Children (age 21 or under) – Other children in household (REF)** | 0.0000 | . | . | . | . | . |
| **Recent immigrant** | 3.3180 | 0.2685 | 12.3600 | <.0001 | 2.7916 | 3.8443 |
| **Not a recent immigrant (REF)** | 0.0000 | . | . | . | . | . |

**Supplementary Table S2f. GLM regression estimates – Mean report card score for STEM subjects for grades 9-12 (AY 2017 – 2020)**

| **Parameter** | **Estimate** | **Standard**  **Error** | **t Value** | **Pr > \|t\|** | **95% Confidence Limits** | |
| --- | --- | --- | --- | --- | --- | --- |
| **Intercept** | 86.9543 | 0.9995 | 86.9900 | <.0001 | 84.9952 | 88.9134 |
| **Treated ADHD** | -5.3817 | 0.2355 | -22.8500 | <.0001 | -5.8434 | -4.9201 |
| **Untreated ADHD** | -6.1624 | 0.1639 | -37.5900 | <.0001 | -6.4836 | -5.8411 |
| **No ADHD (REF)** | 0.0000 | . | . | . | . | . |
| **Age** | -0.7176 | 0.0445 | -16.1200 | <.0001 | -0.8049 | -0.6303 |
| **Male** | -3.1558 | 0.1077 | -29.3100 | <.0001 | -3.3669 | -2.9448 |
| **Female (REF)** | 0.0000 | . | . | . | . | . |
| **Household income quintile Q2** | 1.4254 | 0.2056 | 6.9300 | <.0001 | 1.0224 | 1.8284 |
| **Household income quintile Q3** | 1.7851 | 0.2218 | 8.0500 | <.0001 | 1.3504 | 2.2199 |
| **Household income quintile Q4** | 2.3267 | 0.2386 | 9.7500 | <.0001 | 1.8590 | 2.7944 |
| **Household income quintile Q5 (highest income)** | 3.5202 | 0.2590 | 13.5900 | <.0001 | 3.0126 | 4.0277 |
| **Household income quintile Q1 (lowest income) (REF)** | 0.0000 | . | . | . | . | . |
| **NB Health Zone 2** | 0.8981 | 0.1570 | 5.7200 | <.0001 | 0.5904 | 1.2059 |
| **NB Health Zone 3** | 1.2611 | 0.1552 | 8.1300 | <.0001 | 0.9570 | 1.5652 |
| **NB Health Zone 4** | 3.8535 | 0.2625 | 14.6800 | <.0001 | 3.3391 | 4.3680 |
| **NB Health Zone 5** | 2.3450 | 0.3370 | 6.9600 | <.0001 | 1.6845 | 3.0056 |
| **NB Health Zone 6** | 2.6551 | 0.2377 | 11.1700 | <.0001 | 2.1893 | 3.1209 |
| **NB Health Zone 7** | 1.6507 | 0.2542 | 6.4900 | <.0001 | 1.1525 | 2.1488 |
| **NB Health Zone 1 (REF)** | 0.0000 | . | . | . | . | . |
| **Comorbid conditions - Mood & anxiety disorders (yes)** | -3.4569 | 0.2265 | -15.2600 | <.0001 | -3.9008 | -3.0129 |
| **Comorbid conditions - Mood & anxiety disorders (no) (REF)** | 0.0000 | . | . | . | . | . |
| **Comorbid conditions – One or more of: asthma, diabetes, epilepsy, schizophrenia (yes)** | 1.2991 | 0.6367 | 2.0400 | 0.0413 | 0.0513 | 2.5470 |
| **Comorbid conditions – One or more of: asthma, diabetes, epilepsy, schizophrenia (no) (REF)** | 0.0000 | . | . | . | . | . |
| **Select medications (one or more)** | -2.1662 | 0.2541 | -8.5200 | <.0001 | -2.6643 | -1.6681 |
| **Select medications (none) (REF)** | 0.0000 | . | . | . | . | . |
| **School District - Anglophone** | -0.2777 | 0.6077 | -0.4600 | 0.6477 | -1.4688 | 0.9134 |
| **School District – Francophone (REF)** | 0.0000 | . | . | . | . | . |
| **CIMD - Residential Instability Q2** | 0.2224 | 0.1564 | 1.4200 | 0.1551 | -0.0842 | 0.5289 |
| **CIMD - Residential Instability Q3** | 0.3988 | 0.1688 | 2.3600 | 0.0182 | 0.0679 | 0.7296 |
| **CIMD - Residential Instability Q4** | 0.7773 | 0.1969 | 3.9500 | <.0001 | 0.3915 | 1.1632 |
| **CIMD – Residential Instability Q5 (most deprived)** | 0.2967 | 0.2717 | 1.0900 | 0.2747 | -0.2358 | 0.8292 |
| **CIMD - Residential Instability Q1 (least deprived) (REF)** | 0.0000 | . | . | . | . | . |
| **CIMD - Economic Dependency Q2** | -0.0268 | 0.1218 | -0.2200 | 0.8257 | -0.2657 | 0.2120 |
| **CIMD - Economic Dependency Q3** | 0.5632 | 0.1764 | 3.1900 | 0.0014 | 0.2174 | 0.9089 |
| **CIMD - Economic Dependency Q4** | 1.8874 | 0.2626 | 7.1900 | <.0001 | 1.3728 | 2.4020 |
| **CIMD - Economic Dependency Q5 (most deprived)** | 1.1507 | 0.4166 | 2.7600 | 0.0057 | 0.3342 | 1.9672 |
| **CIMD - Economic Dependency Q1 (least deprived) (REF)** | 0.0000 | . | . | . | . | . |
| **CIMD - Ethnocultural Composition Q2** | -0.3399 | 0.1983 | -1.7100 | 0.0865 | -0.7286 | 0.0487 |
| **CIMD - Ethnocultural Composition Q3** | 0.2334 | 0.1986 | 1.1800 | 0.2398 | -0.1558 | 0.6227 |
| **CIMD - Ethnocultural Composition Q4** | 0.2105 | 0.2070 | 1.0200 | 0.3091 | -0.1951 | 0.6161 |
| **CIMD - Ethnocultural Composition Q5 (most deprived)** | 0.9858 | 0.2109 | 4.6700 | <.0001 | 0.5725 | 1.3992 |
| **CIMD - Ethnocultural Composition Q1 (least deprived) (REF)** | 0.0000 | . | . | . | . | . |
| **CIMD -Situational Vulnerability Q2** | -1.5614 | 0.1875 | -8.3300 | <.0001 | -1.9289 | -1.1939 |
| **CIMD - Situational Vulnerability Q3** | -1.8596 | 0.2112 | -8.8100 | <.0001 | -2.2736 | -1.4457 |
| **CIMD -Situational Vulnerability Q4** | -2.0094 | 0.2067 | -9.7200 | <.0001 | -2.4146 | -1.6043 |
| **CIMD -Situational Vulnerability Q5 (most deprived)** | -2.8229 | 0.2268 | -12.4500 | <.0001 | -3.2674 | -2.3784 |
| **CIMD - Situational Vulnerability Q1 (least deprived) (REF)** | 0.0000 | . | . | . | . | . |
| **Social Assistance (any in past 5 years)** | -7.0625 | 0.1848 | -38.2100 | <.0001 | -7.4247 | -6.7002 |
| **Social Assistance (none in past 5 years) (REF)** | 0.0000 | . | . | . | . | . |
| **Program of Study - French Immersion** | 4.4893 | 0.1329 | 33.7800 | <.0001 | 4.2288 | 4.7498 |
| **Program of Study -Other** | 3.6540 | 0.8853 | 4.1300 | <.0001 | 1.9187 | 5.3892 |
| **Program of Study - French** | 3.1178 | 0.6050 | 5.1500 | <.0001 | 1.9320 | 4.3035 |
| **Program of Study - English (REF)** | 0.0000 | . | . | . | . | . |
| **Household composition – Adults (age 22+) – No adults in household** | -2.5892 | 0.5299 | -4.8900 | <.0001 | -3.6279 | -1.5506 |
| **Household composition – Adults (age 22+) – One adult in household** | -3.0227 | 0.1389 | -21.7700 | <.0001 | -3.2948 | -2.7505 |
| **Household composition – Adults (age 22+) – More than one adult in household (REF)** | 0.0000 | . | . | . | . | . |
| **Household composition – Children (age 21 or under) – Student is only child in household** | -0.4180 | 0.1262 | -3.3100 | 0.0009 | -0.6653 | -0.1707 |
| **Household composition – Children (age 21 or under) – Other children in household (REF)** | 0.0000 | . | . | . | . | . |
| **Recent immigrant** | 3.3538 | 0.3314 | 10.1200 | <.0001 | 2.7042 | 4.0033 |
| **Not a recent immigrant (REF)** | 0.0000 | . | . | . | . | . |

**Supplementary Table S2g. GLM regression estimates – Mean report card score for math for grades 9-12 (AY 2017 – 2020)**

| **Parameter** | **Estimate** | **Standard**  **Error** | **t Value** | **Pr > \|t\|** | **95% Confidence Limits** | |
| --- | --- | --- | --- | --- | --- | --- |
| **Intercept** | 85.2271 | 1.1524 | 73.9600 | <.0001 | 82.9684 | 87.4858 |
| **Treated ADHD** | -5.0414 | 0.2747 | -18.3500 | <.0001 | -5.5799 | -4.5030 |
| **Untreated ADHD** | -5.8444 | 0.1910 | -30.6100 | <.0001 | -6.2186 | -5.4701 |
| **No ADHD (REF)** | 0.0000 | . | . | . | . | . |
| **Age** | -0.7159 | 0.0516 | -13.8800 | <.0001 | -0.8170 | -0.6148 |
| **Male** | -2.9186 | 0.1233 | -23.6700 | <.0001 | -3.1603 | -2.6769 |
| **Female (REF)** | 0.0000 | . | . | . | . | . |
| **Household income quintile Q2** | 1.1123 | 0.2384 | 4.6600 | <.0001 | 0.6450 | 1.5797 |
| **Household income quintile Q3** | 1.5329 | 0.2561 | 5.9900 | <.0001 | 1.0309 | 2.0349 |
| **Household income quintile Q4** | 2.1902 | 0.2752 | 7.9600 | <.0001 | 1.6508 | 2.7295 |
| **Household income quintile Q5 (highest income)** | 3.2502 | 0.2979 | 10.9100 | <.0001 | 2.6663 | 3.8340 |
| **Household income quintile Q1 (lowest income) (REF)** | 0.0000 | . | . | . | . | . |
| **NB Health Zone 2** | 0.6418 | 0.1791 | 3.5800 | 0.0003 | 0.2908 | 0.9928 |
| **NB Health Zone 3** | 0.1610 | 0.1775 | 0.9100 | 0.3644 | -0.1869 | 0.5088 |
| **NB Health Zone 4** | 2.6725 | 0.3089 | 8.6500 | <.0001 | 2.0671 | 3.2780 |
| **NB Health Zone 5** | 0.8380 | 0.3923 | 2.1400 | 0.0327 | 0.0690 | 1.6069 |
| **NB Health Zone 6** | 2.2340 | 0.2766 | 8.0800 | <.0001 | 1.6918 | 2.7762 |
| **NB Health Zone 7** | 1.2618 | 0.2920 | 4.3200 | <.0001 | 0.6894 | 1.8342 |
| **NB Health Zone 1 (REF)** | 0.0000 | . | . | . | . | . |
| **Comorbid conditions - Mood & anxiety disorders (yes)** | -3.3060 | 0.2631 | -12.5700 | <.0001 | -3.8217 | -2.7904 |
| **Comorbid conditions - Mood & anxiety disorders (no) (REF)** | 0.0000 | . | . | . | . | . |
| **Comorbid conditions – One or more of: asthma, diabetes, epilepsy, schizophrenia (yes)** | 0.4854 | 0.7301 | 0.6600 | 0.5061 | -0.9455 | 1.9163 |
| **Comorbid conditions – One or more of: asthma, diabetes, epilepsy, schizophrenia (no) (REF)** | 0.0000 | . | . | . | . | . |
| **Select medications (one or more)** | -1.8143 | 0.2973 | -6.1000 | <.0001 | -2.3970 | -1.2316 |
| **Select medications (none) (REF)** | 0.0000 | . | . | . | . | . |
| **School District - Anglophone** | 1.6562 | 0.6957 | 2.3800 | 0.0173 | 0.2926 | 3.0198 |
| **School District – Francophone (REF)** | 0.0000 | . | . | . | . | . |
| **CIMD - Residential Instability Q2** | 0.4010 | 0.1786 | 2.2500 | 0.0247 | 0.0510 | 0.7510 |
| **CIMD - Residential Instability Q3** | 0.6928 | 0.1931 | 3.5900 | 0.0003 | 0.3144 | 1.0712 |
| **CIMD - Residential Instability Q4** | 1.2272 | 0.2253 | 5.4500 | <.0001 | 0.7856 | 1.6687 |
| **CIMD – Residential Instability Q5 (most deprived)** | 0.9840 | 0.3128 | 3.1500 | 0.0017 | 0.3710 | 1.5969 |
| **CIMD - Residential Instability Q1 (least deprived) (REF)** | 0.0000 | . | . | . | . | . |
| **CIMD - Economic Dependency Q2** | -0.0902 | 0.1397 | -0.6500 | 0.5184 | -0.3640 | 0.1836 |
| **CIMD - Economic Dependency Q3** | 0.1072 | 0.2019 | 0.5300 | 0.5955 | -0.2885 | 0.5028 |
| **CIMD - Economic Dependency Q4** | 1.4425 | 0.2995 | 4.8200 | <.0001 | 0.8554 | 2.0295 |
| **CIMD - Economic Dependency Q5 (most deprived)** | 0.4804 | 0.4780 | 1.0100 | 0.3149 | -0.4565 | 1.4174 |
| **CIMD - Economic Dependency Q1 (least deprived) (REF)** | 0.0000 | . | . | . | . | . |
| **CIMD - Ethnocultural Composition Q2** | -0.4310 | 0.2249 | -1.9200 | 0.0554 | -0.8719 | 0.0099 |
| **CIMD - Ethnocultural Composition Q3** | 0.0343 | 0.2257 | 0.1500 | 0.8793 | -0.4082 | 0.4767 |
| **CIMD - Ethnocultural Composition Q4** | 0.1188 | 0.2356 | 0.5000 | 0.6141 | -0.3430 | 0.5806 |
| **CIMD - Ethnocultural Composition Q5 (most deprived)** | 0.9201 | 0.2399 | 3.8300 | 0.0001 | 0.4498 | 1.3904 |
| **CIMD - Ethnocultural Composition Q1 (least deprived) (REF)** | 0.0000 | . | . | . | . | . |
| **CIMD -Situational Vulnerability Q2** | -1.5509 | 0.2123 | -7.3000 | <.0001 | -1.9670 | -1.1347 |
| **CIMD - Situational Vulnerability Q3** | -1.7326 | 0.2404 | -7.2100 | <.0001 | -2.2037 | -1.2615 |
| **CIMD -Situational Vulnerability Q4** | -1.9539 | 0.2356 | -8.2900 | <.0001 | -2.4156 | -1.4922 |
| **CIMD -Situational Vulnerability Q5 (most deprived)** | -2.7025 | 0.2592 | -10.4300 | <.0001 | -3.2104 | -2.1945 |
| **CIMD - Situational Vulnerability Q1 (least deprived) (REF)** | 0.0000 | . | . | . | . | . |
| **Social Assistance (any in past 5 years)** | -6.6486 | 0.2170 | -30.6300 | <.0001 | -7.0740 | -6.2232 |
| **Social Assistance (none in past 5 years) (REF)** | 0.0000 | . | . | . | . | . |
| **Program of Study - French Immersion** | 4.3050 | 0.1499 | 28.7200 | <.0001 | 4.0112 | 4.5987 |
| **Program of Study -Other** | 8.1634 | 1.1292 | 7.2300 | <.0001 | 5.9502 | 10.3767 |
| **Program of Study - French** | 3.6731 | 0.6917 | 5.3100 | <.0001 | 2.3175 | 5.0288 |
| **Program of Study - English (REF)** | 0.0000 | . | . | . | . | . |
| **Household composition – Adults (age 22+) – No adults in household** | -2.1965 | 0.6951 | -3.1600 | 0.0016 | -3.5588 | -0.8342 |
| **Household composition – Adults (age 22+) – One adult in household** | -3.0304 | 0.1600 | -18.9400 | <.0001 | -3.3440 | -2.7167 |
| **Household composition – Adults (age 22+) – More than one adult in household (REF)** | 0.0000 | . | . | . | . | . |
| **Household composition – Children (age 21 or under) – Student is only child in household** | -0.4738 | 0.1454 | -3.2600 | 0.0011 | -0.7589 | -0.1888 |
| **Household composition – Children (age 21 or under) – Other children in household (REF)** | 0.0000 | . | . | . | . | . |
| **Recent immigrant** | 4.5271 | 0.3739 | 12.1100 | <.0001 | 3.7942 | 5.2599 |
| **Not a recent immigrant (REF)** | 0.0000 | . | . | . | . | . |

**Supplementary Table S2h. GLM regression estimates – Mean report card score for language for grades 9-12 (AY 2017 – 2020)**

| **Parameter** | **Estimate** | **Standard**  **Error** | **t Value** | **Pr > \|t\|** | **95% Confidence Limits** | |
| --- | --- | --- | --- | --- | --- | --- |
| **Intercept** | 83.5587 | 0.8532 | 97.9400 | <.0001 | 81.8864 | 85.2309 |
| **Treated ADHD** | -5.1978 | 0.2005 | -25.9200 | <.0001 | -5.5908 | -4.8048 |
| **Untreated ADHD** | -5.6286 | 0.1375 | -40.9500 | <.0001 | -5.8980 | -5.3592 |
| **No ADHD (REF)** | 0.0000 | . | . | . | . | . |
| **Age** | -0.1771 | 0.0375 | -4.7200 | <.0001 | -0.2506 | -0.1035 |
| **Male** | -5.4893 | 0.0916 | -59.9500 | <.0001 | -5.6687 | -5.3098 |
| **Female (REF)** | 0.0000 | . | . | . | . | . |
| **Household income quintile Q2** | 1.1582 | 0.1739 | 6.6600 | <.0001 | 0.8174 | 1.4990 |
| **Household income quintile Q3** | 1.0646 | 0.1883 | 5.6500 | <.0001 | 0.6956 | 1.4336 |
| **Household income quintile Q4** | 1.5904 | 0.2024 | 7.8600 | <.0001 | 1.1938 | 1.9871 |
| **Household income quintile Q5 (highest income)** | 2.4176 | 0.2200 | 10.9900 | <.0001 | 1.9865 | 2.8487 |
| **Household income quintile Q1 (lowest income) (REF)** | 0.0000 | . | . | . | . | . |
| **NB Health Zone 2** | 2.3178 | 0.1328 | 17.4600 | <.0001 | 2.0576 | 2.5780 |
| **NB Health Zone 3** | 3.1531 | 0.1316 | 23.9500 | <.0001 | 2.8951 | 3.4111 |
| **NB Health Zone 4** | 4.2286 | 0.2268 | 18.6400 | <.0001 | 3.7840 | 4.6731 |
| **NB Health Zone 5** | 1.4695 | 0.2905 | 5.0600 | <.0001 | 0.9002 | 2.0388 |
| **NB Health Zone 6** | 3.1597 | 0.2053 | 15.3900 | <.0001 | 2.7573 | 3.5621 |
| **NB Health Zone 7** | 1.9035 | 0.2156 | 8.8300 | <.0001 | 1.4808 | 2.3261 |
| **NB Health Zone 1 (REF)** | 0.0000 | . | . | . | . | . |
| **Comorbid conditions - Mood & anxiety disorders (yes)** | -2.5527 | 0.1909 | -13.3700 | <.0001 | -2.9268 | -2.1786 |
| **Comorbid conditions - Mood & anxiety disorders (no) (REF)** | 0.0000 | . | . | . | . | . |
| **Comorbid conditions – One or more of: asthma, diabetes, epilepsy, schizophrenia (yes)** | 0.7027 | 0.5345 | 1.3100 | 0.1886 | -0.3449 | 1.7502 |
| **Comorbid conditions – One or more of: asthma, diabetes, epilepsy, schizophrenia (no) (REF)** | 0.0000 | . | . | . | . | . |
| **Select medications (one or more)** | -2.0535 | 0.2130 | -9.6400 | <.0001 | -2.4711 | -1.6359 |
| **Select medications (none) (REF)** | 0.0000 | . | . | . | . | . |
| **School District - Anglophone** | -1.5637 | 0.5246 | -2.9800 | 0.0029 | -2.5919 | -0.5356 |
| **School District – Francophone (REF)** | 0.0000 | . | . | . | . | . |
| **CIMD - Residential Instability Q2** | -0.1784 | 0.1332 | -1.3400 | 0.1803 | -0.4394 | 0.0826 |
| **CIMD - Residential Instability Q3** | 0.1310 | 0.1436 | 0.9100 | 0.3619 | -0.1506 | 0.4125 |
| **CIMD - Residential Instability Q4** | 0.1218 | 0.1674 | 0.7300 | 0.4670 | -0.2063 | 0.4499 |
| **CIMD – Residential Instability Q5 (most deprived)** | 0.0874 | 0.2302 | 0.3800 | 0.7043 | -0.3639 | 0.5386 |
| **CIMD - Residential Instability Q1 (least deprived) (REF)** | 0.0000 | . | . | . | . | . |
| **CIMD - Economic Dependency Q2** | -0.1279 | 0.1036 | -1.2300 | 0.2170 | -0.3309 | 0.0751 |
| **CIMD - Economic Dependency Q3** | 0.4683 | 0.1504 | 3.1100 | 0.0018 | 0.1735 | 0.7632 |
| **CIMD - Economic Dependency Q4** | 1.8556 | 0.2239 | 8.2900 | <.0001 | 1.4168 | 2.2945 |
| **CIMD - Economic Dependency Q5 (most deprived)** | 2.1166 | 0.3544 | 5.9700 | <.0001 | 1.4220 | 2.8113 |
| **CIMD - Economic Dependency Q1 (least deprived) (REF)** | 0.0000 | . | . | . | . | . |
| **CIMD - Ethnocultural Composition Q2** | -0.3554 | 0.1687 | -2.1100 | 0.0352 | -0.6861 | -0.0247 |
| **CIMD - Ethnocultural Composition Q3** | 0.0048 | 0.1691 | 0.0300 | 0.9774 | -0.3266 | 0.3362 |
| **CIMD - Ethnocultural Composition Q4** | -0.3995 | 0.1759 | -2.2700 | 0.0232 | -0.7443 | -0.0547 |
| **CIMD - Ethnocultural Composition Q5 (most deprived)** | 0.3203 | 0.1793 | 1.7900 | 0.0740 | -0.0310 | 0.6717 |
| **CIMD - Ethnocultural Composition Q1 (least deprived) (REF)** | 0.0000 | . | . | . | . | . |
| **CIMD -Situational Vulnerability Q2** | -1.1447 | 0.1592 | -7.1900 | <.0001 | -1.4567 | -0.8326 |
| **CIMD - Situational Vulnerability Q3** | -1.7644 | 0.1798 | -9.8100 | <.0001 | -2.1168 | -1.4119 |
| **CIMD -Situational Vulnerability Q4** | -2.4063 | 0.1756 | -13.7100 | <.0001 | -2.7504 | -2.0622 |
| **CIMD -Situational Vulnerability Q5 (most deprived)** | -3.6428 | 0.1924 | -18.9300 | <.0001 | -4.0200 | -3.2657 |
| **CIMD - Situational Vulnerability Q1 (least deprived) (REF)** | 0.0000 | . | . | . | . | . |
| **Social Assistance (any in past 5 years)** | -6.5121 | 0.1556 | -41.8500 | <.0001 | -6.8171 | -6.2071 |
| **Social Assistance (none in past 5 years) (REF)** | 0.0000 | . | . | . | . | . |
| **Program of Study - French Immersion** | 4.3323 | 0.1121 | 38.6300 | <.0001 | 4.1125 | 4.5521 |
| **Program of Study -Other** | 2.7496 | 0.7751 | 3.5500 | 0.0004 | 1.2304 | 4.2688 |
| **Program of Study - French** | 1.6249 | 0.5215 | 3.1200 | 0.0018 | 0.6027 | 2.6471 |
| **Program of Study - English (REF)** | 0.0000 | . | . | . | . | . |
| **Household composition – Adults (age 22+) – No adults in household** | -3.3145 | 0.4379 | -7.5700 | <.0001 | -4.1728 | -2.4561 |
| **Household composition – Adults (age 22+) – One adult in household** | -2.6371 | 0.1177 | -22.4000 | <.0001 | -2.8678 | -2.4064 |
| **Household composition – Adults (age 22+) – More than one adult in household (REF)** | 0.0000 | . | . | . | . | . |
| **Household composition – Children (age 21 or under) – Student is only child in household** | -0.2620 | 0.1069 | -2.4500 | 0.0142 | -0.4714 | -0.0525 |
| **Household composition – Children (age 21 or under) – Other children in household (REF)** | 0.0000 | . | . | . | . | . |
| **Recent immigrant** | 3.0189 | 0.2872 | 10.5100 | <.0001 | 2.4560 | 3.5818 |
| **Not a recent immigrant (REF)** | 0.0000 | . | . | . | . | . |
