# Supplementary material for: Academic Outcomes in Primary and Secondary School Students Prescribed Long-Acting Stimulants for ADHD Management
Source: J Atten Disord. 2025 Oct 7;30(4):493–505. doi: 10.1177/10870547251378169 (PMC12953683; doi:10.1177/10870547251378169)
Supplement: sj-docx-3-jad-10.1177_10870547251378169 – Supplemental material for Academic Outcomes in Primary and Secondary School Students Prescribed Long-Acting Stimulants for ADHD Management [file sj-docx-3-jad-10.1177_10870547251378169.docx]

**Supplementary Table S3a. GLM regression estimates – Mean overall report card score for grades K-8 (AY 2017 – 2020) (Untreated group as reference)**

| **Parameter** | **Estimate** | **Standard**  **Error** | **t Value** | **Pr > \|t\|** | **95% Confidence Limits** | |
| --- | --- | --- | --- | --- | --- | --- |
| **Intercept** | 2.8808 | 0.0266 | 108.1600 | <.0001 | 2.8286 | 2.9330 |
| **Treated ADHD** | -0.0226 | 0.0041 | -5.5800 | 0.0934 | -0.0305 | 0.0147 |
| **Untreated ADHD (REF)** | 0.0000 | . | . | . | . | . |
| **Age** | 0.0034 | 0.0009 | 3.9400 | <.0001 | 0.0017 | 0.0051 |
| **Male** | -0.0302 | 0.0042 | -7.2300 | <.0001 | -0.0384 | -0.0220 |
| **Female (REF)** | 0.0000 | . | . | . | . | . |
| **Household income quintile Q2** | 0.0040 | 0.0069 | 0.5800 | 0.5599 | -0.0095 | 0.0175 |
| **Household income quintile Q3** | 0.0060 | 0.0079 | 0.7700 | 0.4437 | -0.0094 | 0.0215 |
| **Household income quintile Q4** | 0.0101 | 0.0084 | 1.2000 | 0.2318 | -0.0065 | 0.0267 |
| **Household income quintile Q5 (highest income)** | 0.0171 | 0.0093 | 1.8400 | 0.0652 | -0.0011 | 0.0353 |
| **Household income quintile Q1 (lowest income) (REF)** | 0.0000 | . | . | . | . | . |
| **NB Health Zone 2** | -0.0174 | 0.0055 | -3.1900 | 0.0014 | -0.0281 | -0.0067 |
| **NB Health Zone 3** | 0.1046 | 0.0054 | 19.2400 | <.0001 | 0.0939 | 0.1152 |
| **NB Health Zone 4** | 0.0463 | 0.0129 | 3.6100 | 0.0003 | 0.0212 | 0.0715 |
| **NB Health Zone 5** | 0.0492 | 0.0132 | 3.7200 | 0.0002 | 0.0232 | 0.0751 |
| **NB Health Zone 6** | 0.0355 | 0.0096 | 3.7000 | 0.0002 | 0.0167 | 0.0543 |
| **NB Health Zone 7** | 0.0268 | 0.0102 | 2.6200 | 0.0088 | 0.0068 | 0.0468 |
| **NB Health Zone 1 (REF)** | 0.0000 | . | . | . | . | . |
| **Comorbid conditions - Mood & anxiety disorders (yes)** | 0.0050 | 0.0096 | 0.5100 | 0.6068 | -0.0139 | 0.0239 |
| **Comorbid conditions - Mood & anxiety disorders (no) (REF)** | 0.0000 | . | . | . | . | . |
| **Comorbid conditions – One or more of: asthma, diabetes, epilepsy, schizophrenia (yes)** | 0.0233 | 0.0173 | 1.3500 | 0.1778 | -0.0106 | 0.0573 |
| **Comorbid conditions – One or more of: asthma, diabetes, epilepsy, schizophrenia (no) (REF)** | 0.0000 | . | . | . | . | . |
| **Select medications (one or more)** | -0.0403 | 0.0060 | -6.6700 | <.0001 | -0.0521 | -0.0284 |
| **Select medications (none) (REF)** | 0.0000 | . | . | . | . | . |
| **School District - Anglophone** | -0.1221 | 0.0217 | -5.6400 | <.0001 | -0.1646 | -0.0796 |
| **School District – Francophone (REF)** | 0.0000 | . | . | . | . | . |
| **CIMD - Residential Instability Q2** | -0.0049 | 0.0061 | -0.8200 | 0.4146 | -0.0168 | 0.0069 |
| **CIMD - Residential Instability Q3** | -0.0142 | 0.0064 | -2.2200 | 0.0265 | -0.0268 | -0.0017 |
| **CIMD - Residential Instability Q4** | -0.0283 | 0.0071 | -3.9700 | <.0001 | -0.0422 | -0.0143 |
| **CIMD – Residential Instability Q5 (most deprived)** | -0.0151 | 0.0089 | -1.6900 | 0.0902 | -0.0325 | 0.0024 |
| **CIMD - Residential Instability Q1 (least deprived) (REF)** | 0.0000 | . | . | . | . | . |
| **CIMD - Economic Dependency Q2** | 0.0061 | 0.0072 | 0.8500 | 0.3961 | -0.0080 | 0.0203 |
| **CIMD - Economic Dependency Q3** | -0.0007 | 0.0073 | -0.0900 | 0.9271 | -0.0149 | 0.0135 |
| **CIMD - Economic Dependency Q4** | -0.0022 | 0.0074 | -0.2900 | 0.7682 | -0.0167 | 0.0123 |
| **CIMD - Economic Dependency Q5 (most deprived)** | -0.0049 | 0.0077 | -0.6400 | 0.5218 | -0.0201 | 0.0102 |
| **CIMD - Economic Dependency Q1 (least deprived) (REF)** | 0.0000 | . | . | . | . | . |
| **CIMD - Ethnocultural Composition Q2** | -0.0002 | 0.0045 | -0.0400 | 0.9677 | -0.0091 | 0.0087 |
| **CIMD - Ethnocultural Composition Q3** | -0.0091 | 0.0064 | -1.4300 | 0.1525 | -0.0216 | 0.0034 |
| **CIMD - Ethnocultural Composition Q4** | 0.0021 | 0.0088 | 0.2300 | 0.8160 | -0.0152 | 0.0194 |
| **CIMD - Ethnocultural Composition Q5 (most deprived)** | -0.0441 | 0.0122 | -3.6000 | 0.0003 | -0.0680 | -0.0201 |
| **CIMD - Ethnocultural Composition Q1 (least deprived) (REF)** | 0.0000 | . | . | . | . | . |
| **CIMD -Situational Vulnerability Q2** | -0.0217 | 0.0070 | -3.1000 | 0.0019 | -0.0355 | -0.0080 |
| **CIMD - Situational Vulnerability Q3** | -0.0380 | 0.0079 | -4.8300 | <.0001 | -0.0535 | -0.0226 |
| **CIMD -Situational Vulnerability Q4** | -0.0570 | 0.0077 | -7.4300 | <.0001 | -0.0720 | -0.0419 |
| **CIMD -Situational Vulnerability Q5 (most deprived)** | -0.0739 | 0.0083 | -8.8900 | <.0001 | -0.0902 | -0.0576 |
| **CIMD - Situational Vulnerability Q1 (least deprived) (REF)** | 0.0000 | . | . | . | . | . |
| **Social Assistance (any in past 5 years)** | -0.0978 | 0.0048 | -20.4500 | <.0001 | -0.1072 | -0.0884 |
| **Social Assistance (none in past 5 years) (REF)** | 0.0000 | . | . | . | . | . |
| **Program of Study - French Immersion/Other** | 0.1043 | 0.0067 | 15.6400 | <.0001 | 0.0912 | 0.1174 |
| **Program of Study - French** | 0.0316 | 0.0216 | 1.4600 | 0.1434 | -0.0107 | 0.0740 |
| **Program of Study - English (REF)** | 0.0000 | . | . | . | . | . |
| **Household composition – Adults (age 22+) – No adults in household** | -0.0259 | 0.0203 | -1.2700 | 0.2027 | -0.0657 | 0.0139 |
| **Household composition – Adults (age 22+) – One adult in household** | -0.0338 | 0.0043 | -7.8600 | <.0001 | -0.0422 | -0.0253 |
| **Household composition – Adults (age 22+) – More than one adult in household (REF)** | 0.0000 | . | . | . | . | . |
| **Household composition – Children (age 21 or under) – Student is only child in household** | -0.0128 | 0.0048 | -2.6600 | 0.0079 | -0.0222 | -0.0034 |
| **Household composition – Children (age 21 or under) – Other children in household (REF)** | 0.0000 | . | . | . | . | . |
| **Recent immigrant** | -0.0159 | 0.0245 | -0.6500 | 0.5176 | -0.0640 | 0.0322 |
| **Not a recent immigrant (REF)** | 0.0000 | . | . | . | . | . |

**Supplementary Table S3b. GLM regression estimates – Mean report card score for STEM subjects for grades K-8 (AY 2017 – 2020) (Untreated group as reference)**

| **Parameter** | **Estimate** | **Standard**  **Error** | **t Value** | **Pr > \|t\|** | **95% Confidence Limits** | |
| --- | --- | --- | --- | --- | --- | --- |
| **Intercept** | 2.8696 | 0.0370 | 77.5000 | <.0001 | 2.7970 | 2.9421 |
| **Treated ADHD** | -0.0238 | 0.0056 | -4.2600 | 0.0857 | -0.0347 | 0.0128 |
| **Untreated ADHD (REF)** | 0.0000 | . | . | . | . | . |
| **Age** | -0.0015 | 0.0012 | -1.2600 | 0.2063 | -0.0038 | 0.0008 |
| **Male** | 0.0397 | 0.0058 | 6.9000 | <.0001 | 0.0284 | 0.0510 |
| **Female (REF)** | 0.0000 | . | . | . | . | . |
| **Household income quintile Q2** | -0.0001 | 0.0095 | -0.0100 | 0.9938 | -0.0187 | 0.0186 |
| **Household income quintile Q3** | -0.0001 | 0.0109 | -0.0100 | 0.9938 | -0.0214 | 0.0212 |
| **Household income quintile Q4** | -0.0073 | 0.0117 | -0.6300 | 0.5284 | -0.0302 | 0.0155 |
| **Household income quintile Q5 (highest income)** | 0.0025 | 0.0128 | 0.1900 | 0.8470 | -0.0226 | 0.0276 |
| **Household income quintile Q1 (lowest income) (REF)** | 0.0000 | . | . | . | . | . |
| **NB Health Zone 2** | 0.0050 | 0.0075 | 0.6600 | 0.5102 | -0.0098 | 0.0197 |
| **NB Health Zone 3** | 0.1349 | 0.0075 | 18.0100 | <.0001 | 0.1202 | 0.1496 |
| **NB Health Zone 4** | 0.0474 | 0.0178 | 2.6600 | 0.0077 | 0.0125 | 0.0823 |
| **NB Health Zone 5** | 0.0800 | 0.0182 | 4.4000 | <.0001 | 0.0444 | 0.1157 |
| **NB Health Zone 6** | 0.0218 | 0.0133 | 1.6400 | 0.1009 | -0.0042 | 0.0478 |
| **NB Health Zone 7** | 0.0658 | 0.0141 | 4.6800 | <.0001 | 0.0382 | 0.0934 |
| **NB Health Zone 1 (REF)** | 0.0000 | . | . | . | . | . |
| **Comorbid conditions - Mood & anxiety disorders (yes)** | 0.0029 | 0.0133 | 0.2200 | 0.8265 | -0.0232 | 0.0291 |
| **Comorbid conditions - Mood & anxiety disorders (no) (REF)** | 0.0000 | . | . | . | . | . |
| **Comorbid conditions – One or more of: asthma, diabetes, epilepsy, schizophrenia (yes)** | 0.0028 | 0.0239 | 0.1200 | 0.9081 | -0.0442 | 0.0497 |
| **Comorbid conditions – One or more of: asthma, diabetes, epilepsy, schizophrenia (no) (REF)** | 0.0000 | . | . | . | . | . |
| **Select medications (one or more)** | -0.0308 | 0.0084 | -3.6700 | 0.0002 | -0.0472 | -0.0143 |
| **Select medications (none) (REF)** | 0.0000 | . | . | . | . | . |
| **School District - Anglophone** | -0.1090 | 0.0302 | -3.6000 | 0.0003 | -0.1682 | -0.0497 |
| **School District – Francophone (REF)** | 0.0000 | . | . | . | . | . |
| **CIMD - Residential Instability Q2** | -0.0054 | 0.0083 | -0.6500 | 0.5158 | -0.0218 | 0.0109 |
| **CIMD - Residential Instability Q3** | -0.0173 | 0.0088 | -1.9600 | 0.0500 | -0.0346 | 0.0000 |
| **CIMD - Residential Instability Q4** | -0.0360 | 0.0098 | -3.6800 | 0.0002 | -0.0553 | -0.0168 |
| **CIMD – Residential Instability Q5 (most deprived)** | -0.0234 | 0.0123 | -1.9100 | 0.0564 | -0.0474 | 0.0006 |
| **CIMD - Residential Instability Q1 (least deprived) (REF)** | 0.0000 | . | . | . | . | . |
| **CIMD - Economic Dependency Q2** | 0.0131 | 0.0099 | 1.3200 | 0.1873 | -0.0064 | 0.0326 |
| **CIMD - Economic Dependency Q3** | 0.0046 | 0.0100 | 0.4600 | 0.6481 | -0.0150 | 0.0242 |
| **CIMD - Economic Dependency Q4** | -0.0056 | 0.0102 | -0.5400 | 0.5859 | -0.0256 | 0.0144 |
| **CIMD - Economic Dependency Q5 (most deprived)** | -0.0065 | 0.0106 | -0.6100 | 0.5427 | -0.0273 | 0.0144 |
| **CIMD - Economic Dependency Q1 (least deprived) (REF)** | 0.0000 | . | . | . | . | . |
| **CIMD - Ethnocultural Composition Q2** | 0.0020 | 0.0062 | 0.3200 | 0.7483 | -0.0102 | 0.0142 |
| **CIMD - Ethnocultural Composition Q3** | -0.0128 | 0.0088 | -1.4500 | 0.1465 | -0.0301 | 0.0045 |
| **CIMD - Ethnocultural Composition Q4** | -0.0145 | 0.0122 | -1.1900 | 0.2331 | -0.0384 | 0.0093 |
| **CIMD - Ethnocultural Composition Q5 (most deprived)** | -0.0609 | 0.0169 | -3.6000 | 0.0003 | -0.0941 | -0.0278 |
| **CIMD - Ethnocultural Composition Q1 (least deprived) (REF)** | 0.0000 | . | . | . | . | . |
| **CIMD -Situational Vulnerability Q2** | -0.0279 | 0.0097 | -2.8900 | 0.0039 | -0.0468 | -0.0090 |
| **CIMD - Situational Vulnerability Q3** | -0.0463 | 0.0109 | -4.2600 | <.0001 | -0.0676 | -0.0250 |
| **CIMD -Situational Vulnerability Q4** | -0.0695 | 0.0106 | -6.5700 | <.0001 | -0.0902 | -0.0488 |
| **CIMD -Situational Vulnerability Q5 (most deprived)** | -0.0910 | 0.0115 | -7.9400 | <.0001 | -0.1135 | -0.0686 |
| **CIMD - Situational Vulnerability Q1 (least deprived) (REF)** | 0.0000 | . | . | . | . | . |
| **Social Assistance (any in past 5 years)** | -0.1248 | 0.0066 | -18.9000 | <.0001 | -0.1377 | -0.1118 |
| **Social Assistance (none in past 5 years) (REF)** | 0.0000 | . | . | . | . | . |
| **Program of Study - French Immersion/Other** | 0.0998 | 0.0092 | 10.9000 | <.0001 | 0.0818 | 0.1177 |
| **Program of Study - French** | -0.0296 | 0.0301 | -0.9800 | 0.3258 | -0.0887 | 0.0295 |
| **Program of Study - English (REF)** | 0.0000 | . | . | . | . | . |
| **Household composition – Adults (age 22+) – No adults in household** | -0.0164 | 0.0281 | -0.5800 | 0.5597 | -0.0714 | 0.0387 |
| **Household composition – Adults (age 22+) – One adult in household** | -0.0395 | 0.0059 | -6.6600 | <.0001 | -0.0511 | -0.0278 |
| **Household composition – Adults (age 22+) – More than one adult in household (REF)** | 0.0000 | . | . | . | . | . |
| **Household composition – Children (age 21 or under) – Student is only child in household** | -0.0110 | 0.0067 | -1.6500 | 0.0988 | -0.0240 | 0.0021 |
| **Household composition – Children (age 21 or under) – Other children in household (REF)** | 0.0000 | . | . | . | . | . |
| **Recent immigrant** | 0.0404 | 0.0344 | 1.1800 | 0.2399 | -0.0270 | 0.1079 |
| **Not a recent immigrant (REF)** | 0.0000 | . | . | . | . | . |

**Supplementary Table S3c. GLM regression estimates – Mean report card score for math for grades K-8 (AY 2017 – 2020) (Untreated group as reference)**

| **Parameter** | **Estimate** | **Standard**  **Error** | **t Value** | **Pr > \|t\|** | **95% Confidence Limits** | |
| --- | --- | --- | --- | --- | --- | --- |
| **Intercept** | 2.8853 | 0.0444 | 64.9300 | <.0001 | 2.7982 | 2.9724 |
| **Treated ADHD** | -0.0232 | 0.0066 | -3.5000 | 0.0650 | -0.0363 | 0.0102 |
| **Untreated ADHD (REF)** | 0.0000 | . | . | . | . | . |
| **Age** | -0.0110 | 0.0014 | -7.7900 | <.0001 | -0.0138 | -0.0082 |
| **Male** | 0.0717 | 0.0069 | 10.4500 | <.0001 | 0.0582 | 0.0851 |
| **Female (REF)** | 0.0000 | . | . | . | . | . |
| **Household income quintile Q2** | -0.0026 | 0.0113 | -0.2300 | 0.8207 | -0.0248 | 0.0197 |
| **Household income quintile Q3** | 0.0036 | 0.0130 | 0.2800 | 0.7810 | -0.0218 | 0.0290 |
| **Household income quintile Q4** | -0.0090 | 0.0139 | -0.6500 | 0.5154 | -0.0362 | 0.0182 |
| **Household income quintile Q5 (highest income)** | -0.0036 | 0.0153 | -0.2400 | 0.8116 | -0.0336 | 0.0263 |
| **Household income quintile Q1 (lowest income) (REF)** | 0.0000 | . | . | . | . | . |
| **NB Health Zone 2** | 0.0373 | 0.0090 | 4.1600 | <.0001 | 0.0197 | 0.0548 |
| **NB Health Zone 3** | 0.1842 | 0.0089 | 20.6500 | <.0001 | 0.1667 | 0.2017 |
| **NB Health Zone 4** | 0.0490 | 0.0214 | 2.2900 | 0.0220 | 0.0071 | 0.0910 |
| **NB Health Zone 5** | 0.1225 | 0.0216 | 5.6600 | <.0001 | 0.0801 | 0.1649 |
| **NB Health Zone 6** | 0.0570 | 0.0159 | 3.5900 | 0.0003 | 0.0259 | 0.0881 |
| **NB Health Zone 7** | 0.1020 | 0.0167 | 6.1000 | <.0001 | 0.0693 | 0.1348 |
| **NB Health Zone 1 (REF)** | 0.0000 | . | . | . | . | . |
| **Comorbid conditions - Mood & anxiety disorders (yes)** | 0.0060 | 0.0159 | 0.3800 | 0.7052 | -0.0252 | 0.0372 |
| **Comorbid conditions - Mood & anxiety disorders (no) (REF)** | 0.0000 | . | . | . | . | . |
| **Comorbid conditions – One or more of: asthma, diabetes, epilepsy, schizophrenia (yes)** | 0.0141 | 0.0284 | 0.5000 | 0.6197 | -0.0416 | 0.0698 |
| **Comorbid conditions – One or more of: asthma, diabetes, epilepsy, schizophrenia (no) (REF)** | 0.0000 | . | . | . | . | . |
| **Select medications (one or more)** | -0.0201 | 0.0100 | -2.0000 | 0.0450 | -0.0398 | -0.0004 |
| **Select medications (none) (REF)** | 0.0000 | . | . | . | . | . |
| **School District - Anglophone** | -0.1147 | 0.0364 | -3.1500 | 0.0016 | -0.1860 | -0.0433 |
| **School District – Francophone (REF)** | 0.0000 | . | . | . | . | . |
| **CIMD - Residential Instability Q2** | -0.0194 | 0.0099 | -1.9600 | 0.0504 | -0.0389 | 0.0000 |
| **CIMD - Residential Instability Q3** | -0.0304 | 0.0105 | -2.8900 | 0.0038 | -0.0511 | -0.0098 |
| **CIMD - Residential Instability Q4** | -0.0481 | 0.0117 | -4.1200 | <.0001 | -0.0710 | -0.0253 |
| **CIMD – Residential Instability Q5 (most deprived)** | -0.0420 | 0.0146 | -2.8700 | 0.0041 | -0.0706 | -0.0133 |
| **CIMD - Residential Instability Q1 (least deprived) (REF)** | 0.0000 | . | . | . | . | . |
| **CIMD - Economic Dependency Q2** | 0.0144 | 0.0118 | 1.2200 | 0.2226 | -0.0088 | 0.0376 |
| **CIMD - Economic Dependency Q3** | 0.0008 | 0.0119 | 0.0700 | 0.9440 | -0.0225 | 0.0242 |
| **CIMD - Economic Dependency Q4** | -0.0026 | 0.0121 | -0.2100 | 0.8328 | -0.0264 | 0.0212 |
| **CIMD - Economic Dependency Q5 (most deprived)** | -0.0051 | 0.0127 | -0.4100 | 0.6851 | -0.0299 | 0.0197 |
| **CIMD - Economic Dependency Q1 (least deprived) (REF)** | 0.0000 | . | . | . | . | . |
| **CIMD - Ethnocultural Composition Q2** | 0.0050 | 0.0074 | 0.6700 | 0.5031 | -0.0096 | 0.0195 |
| **CIMD - Ethnocultural Composition Q3** | -0.0089 | 0.0105 | -0.8500 | 0.3961 | -0.0295 | 0.0117 |
| **CIMD - Ethnocultural Composition Q4** | -0.0141 | 0.0145 | -0.9700 | 0.3310 | -0.0425 | 0.0143 |
| **CIMD - Ethnocultural Composition Q5 (most deprived)** | -0.0504 | 0.0202 | -2.5000 | 0.0125 | -0.0899 | -0.0108 |
| **CIMD - Ethnocultural Composition Q1 (least deprived) (REF)** | 0.0000 | . | . | . | . | . |
| **CIMD -Situational Vulnerability Q2** | -0.0323 | 0.0115 | -2.8100 | 0.0049 | -0.0548 | -0.0098 |
| **CIMD - Situational Vulnerability Q3** | -0.0533 | 0.0129 | -4.1200 | <.0001 | -0.0786 | -0.0279 |
| **CIMD -Situational Vulnerability Q4** | -0.0767 | 0.0126 | -6.0900 | <.0001 | -0.1013 | -0.0520 |
| **CIMD -Situational Vulnerability Q5 (most deprived)** | -0.1042 | 0.0137 | -7.6200 | <.0001 | -0.1309 | -0.0774 |
| **CIMD - Situational Vulnerability Q1 (least deprived) (REF)** | 0.0000 | . | . | . | . | . |
| **Social Assistance (any in past 5 years)** | -0.1351 | 0.0079 | -17.1600 | <.0001 | -0.1506 | -0.1197 |
| **Social Assistance (none in past 5 years) (REF)** | 0.0000 | . | . | . | . | . |
| **Program of Study - French Immersion/Other** | 0.1151 | 0.0109 | 10.5800 | <.0001 | 0.0938 | 0.1364 |
| **Program of Study - French** | -0.0116 | 0.0363 | -0.3200 | 0.7492 | -0.0827 | 0.0595 |
| **Program of Study - English (REF)** | 0.0000 | . | . | . | . | . |
| **Household composition – Adults (age 22+) – No adults in household** | 0.0070 | 0.0334 | 0.2100 | 0.8342 | -0.0585 | 0.0725 |
| **Household composition – Adults (age 22+) – One adult in household** | -0.0409 | 0.0071 | -5.7900 | <.0001 | -0.0548 | -0.0271 |
| **Household composition – Adults (age 22+) – More than one adult in household (REF)** | 0.0000 | . | . | . | . | . |
| **Household composition – Children (age 21 or under) – Student is only child in household** | -0.0174 | 0.0079 | -2.2000 | 0.0282 | -0.0329 | -0.0019 |
| **Household composition – Children (age 21 or under) – Other children in household (REF)** | 0.0000 | . | . | . | . | . |
| **Recent immigrant** | 0.0884 | 0.0415 | 2.1300 | 0.0332 | 0.0071 | 0.1696 |
| **Not a recent immigrant (REF)** | 0.0000 | . | . | . | . | . |

**Supplementary Table S3d. GLM regression estimates – Mean report card score for language for grades K-8 (AY 2017 – 2020) (Untreated group as reference)**

| **Parameter** | **Estimate** | **Standard**  **Error** | **t Value** | **Pr > \|t\|** | **95% Confidence Limits** | |
| --- | --- | --- | --- | --- | --- | --- |
| **Intercept** | 2.4040 | 0.0403 | 59.6400 | <.0001 | 2.3250 | 2.4830 |
| **Treated ADHD** | -0.0432 | 0.0061 | -7.1000 | 0.0984 | -0.0551 | 0.0313 |
| **Untreated ADHD (REF)** | 0.0000 | . | . | . | . | . |
| **Age** | 0.0328 | 0.0013 | 25.4200 | <.0001 | 0.0303 | 0.0354 |
| **Male** | -0.1000 | 0.0063 | -15.9300 | <.0001 | -0.1123 | -0.0877 |
| **Female (REF)** | 0.0000 | . | . | . | . | . |
| **Household income quintile Q2** | 0.0034 | 0.0104 | 0.3300 | 0.7441 | -0.0169 | 0.0237 |
| **Household income quintile Q3** | 0.0055 | 0.0118 | 0.4600 | 0.6442 | -0.0177 | 0.0287 |
| **Household income quintile Q4** | 0.0179 | 0.0127 | 1.4100 | 0.1594 | -0.0070 | 0.0427 |
| **Household income quintile Q5 (highest income)** | 0.0280 | 0.0139 | 2.0100 | 0.0445 | 0.0007 | 0.0554 |
| **Household income quintile Q1 (lowest income) (REF)** | 0.0000 | . | . | . | . | . |
| **NB Health Zone 2** | -0.0029 | 0.0082 | -0.3500 | 0.7252 | -0.0189 | 0.0132 |
| **NB Health Zone 3** | 0.1534 | 0.0082 | 18.8000 | <.0001 | 0.1374 | 0.1694 |
| **NB Health Zone 4** | 0.0238 | 0.0195 | 1.2200 | 0.2217 | -0.0144 | 0.0620 |
| **NB Health Zone 5** | 0.0736 | 0.0199 | 3.7000 | 0.0002 | 0.0347 | 0.1125 |
| **NB Health Zone 6** | 0.0445 | 0.0145 | 3.0700 | 0.0021 | 0.0161 | 0.0729 |
| **NB Health Zone 7** | 0.0727 | 0.0153 | 4.7400 | <.0001 | 0.0427 | 0.1028 |
| **NB Health Zone 1 (REF)** | 0.0000 | . | . | . | . | . |
| **Comorbid conditions - Mood & anxiety disorders (yes)** | 0.0027 | 0.0145 | 0.1800 | 0.8539 | -0.0258 | 0.0311 |
| **Comorbid conditions - Mood & anxiety disorders (no) (REF)** | 0.0000 | . | . | . | . | . |
| **Comorbid conditions – One or more of: asthma, diabetes, epilepsy, schizophrenia (yes)** | 0.0532 | 0.0260 | 2.0400 | 0.0413 | 0.0021 | 0.1042 |
| **Comorbid conditions – One or more of: asthma, diabetes, epilepsy, schizophrenia (no) (REF)** | 0.0000 | . | . | . | . | . |
| **Select medications (one or more)** | -0.0302 | 0.0092 | -3.2900 | 0.0010 | -0.0481 | -0.0122 |
| **Select medications (none) (REF)** | 0.0000 | . | . | . | . | . |
| **School District - Anglophone** | -0.0784 | 0.0329 | -2.3800 | 0.0172 | -0.1429 | -0.0139 |
| **School District – Francophone (REF)** | 0.0000 | . | . | . | . | . |
| **CIMD - Residential Instability Q2** | -0.0186 | 0.0091 | -2.0500 | 0.0405 | -0.0364 | -0.0008 |
| **CIMD - Residential Instability Q3** | -0.0089 | 0.0096 | -0.9200 | 0.3558 | -0.0278 | 0.0100 |
| **CIMD - Residential Instability Q4** | -0.0238 | 0.0107 | -2.2300 | 0.0256 | -0.0448 | -0.0029 |
| **CIMD – Residential Instability Q5 (most deprived)** | -0.0203 | 0.0134 | -1.5200 | 0.1294 | -0.0465 | 0.0059 |
| **CIMD - Residential Instability Q1 (least deprived) (REF)** | 0.0000 | . | . | . | . | . |
| **CIMD - Economic Dependency Q2** | 0.0052 | 0.0108 | 0.4800 | 0.6282 | -0.0160 | 0.0265 |
| **CIMD - Economic Dependency Q3** | 0.0044 | 0.0109 | 0.4100 | 0.6848 | -0.0169 | 0.0257 |
| **CIMD - Economic Dependency Q4** | 0.0079 | 0.0111 | 0.7100 | 0.4760 | -0.0139 | 0.0297 |
| **CIMD - Economic Dependency Q5 (most deprived)** | -0.0028 | 0.0116 | -0.2400 | 0.8078 | -0.0255 | 0.0199 |
| **CIMD - Economic Dependency Q1 (least deprived) (REF)** | 0.0000 | . | . | . | . | . |
| **CIMD - Ethnocultural Composition Q2** | 0.0089 | 0.0068 | 1.3100 | 0.1918 | -0.0045 | 0.0222 |
| **CIMD - Ethnocultural Composition Q3** | -0.0035 | 0.0096 | -0.3700 | 0.7133 | -0.0224 | 0.0153 |
| **CIMD - Ethnocultural Composition Q4** | 0.0019 | 0.0133 | 0.1400 | 0.8854 | -0.0241 | 0.0279 |
| **CIMD - Ethnocultural Composition Q5 (most deprived)** | -0.0524 | 0.0185 | -2.8400 | 0.0046 | -0.0887 | -0.0162 |
| **CIMD - Ethnocultural Composition Q1 (least deprived) (REF)** | 0.0000 | . | . | . | . | . |
| **CIMD -Situational Vulnerability Q2** | -0.0438 | 0.0105 | -4.1700 | <.0001 | -0.0644 | -0.0232 |
| **CIMD - Situational Vulnerability Q3** | -0.0706 | 0.0118 | -5.9800 | <.0001 | -0.0938 | -0.0475 |
| **CIMD -Situational Vulnerability Q4** | -0.0845 | 0.0115 | -7.3400 | <.0001 | -0.1071 | -0.0619 |
| **CIMD -Situational Vulnerability Q5 (most deprived)** | -0.1112 | 0.0125 | -8.9100 | <.0001 | -0.1357 | -0.0867 |
| **CIMD - Situational Vulnerability Q1 (least deprived) (REF)** | 0.0000 | . | . | . | . | . |
| **Social Assistance (any in past 5 years)** | -0.1446 | 0.0072 | -20.0900 | <.0001 | -0.1587 | -0.1305 |
| **Social Assistance (none in past 5 years) (REF)** | 0.0000 | . | . | . | . | . |
| **Program of Study - French Immersion/Other** | 0.1203 | 0.0100 | 12.0700 | <.0001 | 0.1007 | 0.1398 |
| **Program of Study - French** | 0.1185 | 0.0328 | 3.6100 | 0.0003 | 0.0542 | 0.1827 |
| **Program of Study - English (REF)** | 0.0000 | . | . | . | . | . |
| **Household composition – Adults (age 22+) – No adults in household** | -0.0297 | 0.0305 | -0.9700 | 0.3304 | -0.0894 | 0.0301 |
| **Household composition – Adults (age 22+) – One adult in household** | -0.0491 | 0.0065 | -7.5900 | <.0001 | -0.0617 | -0.0364 |
| **Household composition – Adults (age 22+) – More than one adult in household (REF)** | 0.0000 | . | . | . | . | . |
| **Household composition – Children (age 21 or under) – Student is only child in household** | -0.0002 | 0.0072 | -0.0200 | 0.9830 | -0.0144 | 0.0140 |
| **Household composition – Children (age 21 or under) – Other children in household (REF)** | 0.0000 | . | . | . | . | . |
| **Recent immigrant** | -0.0166 | 0.0379 | -0.4400 | 0.6609 | -0.0910 | 0.0577 |
| **Not a recent immigrant (REF)** | 0.0000 | . | . | . | . | . |
