# Supplementary material for: Academic Outcomes in Primary and Secondary School Students Prescribed Long-Acting Stimulants for ADHD Management
Source: J Atten Disord. 2025 Oct 7;30(4):493–505. doi: 10.1177/10870547251378169 (PMC12953683; doi:10.1177/10870547251378169)
Supplement: sj-docx-4-jad-10.1177_10870547251378169 – Supplemental material for Academic Outcomes in Primary and Secondary School Students Prescribed Long-Acting Stimulants for ADHD Management [file sj-docx-4-jad-10.1177_10870547251378169.docx]

**Supplementary Table S4a. GLM regression estimates – Proportion of overall report card scores meeting minimum acceptable standard (score of 3 or higher) for grades K-8 (AY 2017 – 2020)**

| **Parameter** | **Estimate** | **Standard**  **Error** | **t**  **Value** | **Pr > \|t\|** | **95% Confidence Limits** | |
| --- | --- | --- | --- | --- | --- | --- |
| **Intercept** | 0.9971 | 0.0064 | 155.1400 | <.0001 | 0.9845 | 1.0097 |
| **Treated ADHD** | -0.1098 | 0.0020 | -55.7100 | <.0001 | -0.1136 | -0.1059 |
| **Untreated ADHD** | -0.0966 | 0.0015 | -62.7700 | <.0001 | -0.0996 | -0.0936 |
| **No ADHD (REF)** | 0.0000 | . | . | . | . | . |
| **Age** | -0.0028 | 0.0002 | -14.9000 | <.0001 | -0.0032 | -0.0024 |
| **Male** | -0.0277 | 0.0009 | -30.8200 | <.0001 | -0.0294 | -0.0259 |
| **Female (REF)** | 0.0000 | . | . | . | . | . |
| **Household income quintile Q2** | 0.0053 | 0.0017 | 3.1000 | 0.0019 | 0.0020 | 0.0087 |
| **Household income quintile Q3** | 0.0126 | 0.0019 | 6.7000 | <.0001 | 0.0089 | 0.0163 |
| **Household income quintile Q4** | 0.0154 | 0.0020 | 7.6100 | <.0001 | 0.0114 | 0.0193 |
| **Household income quintile Q5 (highest income)** | 0.0178 | 0.0022 | 8.1500 | <.0001 | 0.0135 | 0.0220 |
| **Household income quintile Q1 (lowest income) (REF)** | 0.0000 | . | . | . | . | . |
| **NB Health Zone 2** | -0.0044 | 0.0013 | -3.4200 | 0.0006 | -0.0069 | -0.0019 |
| **NB Health Zone 3** | 0.0306 | 0.0013 | 24.3700 | <.0001 | 0.0281 | 0.0330 |
| **NB Health Zone 4** | 0.0099 | 0.0028 | 3.5900 | 0.0003 | 0.0045 | 0.0153 |
| **NB Health Zone 5** | 0.0070 | 0.0033 | 2.1400 | 0.0324 | 0.0006 | 0.0134 |
| **NB Health Zone 6** | 0.0083 | 0.0023 | 3.6200 | 0.0003 | 0.0038 | 0.0129 |
| **NB Health Zone 7** | 0.0209 | 0.0022 | 9.4000 | <.0001 | 0.0165 | 0.0253 |
| **NB Health Zone 1 (REF)** | 0.0000 | . | . | . | . | . |
| **Comorbid conditions - Mood & anxiety disorders (yes)** | -0.0057 | 0.0036 | -1.6100 | 0.1081 | -0.0127 | 0.0013 |
| **Comorbid conditions - Mood & anxiety disorders (no) (REF)** | 0.0000 | . | . | . | . | . |
| **Comorbid conditions – One or more of: asthma, diabetes, epilepsy, schizophrenia (yes)** | 0.0040 | 0.0044 | 0.9000 | 0.3688 | -0.0047 | 0.0127 |
| **Comorbid conditions – One or more of: asthma, diabetes, epilepsy, schizophrenia (no) (REF)** | 0.0000 | . | . | . | . | . |
| **Select medications (one or more)** | -0.0362 | 0.0032 | -11.4400 | <.0001 | -0.0424 | -0.0300 |
| **Select medications (none) (REF)** | 0.0000 | . | . | . | . | . |
| **School District - Anglophone** | -0.0498 | 0.0056 | -8.9700 | <.0001 | -0.0607 | -0.0389 |
| **School District – Francophone (REF)** | 0.0000 | . | . | . | . | . |
| **CIMD - Residential Instability Q2** | -0.0016 | 0.0013 | -1.1800 | 0.2384 | -0.0042 | 0.0010 |
| **CIMD - Residential Instability Q3** | -0.0038 | 0.0014 | -2.6600 | 0.0079 | -0.0066 | -0.0010 |
| **CIMD - Residential Instability Q4** | -0.0084 | 0.0017 | -5.0900 | <.0001 | -0.0117 | -0.0052 |
| **CIMD – Residential Instability Q5 (most deprived)** | -0.0017 | 0.0022 | -0.8000 | 0.4251 | -0.0060 | 0.0025 |
| **CIMD - Residential Instability Q1 (least deprived) (REF)** | 0.0000 | . | . | . | . | . |
| **CIMD - Economic Dependency Q2** | -0.0022 | 0.0016 | -1.3700 | 0.1722 | -0.0054 | 0.0010 |
| **CIMD - Economic Dependency Q3** | -0.0041 | 0.0016 | -2.5100 | 0.0121 | -0.0073 | -0.0009 |
| **CIMD - Economic Dependency Q4** | -0.0039 | 0.0017 | -2.2800 | 0.0228 | -0.0072 | -0.0005 |
| **CIMD - Economic Dependency Q5 (most deprived)** | -0.0015 | 0.0018 | -0.8400 | 0.3983 | -0.0049 | 0.0020 |
| **CIMD - Economic Dependency Q1 (least deprived) (REF)** | 0.0000 | . | . | . | . | . |
| **CIMD - Ethnocultural Composition Q2** | 0.0001 | 0.0010 | 0.0500 | 0.9592 | -0.0020 | 0.0021 |
| **CIMD - Ethnocultural Composition Q3** | -0.0048 | 0.0015 | -3.2300 | 0.0012 | -0.0077 | -0.0019 |
| **CIMD - Ethnocultural Composition Q4** | 0.0004 | 0.0021 | 0.1800 | 0.8547 | -0.0038 | 0.0046 |
| **CIMD - Ethnocultural Composition Q5 (most deprived)** | -0.0274 | 0.0031 | -8.9800 | <.0001 | -0.0334 | -0.0214 |
| **CIMD - Ethnocultural Composition Q1 (least deprived) (REF)** | 0.0000 | . | . | . | . | . |
| **CIMD -Situational Vulnerability Q2** | -0.0118 | 0.0016 | -7.6100 | <.0001 | -0.0149 | -0.0088 |
| **CIMD - Situational Vulnerability Q3** | -0.0171 | 0.0018 | -9.7000 | <.0001 | -0.0205 | -0.0136 |
| **CIMD -Situational Vulnerability Q4** | -0.0265 | 0.0017 | -15.4100 | <.0001 | -0.0299 | -0.0232 |
| **CIMD -Situational Vulnerability Q5 (most deprived)** | -0.0405 | 0.0019 | -21.2100 | <.0001 | -0.0442 | -0.0367 |
| **CIMD - Situational Vulnerability Q1 (least deprived) (REF)** | 0.0000 | . | . | . | . | . |
| **Social Assistance (any in past 5 years)** | -0.0826 | 0.0013 | -62.8300 | <.0001 | -0.0852 | -0.0800 |
| **Social Assistance (none in past 5 years) (REF)** | 0.0000 | . | . | . | . | . |
| **Program of Study - French Immersion/Other** | 0.0437 | 0.0015 | 29.1300 | <.0001 | 0.0408 | 0.0466 |
| **Program of Study - French** | -0.0008 | 0.0056 | -0.1400 | 0.8918 | -0.0116 | 0.0101 |
| **Program of Study - English (REF)** | 0.0000 | . | . | . | . | . |
| **Household composition – Adults (age 22+) – No adults in household** | -0.0505 | 0.0062 | -8.1100 | <.0001 | -0.0627 | -0.0383 |
| **Household composition – Adults (age 22+) – One adult in household** | -0.0286 | 0.0011 | -26.3900 | <.0001 | -0.0307 | -0.0265 |
| **Household composition – Adults (age 22+) – More than one adult in household (REF)** | 0.0000 | . | . | . | . | . |
| **Household composition – Children (age 21 or under) – Student is only child in household** | -0.0007 | 0.0012 | -0.6200 | 0.5378 | -0.0031 | 0.0016 |
| **Household composition – Children (age 21 or under) – Other children in household (REF)** | 0.0000 | . | . | . | . | . |
| **Recent immigrant** | -0.0034 | 0.0024 | -1.4600 | 0.1457 | -0.0081 | 0.0012 |
| **Not a recent immigrant (REF)** | 0.0000 | . | . | . | . | . |

**Supplementary Table S4b. GLM regression estimates – Proportion of report card scores for STEM subjects meeting minimum acceptable standard (score of 3 or higher) for grades K-8 (AY 2017 – 2020)**

| **Parameter** | **Estimate** | **Standard**  **Error** | **t Value** | **Pr > \|t\|** | **95% Confidence Limits** | |
| --- | --- | --- | --- | --- | --- | --- |
| **Intercept** | 1.0089 | 0.0097 | 104.5300 | <.0001 | 0.9900 | 1.0278 |
| **Treated ADHD** | -0.1356 | 0.0029 | -46.3700 | <.0001 | -0.1413 | -0.1298 |
| **Untreated ADHD** | -0.1209 | 0.0023 | -52.8700 | <.0001 | -0.1254 | -0.1164 |
| **No ADHD (REF)** | 0.0000 | . | . | . | . | . |
| **Age** | -0.0046 | 0.0003 | -16.4700 | <.0001 | -0.0052 | -0.0041 |
| **Male** | 0.0023 | 0.0013 | 1.7600 | 0.0784 | -0.0003 | 0.0049 |
| **Female (REF)** | 0.0000 | . | . | . | . | . |
| **Household income quintile Q2** | 0.0060 | 0.0025 | 2.3700 | 0.0177 | 0.0010 | 0.0110 |
| **Household income quintile Q3** | 0.0142 | 0.0028 | 5.0700 | <.0001 | 0.0087 | 0.0196 |
| **Household income quintile Q4** | 0.0159 | 0.0030 | 5.3200 | <.0001 | 0.0101 | 0.0218 |
| **Household income quintile Q5 (highest income)** | 0.0192 | 0.0032 | 5.9300 | <.0001 | 0.0128 | 0.0255 |
| **Household income quintile Q1 (lowest income) (REF)** | 0.0000 | . | . | . | . | . |
| **NB Health Zone 2** | 0.0130 | 0.0019 | 6.8700 | <.0001 | 0.0093 | 0.0167 |
| **NB Health Zone 3** | 0.0525 | 0.0019 | 28.2400 | <.0001 | 0.0489 | 0.0562 |
| **NB Health Zone 4** | 0.0151 | 0.0041 | 3.6900 | 0.0002 | 0.0071 | 0.0231 |
| **NB Health Zone 5** | 0.0207 | 0.0049 | 4.2600 | <.0001 | 0.0111 | 0.0302 |
| **NB Health Zone 6** | 0.0098 | 0.0034 | 2.8700 | 0.0041 | 0.0031 | 0.0165 |
| **NB Health Zone 7** | 0.0454 | 0.0033 | 13.7800 | <.0001 | 0.0390 | 0.0519 |
| **NB Health Zone 1 (REF)** | 0.0000 | . | . | . | . | . |
| **Comorbid conditions - Mood & anxiety disorders (yes)** | -0.0087 | 0.0053 | -1.6300 | 0.1021 | -0.0190 | 0.0017 |
| **Comorbid conditions - Mood & anxiety disorders (no) (REF)** | 0.0000 | . | . | . | . | . |
| **Comorbid conditions – One or more of: asthma, diabetes, epilepsy, schizophrenia (yes)** | 0.0055 | 0.0066 | 0.8300 | 0.4071 | -0.0075 | 0.0184 |
| **Comorbid conditions – One or more of: asthma, diabetes, epilepsy, schizophrenia (no) (REF)** | 0.0000 | . | . | . | . | . |
| **Select medications (one or more)** | -0.0252 | 0.0048 | -5.2800 | <.0001 | -0.0346 | -0.0159 |
| **Select medications (none) (REF)** | 0.0000 | . | . | . | . | . |
| **School District - Anglophone** | -0.0779 | 0.0084 | -9.2900 | <.0001 | -0.0943 | -0.0615 |
| **School District – Francophone (REF)** | 0.0000 | . | . | . | . | . |
| **CIMD - Residential Instability Q2** | -0.0044 | 0.0020 | -2.2400 | 0.0249 | -0.0083 | -0.0006 |
| **CIMD - Residential Instability Q3** | -0.0060 | 0.0021 | -2.8400 | 0.0045 | -0.0102 | -0.0019 |
| **CIMD - Residential Instability Q4** | -0.0117 | 0.0025 | -4.7700 | <.0001 | -0.0165 | -0.0069 |
| **CIMD – Residential Instability Q5 (most deprived)** | -0.0040 | 0.0032 | -1.2300 | 0.2170 | -0.0103 | 0.0023 |
| **CIMD - Residential Instability Q1 (least deprived) (REF)** | 0.0000 | . | . | . | . | . |
| **CIMD - Economic Dependency Q2** | -0.0015 | 0.0024 | -0.6400 | 0.5243 | -0.0063 | 0.0032 |
| **CIMD - Economic Dependency Q3** | -0.0059 | 0.0024 | -2.4500 | 0.0143 | -0.0107 | -0.0012 |
| **CIMD - Economic Dependency Q4** | -0.0057 | 0.0025 | -2.2700 | 0.0234 | -0.0106 | -0.0008 |
| **CIMD - Economic Dependency Q5 (most deprived)** | -0.0054 | 0.0026 | -2.0700 | 0.0383 | -0.0105 | -0.0003 |
| **CIMD - Economic Dependency Q1 (least deprived) (REF)** | 0.0000 | . | . | . | . | . |
| **CIMD - Ethnocultural Composition Q2** | -0.0007 | 0.0016 | -0.4700 | 0.6379 | -0.0038 | 0.0023 |
| **CIMD - Ethnocultural Composition Q3** | -0.0063 | 0.0022 | -2.8700 | 0.0041 | -0.0106 | -0.0020 |
| **CIMD - Ethnocultural Composition Q4** | -0.0045 | 0.0032 | -1.4300 | 0.1535 | -0.0108 | 0.0017 |
| **CIMD - Ethnocultural Composition Q5 (most deprived)** | -0.0472 | 0.0046 | -10.3600 | <.0001 | -0.0562 | -0.0383 |
| **CIMD - Ethnocultural Composition Q1 (least deprived) (REF)** | 0.0000 | . | . | . | . | . |
| **CIMD -Situational Vulnerability Q2** | -0.0154 | 0.0023 | -6.6700 | <.0001 | -0.0199 | -0.0109 |
| **CIMD - Situational Vulnerability Q3** | -0.0220 | 0.0026 | -8.4200 | <.0001 | -0.0271 | -0.0169 |
| **CIMD -Situational Vulnerability Q4** | -0.0330 | 0.0026 | -12.9500 | <.0001 | -0.0381 | -0.0281 |
| **CIMD -Situational Vulnerability Q5 (most deprived)** | -0.0493 | 0.0028 | -17.4200 | <.0001 | -0.0548 | -0.0437 |
| **CIMD - Situational Vulnerability Q1 (least deprived) (REF)** | 0.0000 | . | . | . | . | . |
| **Social Assistance (any in past 5 years)** | -0.1136 | 0.0020 | -58.2000 | <.0001 | -0.1174 | -0.1098 |
| **Social Assistance (none in past 5 years) (REF)** | 0.0000 | . | . | . | . | . |
| **Program of Study - French Immersion/Other** | 0.0435 | 0.0022 | 19.6200 | <.0001 | 0.0392 | 0.0479 |
| **Program of Study - French** | -0.0254 | 0.0084 | -3.0300 | 0.0025 | -0.0418 | -0.0090 |
| **Program of Study - English (REF)** | 0.0000 | . | . | . | . | . |
| **Household composition – Adults (age 22+) – No adults in household** | -0.0476 | 0.0092 | -5.1500 | <.0001 | -0.0657 | -0.0295 |
| **Household composition – Adults (age 22+) – One adult in household** | -0.0357 | 0.0016 | -22.2400 | <.0001 | -0.0389 | -0.0326 |
| **Household composition – Adults (age 22+) – More than one adult in household (REF)** | 0.0000 | . | . | . | . | . |
| **Household composition – Children (age 21 or under) – Student is only child in household** | 0.0003 | 0.0018 | 0.1800 | 0.8596 | -0.0032 | 0.0038 |
| **Household composition – Children (age 21 or under) – Other children in household (REF)** | 0.0000 | . | . | . | . | . |
| **Recent immigrant** | -0.0103 | 0.0035 | -2.9200 | 0.0035 | -0.0172 | -0.0034 |
| **Not a recent immigrant (REF)** | 0.0000 | . | . | . | . | . |

**Supplementary Table S4c. GLM regression estimates – Proportion of report card scores for math meeting minimum acceptable standard (score of 3 or higher) for grades K-8 (AY 2017 – 2020)**

| **Parameter** | **Estimate** | **Standard**  **Error** | **t Value** | **Pr > \|t\|** | **95% Confidence Limits** | |
| --- | --- | --- | --- | --- | --- | --- |
| **Intercept** | 1.0122 | 0.0120 | 84.3800 | <.0001 | 0.9887 | 1.0357 |
| **Treated ADHD** | -0.1558 | 0.0036 | -42.9600 | <.0001 | -0.1629 | -0.1487 |
| **Untreated ADHD** | -0.1381 | 0.0028 | -48.6700 | <.0001 | -0.1437 | -0.1325 |
| **No ADHD (REF)** | 0.0000 | . | . | . | . | . |
| **Age** | -0.0101 | 0.0004 | -29.0100 | <.0001 | -0.0108 | -0.0094 |
| **Male** | 0.0146 | 0.0017 | 8.8800 | <.0001 | 0.0114 | 0.0178 |
| **Female (REF)** | 0.0000 | . | . | . | . | . |
| **Household income quintile Q2** | 0.0093 | 0.0031 | 2.9600 | 0.0030 | 0.0032 | 0.0154 |
| **Household income quintile Q3** | 0.0177 | 0.0035 | 5.1400 | <.0001 | 0.0110 | 0.0245 |
| **Household income quintile Q4** | 0.0209 | 0.0037 | 5.6600 | <.0001 | 0.0137 | 0.0282 |
| **Household income quintile Q5 (highest income)** | 0.0243 | 0.0040 | 6.0700 | <.0001 | 0.0164 | 0.0321 |
| **Household income quintile Q1 (lowest income) (REF)** | 0.0000 | . | . | . | . | . |
| **NB Health Zone 2** | 0.0271 | 0.0023 | 11.6100 | <.0001 | 0.0226 | 0.0317 |
| **NB Health Zone 3** | 0.0716 | 0.0023 | 31.1000 | <.0001 | 0.0671 | 0.0761 |
| **NB Health Zone 4** | 0.0175 | 0.0051 | 3.4400 | 0.0006 | 0.0075 | 0.0274 |
| **NB Health Zone 5** | 0.0408 | 0.0060 | 6.8000 | <.0001 | 0.0291 | 0.0526 |
| **NB Health Zone 6** | 0.0245 | 0.0042 | 5.7700 | <.0001 | 0.0161 | 0.0328 |
| **NB Health Zone 7** | 0.0624 | 0.0041 | 15.3000 | <.0001 | 0.0544 | 0.0704 |
| **NB Health Zone 1 (REF)** | 0.0000 | . | . | . | . | . |
| **Comorbid conditions - Mood & anxiety disorders (yes)** | -0.0133 | 0.0066 | -2.0300 | 0.0425 | -0.0262 | -0.0005 |
| **Comorbid conditions - Mood & anxiety disorders (no) (REF)** | 0.0000 | . | . | . | . | . |
| **Comorbid conditions – One or more of: asthma, diabetes, epilepsy, schizophrenia (yes)** | 0.0045 | 0.0082 | 0.5500 | 0.5841 | -0.0116 | 0.0205 |
| **Comorbid conditions – One or more of: asthma, diabetes, epilepsy, schizophrenia (no) (REF)** | 0.0000 | . | . | . | . | . |
| **Select medications (one or more)** | -0.0197 | 0.0060 | -3.3000 | 0.0010 | -0.0313 | -0.0080 |
| **Select medications (none) (REF)** | 0.0000 | . | . | . | . | . |
| **School District - Anglophone** | -0.0663 | 0.0104 | -6.3500 | <.0001 | -0.0867 | -0.0458 |
| **School District – Francophone (REF)** | 0.0000 | . | . | . | . | . |
| **CIMD - Residential Instability Q2** | -0.0059 | 0.0024 | -2.4200 | 0.0156 | -0.0107 | -0.0011 |
| **CIMD - Residential Instability Q3** | -0.0076 | 0.0026 | -2.9000 | 0.0037 | -0.0128 | -0.0025 |
| **CIMD - Residential Instability Q4** | -0.0133 | 0.0030 | -4.3900 | <.0001 | -0.0193 | -0.0074 |
| **CIMD – Residential Instability Q5 (most deprived)** | -0.0080 | 0.0040 | -2.0200 | 0.0434 | -0.0158 | -0.0002 |
| **CIMD - Residential Instability Q1 (least deprived) (REF)** | 0.0000 | . | . | . | . | . |
| **CIMD - Economic Dependency Q2** | -0.0013 | 0.0030 | -0.4500 | 0.6519 | -0.0072 | 0.0045 |
| **CIMD - Economic Dependency Q3** | -0.0051 | 0.0030 | -1.7000 | 0.0883 | -0.0110 | 0.0008 |
| **CIMD - Economic Dependency Q4** | -0.0033 | 0.0031 | -1.0800 | 0.2815 | -0.0094 | 0.0027 |
| **CIMD - Economic Dependency Q5 (most deprived)** | -0.0031 | 0.0032 | -0.9700 | 0.3329 | -0.0094 | 0.0032 |
| **CIMD - Economic Dependency Q1 (least deprived) (REF)** | 0.0000 | . | . | . | . | . |
| **CIMD - Ethnocultural Composition Q2** | -0.0010 | 0.0019 | -0.5100 | 0.6089 | -0.0047 | 0.0028 |
| **CIMD - Ethnocultural Composition Q3** | -0.0067 | 0.0027 | -2.4700 | 0.0134 | -0.0120 | -0.0014 |
| **CIMD - Ethnocultural Composition Q4** | -0.0085 | 0.0039 | -2.1600 | 0.0305 | -0.0162 | -0.0008 |
| **CIMD - Ethnocultural Composition Q5 (most deprived)** | -0.0438 | 0.0057 | -7.7300 | <.0001 | -0.0549 | -0.0327 |
| **CIMD - Ethnocultural Composition Q1 (least deprived) (REF)** | 0.0000 | . | . | . | . | . |
| **CIMD -Situational Vulnerability Q2** | -0.0197 | 0.0029 | -6.9100 | <.0001 | -0.0253 | -0.0141 |
| **CIMD - Situational Vulnerability Q3** | -0.0264 | 0.0032 | -8.1800 | <.0001 | -0.0327 | -0.0201 |
| **CIMD -Situational Vulnerability Q4** | -0.0401 | 0.0032 | -12.6900 | <.0001 | -0.0463 | -0.0339 |
| **CIMD -Situational Vulnerability Q5 (most deprived)** | -0.0594 | 0.0035 | -16.9600 | <.0001 | -0.0663 | -0.0525 |
| **CIMD - Situational Vulnerability Q1 (least deprived) (REF)** | 0.0000 | . | . | . | . | . |
| **Social Assistance (any in past 5 years)** | -0.1264 | 0.0024 | -52.2300 | <.0001 | -0.1311 | -0.1216 |
| **Social Assistance (none in past 5 years) (REF)** | 0.0000 | . | . | . | . | . |
| **Program of Study - French Immersion/Other** | 0.0450 | 0.0027 | 16.3900 | <.0001 | 0.0396 | 0.0503 |
| **Program of Study - French** | -0.0267 | 0.0104 | -2.5600 | 0.0104 | -0.0471 | -0.0063 |
| **Program of Study - English (REF)** | 0.0000 | . | . | . | . | . |
| **Household composition – Adults (age 22+) – No adults in household** | -0.0347 | 0.0115 | -3.0300 | 0.0024 | -0.0572 | -0.0123 |
| **Household composition – Adults (age 22+) – One adult in household** | -0.0400 | 0.0020 | -20.1300 | <.0001 | -0.0439 | -0.0361 |
| **Household composition – Adults (age 22+) – More than one adult in household (REF)** | 0.0000 | . | . | . | . | . |
| **Household composition – Children (age 21 or under) – Student is only child in household** | -0.0028 | 0.0022 | -1.2500 | 0.2119 | -0.0071 | 0.0016 |
| **Household composition – Children (age 21 or under) – Other children in household (REF)** | 0.0000 | . | . | . | . | . |
| **Recent immigrant** | -0.0084 | 0.0044 | -1.9100 | 0.0560 | -0.0170 | 0.0002 |
| **Not a recent immigrant (REF)** | 0.0000 | . | . | . | . | . |

**Supplementary Table S4d. GLM regression estimates – Proportion of report card scores for language meeting minimum acceptable standard (score of 3 or higher) for grades K-8 (AY 2017 – 2020)**

| **Parameter** | **Estimate** | **Standard**  **Error** | **t Value** | **Pr > \|t\|** | **95% Confidence Limits** | |
| --- | --- | --- | --- | --- | --- | --- |
| **Intercept** | 0.8353 | 0.0115 | 72.3600 | <.0001 | 0.8127 | 0.8579 |
| **Treated ADHD** | -0.1872 | 0.0035 | -53.6900 | <.0001 | -0.1940 | -0.1803 |
| **Untreated ADHD** | -0.1557 | 0.0027 | -57.1200 | <.0001 | -0.1611 | -0.1504 |
| **No ADHD (REF)** | 0.0000 | . | . | . | . | . |
| **Age** | 0.0085 | 0.0003 | 25.4600 | <.0001 | 0.0079 | 0.0092 |
| **Male** | -0.0694 | 0.0016 | -43.8100 | <.0001 | -0.0725 | -0.0663 |
| **Female (REF)** | 0.0000 | . | . | . | . | . |
| **Household income quintile Q2** | 0.0034 | 0.0030 | 1.1400 | 0.2544 | -0.0025 | 0.0094 |
| **Household income quintile Q3** | 0.0108 | 0.0033 | 3.2400 | 0.0012 | 0.0043 | 0.0173 |
| **Household income quintile Q4** | 0.0200 | 0.0036 | 5.6200 | <.0001 | 0.0130 | 0.0270 |
| **Household income quintile Q5 (highest income)** | 0.0252 | 0.0039 | 6.5500 | <.0001 | 0.0177 | 0.0327 |
| **Household income quintile Q1 (lowest income) (REF)** | 0.0000 | . | . | . | . | . |
| **NB Health Zone 2** | 0.0001 | 0.0023 | 0.0300 | 0.9753 | -0.0043 | 0.0045 |
| **NB Health Zone 3** | 0.0484 | 0.0022 | 21.8400 | <.0001 | 0.0441 | 0.0528 |
| **NB Health Zone 4** | 0.0186 | 0.0049 | 3.8100 | 0.0001 | 0.0090 | 0.0282 |
| **NB Health Zone 5** | 0.0114 | 0.0058 | 1.9800 | 0.0482 | 0.0001 | 0.0228 |
| **NB Health Zone 6** | 0.0259 | 0.0041 | 6.3500 | <.0001 | 0.0179 | 0.0339 |
| **NB Health Zone 7** | 0.0376 | 0.0039 | 9.5800 | <.0001 | 0.0299 | 0.0453 |
| **NB Health Zone 1 (REF)** | 0.0000 | . | . | . | . | . |
| **Comorbid conditions - Mood & anxiety disorders (yes)** | -0.0036 | 0.0063 | -0.5700 | 0.5681 | -0.0160 | 0.0088 |
| **Comorbid conditions - Mood & anxiety disorders (no) (REF)** | 0.0000 | . | . | . | . | . |
| **Comorbid conditions – One or more of: asthma, diabetes, epilepsy, schizophrenia (yes)** | 0.0078 | 0.0079 | 0.9900 | 0.3209 | -0.0076 | 0.0232 |
| **Comorbid conditions – One or more of: asthma, diabetes, epilepsy, schizophrenia (no) (REF)** | 0.0000 | . | . | . | . | . |
| **Select medications (one or more)** | -0.0281 | 0.0057 | -4.9200 | <.0001 | -0.0393 | -0.0169 |
| **Select medications (none) (REF)** | 0.0000 | . | . | . | . | . |
| **School District - Anglophone** | -0.0514 | 0.0100 | -5.1200 | <.0001 | -0.0711 | -0.0317 |
| **School District – Francophone (REF)** | 0.0000 | . | . | . | . | . |
| **CIMD - Residential Instability Q2** | -0.0017 | 0.0023 | -0.7300 | 0.4677 | -0.0063 | 0.0029 |
| **CIMD - Residential Instability Q3** | 0.0017 | 0.0025 | 0.6700 | 0.5004 | -0.0033 | 0.0067 |
| **CIMD - Residential Instability Q4** | -0.0056 | 0.0029 | -1.9300 | 0.0533 | -0.0114 | 0.0001 |
| **CIMD – Residential Instability Q5 (most deprived)** | -0.0011 | 0.0038 | -0.2900 | 0.7713 | -0.0086 | 0.0064 |
| **CIMD - Residential Instability Q1 (least deprived) (REF)** | 0.0000 | . | . | . | . | . |
| **CIMD - Economic Dependency Q2** | -0.0062 | 0.0029 | -2.1700 | 0.0299 | -0.0119 | -0.0006 |
| **CIMD - Economic Dependency Q3** | -0.0062 | 0.0029 | -2.1500 | 0.0312 | -0.0119 | -0.0006 |
| **CIMD - Economic Dependency Q4** | -0.0082 | 0.0030 | -2.7400 | 0.0062 | -0.0140 | -0.0023 |
| **CIMD - Economic Dependency Q5 (most deprived)** | -0.0072 | 0.0031 | -2.3300 | 0.0197 | -0.0133 | -0.0012 |
| **CIMD - Economic Dependency Q1 (least deprived) (REF)** | 0.0000 | . | . | . | . | . |
| **CIMD - Ethnocultural Composition Q2** | 0.0027 | 0.0018 | 1.4500 | 0.1469 | -0.0009 | 0.0063 |
| **CIMD - Ethnocultural Composition Q3** | -0.0015 | 0.0026 | -0.5600 | 0.5765 | -0.0066 | 0.0037 |
| **CIMD - Ethnocultural Composition Q4** | -0.0026 | 0.0038 | -0.6800 | 0.4971 | -0.0100 | 0.0049 |
| **CIMD - Ethnocultural Composition Q5 (most deprived)** | -0.0536 | 0.0055 | -9.8300 | <.0001 | -0.0643 | -0.0429 |
| **CIMD - Ethnocultural Composition Q1 (least deprived) (REF)** | 0.0000 | . | . | . | . | . |
| **CIMD -Situational Vulnerability Q2** | -0.0190 | 0.0028 | -6.9300 | <.0001 | -0.0244 | -0.0136 |
| **CIMD - Situational Vulnerability Q3** | -0.0273 | 0.0031 | -8.8000 | <.0001 | -0.0334 | -0.0212 |
| **CIMD -Situational Vulnerability Q4** | -0.0424 | 0.0030 | -13.9400 | <.0001 | -0.0483 | -0.0364 |
| **CIMD -Situational Vulnerability Q5 (most deprived)** | -0.0611 | 0.0034 | -18.1200 | <.0001 | -0.0677 | -0.0545 |
| **CIMD - Situational Vulnerability Q1 (least deprived) (REF)** | 0.0000 | . | . | . | . | . |
| **Social Assistance (any in past 5 years)** | -0.1396 | 0.0023 | -59.9600 | <.0001 | -0.1441 | -0.1350 |
| **Social Assistance (none in past 5 years) (REF)** | 0.0000 | . | . | . | . | . |
| **Program of Study - French Immersion/Other** | 0.0544 | 0.0026 | 20.6000 | <.0001 | 0.0492 | 0.0596 |
| **Program of Study - French** | 0.0232 | 0.0100 | 2.3200 | 0.0206 | 0.0036 | 0.0429 |
| **Program of Study - English (REF)** | 0.0000 | . | . | . | . | . |
| **Household composition – Adults (age 22+) – No adults in household** | -0.0785 | 0.0110 | -7.1400 | <.0001 | -0.1001 | -0.0569 |
| **Household composition – Adults (age 22+) – One adult in household** | -0.0446 | 0.0019 | -23.2700 | <.0001 | -0.0483 | -0.0408 |
| **Household composition – Adults (age 22+) – More than one adult in household (REF)** | 0.0000 | . | . | . | . | . |
| **Household composition – Children (age 21 or under) – Student is only child in household** | 0.0080 | 0.0021 | 3.7100 | 0.0002 | 0.0038 | 0.0122 |
| **Household composition – Children (age 21 or under) – Other children in household (REF)** | 0.0000 | . | . | . | . | . |
| **Recent immigrant** | -0.0291 | 0.0042 | -6.8600 | <.0001 | -0.0374 | -0.0208 |
| **Not a recent immigrant (REF)** | 0.0000 | . | . | . | . | . |

**Supplementary Table S4e. GLM regression estimates – Proportion of overall report card scores meeting minimum acceptable standard (score of 3 or higher) for grades K-8 (AY 2017 – 2020) (Untreated group as reference)**

| **Parameter** | **Estimate** | **Standard**  **Error** | **t Value** | **Pr > \|t\|** | **95% Confidence Limits** | |
| --- | --- | --- | --- | --- | --- | --- |
| **Intercept** | 0.9454 | 0.0194 | 48.6900 | <.0001 | 0.9073 | 0.9834 |
| **Treated ADHD** | 0.0137 | 0.0029 | -4.6700 | 0.1475 | -0.0080 | 0.0194 |
| **Untreated ADHD (REF)** | 0.0000 | . | . | . | . | . |
| **Age** | -0.0059 | 0.0006 | -9.5100 | <.0001 | -0.0071 | -0.0047 |
| **Male** | -0.0225 | 0.0030 | -7.4300 | <.0001 | -0.0284 | -0.0166 |
| **Female (REF)** | 0.0000 | . | . | . | . | . |
| **Household income quintile Q2** | -0.0006 | 0.0050 | -0.1100 | 0.9092 | -0.0103 | 0.0092 |
| **Household income quintile Q3** | 0.0081 | 0.0057 | 1.4200 | 0.1548 | -0.0031 | 0.0193 |
| **Household income quintile Q4** | 0.0076 | 0.0061 | 1.2400 | 0.2148 | -0.0044 | 0.0196 |
| **Household income quintile Q5 (highest income)** | 0.0185 | 0.0067 | 2.7500 | 0.0059 | 0.0053 | 0.0317 |
| **Household income quintile Q1 (lowest income) (REF)** | 0.0000 | . | . | . | . | . |
| **NB Health Zone 2** | -0.0222 | 0.0040 | -5.6100 | <.0001 | -0.0299 | -0.0144 |
| **NB Health Zone 3** | 0.0555 | 0.0039 | 14.1100 | <.0001 | 0.0478 | 0.0632 |
| **NB Health Zone 4** | 0.0104 | 0.0093 | 1.1200 | 0.2648 | -0.0079 | 0.0286 |
| **NB Health Zone 5** | 0.0078 | 0.0096 | 0.8100 | 0.4163 | -0.0110 | 0.0265 |
| **NB Health Zone 6** | -0.0030 | 0.0069 | -0.4300 | 0.6672 | -0.0166 | 0.0106 |
| **NB Health Zone 7** | 0.0083 | 0.0074 | 1.1200 | 0.2635 | -0.0062 | 0.0228 |
| **NB Health Zone 1 (REF)** | 0.0000 | . | . | . | . | . |
| **Comorbid conditions - Mood & anxiety disorders (yes)** | 0.0073 | 0.0070 | 1.0400 | 0.2979 | -0.0064 | 0.0209 |
| **Comorbid conditions - Mood & anxiety disorders (no) (REF)** | 0.0000 | . | . | . | . | . |
| **Comorbid conditions – One or more of: asthma, diabetes, epilepsy, schizophrenia (yes)** | 0.0201 | 0.0125 | 1.6000 | 0.1091 | -0.0045 | 0.0446 |
| **Comorbid conditions – One or more of: asthma, diabetes, epilepsy, schizophrenia (no) (REF)** | 0.0000 | . | . | . | . | . |
| **Select medications (one or more)** | -0.0277 | 0.0044 | -6.3500 | <.0001 | -0.0363 | -0.0192 |
| **Select medications (none) (REF)** | 0.0000 | . | . | . | . | . |
| **School District - Anglophone** | -0.0720 | 0.0158 | -4.5400 | <.0001 | -0.1030 | -0.0409 |
| **School District – Francophone (REF)** | 0.0000 | . | . | . | . | . |
| **CIMD - Residential Instability Q2** | -0.0022 | 0.0044 | -0.5100 | 0.6089 | -0.0108 | 0.0064 |
| **CIMD - Residential Instability Q3** | -0.0076 | 0.0046 | -1.6400 | 0.1005 | -0.0167 | 0.0015 |
| **CIMD - Residential Instability Q4** | -0.0119 | 0.0051 | -2.3200 | 0.0203 | -0.0220 | -0.0019 |
| **CIMD – Residential Instability Q5 (most deprived)** | -0.0031 | 0.0064 | -0.4800 | 0.6323 | -0.0157 | 0.0095 |
| **CIMD - Residential Instability Q1 (least deprived) (REF)** | 0.0000 | . | . | . | . | . |
| **CIMD - Economic Dependency Q2** | -0.0005 | 0.0052 | -0.0900 | 0.9278 | -0.0107 | 0.0098 |
| **CIMD - Economic Dependency Q3** | -0.0059 | 0.0052 | -1.1200 | 0.2636 | -0.0161 | 0.0044 |
| **CIMD - Economic Dependency Q4** | -0.0050 | 0.0054 | -0.9400 | 0.3475 | -0.0155 | 0.0055 |
| **CIMD - Economic Dependency Q5 (most deprived)** | -0.0065 | 0.0056 | -1.1600 | 0.2451 | -0.0174 | 0.0045 |
| **CIMD - Economic Dependency Q1 (least deprived) (REF)** | 0.0000 | . | . | . | . | . |
| **CIMD - Ethnocultural Composition Q2** | 0.0004 | 0.0033 | 0.1100 | 0.9107 | -0.0061 | 0.0068 |
| **CIMD - Ethnocultural Composition Q3** | -0.0087 | 0.0046 | -1.8900 | 0.0590 | -0.0178 | 0.0003 |
| **CIMD - Ethnocultural Composition Q4** | 0.0007 | 0.0064 | 0.1100 | 0.9134 | -0.0118 | 0.0132 |
| **CIMD - Ethnocultural Composition Q5 (most deprived)** | -0.0379 | 0.0089 | -4.2800 | <.0001 | -0.0553 | -0.0205 |
| **CIMD - Ethnocultural Composition Q1 (least deprived) (REF)** | 0.0000 | . | . | . | . | . |
| **CIMD -Situational Vulnerability Q2** | -0.0112 | 0.0051 | -2.2100 | 0.0270 | -0.0212 | -0.0013 |
| **CIMD - Situational Vulnerability Q3** | -0.0225 | 0.0057 | -3.9500 | <.0001 | -0.0337 | -0.0113 |
| **CIMD -Situational Vulnerability Q4** | -0.0357 | 0.0056 | -6.4300 | <.0001 | -0.0466 | -0.0248 |
| **CIMD -Situational Vulnerability Q5 (most deprived)** | -0.0446 | 0.0060 | -7.4000 | <.0001 | -0.0563 | -0.0328 |
| **CIMD - Situational Vulnerability Q1 (least deprived) (REF)** | 0.0000 | . | . | . | . | . |
| **Social Assistance (any in past 5 years)** | -0.0636 | 0.0035 | -18.3700 | <.0001 | -0.0703 | -0.0568 |
| **Social Assistance (none in past 5 years) (REF)** | 0.0000 | . | . | . | . | . |
| **Program of Study - French Immersion** | 0.0729 | 0.0048 | 15.0800 | <.0001 | 0.0634 | 0.0823 |
| **Program of Study - Other** | 0.1181 | 0.0744 | 1.5900 | 0.1122 | -0.0276 | 0.2639 |
| **Program of Study - French** | 0.0097 | 0.0158 | 0.6100 | 0.5390 | -0.0213 | 0.0407 |
| **Program of Study - English (REF)** | 0.0000 | . | . | . | . | . |
| **Household composition – Adults (age 22+) – No adults in household** | -0.0312 | 0.0147 | -2.1200 | 0.0338 | -0.0600 | -0.0024 |
| **Household composition – Adults (age 22+) – One adult in household** | -0.0232 | 0.0031 | -7.4700 | <.0001 | -0.0293 | -0.0171 |
| **Household composition – Adults (age 22+) – More than one adult in household (REF)** | 0.0000 | . | . | . | . | . |
| **Household composition – Children (age 21 or under) – Student is only child in household** | -0.0059 | 0.0035 | -1.7100 | 0.0882 | -0.0128 | 0.0009 |
| **Household composition – Children (age 21 or under) – Other children in household (REF)** | 0.0000 | . | . | . | . | . |
| **Recent immigrant** | -0.0223 | 0.0178 | -1.2600 | 0.2094 | -0.0571 | 0.0125 |
| **Not a recent immigrant (REF)** | 0.0000 | . | . | . | . | . |

**Supplementary Table S4f. GLM regression estimates – Proportion of report card scores for STEM subjects meeting minimum acceptable standard (score of 3 or higher) for grades K-8 (AY 2017 – 2020) (Untreated group as reference)**

| **Parameter** | **Estimate** | **Standard**  **Error** | **t Value** | **Pr > \|t\|** | **95% Confidence Limits** | |
| --- | --- | --- | --- | --- | --- | --- |
| **Intercept** | 0.8731 | 0.0292 | 29.9000 | <.0001 | 0.8159 | 0.9304 |
| **Treated ADHD** | 0.0152 | 0.0044 | -3.4700 | 0.2005 | -0.0066 | 0.0238 |
| **Untreated ADHD (REF)** | 0.0000 | . | . | . | . | . |
| **Age** | -0.0041 | 0.0009 | -4.4300 | <.0001 | -0.0059 | -0.0023 |
| **Male** | 0.0301 | 0.0045 | 6.6600 | <.0001 | 0.0212 | 0.0389 |
| **Female (REF)** | 0.0000 | . | . | . | . | . |
| **Household income quintile Q2** | -0.0050 | 0.0075 | -0.6700 | 0.5020 | -0.0196 | 0.0096 |
| **Household income quintile Q3** | 0.0060 | 0.0085 | 0.7000 | 0.4851 | -0.0108 | 0.0227 |
| **Household income quintile Q4** | -0.0103 | 0.0091 | -1.1300 | 0.2598 | -0.0282 | 0.0076 |
| **Household income quintile Q5 (highest income)** | 0.0086 | 0.0100 | 0.8500 | 0.3935 | -0.0111 | 0.0283 |
| **Household income quintile Q1 (lowest income) (REF)** | 0.0000 | . | . | . | . | . |
| **NB Health Zone 2** | -0.0033 | 0.0059 | -0.5600 | 0.5732 | -0.0149 | 0.0082 |
| **NB Health Zone 3** | 0.0797 | 0.0059 | 13.5700 | <.0001 | 0.0682 | 0.0913 |
| **NB Health Zone 4** | 0.0204 | 0.0140 | 1.4600 | 0.1438 | -0.0070 | 0.0478 |
| **NB Health Zone 5** | 0.0188 | 0.0143 | 1.3200 | 0.1883 | -0.0092 | 0.0468 |
| **NB Health Zone 6** | 0.0021 | 0.0104 | 0.2000 | 0.8390 | -0.0183 | 0.0225 |
| **NB Health Zone 7** | 0.0281 | 0.0110 | 2.5500 | 0.0109 | 0.0065 | 0.0498 |
| **NB Health Zone 1 (REF)** | 0.0000 | . | . | . | . | . |
| **Comorbid conditions - Mood & anxiety disorders (yes)** | 0.0045 | 0.0105 | 0.4300 | 0.6705 | -0.0161 | 0.0250 |
| **Comorbid conditions - Mood & anxiety disorders (no) (REF)** | 0.0000 | . | . | . | . | . |
| **Comorbid conditions – One or more of: asthma, diabetes, epilepsy, schizophrenia (yes)** | 0.0314 | 0.0188 | 1.6700 | 0.0942 | -0.0054 | 0.0683 |
| **Comorbid conditions – One or more of: asthma, diabetes, epilepsy, schizophrenia (no) (REF)** | 0.0000 | . | . | . | . | . |
| **Select medications (one or more)** | -0.0170 | 0.0066 | -2.5800 | 0.0100 | -0.0299 | -0.0041 |
| **Select medications (none) (REF)** | 0.0000 | . | . | . | . | . |
| **School District - Anglophone** | -0.0839 | 0.0239 | -3.5100 | 0.0004 | -0.1307 | -0.0371 |
| **School District – Francophone (REF)** | 0.0000 | . | . | . | . | . |
| **CIMD - Residential Instability Q2** | -0.0098 | 0.0065 | -1.4900 | 0.1350 | -0.0226 | 0.0030 |
| **CIMD - Residential Instability Q3** | -0.0169 | 0.0069 | -2.4400 | 0.0146 | -0.0305 | -0.0033 |
| **CIMD - Residential Instability Q4** | -0.0239 | 0.0077 | -3.1000 | 0.0019 | -0.0389 | -0.0088 |
| **CIMD – Residential Instability Q5 (most deprived)** | -0.0197 | 0.0096 | -2.0500 | 0.0408 | -0.0385 | -0.0008 |
| **CIMD - Residential Instability Q1 (least deprived) (REF)** | 0.0000 | . | . | . | . | . |
| **CIMD - Economic Dependency Q2** | 0.0058 | 0.0078 | 0.7500 | 0.4536 | -0.0094 | 0.0211 |
| **CIMD - Economic Dependency Q3** | -0.0027 | 0.0078 | -0.3400 | 0.7332 | -0.0180 | 0.0127 |
| **CIMD - Economic Dependency Q4** | -0.0048 | 0.0080 | -0.5900 | 0.5522 | -0.0205 | 0.0109 |
| **CIMD - Economic Dependency Q5 (most deprived)** | -0.0054 | 0.0083 | -0.6500 | 0.5169 | -0.0217 | 0.0109 |
| **CIMD - Economic Dependency Q1 (least deprived) (REF)** | 0.0000 | . | . | . | . | . |
| **CIMD - Ethnocultural Composition Q2** | 0.0052 | 0.0049 | 1.0600 | 0.2891 | -0.0044 | 0.0148 |
| **CIMD - Ethnocultural Composition Q3** | -0.0112 | 0.0069 | -1.6200 | 0.1056 | -0.0247 | 0.0024 |
| **CIMD - Ethnocultural Composition Q4** | -0.0108 | 0.0095 | -1.1300 | 0.2566 | -0.0295 | 0.0079 |
| **CIMD - Ethnocultural Composition Q5 (most deprived)** | -0.0481 | 0.0133 | -3.6300 | 0.0003 | -0.0741 | -0.0221 |
| **CIMD - Ethnocultural Composition Q1 (least deprived) (REF)** | 0.0000 | . | . | . | . | . |
| **CIMD -Situational Vulnerability Q2** | -0.0165 | 0.0076 | -2.1700 | 0.0297 | -0.0313 | -0.0016 |
| **CIMD - Situational Vulnerability Q3** | -0.0308 | 0.0085 | -3.6100 | 0.0003 | -0.0475 | -0.0141 |
| **CIMD -Situational Vulnerability Q4** | -0.0496 | 0.0083 | -5.9800 | <.0001 | -0.0659 | -0.0334 |
| **CIMD -Situational Vulnerability Q5 (most deprived)** | -0.0595 | 0.0090 | -6.6100 | <.0001 | -0.0771 | -0.0418 |
| **CIMD - Situational Vulnerability Q1 (least deprived) (REF)** | 0.0000 | . | . | . | . | . |
| **Social Assistance (any in past 5 years)** | -0.0827 | 0.0052 | -15.9800 | <.0001 | -0.0929 | -0.0726 |
| **Social Assistance (none in past 5 years) (REF)** | 0.0000 | . | . | . | . | . |
| **Program of Study - French Immersion** | 0.0751 | 0.0072 | 10.4500 | <.0001 | 0.0611 | 0.0892 |
| **Program of Study - Other** | 0.0686 | 0.1345 | 0.5100 | 0.6099 | -0.1951 | 0.3323 |
| **Program of Study - French** | -0.0102 | 0.0238 | -0.4300 | 0.6686 | -0.0569 | 0.0365 |
| **Program of Study - English (REF)** | 0.0000 | . | . | . | . | . |
| **Household composition – Adults (age 22+) – No adults in household** | -0.0086 | 0.0220 | -0.3900 | 0.6969 | -0.0518 | 0.0346 |
| **Household composition – Adults (age 22+) – One adult in household** | -0.0290 | 0.0046 | -6.2500 | <.0001 | -0.0381 | -0.0199 |
| **Household composition – Adults (age 22+) – More than one adult in household (REF)** | 0.0000 | . | . | . | . | . |
| **Household composition – Children (age 21 or under) – Student is only child in household** | -0.0059 | 0.0052 | -1.1300 | 0.2605 | -0.0161 | 0.0044 |
| **Household composition – Children (age 21 or under) – Other children in household (REF)** | 0.0000 | . | . | . | . | . |
| **Recent immigrant** | 0.0095 | 0.0270 | 0.3500 | 0.7250 | -0.0434 | 0.0624 |
| **Not a recent immigrant (REF)** | 0.0000 | . | . | . | . | . |

**Supplementary Table S4g. GLM regression estimates – Proportion of report card scores for math meeting minimum acceptable standard (score of 3 or higher) for grades K-8 (AY 2017 – 2020) (Untreated group as reference)**

| **Parameter** | **Estimate** | **Standard**  **Error** | **t Value** | **Pr > \|t\|** | **95% Confidence Limits** | |
| --- | --- | --- | --- | --- | --- | --- |
| **Intercept** | 0.8444 | 0.0362 | 23.3500 | <.0001 | 0.7735 | 0.9153 |
| **Treated ADHD** | 0.0169 | 0.0054 | -3.1300 | 0.1700 | -0.0063 | 0.0274 |
| **Untreated ADHD (REF)** | 0.0000 | . | . | . | . | . |
| **Age** | -0.0107 | 0.0011 | -9.3700 | <.0001 | -0.0130 | -0.0085 |
| **Male** | 0.0543 | 0.0056 | 9.7600 | <.0001 | 0.0434 | 0.0651 |
| **Female (REF)** | 0.0000 | . | . | . | . | . |
| **Household income quintile Q2** | -0.0065 | 0.0092 | -0.7100 | 0.4768 | -0.0245 | 0.0115 |
| **Household income quintile Q3** | 0.0090 | 0.0105 | 0.8600 | 0.3921 | -0.0116 | 0.0295 |
| **Household income quintile Q4** | -0.0119 | 0.0112 | -1.0600 | 0.2901 | -0.0339 | 0.0101 |
| **Household income quintile Q5 (highest income)** | 0.0094 | 0.0124 | 0.7600 | 0.4474 | -0.0148 | 0.0336 |
| **Household income quintile Q1 (lowest income) (REF)** | 0.0000 | . | . | . | . | . |
| **NB Health Zone 2** | 0.0129 | 0.0073 | 1.7800 | 0.0751 | -0.0013 | 0.0271 |
| **NB Health Zone 3** | 0.1079 | 0.0072 | 14.9300 | <.0001 | 0.0938 | 0.1221 |
| **NB Health Zone 4** | 0.0076 | 0.0174 | 0.4400 | 0.6606 | -0.0264 | 0.0417 |
| **NB Health Zone 5** | 0.0473 | 0.0175 | 2.7000 | 0.0070 | 0.0129 | 0.0816 |
| **NB Health Zone 6** | 0.0251 | 0.0128 | 1.9600 | 0.0506 | -0.0001 | 0.0503 |
| **NB Health Zone 7** | 0.0454 | 0.0135 | 3.3500 | 0.0008 | 0.0188 | 0.0719 |
| **NB Health Zone 1 (REF)** | 0.0000 | . | . | . | . | . |
| **Comorbid conditions - Mood & anxiety disorders (yes)** | 0.0001 | 0.0129 | 0.0100 | 0.9923 | -0.0251 | 0.0254 |
| **Comorbid conditions - Mood & anxiety disorders (no) (REF)** | 0.0000 | . | . | . | . | . |
| **Comorbid conditions – One or more of: asthma, diabetes, epilepsy, schizophrenia (yes)** | 0.0360 | 0.0230 | 1.5700 | 0.1175 | -0.0091 | 0.0812 |
| **Comorbid conditions – One or more of: asthma, diabetes, epilepsy, schizophrenia (no) (REF)** | 0.0000 | . | . | . | . | . |
| **Select medications (one or more)** | -0.0093 | 0.0081 | -1.1400 | 0.2532 | -0.0252 | 0.0066 |
| **Select medications (none) (REF)** | 0.0000 | . | . | . | . | . |
| **School District - Anglophone** | -0.0561 | 0.0297 | -1.8900 | 0.0588 | -0.1142 | 0.0021 |
| **School District – Francophone (REF)** | 0.0000 | . | . | . | . | . |
| **CIMD - Residential Instability Q2** | -0.0203 | 0.0080 | -2.5200 | 0.0118 | -0.0360 | -0.0045 |
| **CIMD - Residential Instability Q3** | -0.0195 | 0.0085 | -2.2800 | 0.0223 | -0.0362 | -0.0028 |
| **CIMD - Residential Instability Q4** | -0.0290 | 0.0095 | -3.0700 | 0.0021 | -0.0476 | -0.0105 |
| **CIMD – Residential Instability Q5 (most deprived)** | -0.0292 | 0.0118 | -2.4600 | 0.0138 | -0.0524 | -0.0059 |
| **CIMD - Residential Instability Q1 (least deprived) (REF)** | 0.0000 | . | . | . | . | . |
| **CIMD - Economic Dependency Q2** | 0.0109 | 0.0096 | 1.1400 | 0.2549 | -0.0079 | 0.0297 |
| **CIMD - Economic Dependency Q3** | -0.0004 | 0.0096 | -0.0500 | 0.9639 | -0.0193 | 0.0185 |
| **CIMD - Economic Dependency Q4** | -0.0003 | 0.0098 | -0.0300 | 0.9789 | -0.0195 | 0.0190 |
| **CIMD - Economic Dependency Q5 (most deprived)** | -0.0009 | 0.0102 | -0.0900 | 0.9293 | -0.0210 | 0.0192 |
| **CIMD - Economic Dependency Q1 (least deprived) (REF)** | 0.0000 | . | . | . | . | . |
| **CIMD - Ethnocultural Composition Q2** | 0.0046 | 0.0060 | 0.7700 | 0.4412 | -0.0072 | 0.0164 |
| **CIMD - Ethnocultural Composition Q3** | -0.0141 | 0.0085 | -1.6500 | 0.0981 | -0.0307 | 0.0026 |
| **CIMD - Ethnocultural Composition Q4** | -0.0154 | 0.0117 | -1.3100 | 0.1897 | -0.0384 | 0.0076 |
| **CIMD - Ethnocultural Composition Q5 (most deprived)** | -0.0452 | 0.0163 | -2.7700 | 0.0057 | -0.0772 | -0.0132 |
| **CIMD - Ethnocultural Composition Q1 (least deprived) (REF)** | 0.0000 | . | . | . | . | . |
| **CIMD -Situational Vulnerability Q2** | -0.0238 | 0.0093 | -2.5600 | 0.0105 | -0.0421 | -0.0056 |
| **CIMD - Situational Vulnerability Q3** | -0.0383 | 0.0105 | -3.6500 | 0.0003 | -0.0588 | -0.0177 |
| **CIMD -Situational Vulnerability Q4** | -0.0535 | 0.0102 | -5.2500 | <.0001 | -0.0735 | -0.0335 |
| **CIMD -Situational Vulnerability Q5 (most deprived)** | -0.0715 | 0.0111 | -6.4600 | <.0001 | -0.0931 | -0.0498 |
| **CIMD - Situational Vulnerability Q1 (least deprived) (REF)** | 0.0000 | . | . | . | . | . |
| **Social Assistance (any in past 5 years)** | -0.0867 | 0.0064 | -13.6000 | <.0001 | -0.0993 | -0.0742 |
| **Social Assistance (none in past 5 years) (REF)** | 0.0000 | . | . | . | . | . |
| **Program of Study - French Immersion** | 0.0807 | 0.0088 | 9.1500 | <.0001 | 0.0634 | 0.0980 |
| **Program of Study - Other** | -0.1120 | 0.2012 | -0.5600 | 0.5777 | -0.5065 | 0.2824 |
| **Program of Study - French** | 0.0008 | 0.0296 | 0.0300 | 0.9777 | -0.0571 | 0.0588 |
| **Program of Study - English (REF)** | 0.0000 | . | . | . | . | . |
| **Household composition – Adults (age 22+) – No adults in household** | 0.0056 | 0.0271 | 0.2100 | 0.8369 | -0.0475 | 0.0587 |
| **Household composition – Adults (age 22+) – One adult in household** | -0.0296 | 0.0057 | -5.1700 | <.0001 | -0.0408 | -0.0184 |
| **Household composition – Adults (age 22+) – More than one adult in household (REF)** | 0.0000 | . | . | . | . | . |
| **Household composition – Children (age 21 or under) – Student is only child in household** | -0.0098 | 0.0064 | -1.5300 | 0.1272 | -0.0224 | 0.0028 |
| **Household composition – Children (age 21 or under) – Other children in household (REF)** | 0.0000 | . | . | . | . | . |
| **Recent immigrant** | 0.0380 | 0.0336 | 1.1300 | 0.2577 | -0.0278 | 0.1039 |
| **Not a recent immigrant (REF)** | 0.0000 | . | . | . | . | . |

**Supplementary Table S4h. GLM regression estimates – Proportion of report card scores for language meeting minimum acceptable standard (score of 3 or higher) for grades K-8 (AY 2017 – 2020) (Untreated group as reference)**

| **Parameter** | **Estimate** | **Standard**  **Error** | **t Value** | **Pr > \|t\|** | **95% Confidence Limits** | |
| --- | --- | --- | --- | --- | --- | --- |
| **Intercept** | 0.5878 | 0.0321 | 18.3400 | <.0001 | 0.5249 | 0.6506 |
| **Treated ADHD** | -0.0314 | 0.0048 | -6.5300 | 0.2650 | -0.0408 | -0.0220 |
| **Untreated ADHD (REF)** | 0.0000 | . | . | . | . | . |
| **Age** | 0.0160 | 0.0010 | 15.6400 | <.0001 | 0.0140 | 0.0180 |
| **Male** | -0.0660 | 0.0050 | -13.3000 | <.0001 | -0.0757 | -0.0562 |
| **Female (REF)** | 0.0000 | . | . | . | . | . |
| **Household income quintile Q2** | -0.0055 | 0.0082 | -0.6700 | 0.5027 | -0.0215 | 0.0106 |
| **Household income quintile Q3** | 0.0056 | 0.0094 | 0.5900 | 0.5521 | -0.0128 | 0.0239 |
| **Household income quintile Q4** | 0.0081 | 0.0100 | 0.8100 | 0.4165 | -0.0115 | 0.0278 |
| **Household income quintile Q5 (highest income)** | 0.0229 | 0.0110 | 2.0800 | 0.0376 | 0.0013 | 0.0445 |
| **Household income quintile Q1 (lowest income) (REF)** | 0.0000 | . | . | . | . | . |
| **NB Health Zone 2** | -0.0004 | 0.0065 | -0.0700 | 0.9462 | -0.0131 | 0.0123 |
| **NB Health Zone 3** | 0.1054 | 0.0064 | 16.3500 | <.0001 | 0.0928 | 0.1181 |
| **NB Health Zone 4** | 0.0260 | 0.0154 | 1.6900 | 0.0909 | -0.0041 | 0.0562 |
| **NB Health Zone 5** | 0.0487 | 0.0157 | 3.1000 | 0.0019 | 0.0179 | 0.0794 |
| **NB Health Zone 6** | 0.0064 | 0.0114 | 0.5600 | 0.5778 | -0.0161 | 0.0288 |
| **NB Health Zone 7** | 0.0528 | 0.0121 | 4.3600 | <.0001 | 0.0290 | 0.0765 |
| **NB Health Zone 1 (REF)** | 0.0000 | . | . | . | . | . |
| **Comorbid conditions - Mood & anxiety disorders (yes)** | 0.0139 | 0.0115 | 1.2200 | 0.2241 | -0.0085 | 0.0364 |
| **Comorbid conditions - Mood & anxiety disorders (no) (REF)** | 0.0000 | . | . | . | . | . |
| **Comorbid conditions – One or more of: asthma, diabetes, epilepsy, schizophrenia (yes)** | 0.0384 | 0.0206 | 1.8600 | 0.0624 | -0.0020 | 0.0787 |
| **Comorbid conditions – One or more of: asthma, diabetes, epilepsy, schizophrenia (no) (REF)** | 0.0000 | . | . | . | . | . |
| **Select medications (one or more)** | -0.0176 | 0.0072 | -2.4300 | 0.0150 | -0.0318 | -0.0034 |
| **Select medications (none) (REF)** | 0.0000 | . | . | . | . | . |
| **School District - Anglophone** | -0.0778 | 0.0262 | -2.9700 | 0.0030 | -0.1292 | -0.0264 |
| **School District – Francophone (REF)** | 0.0000 | . | . | . | . | . |
| **CIMD - Residential Instability Q2** | -0.0087 | 0.0072 | -1.2200 | 0.2235 | -0.0228 | 0.0053 |
| **CIMD - Residential Instability Q3** | -0.0014 | 0.0076 | -0.1900 | 0.8521 | -0.0163 | 0.0135 |
| **CIMD - Residential Instability Q4** | -0.0051 | 0.0084 | -0.6100 | 0.5427 | -0.0217 | 0.0114 |
| **CIMD – Residential Instability Q5 (most deprived)** | -0.0057 | 0.0106 | -0.5400 | 0.5920 | -0.0264 | 0.0150 |
| **CIMD - Residential Instability Q1 (least deprived) (REF)** | 0.0000 | . | . | . | . | . |
| **CIMD - Economic Dependency Q2** | -0.0039 | 0.0086 | -0.4500 | 0.6508 | -0.0206 | 0.0129 |
| **CIMD - Economic Dependency Q3** | -0.0053 | 0.0086 | -0.6100 | 0.5395 | -0.0221 | 0.0116 |
| **CIMD - Economic Dependency Q4** | -0.0024 | 0.0088 | -0.2700 | 0.7867 | -0.0196 | 0.0148 |
| **CIMD - Economic Dependency Q5 (most deprived)** | -0.0091 | 0.0091 | -1.0000 | 0.3190 | -0.0270 | 0.0088 |
| **CIMD - Economic Dependency Q1 (least deprived) (REF)** | 0.0000 | . | . | . | . | . |
| **CIMD - Ethnocultural Composition Q2** | 0.0060 | 0.0054 | 1.1200 | 0.2633 | -0.0045 | 0.0165 |
| **CIMD - Ethnocultural Composition Q3** | 0.0013 | 0.0076 | 0.1700 | 0.8682 | -0.0136 | 0.0161 |
| **CIMD - Ethnocultural Composition Q4** | 0.0060 | 0.0105 | 0.5800 | 0.5646 | -0.0145 | 0.0266 |
| **CIMD - Ethnocultural Composition Q5 (most deprived)** | -0.0403 | 0.0146 | -2.7600 | 0.0058 | -0.0689 | -0.0116 |
| **CIMD - Ethnocultural Composition Q1 (least deprived) (REF)** | 0.0000 | . | . | . | . | . |
| **CIMD -Situational Vulnerability Q2** | -0.0224 | 0.0083 | -2.7000 | 0.0069 | -0.0387 | -0.0061 |
| **CIMD - Situational Vulnerability Q3** | -0.0359 | 0.0093 | -3.8500 | 0.0001 | -0.0542 | -0.0176 |
| **CIMD -Situational Vulnerability Q4** | -0.0524 | 0.0091 | -5.7700 | <.0001 | -0.0703 | -0.0346 |
| **CIMD -Situational Vulnerability Q5 (most deprived)** | -0.0642 | 0.0099 | -6.5000 | <.0001 | -0.0835 | -0.0448 |
| **CIMD - Situational Vulnerability Q1 (least deprived) (REF)** | 0.0000 | . | . | . | . | . |
| **Social Assistance (any in past 5 years)** | -0.0944 | 0.0057 | -16.5900 | <.0001 | -0.1055 | -0.0832 |
| **Social Assistance (none in past 5 years) (REF)** | 0.0000 | . | . | . | . | . |
| **Program of Study - French Immersion** | 0.0936 | 0.0079 | 11.8700 | <.0001 | 0.0781 | 0.1090 |
| **Program of Study - Other** | -0.1288 | 0.1615 | -0.8000 | 0.4251 | -0.4453 | 0.1877 |
| **Program of Study - French** | 0.0628 | 0.0261 | 2.4000 | 0.0162 | 0.0116 | 0.1140 |
| **Program of Study - English (REF)** | 0.0000 | . | . | . | . | . |
| **Household composition – Adults (age 22+) – No adults in household** | -0.0273 | 0.0241 | -1.1300 | 0.2565 | -0.0746 | 0.0199 |
| **Household composition – Adults (age 22+) – One adult in household** | -0.0339 | 0.0051 | -6.6400 | <.0001 | -0.0439 | -0.0239 |
| **Household composition – Adults (age 22+) – More than one adult in household (REF)** | 0.0000 | . | . | . | . | . |
| **Household composition – Children (age 21 or under) – Student is only child in household** | 0.0052 | 0.0057 | 0.9100 | 0.3608 | -0.0060 | 0.0165 |
| **Household composition – Children (age 21 or under) – Other children in household (REF)** | 0.0000 | . | . | . | . | . |
| **Recent immigrant** | -0.0443 | 0.0300 | -1.4800 | 0.1391 | -0.1031 | 0.0144 |
| **Not a recent immigrant (REF)** | 0.0000 | . | . | . | . | . |
