# Supplementary material for: Academic Outcomes in Primary and Secondary School Students Prescribed Long-Acting Stimulants for ADHD Management
Source: J Atten Disord. 2025 Oct 7;30(4):493–505. doi: 10.1177/10870547251378169 (PMC12953683; doi:10.1177/10870547251378169)
Supplement: sj-docx-5-jad-10.1177_10870547251378169 – Supplemental material for Academic Outcomes in Primary and Secondary School Students Prescribed Long-Acting Stimulants for ADHD Management [file sj-docx-5-jad-10.1177_10870547251378169.docx]

**Supplementary Table S5a. GLM regression estimates – Mean overall report card score for grades 9-12 (AY 2017 – 2020) (Untreated group as reference)**

| **Parameter** | **Estimate** | **Standard**  **Error** | **t Value** | **Pr > \|t\|** | **95% Confidence Limits** | |
| --- | --- | --- | --- | --- | --- | --- |
| **Intercept** | 73.4777 | 1.9675 | 37.3500 | <.0001 | 69.6212 | 77.3342 |
| **Treated ADHD** | 1.1102 | 0.2466 | 4.5000 | <.0001 | 0.6267 | 1.5936 |
| **Untreated ADHD (REF)** | 0.0000 | . | . | . | . | . |
| **Age** | 0.0441 | 0.0858 | 0.5100 | 0.6073 | -0.1241 | 0.2124 |
| **Male** | -3.4994 | 0.2303 | -15.1900 | <.0001 | -3.9509 | -3.0480 |
| **Female (REF)** | 0.0000 | . | . | . | . | . |
| **Household income quintile Q2** | 0.3352 | 0.3906 | 0.8600 | 0.3908 | -0.4304 | 1.1009 |
| **Household income quintile Q3** | 0.4555 | 0.4273 | 1.0700 | 0.2865 | -0.3821 | 1.2930 |
| **Household income quintile Q4** | 1.9728 | 0.4588 | 4.3000 | <.0001 | 1.0735 | 2.8720 |
| **Household income quintile Q5 (highest income)** | 2.5219 | 0.5026 | 5.0200 | <.0001 | 1.5368 | 3.5070 |
| **Household income quintile Q1 (lowest income) (REF)** | 0.0000 | . | . | . | . | . |
| **NB Health Zone 2** | 1.7811 | 0.3206 | 5.5600 | <.0001 | 1.1526 | 2.4095 |
| **NB Health Zone 3** | 2.7949 | 0.3180 | 8.7900 | <.0001 | 2.1715 | 3.4182 |
| **NB Health Zone 4** | 2.6787 | 0.5255 | 5.1000 | <.0001 | 1.6487 | 3.7088 |
| **NB Health Zone 5** | 1.9747 | 0.6293 | 3.1400 | 0.0017 | 0.7412 | 3.2082 |
| **NB Health Zone 6** | 1.7746 | 0.4319 | 4.1100 | <.0001 | 0.9280 | 2.6213 |
| **NB Health Zone 7** | 1.3452 | 0.5437 | 2.4700 | 0.0134 | 0.2795 | 2.4109 |
| **NB Health Zone 1 (REF)** | 0.0000 | . | . | . | . | . |
| **Comorbid conditions - Mood & anxiety disorders (yes)** | -2.3102 | 0.3580 | -6.4500 | <.0001 | -3.0120 | -1.6084 |
| **Comorbid conditions - Mood & anxiety disorders (no) (REF)** | 0.0000 | . | . | . | . | . |
| **Comorbid conditions – One or more of: asthma, diabetes, epilepsy, schizophrenia (yes)** | 0.3996 | 1.0204 | 0.3900 | 0.6954 | -1.6005 | 2.3996 |
| **Comorbid conditions – One or more of: asthma, diabetes, epilepsy, schizophrenia (no) (REF)** | 0.0000 | . | . | . | . | . |
| **Select medications (one or more)** | -1.0060 | 0.3048 | -3.3000 | 0.0010 | -1.6035 | -0.4086 |
| **Select medications (none) (REF)** | 0.0000 | . | . | . | . | . |
| **School District - Anglophone** | -3.3932 | 1.2093 | -2.8100 | 0.0050 | -5.7634 | -1.0229 |
| **School District – Francophone (REF)** | 0.0000 | . | . | . | . | . |
| **CIMD - Residential Instability Q2** | 0.2508 | 0.3254 | 0.7700 | 0.4409 | -0.3871 | 0.8887 |
| **CIMD - Residential Instability Q3** | 0.5017 | 0.3407 | 1.4700 | 0.1408 | -0.1660 | 1.1695 |
| **CIMD - Residential Instability Q4** | 0.9266 | 0.3903 | 2.3700 | 0.0176 | 0.1615 | 1.6916 |
| **CIMD – Residential Instability Q5 (most deprived)** | -0.0871 | 0.5223 | -0.1700 | 0.8676 | -1.1109 | 0.9367 |
| **CIMD - Residential Instability Q1 (least deprived) (REF)** | 0.0000 | . | . | . | . | . |
| **CIMD - Economic Dependency Q2** | -0.0591 | 0.4102 | -0.1400 | 0.8854 | -0.8631 | 0.7449 |
| **CIMD - Economic Dependency Q3** | -0.7733 | 0.4129 | -1.8700 | 0.0611 | -1.5827 | 0.0361 |
| **CIMD - Economic Dependency Q4** | -0.2820 | 0.4244 | -0.6600 | 0.5064 | -1.1139 | 0.5499 |
| **CIMD - Economic Dependency Q5 (most deprived)** | 0.5421 | 0.4320 | 1.2500 | 0.2095 | -0.3046 | 1.3888 |
| **CIMD - Economic Dependency Q1 (least deprived) (REF)** | 0.0000 | . | . | . | . | . |
| **CIMD - Ethnocultural Composition Q2** | -0.4951 | 0.2426 | -2.0400 | 0.0413 | -0.9707 | -0.0195 |
| **CIMD - Ethnocultural Composition Q3** | 0.2152 | 0.3558 | 0.6000 | 0.5452 | -0.4821 | 0.9126 |
| **CIMD - Ethnocultural Composition Q4** | 0.0092 | 0.5478 | 0.0200 | 0.9867 | -1.0646 | 1.0829 |
| **CIMD - Ethnocultural Composition Q5 (most deprived)** | 0.5640 | 0.8124 | 0.6900 | 0.4876 | -1.0284 | 2.1563 |
| **CIMD - Ethnocultural Composition Q1 (least deprived) (REF)** | 0.0000 | . | . | . | . | . |
| **CIMD -Situational Vulnerability Q2** | -1.2479 | 0.3954 | -3.1600 | 0.0016 | -2.0230 | -0.4728 |
| **CIMD - Situational Vulnerability Q3** | -0.8513 | 0.4423 | -1.9200 | 0.0543 | -1.7182 | 0.0156 |
| **CIMD -Situational Vulnerability Q4** | -2.4706 | 0.4285 | -5.7700 | <.0001 | -3.3104 | -1.6308 |
| **CIMD -Situational Vulnerability Q5 (most deprived)** | -1.6462 | 0.4564 | -3.6100 | 0.0003 | -2.5407 | -0.7517 |
| **CIMD - Situational Vulnerability Q1 (least deprived) (REF)** | 0.0000 | . | . | . | . | . |
| **Social Assistance (any in past 5 years)** | -4.6825 | 0.3020 | -15.5100 | <.0001 | -5.2744 | -4.0906 |
| **Social Assistance (none in past 5 years) (REF)** | 0.0000 | . | . | . | . | . |
| **Program of Study - French Immersion** | 4.8578 | 0.3163 | 15.3600 | <.0001 | 4.2378 | 5.4778 |
| **Program of Study - Other** | 4.2827 | 1.3710 | 3.1200 | 0.0018 | 1.5955 | 6.9699 |
| **Program of Study - French** | 2.3634 | 1.2075 | 1.9600 | 0.0503 | -0.0035 | 4.7302 |
| **Program of Study - English (REF)** | 0.0000 | . | . | . | . | . |
| **Household composition – Adults (age 22+) – No adults in household** | -1.6572 | 0.7042 | -2.3500 | 0.0186 | -3.0376 | -0.2768 |
| **Household composition – Adults (age 22+) – One adult in household** | -2.4769 | 0.2513 | -9.8600 | <.0001 | -2.9696 | -1.9843 |
| **Household composition – Adults (age 22+) – More than one adult in household (REF)** | 0.0000 | . | . | . | . | . |
| **Household composition – Children (age 21 or under) – Student is only child in household** | -0.6772 | 0.2381 | -2.8400 | 0.0045 | -1.1439 | -0.2106 |
| **Household composition – Children (age 21 or under) – Other children in household (REF)** | 0.0000 | . | . | . | . | . |
| **Recent immigrant** | 2.1329 | 1.9381 | 1.1000 | 0.2711 | -1.6660 | 5.9319 |
| **Not a recent immigrant (REF)** | 0.0000 | . | . | . | . | . |

**Supplementary Table S5b. GLM regression estimates – Mean report card score for STEM subjects for grades 9-12 (AY 2017 – 2020) (Untreated group as reference)**

| **Parameter** | **Estimate** | **Standard**  **Error** | **t Value** | **Pr > \|t\|** | **95% Confidence Limits** | |
| --- | --- | --- | --- | --- | --- | --- |
| **Intercept** | 68.6625 | 2.4419 | 28.1200 | <.0001 | 63.8761 | 73.4488 |
| **Treated ADHD** | 0.6282 | 0.3036 | 2.0700 | 0.0385 | 0.0331 | 1.2233 |
| **Untreated ADHD (REF)** | 0.0000 | . | . | . | . | . |
| **Age** | -0.1393 | 0.1087 | -1.2800 | 0.2001 | -0.3524 | 0.0738 |
| **Male** | -2.0407 | 0.2843 | -7.1800 | <.0001 | -2.5979 | -1.4835 |
| **Female (REF)** | 0.0000 | . | . | . | . | . |
| **Household income quintile Q2** | 0.9448 | 0.4878 | 1.9400 | 0.0528 | -0.0114 | 1.9009 |
| **Household income quintile Q3** | 1.7134 | 0.5324 | 3.2200 | 0.0013 | 0.6699 | 2.7569 |
| **Household income quintile Q4** | 2.7305 | 0.5725 | 4.7700 | <.0001 | 1.6084 | 3.8526 |
| **Household income quintile Q5 (highest income)** | 3.2175 | 0.6242 | 5.1500 | <.0001 | 1.9940 | 4.4411 |
| **Household income quintile Q1 (lowest income) (REF)** | 0.0000 | . | . | . | . | . |
| **NB Health Zone 2** | 0.8072 | 0.3950 | 2.0400 | 0.0410 | 0.0329 | 1.5814 |
| **NB Health Zone 3** | 2.1222 | 0.3901 | 5.4400 | <.0001 | 1.3576 | 2.8867 |
| **NB Health Zone 4** | 3.6369 | 0.6697 | 5.4300 | <.0001 | 2.3242 | 4.9496 |
| **NB Health Zone 5** | 1.5971 | 0.7827 | 2.0400 | 0.0413 | 0.0629 | 3.1313 |
| **NB Health Zone 6** | 0.8067 | 0.5398 | 1.4900 | 0.1350 | -0.2513 | 1.8647 |
| **NB Health Zone 7** | 1.5493 | 0.6716 | 2.3100 | 0.0211 | 0.2330 | 2.8656 |
| **NB Health Zone 1 (REF)** | 0.0000 | . | . | . | . | . |
| **Comorbid conditions - Mood & anxiety disorders (yes)** | -1.8047 | 0.4454 | -4.0500 | <.0001 | -2.6777 | -0.9316 |
| **Comorbid conditions - Mood & anxiety disorders (no) (REF)** | 0.0000 | . | . | . | . | . |
| **Comorbid conditions – One or more of: asthma, diabetes, epilepsy, schizophrenia (yes)** | 1.1476 | 1.3067 | 0.8800 | 0.3798 | -1.4136 | 3.7088 |
| **Comorbid conditions – One or more of: asthma, diabetes, epilepsy, schizophrenia (no) (REF)** | 0.0000 | . | . | . | . | . |
| **Select medications (one or more)** | 0.0279 | 0.3788 | 0.0700 | 0.9414 | -0.7147 | 0.7704 |
| **Select medications (none) (REF)** | 0.0000 | . | . | . | . | . |
| **School District - Anglophone** | 0.6471 | 1.4830 | 0.4400 | 0.6626 | -2.2597 | 3.5540 |
| **School District – Francophone (REF)** | 0.0000 | . | . | . | . | . |
| **CIMD - Residential Instability Q2** | 0.6529 | 0.3991 | 1.6400 | 0.1018 | -0.1293 | 1.4351 |
| **CIMD - Residential Instability Q3** | 0.7544 | 0.4192 | 1.8000 | 0.0719 | -0.0673 | 1.5761 |
| **CIMD - Residential Instability Q4** | 1.6838 | 0.4794 | 3.5100 | 0.0004 | 0.7441 | 2.6234 |
| **CIMD – Residential Instability Q5 (most deprived)** | 0.8310 | 0.6481 | 1.2800 | 0.1998 | -0.4394 | 2.1014 |
| **CIMD - Residential Instability Q1 (least deprived) (REF)** | 0.0000 | . | . | . | . | . |
| **CIMD - Economic Dependency Q2** | -0.3333 | 0.5002 | -0.6700 | 0.5053 | -1.3138 | 0.6473 |
| **CIMD - Economic Dependency Q3** | -0.6238 | 0.5042 | -1.2400 | 0.2161 | -1.6121 | 0.3646 |
| **CIMD - Economic Dependency Q4** | -0.4046 | 0.5189 | -0.7800 | 0.4356 | -1.4218 | 0.6126 |
| **CIMD - Economic Dependency Q5 (most deprived)** | 0.1390 | 0.5291 | 0.2600 | 0.7928 | -0.8981 | 1.1760 |
| **CIMD - Economic Dependency Q1 (least deprived) (REF)** | 0.0000 | . | . | . | . | . |
| **CIMD - Ethnocultural Composition Q2** | -0.0151 | 0.3003 | -0.0500 | 0.9599 | -0.6038 | 0.5736 |
| **CIMD - Ethnocultural Composition Q3** | -0.0680 | 0.4373 | -0.1600 | 0.8765 | -0.9252 | 0.7892 |
| **CIMD - Ethnocultural Composition Q4** | -0.0599 | 0.6717 | -0.0900 | 0.9289 | -1.3766 | 1.2567 |
| **CIMD - Ethnocultural Composition Q5 (most deprived)** | -0.1116 | 1.0018 | -0.1100 | 0.9113 | -2.0752 | 1.8520 |
| **CIMD - Ethnocultural Composition Q1 (least deprived) (REF)** | 0.0000 | . | . | . | . | . |
| **CIMD -Situational Vulnerability Q2** | -1.7224 | 0.4814 | -3.5800 | 0.0003 | -2.6661 | -0.7787 |
| **CIMD - Situational Vulnerability Q3** | -1.4700 | 0.5421 | -2.7100 | 0.0067 | -2.5327 | -0.4074 |
| **CIMD -Situational Vulnerability Q4** | -2.1075 | 0.5256 | -4.0100 | <.0001 | -3.1377 | -1.0773 |
| **CIMD -Situational Vulnerability Q5 (most deprived)** | -1.2255 | 0.5629 | -2.1800 | 0.0295 | -2.3289 | -0.1221 |
| **CIMD - Situational Vulnerability Q1 (least deprived) (REF)** | 0.0000 | . | . | . | . | . |
| **Social Assistance (any in past 5 years)** | -3.7536 | 0.3811 | -9.8500 | <.0001 | -4.5006 | -3.0067 |
| **Social Assistance (none in past 5 years) (REF)** | 0.0000 | . | . | . | . | . |
| **Program of Study - French Immersion** | 4.1868 | 0.3801 | 11.0100 | <.0001 | 3.4417 | 4.9319 |
| **Program of Study - Other** | 7.1594 | 1.7589 | 4.0700 | <.0001 | 3.7117 | 10.6071 |
| **Program of Study - French** | 3.9684 | 1.4813 | 2.6800 | 0.0074 | 1.0648 | 6.8719 |
| **Program of Study - English (REF)** | 0.0000 | . | . | . | . | . |
| **Household composition – Adults (age 22+) – No adults in household** | -1.1577 | 1.0087 | -1.1500 | 0.2511 | -3.1348 | 0.8194 |
| **Household composition – Adults (age 22+) – One adult in household** | -2.3872 | 0.3109 | -7.6800 | <.0001 | -2.9967 | -1.7777 |
| **Household composition – Adults (age 22+) – More than one adult in household (REF)** | 0.0000 | . | . | . | . | . |
| **Household composition – Children (age 21 or under) – Student is only child in household** | -0.3003 | 0.2951 | -1.0200 | 0.3089 | -0.8787 | 0.2782 |
| **Household composition – Children (age 21 or under) – Other children in household (REF)** | 0.0000 | . | . | . | . | . |
| **Recent immigrant** | 2.3628 | 2.4124 | 0.9800 | 0.3274 | -2.3657 | 7.0914 |
| **Not a recent immigrant (REF)** | 0.0000 | . | . | . | . | . |

**Supplementary Table S5c. GLM regression estimates – Mean report card score for math for grades 9-12 (AY 2017 – 2020) (Untreated group as reference)**

| **Parameter** | **Estimate** | **Standard**  **Error** | **t Value** | **Pr > \|t\|** | **95% Confidence Limits** | |
| --- | --- | --- | --- | --- | --- | --- |
| **Intercept** | 65.5076 | 2.8862 | 22.7000 | <.0001 | 59.8503 | 71.1650 |
| **Treated ADHD** | 0.4459 | 0.3520 | 1.2700 | 0.2053 | -0.2441 | 1.1360 |
| **Untreated ADHD (REF)** | 0.0000 | . | . | . | . | . |
| **Age** | -0.0207 | 0.1282 | -0.1600 | 0.8718 | -0.2720 | 0.2306 |
| **Male** | -2.1306 | 0.3283 | -6.4900 | <.0001 | -2.7743 | -1.4870 |
| **Female (REF)** | 0.0000 | . | . | . | . | . |
| **Household income quintile Q2** | 0.5462 | 0.5692 | 0.9600 | 0.3372 | -0.5695 | 1.6620 |
| **Household income quintile Q3** | 1.6029 | 0.6173 | 2.6000 | 0.0094 | 0.3930 | 2.8128 |
| **Household income quintile Q4** | 2.4995 | 0.6620 | 3.7800 | 0.0002 | 1.2019 | 3.7971 |
| **Household income quintile Q5 (highest income)** | 2.7280 | 0.7202 | 3.7900 | 0.0002 | 1.3162 | 4.1398 |
| **Household income quintile Q1 (lowest income) (REF)** | 0.0000 | . | . | . | . | . |
| **NB Health Zone 2** | 0.1254 | 0.4568 | 0.2700 | 0.7836 | -0.7700 | 1.0209 |
| **NB Health Zone 3** | 1.0012 | 0.4514 | 2.2200 | 0.0266 | 0.1164 | 1.8860 |
| **NB Health Zone 4** | 2.6292 | 0.7912 | 3.3200 | 0.0009 | 1.0783 | 4.1801 |
| **NB Health Zone 5** | 1.1344 | 0.9079 | 1.2500 | 0.2115 | -0.6453 | 2.9140 |
| **NB Health Zone 6** | 0.6495 | 0.6307 | 1.0300 | 0.3031 | -0.5867 | 1.8858 |
| **NB Health Zone 7** | 0.1939 | 0.7796 | 0.2500 | 0.8036 | -1.3343 | 1.7222 |
| **NB Health Zone 1 (REF)** | 0.0000 | . | . | . | . | . |
| **Comorbid conditions - Mood & anxiety disorders (yes)** | -1.4838 | 0.5153 | -2.8800 | 0.0040 | -2.4938 | -0.4737 |
| **Comorbid conditions - Mood & anxiety disorders (no) (REF)** | 0.0000 | . | . | . | . | . |
| **Comorbid conditions – One or more of: asthma, diabetes, epilepsy, schizophrenia (yes)** | -0.1693 | 1.5731 | -0.1100 | 0.9143 | -3.2528 | 2.9143 |
| **Comorbid conditions – One or more of: asthma, diabetes, epilepsy, schizophrenia (no) (REF)** | 0.0000 | . | . | . | . | . |
| **Select medications (one or more)** | 0.5081 | 0.4395 | 1.1600 | 0.2477 | -0.3534 | 1.3696 |
| **Select medications (none) (REF)** | 0.0000 | . | . | . | . | . |
| **School District - Anglophone** | 3.0877 | 1.7577 | 1.7600 | 0.0790 | -0.3578 | 6.5331 |
| **School District – Francophone (REF)** | 0.0000 | . | . | . | . | . |
| **CIMD - Residential Instability Q2** | 0.6389 | 0.4583 | 1.3900 | 0.1634 | -0.2595 | 1.5373 |
| **CIMD - Residential Instability Q3** | 1.0527 | 0.4833 | 2.1800 | 0.0294 | 0.1053 | 2.0002 |
| **CIMD - Residential Instability Q4** | 2.0529 | 0.5524 | 3.7200 | 0.0002 | 0.9702 | 3.1356 |
| **CIMD – Residential Instability Q5 (most deprived)** | 1.6232 | 0.7506 | 2.1600 | 0.0306 | 0.1519 | 3.0945 |
| **CIMD - Residential Instability Q1 (least deprived) (REF)** | 0.0000 | . | . | . | . | . |
| **CIMD - Economic Dependency Q2** | -0.5349 | 0.5705 | -0.9400 | 0.3485 | -1.6531 | 0.5833 |
| **CIMD - Economic Dependency Q3** | -0.5755 | 0.5795 | -0.9900 | 0.3207 | -1.7113 | 0.5604 |
| **CIMD - Economic Dependency Q4** | -0.5965 | 0.5940 | -1.0000 | 0.3153 | -1.7607 | 0.5678 |
| **CIMD - Economic Dependency Q5 (most deprived)** | 0.0822 | 0.6066 | 0.1400 | 0.8922 | -1.1068 | 1.2713 |
| **CIMD - Economic Dependency Q1 (least deprived) (REF)** | 0.0000 | . | . | . | . | . |
| **CIMD - Ethnocultural Composition Q2** | -0.0273 | 0.3465 | -0.0800 | 0.9373 | -0.7066 | 0.6520 |
| **CIMD - Ethnocultural Composition Q3** | -0.6543 | 0.5050 | -1.3000 | 0.1951 | -1.6443 | 0.3356 |
| **CIMD - Ethnocultural Composition Q4** | -0.3291 | 0.7728 | -0.4300 | 0.6702 | -1.8439 | 1.1857 |
| **CIMD - Ethnocultural Composition Q5 (most deprived)** | 0.5239 | 1.1907 | 0.4400 | 0.6599 | -1.8099 | 2.8578 |
| **CIMD - Ethnocultural Composition Q1 (least deprived) (REF)** | 0.0000 | . | . | . | . | . |
| **CIMD -Situational Vulnerability Q2** | -1.9723 | 0.5502 | -3.5800 | 0.0003 | -3.0508 | -0.8937 |
| **CIMD - Situational Vulnerability Q3** | -1.0212 | 0.6196 | -1.6500 | 0.0994 | -2.2357 | 0.1933 |
| **CIMD -Situational Vulnerability Q4** | -2.3788 | 0.6027 | -3.9500 | <.0001 | -3.5601 | -1.1975 |
| **CIMD -Situational Vulnerability Q5 (most deprived)** | -1.1804 | 0.6448 | -1.8300 | 0.0672 | -2.4443 | 0.0835 |
| **CIMD - Situational Vulnerability Q1 (least deprived) (REF)** | 0.0000 | . | . | . | . | . |
| **Social Assistance (any in past 5 years)** | -3.8335 | 0.4493 | -8.5300 | <.0001 | -4.7141 | -2.9528 |
| **Social Assistance (none in past 5 years) (REF)** | 0.0000 | . | . | . | . | . |
| **Program of Study - French Immersion** | 4.0794 | 0.4271 | 9.5500 | <.0001 | 3.2421 | 4.9166 |
| **Program of Study - Other** | 13.4627 | 2.2218 | 6.0600 | <.0001 | 9.1076 | 17.8179 |
| **Program of Study - French** | 5.3100 | 1.7578 | 3.0200 | 0.0025 | 1.8644 | 8.7556 |
| **Program of Study - English (REF)** | 0.0000 | . | . | . | . | . |
| **Household composition – Adults (age 22+) – No adults in household** | -3.1886 | 1.3357 | -2.3900 | 0.0170 | -5.8068 | -0.5704 |
| **Household composition – Adults (age 22+) – One adult in household** | -2.4360 | 0.3589 | -6.7900 | <.0001 | -3.1396 | -1.7324 |
| **Household composition – Adults (age 22+) – More than one adult in household (REF)** | 0.0000 | . | . | . | . | . |
| **Household composition – Children (age 21 or under) – Student is only child in household** | -0.3302 | 0.3435 | -0.9600 | 0.3364 | -1.0036 | 0.3431 |
| **Household composition – Children (age 21 or under) – Other children in household (REF)** | 0.0000 | . | . | . | . | . |
| **Recent immigrant** | 3.2038 | 2.6759 | 1.2000 | 0.2312 | -2.0414 | 8.4490 |
| **Not a recent immigrant (REF)** | 0.0000 | . | . | . | . | . |

**Supplementary Table S5d. GLM regression estimates – Mean report card score for language for grades 9-12 (AY 2017 – 2020) (Untreated group as reference)**

| **Parameter** | **Estimate** | **Standard**  **Error** | **t Value** | **Pr > \|t\|** | **95% Confidence Limits** | |
| --- | --- | --- | --- | --- | --- | --- |
| **Intercept** | 70.1140 | 2.0991 | 33.4000 | <.0001 | 65.9995 | 74.2285 |
| **Treated ADHD** | 0.5080 | 0.2623 | 1.9400 | 0.0428 | -0.0060 | 1.0221 |
| **Untreated ADHD (REF)** | 0.0000 | . | . | . | . | . |
| **Age** | 0.2789 | 0.0919 | 3.0400 | 0.0024 | 0.0988 | 0.4589 |
| **Male** | -4.5394 | 0.2439 | -18.6200 | <.0001 | -5.0174 | -4.0614 |
| **Female (REF)** | 0.0000 | . | . | . | . | . |
| **Household income quintile Q2** | 0.1496 | 0.4174 | 0.3600 | 0.7201 | -0.6686 | 0.9678 |
| **Household income quintile Q3** | 0.0839 | 0.4575 | 0.1800 | 0.8545 | -0.8129 | 0.9807 |
| **Household income quintile Q4** | 1.3118 | 0.4910 | 2.6700 | 0.0076 | 0.3494 | 2.2742 |
| **Household income quintile Q5 (highest income)** | 1.2129 | 0.5361 | 2.2600 | 0.0237 | 0.1619 | 2.2638 |
| **Household income quintile Q1 (lowest income) (REF)** | 0.0000 | . | . | . | . | . |
| **NB Health Zone 2** | 2.1117 | 0.3373 | 6.2600 | <.0001 | 1.4505 | 2.7729 |
| **NB Health Zone 3** | 3.1359 | 0.3343 | 9.3800 | <.0001 | 2.4806 | 3.7912 |
| **NB Health Zone 4** | 3.7794 | 0.5811 | 6.5000 | <.0001 | 2.6405 | 4.9184 |
| **NB Health Zone 5** | 0.9513 | 0.6757 | 1.4100 | 0.1592 | -0.3731 | 2.2757 |
| **NB Health Zone 6** | 2.2479 | 0.4681 | 4.8000 | <.0001 | 1.3303 | 3.1655 |
| **NB Health Zone 7** | 0.4216 | 0.5776 | 0.7300 | 0.4654 | -0.7106 | 1.5539 |
| **NB Health Zone 1 (REF)** | 0.0000 | . | . | . | . | . |
| **Comorbid conditions - Mood & anxiety disorders (yes)** | -1.2406 | 0.3815 | -3.2500 | 0.0012 | -1.9884 | -0.4927 |
| **Comorbid conditions - Mood & anxiety disorders (no) (REF)** | 0.0000 | . | . | . | . | . |
| **Comorbid conditions – One or more of: asthma, diabetes, epilepsy, schizophrenia (yes)** | 0.7920 | 1.1112 | 0.7100 | 0.4760 | -1.3861 | 2.9700 |
| **Comorbid conditions – One or more of: asthma, diabetes, epilepsy, schizophrenia (no) (REF)** | 0.0000 | . | . | . | . |  |
| **Select medications (one or more)** | -0.8185 | 0.3259 | -2.5100 | 0.0120 | -1.4574 | -0.1796 |
| **Select medications (none) (REF)** | 0.0000 | . | . | . | . | . |
| **School District - Anglophone** | -1.3328 | 1.2896 | -1.0300 | 0.3014 | -3.8605 | 1.1949 |
| **School District – Francophone (REF)** | 0.0000 | . | . | . | . | . |
| **CIMD - Residential Instability Q2** | -0.1053 | 0.3431 | -0.3100 | 0.7588 | -0.7778 | 0.5671 |
| **CIMD - Residential Instability Q3** | 0.3493 | 0.3611 | 0.9700 | 0.3335 | -0.3586 | 1.0571 |
| **CIMD - Residential Instability Q4** | 0.7859 | 0.4136 | 1.9000 | 0.0574 | -0.0248 | 1.5966 |
| **CIMD – Residential Instability Q5 (most deprived)** | -0.3206 | 0.5555 | -0.5800 | 0.5638 | -1.4095 | 0.7682 |
| **CIMD - Residential Instability Q1 (least deprived) (REF)** | 0.0000 | . | . | . | . | . |
| **CIMD - Economic Dependency Q2** | -0.4358 | 0.4297 | -1.0100 | 0.3105 | -1.2780 | 0.4064 |
| **CIMD - Economic Dependency Q3** | -0.4666 | 0.4341 | -1.0800 | 0.2824 | -1.3174 | 0.3842 |
| **CIMD - Economic Dependency Q4** | -0.3674 | 0.4464 | -0.8200 | 0.4105 | -1.2424 | 0.5076 |
| **CIMD - Economic Dependency Q5 (most deprived)** | 0.1047 | 0.4544 | 0.2300 | 0.8178 | -0.7860 | 0.9954 |
| **CIMD - Economic Dependency Q1 (least deprived) (REF)** | 0.0000 | . | . | . | . | . |
| **CIMD - Ethnocultural Composition Q2** | -0.2309 | 0.2577 | -0.9000 | 0.3703 | -0.7361 | 0.2742 |
| **CIMD - Ethnocultural Composition Q3** | 0.3686 | 0.3768 | 0.9800 | 0.3280 | -0.3700 | 1.1071 |
| **CIMD - Ethnocultural Composition Q4** | -0.0565 | 0.5761 | -0.1000 | 0.9218 | -1.1857 | 1.0726 |
| **CIMD - Ethnocultural Composition Q5 (most deprived)** | 1.2794 | 0.8737 | 1.4600 | 0.1431 | -0.4332 | 2.9920 |
| **CIMD - Ethnocultural Composition Q1 (least deprived) (REF)** | 0.0000 | . | . | . | . | . |
| **CIMD -Situational Vulnerability Q2** | -1.4545 | 0.4129 | -3.5200 | 0.0004 | -2.2639 | -0.6452 |
| **CIMD - Situational Vulnerability Q3** | -1.7884 | 0.4632 | -3.8600 | 0.0001 | -2.6963 | -0.8806 |
| **CIMD -Situational Vulnerability Q4** | -2.9089 | 0.4501 | -6.4600 | <.0001 | -3.7912 | -2.0266 |
| **CIMD -Situational Vulnerability Q5 (most deprived)** | -2.8794 | 0.4808 | -5.9900 | <.0001 | -3.8217 | -1.9370 |
| **CIMD - Situational Vulnerability Q1 (least deprived) (REF)** | 0.0000 | . | . | . | . | . |
| **Social Assistance (any in past 5 years)** | -4.2778 | 0.3271 | -13.0800 | <.0001 | -4.9190 | -3.6366 |
| **Social Assistance (none in past 5 years) (REF)** | 0.0000 | . | . | . | . | . |
| **Program of Study - French Immersion** | 3.6771 | 0.3250 | 11.3100 | <.0001 | 3.0400 | 4.3142 |
| **Program of Study - Other** | 4.3313 | 1.5641 | 2.7700 | 0.0056 | 1.2655 | 7.3971 |
| **Program of Study - French** | 1.3514 | 1.2879 | 1.0500 | 0.2940 | -1.1729 | 3.8757 |
| **Program of Study - English (REF)** | 0.0000 | . | . | . | . | . |
| **Household composition – Adults (age 22+) – No adults in household** | -0.4102 | 0.8489 | -0.4800 | 0.6290 | -2.0741 | 1.2537 |
| **Household composition – Adults (age 22+) – One adult in household** | -2.0247 | 0.2675 | -7.5700 | <.0001 | -2.5491 | -1.5004 |
| **Household composition – Adults (age 22+) – More than one adult in household (REF)** | 0.0000 | . | . | . | . | . |
| **Household composition – Children (age 21 or under) – Student is only child in household** | -0.3428 | 0.2532 | -1.3500 | 0.1757 | -0.8391 | 0.1534 |
| **Household composition – Children (age 21 or under) – Other children in household (REF)** | 0.0000 | . | . | . | . | . |
| **Recent immigrant** | 3.2193 | 2.0477 | 1.5700 | 0.1159 | -0.7944 | 7.2330 |
| **Not a recent immigrant (REF)** | 0.0000 | . | . | . | . | . |
