# Supplementary material for: Academic Outcomes in Primary and Secondary School Students Prescribed Long-Acting Stimulants for ADHD Management
Source: J Atten Disord. 2025 Oct 7;30(4):493–505. doi: 10.1177/10870547251378169 (PMC12953683; doi:10.1177/10870547251378169)
Supplement: sj-docx-6-jad-10.1177_10870547251378169 – Supplemental material for Academic Outcomes in Primary and Secondary School Students Prescribed Long-Acting Stimulants for ADHD Management [file sj-docx-6-jad-10.1177_10870547251378169.docx]

**Supplementary Table S6a. GLM regression estimates – Proportion of overall courses failed (score < 60%) for grades 9-12 (AY 2017 – 2020)**

| **Parameter** | **Estimate** | **Standard**  **Error** | **t Value** | **Pr > \|t\|** | **95% Confidence Limits** | |
| --- | --- | --- | --- | --- | --- | --- |
| **Intercept** | 0.0010 | 0.0121 | 0.0800 | 0.9361 | -0.0228 | 0.0247 |
| **Treated ADHD** | 0.0354 | 0.0028 | 12.5300 | <.0001 | 0.0298 | 0.0409 |
| **Untreated ADHD** | 0.0614 | 0.0019 | 31.7500 | <.0001 | 0.0576 | 0.0652 |
| **No ADHD (REF)** | 0.0000 | . | . | . | . | . |
| **Age** | 0.0035 | 0.0005 | 6.5200 | <.0001 | 0.0024 | 0.0045 |
| **Male** | 0.0261 | 0.0013 | 19.9800 | <.0001 | 0.0236 | 0.0287 |
| **Female (REF)** | 0.0000 | . | . | . | . | . |
| **Household income quintile Q2** | -0.0139 | 0.0025 | -5.6300 | <.0001 | -0.0187 | -0.0091 |
| **Household income quintile Q3** | -0.0186 | 0.0027 | -6.9400 | <.0001 | -0.0238 | -0.0133 |
| **Household income quintile Q4** | -0.0235 | 0.0029 | -8.1700 | <.0001 | -0.0291 | -0.0179 |
| **Household income quintile Q5 (highest income)** | -0.0341 | 0.0031 | -10.8900 | <.0001 | -0.0402 | -0.0279 |
| **Household income quintile Q1 (lowest income) (REF)** | 0.0000 | . | . | . | . | . |
| **NB Health Zone 2** | -0.0072 | 0.0019 | -3.7800 | 0.0002 | -0.0109 | -0.0035 |
| **NB Health Zone 3** | -0.0194 | 0.0019 | -10.2800 | <.0001 | -0.0231 | -0.0157 |
| **NB Health Zone 4** | -0.0292 | 0.0031 | -9.2800 | <.0001 | -0.0353 | -0.0230 |
| **NB Health Zone 5** | -0.0116 | 0.0041 | -2.8600 | 0.0042 | -0.0196 | -0.0037 |
| **NB Health Zone 6** | -0.0204 | 0.0029 | -7.1500 | <.0001 | -0.0260 | -0.0148 |
| **NB Health Zone 7** | -0.0106 | 0.0031 | -3.4300 | 0.0006 | -0.0166 | -0.0045 |
| **NB Health Zone 1 (REF)** | 0.0000 | . | . | . | . | . |
| **Comorbid conditions - Mood & anxiety disorders (yes)** | 0.0418 | 0.0027 | 15.4800 | <.0001 | 0.0365 | 0.0471 |
| **Comorbid conditions - Mood & anxiety disorders (no) (REF)** | 0.0000 | . | . | . | . | . |
| **Comorbid conditions – One or more of: asthma, diabetes, epilepsy, schizophrenia (yes)** | -0.0075 | 0.0076 | -0.9800 | 0.3248 | -0.0223 | 0.0074 |
| **Comorbid conditions – One or more of: asthma, diabetes, epilepsy, schizophrenia (no) (REF)** | 0.0000 | . | . | . | . | . |
| **Select medications (one or more)** | 0.0375 | 0.0030 | 12.5800 | <.0001 | 0.0316 | 0.0433 |
| **Select medications (none) (REF)** | 0.0000 | . | . | . | . | . |
| **School District - Anglophone** | 0.0229 | 0.0075 | 3.0700 | 0.0021 | 0.0083 | 0.0376 |
| **School District – Francophone (REF)** | 0.0000 | . | . | . | . | . |
| **CIMD - Residential Instability Q2** | -0.0015 | 0.0019 | -0.7700 | 0.4406 | -0.0052 | 0.0023 |
| **CIMD - Residential Instability Q3** | -0.0037 | 0.0021 | -1.7800 | 0.0753 | -0.0077 | 0.0004 |
| **CIMD - Residential Instability Q4** | 0.0028 | 0.0024 | 1.1900 | 0.2352 | -0.0018 | 0.0075 |
| **CIMD – Residential Instability Q5 (most deprived)** | 0.0091 | 0.0033 | 2.7700 | 0.0056 | 0.0027 | 0.0155 |
| **CIMD - Residential Instability Q1 (least deprived) (REF)** | 0.0000 | . | . | . | . | . |
| **CIMD - Economic Dependency Q2** | 0.0059 | 0.0024 | 2.4300 | 0.0149 | 0.0012 | 0.0107 |
| **CIMD - Economic Dependency Q3** | 0.0026 | 0.0024 | 1.0900 | 0.2769 | -0.0021 | 0.0074 |
| **CIMD - Economic Dependency Q4** | 0.0030 | 0.0025 | 1.1800 | 0.2372 | -0.0020 | 0.0079 |
| **CIMD - Economic Dependency Q5 (most deprived)** | -0.0101 | 0.0026 | -3.9100 | <.0001 | -0.0151 | -0.0050 |
| **CIMD - Economic Dependency Q1 (least deprived) (REF)** | 0.0000 | . | . | . | . | . |
| **CIMD - Ethnocultural Composition Q2** | 0.0060 | 0.0015 | 4.0400 | <.0001 | 0.0031 | 0.0089 |
| **CIMD - Ethnocultural Composition Q3** | 0.0027 | 0.0021 | 1.2400 | 0.2150 | -0.0015 | 0.0069 |
| **CIMD - Ethnocultural Composition Q4** | -0.0041 | 0.0032 | -1.2800 | 0.1999 | -0.0104 | 0.0022 |
| **CIMD - Ethnocultural Composition Q5 (most deprived)** | -0.0083 | 0.0050 | -1.6700 | 0.0953 | -0.0181 | 0.0015 |
| **CIMD - Ethnocultural Composition Q1 (least deprived) (REF)** | 0.0000 | . | . | . | . | . |
| **CIMD -Situational Vulnerability Q2** | 0.0052 | 0.0023 | 2.2700 | 0.0230 | 0.0007 | 0.0097 |
| **CIMD - Situational Vulnerability Q3** | 0.0065 | 0.0026 | 2.5100 | 0.0122 | 0.0014 | 0.0115 |
| **CIMD -Situational Vulnerability Q4** | 0.0111 | 0.0025 | 4.3900 | <.0001 | 0.0061 | 0.0160 |
| **CIMD -Situational Vulnerability Q5 (most deprived)** | 0.0178 | 0.0028 | 6.4700 | <.0001 | 0.0124 | 0.0232 |
| **CIMD - Situational Vulnerability Q1 (least deprived) (REF)** | 0.0000 | . | . | . | . | . |
| **Social Assistance (any in past 5 years)** | 0.0912 | 0.0022 | 42.0500 | <.0001 | 0.0870 | 0.0955 |
| **Social Assistance (none in past 5 years) (REF)** | 0.0000 | . | . | . | . | . |
| **Program of Study - French Immersion** | -0.0384 | 0.0016 | -23.5200 | <.0001 | -0.0416 | -0.0352 |
| **Program of Study - Other** | -0.0080 | 0.0098 | -0.8200 | 0.4137 | -0.0272 | 0.0112 |
| **Program of Study - French** | -0.0012 | 0.0074 | -0.1600 | 0.8743 | -0.0157 | 0.0134 |
| **Program of Study - English (REF)** | 0.0000 | . | . | . | . | . |
| **Household composition – Adults (age 22+) – No adults in household** | 0.0424 | 0.0055 | 7.7500 | <.0001 | 0.0317 | 0.0531 |
| **Household composition – Adults (age 22+) – One adult in household** | 0.0315 | 0.0017 | 18.8700 | <.0001 | 0.0282 | 0.0348 |
| **Household composition – Adults (age 22+) – More than one adult in household (REF)** | 0.0000 | . | . | . | . | . |
| **Household composition – Children (age 21 or under) – Student is only child in household** | 0.0015 | 0.0015 | 1.0100 | 0.3110 | -0.0014 | 0.0045 |
| **Household composition – Children (age 21 or under) – Other children in household (REF)** | 0.0000 | . | . | . | . | . |
| **Recent immigrant** | -0.0274 | 0.0040 | -6.7900 | <.0001 | -0.0353 | -0.0195 |
| **Not a recent immigrant (REF)** | 0.0000 | . | . | . | . | . |

**Supplementary Table S6b. GLM regression estimates – Proportion of STEM courses failed (score < 60%) for grades 9-12 (AY 2017 – 2020)**

| **Parameter** | **Estimate** | **Standard**  **Error** | **t Value** | **Pr > \|t\|** | **95% Confidence Limits** | |
| --- | --- | --- | --- | --- | --- | --- |
| **Intercept** | -0.0240 | 0.0177 | -1.3600 | 0.1748 | -0.0588 | 0.0107 |
| **Treated ADHD** | 0.0525 | 0.0042 | 12.6000 | <.0001 | 0.0443 | 0.0606 |
| **Untreated ADHD** | 0.0685 | 0.0029 | 23.7200 | <.0001 | 0.0629 | 0.0742 |
| **No ADHD (REF)** | 0.0000 | . | . | . | . | . |
| **Age** | 0.0086 | 0.0008 | 10.8800 | <.0001 | 0.0071 | 0.0102 |
| **Male** | 0.0291 | 0.0019 | 15.2900 | <.0001 | 0.0254 | 0.0329 |
| **Female (REF)** | 0.0000 | . | . | . | . | . |
| **Household income quintile Q2** | -0.0174 | 0.0036 | -4.8000 | <.0001 | -0.0245 | -0.0103 |
| **Household income quintile Q3** | -0.0244 | 0.0039 | -6.2200 | <.0001 | -0.0321 | -0.0167 |
| **Household income quintile Q4** | -0.0292 | 0.0042 | -6.9200 | <.0001 | -0.0375 | -0.0209 |
| **Household income quintile Q5 (highest income)** | -0.0438 | 0.0046 | -9.5500 | <.0001 | -0.0528 | -0.0348 |
| **Household income quintile Q1 (lowest income) (REF)** | 0.0000 | . | . | . | . | . |
| **NB Health Zone 2** | -0.0052 | 0.0028 | -1.8600 | 0.0623 | -0.0106 | 0.0003 |
| **NB Health Zone 3** | -0.0154 | 0.0027 | -5.6100 | <.0001 | -0.0208 | -0.0100 |
| **NB Health Zone 4** | -0.0421 | 0.0047 | -9.0100 | <.0001 | -0.0513 | -0.0330 |
| **NB Health Zone 5** | -0.0229 | 0.0060 | -3.8300 | 0.0001 | -0.0346 | -0.0112 |
| **NB Health Zone 6** | -0.0219 | 0.0042 | -5.2000 | <.0001 | -0.0302 | -0.0137 |
| **NB Health Zone 7** | -0.0158 | 0.0045 | -3.5000 | 0.0005 | -0.0246 | -0.0069 |
| **NB Health Zone 1 (REF)** | 0.0000 | . | . | . | . | . |
| **Comorbid conditions - Mood & anxiety disorders (yes)** | 0.0450 | 0.0040 | 11.2600 | <.0001 | 0.0372 | 0.0528 |
| **Comorbid conditions - Mood & anxiety disorders (no) (REF)** | 0.0000 | . | . | . | . | . |
| **Comorbid conditions – One or more of: asthma, diabetes, epilepsy, schizophrenia (yes)** | -0.0185 | 0.0113 | -1.6400 | 0.1019 | -0.0407 | 0.0037 |
| **Comorbid conditions – One or more of: asthma, diabetes, epilepsy, schizophrenia (no) (REF)** | 0.0000 | . | . | . | . | . |
| **Select medications (one or more)** | 0.0269 | 0.0045 | 6.0100 | <.0001 | 0.0181 | 0.0356 |
| **Select medications (none) (REF)** | 0.0000 | . | . | . | . | . |
| **School District - Anglophone** | -0.0128 | 0.0107 | -1.1900 | 0.2327 | -0.0339 | 0.0082 |
| **School District – Francophone (REF)** | 0.0000 | . | . | . | . | . |
| **CIMD - Residential Instability Q2** | -0.0039 | 0.0028 | -1.4100 | 0.1582 | -0.0093 | 0.0015 |
| **CIMD - Residential Instability Q3** | -0.0058 | 0.0030 | -1.9500 | 0.0517 | -0.0117 | 0.0000 |
| **CIMD - Residential Instability Q4** | -0.0061 | 0.0035 | -1.7600 | 0.0789 | -0.0130 | 0.0007 |
| **CIMD – Residential Instability Q5 (most deprived)** | 0.0049 | 0.0048 | 1.0300 | 0.3033 | -0.0045 | 0.0143 |
| **CIMD - Residential Instability Q1 (least deprived) (REF)** | 0.0000 | . | . | . | . | . |
| **CIMD - Economic Dependency Q2** | 0.0109 | 0.0035 | 3.1100 | 0.0019 | 0.0041 | 0.0178 |
| **CIMD - Economic Dependency Q3** | 0.0008 | 0.0035 | 0.2300 | 0.8169 | -0.0061 | 0.0077 |
| **CIMD - Economic Dependency Q4** | 0.0009 | 0.0037 | 0.2400 | 0.8130 | -0.0063 | 0.0081 |
| **CIMD - Economic Dependency Q5 (most deprived)** | -0.0110 | 0.0037 | -2.9400 | 0.0032 | -0.0183 | -0.0037 |
| **CIMD - Economic Dependency Q1 (least deprived) (REF)** | 0.0000 | . | . | . | . | . |
| **CIMD - Ethnocultural Composition Q2** | 0.0032 | 0.0022 | 1.5000 | 0.1330 | -0.0010 | 0.0075 |
| **CIMD - Ethnocultural Composition Q3** | -0.0010 | 0.0031 | -0.3100 | 0.7582 | -0.0071 | 0.0052 |
| **CIMD - Ethnocultural Composition Q4** | -0.0032 | 0.0046 | -0.6900 | 0.4877 | -0.0123 | 0.0059 |
| **CIMD - Ethnocultural Composition Q5 (most deprived)** | -0.0090 | 0.0073 | -1.2300 | 0.2170 | -0.0234 | 0.0053 |
| **CIMD - Ethnocultural Composition Q1 (least deprived) (REF)** | 0.0000 | . | . | . | . | . |
| **CIMD -Situational Vulnerability Q2** | 0.0103 | 0.0033 | 3.1100 | 0.0019 | 0.0038 | 0.0168 |
| **CIMD - Situational Vulnerability Q3** | 0.0123 | 0.0037 | 3.2800 | 0.0010 | 0.0049 | 0.0196 |
| **CIMD -Situational Vulnerability Q4** | 0.0131 | 0.0037 | 3.5700 | 0.0004 | 0.0059 | 0.0202 |
| **CIMD -Situational Vulnerability Q5 (most deprived)** | 0.0214 | 0.0040 | 5.3200 | <.0001 | 0.0135 | 0.0292 |
| **CIMD - Situational Vulnerability Q1 (least deprived) (REF)** | 0.0000 | . | . | . | . | . |
| **Social Assistance (any in past 5 years)** | 0.0960 | 0.0032 | 29.6100 | <.0001 | 0.0896 | 0.1023 |
| **Social Assistance (none in past 5 years) (REF)** | 0.0000 | . | . | . | . | . |
| **Program of Study - French Immersion** | -0.0399 | 0.0024 | -16.9800 | <.0001 | -0.0445 | -0.0353 |
| **Program of Study - Other** | -0.0052 | 0.0156 | -0.3300 | 0.7382 | -0.0359 | 0.0254 |
| **Program of Study - French** | 0.0012 | 0.0107 | 0.1200 | 0.9078 | -0.0197 | 0.0222 |
| **Program of Study - English (REF)** | 0.0000 | . | . | . | . | . |
| **Household composition – Adults (age 22+) – No adults in household** | 0.0336 | 0.0093 | 3.6000 | 0.0003 | 0.0153 | 0.0519 |
| **Household composition – Adults (age 22+) – One adult in household** | 0.0377 | 0.0025 | 15.3600 | <.0001 | 0.0329 | 0.0425 |
| **Household composition – Adults (age 22+) – More than one adult in household (REF)** | 0.0000 | . | . | . | . | . |
| **Household composition – Children (age 21 or under) – Student is only child in household** | 0.0019 | 0.0022 | 0.8300 | 0.4051 | -0.0025 | 0.0062 |
| **Household composition – Children (age 21 or under) – Other children in household (REF)** | 0.0000 | . | . | . | . | . |
| **Recent immigrant** | -0.0104 | 0.0058 | -1.7900 | 0.0740 | -0.0218 | 0.0010 |
| **Not a recent immigrant (REF)** | 0.0000 | . | . | . | . | . |

**Supplementary Table S6c. GLM regression estimates – Proportion of math courses failed (score < 60%) for grades 9-12 (AY 2017 – 2020)**

| **Parameter** | **Estimate** | **Standard**  **Error** | **t Value** | **Pr > \|t\|** | **95% Confidence Limits** | |
| --- | --- | --- | --- | --- | --- | --- |
| **Intercept** | 0.0291 | 0.0215 | 1.3500 | 0.1769 | -0.0131 | 0.0712 |
| **Treated ADHD** | 0.0458 | 0.0051 | 8.9800 | <.0001 | 0.0358 | 0.0557 |
| **Untreated ADHD** | 0.0635 | 0.0035 | 18.0800 | <.0001 | 0.0566 | 0.0704 |
| **No ADHD (REF)** | 0.0000 | . | . | . | . | . |
| **Age** | 0.0063 | 0.0010 | 6.4700 | <.0001 | 0.0044 | 0.0082 |
| **Male** | 0.0305 | 0.0023 | 13.3000 | <.0001 | 0.0260 | 0.0350 |
| **Female (REF)** | 0.0000 | . | . | . | . | . |
| **Household income quintile Q2** | -0.0184 | 0.0044 | -4.1800 | <.0001 | -0.0270 | -0.0098 |
| **Household income quintile Q3** | -0.0250 | 0.0047 | -5.2700 | <.0001 | -0.0343 | -0.0157 |
| **Household income quintile Q4** | -0.0293 | 0.0051 | -5.7500 | <.0001 | -0.0393 | -0.0193 |
| **Household income quintile Q5 (highest income)** | -0.0431 | 0.0055 | -7.7800 | <.0001 | -0.0540 | -0.0323 |
| **Household income quintile Q1 (lowest income) (REF)** | 0.0000 | . | . | . | . | . |
| **NB Health Zone 2** | -0.0026 | 0.0033 | -0.8000 | 0.4256 | -0.0092 | 0.0039 |
| **NB Health Zone 3** | -0.0064 | 0.0033 | -1.9400 | 0.0518 | -0.0128 | 0.0001 |
| **NB Health Zone 4** | -0.0268 | 0.0058 | -4.6000 | <.0001 | -0.0383 | -0.0154 |
| **NB Health Zone 5** | -0.0049 | 0.0073 | -0.6700 | 0.5043 | -0.0193 | 0.0095 |
| **NB Health Zone 6** | -0.0197 | 0.0052 | -3.7700 | 0.0002 | -0.0299 | -0.0095 |
| **NB Health Zone 7** | -0.0177 | 0.0054 | -3.2700 | 0.0011 | -0.0283 | -0.0071 |
| **NB Health Zone 1 (REF)** | 0.0000 | . | . | . | . | . |
| **Comorbid conditions - Mood & anxiety disorders (yes)** | 0.0439 | 0.0049 | 9.0200 | <.0001 | 0.0344 | 0.0534 |
| **Comorbid conditions - Mood & anxiety disorders (no) (REF)** | 0.0000 | . | . | . | . | . |
| **Comorbid conditions – One or more of: asthma, diabetes, epilepsy, schizophrenia (yes)** | -0.0192 | 0.0137 | -1.4000 | 0.1606 | -0.0460 | 0.0076 |
| **Comorbid conditions – One or more of: asthma, diabetes, epilepsy, schizophrenia (no) (REF)** | 0.0000 | . | . | . | . | . |
| **Select medications (one or more)** | 0.0234 | 0.0055 | 4.2600 | <.0001 | 0.0126 | 0.0341 |
| **Select medications (none) (REF)** | 0.0000 | . | . | . | . | . |
| **School District - Anglophone** | -0.0322 | 0.0128 | -2.5200 | 0.0118 | -0.0572 | -0.0071 |
| **School District – Francophone (REF)** | 0.0000 | . | . | . | . | . |
| **CIMD - Residential Instability Q2** | -0.0083 | 0.0033 | -2.4800 | 0.0132 | -0.0148 | -0.0017 |
| **CIMD - Residential Instability Q3** | -0.0090 | 0.0036 | -2.4900 | 0.0128 | -0.0160 | -0.0019 |
| **CIMD - Residential Instability Q4** | -0.0096 | 0.0042 | -2.2900 | 0.0223 | -0.0178 | -0.0014 |
| **CIMD – Residential Instability Q5 (most deprived)** | -0.0018 | 0.0058 | -0.3100 | 0.7540 | -0.0132 | 0.0095 |
| **CIMD - Residential Instability Q1 (least deprived) (REF)** | 0.0000 | . | . | . | . | . |
| **CIMD - Economic Dependency Q2** | 0.0112 | 0.0042 | 2.6700 | 0.0077 | 0.0030 | 0.0194 |
| **CIMD - Economic Dependency Q3** | -0.0008 | 0.0042 | -0.2000 | 0.8442 | -0.0091 | 0.0074 |
| **CIMD - Economic Dependency Q4** | 0.0011 | 0.0044 | 0.2500 | 0.8008 | -0.0075 | 0.0097 |
| **CIMD - Economic Dependency Q5 (most deprived)** | -0.0108 | 0.0045 | -2.4100 | 0.0158 | -0.0195 | -0.0020 |
| **CIMD - Economic Dependency Q1 (least deprived) (REF)** | 0.0000 | . | . | . | . | . |
| **CIMD - Ethnocultural Composition Q2** | 0.0036 | 0.0026 | 1.3900 | 0.1655 | -0.0015 | 0.0087 |
| **CIMD - Ethnocultural Composition Q3** | 0.0047 | 0.0038 | 1.2500 | 0.2100 | -0.0027 | 0.0121 |
| **CIMD - Ethnocultural Composition Q4** | 0.0009 | 0.0056 | 0.1600 | 0.8744 | -0.0100 | 0.0118 |
| **CIMD - Ethnocultural Composition Q5 (most deprived)** | -0.0088 | 0.0088 | -1.0000 | 0.3161 | -0.0261 | 0.0084 |
| **CIMD - Ethnocultural Composition Q1 (least deprived) (REF)** | 0.0000 | . | . | . | . | . |
| **CIMD -Situational Vulnerability Q2** | 0.0123 | 0.0040 | 3.1100 | 0.0019 | 0.0046 | 0.0201 |
| **CIMD - Situational Vulnerability Q3** | 0.0108 | 0.0045 | 2.4100 | 0.0160 | 0.0020 | 0.0196 |
| **CIMD -Situational Vulnerability Q4** | 0.0144 | 0.0044 | 3.2900 | 0.0010 | 0.0058 | 0.0230 |
| **CIMD -Situational Vulnerability Q5 (most deprived)** | 0.0226 | 0.0048 | 4.6900 | <.0001 | 0.0131 | 0.0320 |
| **CIMD - Situational Vulnerability Q1 (least deprived) (REF)** | 0.0000 | . | . | . | . | . |
| **Social Assistance (any in past 5 years)** | 0.0930 | 0.0039 | 23.5900 | <.0001 | 0.0853 | 0.1007 |
| **Social Assistance (none in past 5 years) (REF)** | 0.0000 | . | . | . | . | . |
| **Program of Study - French Immersion** | -0.0370 | 0.0028 | -13.2800 | <.0001 | -0.0424 | -0.0315 |
| **Program of Study - Other** | -0.0580 | 0.0209 | -2.7700 | 0.0056 | -0.0991 | -0.0170 |
| **Program of Study - French** | -0.0004 | 0.0127 | -0.0300 | 0.9765 | -0.0252 | 0.0245 |
| **Program of Study - English (REF)** | 0.0000 | . | . | . | . | . |
| **Household composition – Adults (age 22+) – No adults in household** | 0.0291 | 0.0130 | 2.2400 | 0.0250 | 0.0037 | 0.0545 |
| **Household composition – Adults (age 22+) – One adult in household** | 0.0405 | 0.0030 | 13.6700 | <.0001 | 0.0347 | 0.0463 |
| **Household composition – Adults (age 22+) – More than one adult in household (REF)** | 0.0000 | . | . | . | . | . |
| **Household composition – Children (age 21 or under) – Student is only child in household** | 0.0028 | 0.0027 | 1.0400 | 0.2965 | -0.0025 | 0.0081 |
| **Household composition – Children (age 21 or under) – Other children in household (REF)** | 0.0000 | . | . | . | . | . |
| **Recent immigrant** | -0.0158 | 0.0069 | -2.3000 | 0.0217 | -0.0293 | -0.0023 |
| **Not a recent immigrant (REF)** | 0.0000 | . | . | . | . | . |

**Supplementary Table S6d. GLM regression estimates – Proportion of language courses failed (score < 60%) for grades 9-12 (AY 2017 – 2020)**

| **Parameter** | **Estimate** | **Standard**  **Error** | **t Value** | **Pr > \|t\|** | **95% Confidence Limits** | |
| --- | --- | --- | --- | --- | --- | --- |
| **Intercept** | 0.0578 | 0.0147 | 3.9300 | <.0001 | 0.0290 | 0.0866 |
| **Treated ADHD** | 0.0340 | 0.0035 | 9.8400 | <.0001 | 0.0272 | 0.0408 |
| **Untreated ADHD** | 0.0533 | 0.0024 | 22.5500 | <.0001 | 0.0487 | 0.0580 |
| **No ADHD (REF)** | 0.0000 | . | . | . | . | . |
| **Age** | -0.0015 | 0.0006 | -2.3700 | 0.0178 | -0.0028 | -0.0003 |
| **Male** | 0.0302 | 0.0016 | 19.1700 | <.0001 | 0.0271 | 0.0333 |
| **Female (REF)** | 0.0000 | . | . | . | . | . |
| **Household income quintile Q2** | -0.0119 | 0.0030 | -3.9800 | <.0001 | -0.0178 | -0.0060 |
| **Household income quintile Q3** | -0.0117 | 0.0032 | -3.6000 | 0.0003 | -0.0180 | -0.0053 |
| **Household income quintile Q4** | -0.0133 | 0.0035 | -3.8200 | 0.0001 | -0.0201 | -0.0065 |
| **Household income quintile Q5 (highest income)** | -0.0180 | 0.0038 | -4.7600 | <.0001 | -0.0255 | -0.0106 |
| **Household income quintile Q1 (lowest income) (REF)** | 0.0000 | . | . | . | . | . |
| **NB Health Zone 2** | -0.0076 | 0.0023 | -3.3100 | 0.0009 | -0.0121 | -0.0031 |
| **NB Health Zone 3** | -0.0167 | 0.0023 | -7.3800 | <.0001 | -0.0212 | -0.0123 |
| **NB Health Zone 4** | -0.0355 | 0.0039 | -9.0800 | <.0001 | -0.0432 | -0.0278 |
| **NB Health Zone 5** | -0.0006 | 0.0050 | -0.1200 | 0.9027 | -0.0104 | 0.0092 |
| **NB Health Zone 6** | -0.0246 | 0.0035 | -6.9400 | <.0001 | -0.0315 | -0.0176 |
| **NB Health Zone 7** | 0.0051 | 0.0037 | 1.3800 | 0.1684 | -0.0022 | 0.0124 |
| **NB Health Zone 1 (REF)** | 0.0000 | . | . | . | . | . |
| **Comorbid conditions - Mood & anxiety disorders (yes)** | 0.0308 | 0.0033 | 9.3600 | <.0001 | 0.0243 | 0.0372 |
| **Comorbid conditions - Mood & anxiety disorders (no) (REF)** | 0.0000 | . | . | . | . | . |
| **Comorbid conditions – One or more of: asthma, diabetes, epilepsy, schizophrenia (yes)** | -0.0143 | 0.0092 | -1.5500 | 0.1217 | -0.0323 | 0.0038 |
| **Comorbid conditions – One or more of: asthma, diabetes, epilepsy, schizophrenia (no) (REF)** | 0.0000 | . | . | . | . | . |
| **Select medications (one or more)** | 0.0271 | 0.0037 | 7.4000 | <.0001 | 0.0199 | 0.0343 |
| **Select medications (none) (REF)** | 0.0000 | . | . | . | . | . |
| **School District - Anglophone** | -0.0004 | 0.0090 | -0.0400 | 0.9677 | -0.0181 | 0.0173 |
| **School District – Francophone (REF)** | 0.0000 | . | . | . | . | . |
| **CIMD - Residential Instability Q2** | -0.0009 | 0.0023 | -0.3900 | 0.6971 | -0.0054 | 0.0036 |
| **CIMD - Residential Instability Q3** | -0.0028 | 0.0025 | -1.1400 | 0.2530 | -0.0077 | 0.0020 |
| **CIMD - Residential Instability Q4** | 0.0057 | 0.0029 | 1.9900 | 0.0467 | 0.0001 | 0.0114 |
| **CIMD – Residential Instability Q5 (most deprived)** | 0.0092 | 0.0040 | 2.3200 | 0.0206 | 0.0014 | 0.0170 |
| **CIMD - Residential Instability Q1 (least deprived) (REF)** | 0.0000 | . | . | . | . | . |
| **CIMD - Economic Dependency Q2** | 0.0085 | 0.0029 | 2.9100 | 0.0036 | 0.0028 | 0.0142 |
| **CIMD - Economic Dependency Q3** | 0.0062 | 0.0029 | 2.1300 | 0.0328 | 0.0005 | 0.0119 |
| **CIMD - Economic Dependency Q4** | 0.0085 | 0.0030 | 2.8100 | 0.0050 | 0.0026 | 0.0145 |
| **CIMD - Economic Dependency Q5 (most deprived)** | -0.0058 | 0.0031 | -1.8900 | 0.0591 | -0.0119 | 0.0002 |
| **CIMD - Economic Dependency Q1 (least deprived) (REF)** | 0.0000 | . | . | . | . | . |
| **CIMD - Ethnocultural Composition Q2** | 0.0048 | 0.0018 | 2.6700 | 0.0076 | 0.0013 | 0.0083 |
| **CIMD - Ethnocultural Composition Q3** | 0.0040 | 0.0026 | 1.5600 | 0.1197 | -0.0010 | 0.0091 |
| **CIMD - Ethnocultural Composition Q4** | -0.0020 | 0.0039 | -0.5100 | 0.6104 | -0.0095 | 0.0056 |
| **CIMD - Ethnocultural Composition Q5 (most deprived)** | -0.0151 | 0.0061 | -2.4700 | 0.0135 | -0.0270 | -0.0031 |
| **CIMD - Ethnocultural Composition Q1 (least deprived) (REF)** | 0.0000 | . | . | . | . | . |
| **CIMD -Situational Vulnerability Q2** | 0.0056 | 0.0027 | 2.0300 | 0.0422 | 0.0002 | 0.0110 |
| **CIMD - Situational Vulnerability Q3** | 0.0131 | 0.0031 | 4.2300 | <.0001 | 0.0070 | 0.0192 |
| **CIMD -Situational Vulnerability Q4** | 0.0200 | 0.0030 | 6.6100 | <.0001 | 0.0141 | 0.0259 |
| **CIMD -Situational Vulnerability Q5 (most deprived)** | 0.0332 | 0.0033 | 10.0000 | <.0001 | 0.0267 | 0.0397 |
| **CIMD - Situational Vulnerability Q1 (least deprived) (REF)** | 0.0000 | . | . | . | . | . |
| **Social Assistance (any in past 5 years)** | 0.0832 | 0.0027 | 31.1300 | <.0001 | 0.0779 | 0.0884 |
| **Social Assistance (none in past 5 years) (REF)** | 0.0000 | . | . | . | . | . |
| **Program of Study - French Immersion** | -0.0235 | 0.0019 | -12.1700 | <.0001 | -0.0273 | -0.0197 |
| **Program of Study - Other** | -0.0185 | 0.0133 | -1.3800 | 0.1665 | -0.0446 | 0.0077 |
| **Program of Study - French** | -0.0003 | 0.0090 | -0.0400 | 0.9698 | -0.0179 | 0.0173 |
| **Program of Study - English (REF)** | 0.0000 | . | . | . | . | . |
| **Household composition – Adults (age 22+) – No adults in household** | 0.0481 | 0.0075 | 6.4200 | <.0001 | 0.0334 | 0.0628 |
| **Household composition – Adults (age 22+) – One adult in household** | 0.0260 | 0.0020 | 12.8400 | <.0001 | 0.0221 | 0.0300 |
| **Household composition – Adults (age 22+) – More than one adult in household (REF)** | 0.0000 | . | . | . | . | . |
| **Household composition – Children (age 21 or under) – Student is only child in household** | -0.0011 | 0.0018 | -0.6200 | 0.5383 | -0.0047 | 0.0025 |
| **Household composition – Children (age 21 or under) – Other children in household (REF)** | 0.0000 | . | . | . | . | . |
| **Recent immigrant** | -0.0295 | 0.0049 | -5.9600 | <.0001 | -0.0392 | -0.0198 |
| **Not a recent immigrant (REF)** | 0.0000 | . | . | . | . | . |

**Supplementary Table S6e. GLM regression estimates – Proportion of overall courses failed (score < 60%) for grades 9-12 (AY 2017 – 2020) (Untreated group as reference)**

| **Parameter** | **Estimate** | **Standard**  **Error** | **t Value** | **Pr > \|t\|** | **95% Confidence Limits** | |
| --- | --- | --- | --- | --- | --- | --- |
| **Intercept** | 0.1672 | 0.0412 | 4.0500 | <.0001 | 0.0863 | 0.2480 |
| **Treated ADHD** | -0.0259 | 0.0055 | -4.7200 | <.0001 | -0.0366 | -0.0151 |
| **Untreated ADHD (REF)** | 0.0000 | . | . | . | . | . |
| **Age** | -0.0050 | 0.0019 | -2.6200 | 0.0089 | -0.0087 | -0.0013 |
| **Male** | 0.0285 | 0.0050 | 5.6800 | <.0001 | 0.0187 | 0.0384 |
| **Female (REF)** | 0.0000 | . | . | . | . | . |
| **Household income quintile Q2** | 0.0004 | 0.0085 | 0.0400 | 0.9665 | -0.0164 | 0.0171 |
| **Household income quintile Q3** | -0.0052 | 0.0095 | -0.5500 | 0.5838 | -0.0238 | 0.0134 |
| **Household income quintile Q4** | -0.0268 | 0.0102 | -2.6200 | 0.0088 | -0.0468 | -0.0067 |
| **Household income quintile Q5 (highest income)** | -0.0333 | 0.0111 | -2.9800 | 0.0029 | -0.0551 | -0.0114 |
| **Household income quintile Q1 (lowest income) (REF)** | 0.0000 | . | . | . | . | . |
| **NB Health Zone 2** | -0.0088 | 0.0065 | -1.3600 | 0.1727 | -0.0215 | 0.0039 |
| **NB Health Zone 3** | -0.0312 | 0.0065 | -4.7800 | <.0001 | -0.0440 | -0.0184 |
| **NB Health Zone 4** | -0.0543 | 0.0154 | -3.5200 | 0.0004 | -0.0845 | -0.0240 |
| **NB Health Zone 5** | -0.0013 | 0.0147 | -0.0900 | 0.9290 | -0.0302 | 0.0275 |
| **NB Health Zone 6** | -0.0343 | 0.0117 | -2.9200 | 0.0035 | -0.0572 | -0.0113 |
| **NB Health Zone 7** | -0.0011 | 0.0117 | -0.0900 | 0.9280 | -0.0239 | 0.0218 |
| **NB Health Zone 1 (REF)** | 0.0000 | . | . | . | . | . |
| **Comorbid conditions - Mood & anxiety disorders (yes)** | 0.0447 | 0.0077 | 5.8100 | <.0001 | 0.0296 | 0.0598 |
| **Comorbid conditions - Mood & anxiety disorders (no) (REF)** | 0.0000 | . | . | . | . | . |
| **Comorbid conditions – One or more of: asthma, diabetes, epilepsy, schizophrenia (yes)** | -0.0231 | 0.0221 | -1.0500 | 0.2955 | -0.0663 | 0.0202 |
| **Comorbid conditions – One or more of: asthma, diabetes, epilepsy, schizophrenia (no) (REF)** | 0.0000 | . | . | . | . | . |
| **Select medications (one or more)** | 0.0232 | 0.0067 | 3.4800 | 0.0005 | 0.0101 | 0.0363 |
| **Select medications (none) (REF)** | 0.0000 | . | . | . | . | . |
| **School District - Anglophone** | 0.0391 | 0.0250 | 1.5700 | 0.1172 | -0.0098 | 0.0881 |
| **School District – Francophone (REF)** | 0.0000 | . | . | . | . | . |
| **CIMD - Residential Instability Q2** | -0.0053 | 0.0071 | -0.7500 | 0.4505 | -0.0192 | 0.0085 |
| **CIMD - Residential Instability Q3** | -0.0130 | 0.0076 | -1.7200 | 0.0852 | -0.0278 | 0.0018 |
| **CIMD - Residential Instability Q4** | -0.0043 | 0.0085 | -0.5100 | 0.6111 | -0.0210 | 0.0124 |
| **CIMD – Residential Instability Q5 (most deprived)** | 0.0075 | 0.0112 | 0.6700 | 0.5037 | -0.0144 | 0.0294 |
| **CIMD - Residential Instability Q1 (least deprived) (REF)** | 0.0000 | . | . | . | . | . |
| **CIMD - Economic Dependency Q2** | 0.0004 | 0.0087 | 0.0400 | 0.9664 | -0.0166 | 0.0174 |
| **CIMD - Economic Dependency Q3** | 0.0168 | 0.0087 | 1.9200 | 0.0544 | -0.0003 | 0.0338 |
| **CIMD - Economic Dependency Q4** | 0.0099 | 0.0090 | 1.1100 | 0.2691 | -0.0077 | 0.0275 |
| **CIMD - Economic Dependency Q5 (most deprived)** | -0.0059 | 0.0092 | -0.6400 | 0.5194 | -0.0240 | 0.0121 |
| **CIMD - Economic Dependency Q1 (least deprived) (REF)** | 0.0000 | . | . | . | . | . |
| **CIMD - Ethnocultural Composition Q2** | 0.0135 | 0.0054 | 2.5100 | 0.0121 | 0.0029 | 0.0240 |
| **CIMD - Ethnocultural Composition Q3** | 0.0067 | 0.0077 | 0.8700 | 0.3866 | -0.0084 | 0.0218 |
| **CIMD - Ethnocultural Composition Q4** | 0.0052 | 0.0116 | 0.4500 | 0.6536 | -0.0176 | 0.0281 |
| **CIMD - Ethnocultural Composition Q5 (most deprived)** | 0.0091 | 0.0163 | 0.5600 | 0.5784 | -0.0229 | 0.0411 |
| **CIMD - Ethnocultural Composition Q1 (least deprived) (REF)** | 0.0000 | . | . | . | . | . |
| **CIMD -Situational Vulnerability Q2** | 0.0076 | 0.0083 | 0.9200 | 0.3562 | -0.0086 | 0.0238 |
| **CIMD - Situational Vulnerability Q3** | 0.0082 | 0.0094 | 0.8700 | 0.3835 | -0.0103 | 0.0267 |
| **CIMD -Situational Vulnerability Q4** | 0.0298 | 0.0090 | 3.3000 | 0.0010 | 0.0121 | 0.0476 |
| **CIMD -Situational Vulnerability Q5 (most deprived)** | 0.0115 | 0.0098 | 1.1700 | 0.2421 | -0.0077 | 0.0307 |
| **CIMD - Situational Vulnerability Q1 (least deprived) (REF)** | 0.0000 | . | . | . | . | . |
| **Social Assistance (any in past 5 years)** | 0.0747 | 0.0063 | 11.8400 | <.0001 | 0.0624 | 0.0871 |
| **Social Assistance (none in past 5 years) (REF)** | 0.0000 | . | . | . | . | . |
| **Program of Study - French Immersion** | -0.0378 | 0.0062 | -6.1300 | <.0001 | -0.0498 | -0.0257 |
| **Program of Study - Other** | 0.0808 | 0.0325 | 2.4900 | 0.0129 | 0.0171 | 0.1445 |
| **Program of Study - French** | -0.0041 | 0.0245 | -0.1700 | 0.8677 | -0.0520 | 0.0439 |
| **Program of Study - English (REF)** | 0.0000 | . | . | . | . | . |
| **Household composition – Adults (age 22+) – No adults in household** | 0.0417 | 0.0151 | 2.7600 | 0.0057 | 0.0121 | 0.0713 |
| **Household composition – Adults (age 22+) – One adult in household** | 0.0405 | 0.0056 | 7.2800 | <.0001 | 0.0296 | 0.0514 |
| **Household composition – Adults (age 22+) – More than one adult in household (REF)** | 0.0000 | . | . | . | . | . |
| **Household composition – Children (age 21 or under) – Student is only child in household** | 0.0111 | 0.0053 | 2.1100 | 0.0349 | 0.0008 | 0.0214 |
| **Household composition – Children (age 21 or under) – Other children in household (REF)** | 0.0000 | . | . | . | . | . |
| **Recent immigrant** | -0.0456 | 0.0403 | -1.1300 | 0.2575 | -0.1245 | 0.0333 |
| **Not a recent immigrant (REF)** | 0.0000 | . | . | . | . | . |

**Supplementary Table S6f. GLM regression estimates – Proportion of STEM courses failed (score < 60%) for grades 9-12 (AY 2017 – 2020) (Untreated group as reference)**

| **Parameter** | **Estimate** | **Standard**  **Error** | **t Value** | **Pr > \|t\|** | **95% Confidence Limits** | |
| --- | --- | --- | --- | --- | --- | --- |
| **Intercept** | 0.2908 | 0.1363 | 2.1300 | 0.0329 | 0.0236 | 0.5581 |
| **Treated ADHD** | -0.0161 | 0.0076 | -2.1100 | 0.0348 | -0.0310 | -0.0012 |
| **Untreated ADHD (REF)** | 0.0000 | . | . | . | . | . |
| **Age** | -0.0019 | 0.0027 | -0.7000 | 0.4829 | -0.0071 | 0.0033 |
| **Male** | 0.0170 | 0.0069 | 2.4600 | 0.0139 | 0.0035 | 0.0305 |
| **Female (REF)** | 0.0000 | . | . | . | . | . |
| **Household income quintile Q2** | -0.0120 | 0.0119 | -1.0100 | 0.3145 | -0.0353 | 0.0114 |
| **Household income quintile Q3** | -0.0268 | 0.0134 | -2.0000 | 0.0452 | -0.0529 | -0.0006 |
| **Household income quintile Q4** | -0.0441 | 0.0143 | -3.0800 | 0.0021 | -0.0722 | -0.0160 |
| **Household income quintile Q5 (highest income)** | -0.0498 | 0.0156 | -3.1900 | 0.0014 | -0.0805 | -0.0192 |
| **Household income quintile Q1 (lowest income) (REF)** | 0.0000 | . | . | . | . | . |
| **NB Health Zone 2** | 0.0003 | 0.0086 | 0.0300 | 0.9765 | -0.0167 | 0.0172 |
| **NB Health Zone 3** | -0.0241 | 0.0088 | -2.7600 | 0.0058 | -0.0413 | -0.0070 |
| **NB Health Zone 4** | -0.0154 | 0.0370 | -0.4200 | 0.6772 | -0.0880 | 0.0572 |
| **NB Health Zone 5** | -0.0029 | 0.0216 | -0.1400 | 0.8922 | -0.0454 | 0.0395 |
| **NB Health Zone 6** | -0.0619 | 0.0214 | -2.9000 | 0.0038 | -0.1038 | -0.0200 |
| **NB Health Zone 7** | -0.0103 | 0.0161 | -0.6400 | 0.5201 | -0.0418 | 0.0212 |
| **NB Health Zone 1 (REF)** | 0.0000 | . | . | . | . | . |
| **Comorbid conditions - Mood & anxiety disorders (yes)** | 0.0353 | 0.0105 | 3.3800 | 0.0007 | 0.0148 | 0.0558 |
| **Comorbid conditions - Mood & anxiety disorders (no) (REF)** | 0.0000 | . | . | . | . | . |
| **Comorbid conditions – One or more of: asthma, diabetes, epilepsy, schizophrenia (yes)** | -0.0107 | 0.0309 | -0.3500 | 0.7297 | -0.0713 | 0.0499 |
| **Comorbid conditions – One or more of: asthma, diabetes, epilepsy, schizophrenia (no) (REF)** | 0.0000 | . | . | . | . | . |
| **Select medications (one or more)** | 0.0035 | 0.0094 | 0.3700 | 0.7080 | -0.0148 | 0.0219 |
| **Select medications (none) (REF)** | 0.0000 | . | . | . | . | . |
| **School District - Anglophone** | -0.0885 | 0.1268 | -0.7000 | 0.4855 | -0.3371 | 0.1601 |
| **School District – Francophone (REF)** | 0.0000 | . | . | . | . | . |
| **CIMD - Residential Instability Q2** | -0.0099 | 0.0097 | -1.0200 | 0.3071 | -0.0288 | 0.0091 |
| **CIMD - Residential Instability Q3** | -0.0172 | 0.0105 | -1.6400 | 0.1019 | -0.0379 | 0.0034 |
| **CIMD - Residential Instability Q4** | -0.0142 | 0.0117 | -1.2200 | 0.2236 | -0.0371 | 0.0087 |
| **CIMD – Residential Instability Q5 (most deprived)** | 0.0056 | 0.0152 | 0.3700 | 0.7137 | -0.0242 | 0.0354 |
| **CIMD - Residential Instability Q1 (least deprived) (REF)** | 0.0000 | . | . | . | . | . |
| **CIMD - Economic Dependency Q2** | 0.0169 | 0.0117 | 1.4500 | 0.1462 | -0.0059 | 0.0398 |
| **CIMD - Economic Dependency Q3** | 0.0238 | 0.0117 | 2.0400 | 0.0415 | 0.0009 | 0.0467 |
| **CIMD - Economic Dependency Q4** | 0.0144 | 0.0121 | 1.2000 | 0.2318 | -0.0092 | 0.0381 |
| **CIMD - Economic Dependency Q5 (most deprived)** | -0.0016 | 0.0125 | -0.1300 | 0.8955 | -0.0261 | 0.0228 |
| **CIMD - Economic Dependency Q1 (least deprived) (REF)** | 0.0000 | . | . | . | . | . |
| **CIMD - Ethnocultural Composition Q2** | -0.0044 | 0.0075 | -0.5800 | 0.5587 | -0.0190 | 0.0103 |
| **CIMD - Ethnocultural Composition Q3** | 0.0049 | 0.0106 | 0.4700 | 0.6419 | -0.0158 | 0.0257 |
| **CIMD - Ethnocultural Composition Q4** | 0.0139 | 0.0157 | 0.8900 | 0.3759 | -0.0169 | 0.0447 |
| **CIMD - Ethnocultural Composition Q5 (most deprived)** | -0.0013 | 0.0215 | -0.0600 | 0.9530 | -0.0435 | 0.0409 |
| **CIMD - Ethnocultural Composition Q1 (least deprived) (REF)** | 0.0000 | . | . | . | . | . |
| **CIMD -Situational Vulnerability Q2** | 0.0186 | 0.0110 | 1.6900 | 0.0906 | -0.0029 | 0.0402 |
| **CIMD - Situational Vulnerability Q3** | 0.0127 | 0.0127 | 1.0000 | 0.3188 | -0.0123 | 0.0377 |
| **CIMD -Situational Vulnerability Q4** | 0.0166 | 0.0122 | 1.3600 | 0.1728 | -0.0073 | 0.0406 |
| **CIMD -Situational Vulnerability Q5 (most deprived)** | -0.0026 | 0.0135 | -0.1900 | 0.8485 | -0.0290 | 0.0238 |
| **CIMD - Situational Vulnerability Q1 (least deprived) (REF)** | 0.0000 | . | . | . | . | . |
| **Social Assistance (any in past 5 years)** | 0.0718 | 0.0086 | 8.3100 | <.0001 | 0.0548 | 0.0887 |
| **Social Assistance (none in past 5 years) (REF)** | 0.0000 | . | . | . | . | . |
| **Program of Study - French Immersion** | -0.0373 | 0.0079 | -4.7500 | <.0001 | -0.0527 | -0.0219 |
| **Program of Study - Other** | 0.2461 | 0.0797 | 3.0900 | 0.0020 | 0.0898 | 0.4024 |
| **Program of Study - French** | -0.0093 | 0.0339 | -0.2800 | 0.7829 | -0.0757 | 0.0571 |
| **Program of Study - English (REF)** | 0.0000 | . | . | . | . | . |
| **Household composition – Adults (age 22+) – No adults in household** | 0.0594 | 0.0233 | 2.5400 | 0.0110 | 0.0136 | 0.1052 |
| **Household composition – Adults (age 22+) – One adult in household** | 0.0417 | 0.0077 | 5.3900 | <.0001 | 0.0266 | 0.0569 |
| **Household composition – Adults (age 22+) – More than one adult in household (REF)** | 0.0000 | . | . | . | . | . |
| **Household composition – Children (age 21 or under) – Student is only child in household** | 0.0132 | 0.0073 | 1.8200 | 0.0692 | -0.0010 | 0.0275 |
| **Household composition – Children (age 21 or under) – Other children in household (REF)** | 0.0000 | . | . | . | . | . |
| **Recent immigrant** | -0.0589 | 0.0530 | -1.1100 | 0.2669 | -0.1628 | 0.0451 |
| **Not a recent immigrant (REF)** | 0.0000 | . | . | . | . | . |

**Supplementary Table S6g. GLM regression estimates – Proportion of math courses failed (score < 60%) for grades 9-12 (AY 2017 – 2020) (Untreated group as reference)**

| **Parameter** | **Estimate** | **Standard**  **Error** | **t Value** | **Pr > \|t\|** | **95% Confidence Limits** | |
| --- | --- | --- | --- | --- | --- | --- |
| **Intercept** | 0.4713 | 0.1738 | 2.7100 | 0.0067 | 0.1307 | 0.8119 |
| **Treated ADHD** | -0.0163 | 0.0088 | -1.8500 | 0.0249 | -0.0336 | -0.0190 |
| **Untreated ADHD (REF)** | 0.0000 | . | . | . | . | . |
| **Age** | -0.0068 | 0.0032 | -2.1200 | 0.0342 | -0.0131 | -0.0005 |
| **Male** | 0.0155 | 0.0080 | 1.9300 | 0.0533 | -0.0002 | 0.0312 |
| **Female (REF)** | 0.0000 | . | . | . | . | . |
| **Household income quintile Q2** | 0.0019 | 0.0139 | 0.1400 | 0.8895 | -0.0253 | 0.0291 |
| **Household income quintile Q3** | -0.0268 | 0.0155 | -1.7300 | 0.0835 | -0.0572 | 0.0035 |
| **Household income quintile Q4** | -0.0364 | 0.0166 | -2.1900 | 0.0282 | -0.0690 | -0.0039 |
| **Household income quintile Q5 (highest income)** | -0.0466 | 0.0181 | -2.5800 | 0.0099 | -0.0820 | -0.0112 |
| **Household income quintile Q1 (lowest income) (REF)** | 0.0000 | . | . | . | . | . |
| **NB Health Zone 2** | 0.0001 | 0.0101 | 0.0100 | 0.9894 | -0.0196 | 0.0199 |
| **NB Health Zone 3** | -0.0198 | 0.0102 | -1.9400 | 0.0525 | -0.0398 | 0.0002 |
| **NB Health Zone 4** | -0.0104 | 0.0440 | -0.2400 | 0.8122 | -0.0966 | 0.0757 |
| **NB Health Zone 5** | -0.0261 | 0.0251 | -1.0400 | 0.2991 | -0.0753 | 0.0232 |
| **NB Health Zone 6** | -0.0876 | 0.0249 | -3.5100 | 0.0005 | -0.1365 | -0.0387 |
| **NB Health Zone 7** | -0.0236 | 0.0187 | -1.2700 | 0.2056 | -0.0602 | 0.0130 |
| **NB Health Zone 1 (REF)** | 0.0000 | . | . | . | . | . |
| **Comorbid conditions - Mood & anxiety disorders (yes)** | 0.0305 | 0.0122 | 2.5100 | 0.0121 | 0.0067 | 0.0544 |
| **Comorbid conditions - Mood & anxiety disorders (no) (REF)** | 0.0000 | . | . | . | . | . |
| **Comorbid conditions – One or more of: asthma, diabetes, epilepsy, schizophrenia (yes)** | -0.0266 | 0.0374 | -0.7100 | 0.4779 | -0.1000 | 0.0468 |
| **Comorbid conditions – One or more of: asthma, diabetes, epilepsy, schizophrenia (no) (REF)** | 0.0000 | . | . | . | . | . |
| **Select medications (one or more)** | -0.0121 | 0.0109 | -1.1000 | 0.2696 | -0.0335 | 0.0094 |
| **Select medications (none) (REF)** | 0.0000 | . | . | . | . | . |
| **School District - Anglophone** | -0.1952 | 0.1631 | -1.2000 | 0.2316 | -0.5150 | 0.1246 |
| **School District – Francophone (REF)** | 0.0000 | . | . | . | . | . |
| **CIMD - Residential Instability Q2** | -0.0172 | 0.0111 | -1.5400 | 0.1231 | -0.0390 | 0.0047 |
| **CIMD - Residential Instability Q3** | -0.0377 | 0.0122 | -3.0900 | 0.0020 | -0.0615 | -0.0138 |
| **CIMD - Residential Instability Q4** | -0.0145 | 0.0135 | -1.0800 | 0.2810 | -0.0409 | 0.0119 |
| **CIMD – Residential Instability Q5 (most deprived)** | 0.0011 | 0.0176 | 0.0600 | 0.9488 | -0.0333 | 0.0356 |
| **CIMD - Residential Instability Q1 (least deprived) (REF)** | 0.0000 | . | . | . | . | . |
| **CIMD - Economic Dependency Q2** | 0.0241 | 0.0134 | 1.8000 | 0.0723 | -0.0022 | 0.0504 |
| **CIMD - Economic Dependency Q3** | 0.0287 | 0.0135 | 2.1300 | 0.0335 | 0.0022 | 0.0551 |
| **CIMD - Economic Dependency Q4** | 0.0280 | 0.0139 | 2.0200 | 0.0437 | 0.0008 | 0.0553 |
| **CIMD - Economic Dependency Q5 (most deprived)** | 0.0104 | 0.0144 | 0.7300 | 0.4672 | -0.0177 | 0.0386 |
| **CIMD - Economic Dependency Q1 (least deprived) (REF)** | 0.0000 | . | . | . | . | . |
| **CIMD - Ethnocultural Composition Q2** | -0.0078 | 0.0086 | -0.9000 | 0.3658 | -0.0247 | 0.0091 |
| **CIMD - Ethnocultural Composition Q3** | 0.0164 | 0.0122 | 1.3400 | 0.1788 | -0.0075 | 0.0404 |
| **CIMD - Ethnocultural Composition Q4** | 0.0187 | 0.0181 | 1.0300 | 0.3027 | -0.0168 | 0.0541 |
| **CIMD - Ethnocultural Composition Q5 (most deprived)** | -0.0253 | 0.0257 | -0.9800 | 0.3257 | -0.0757 | 0.0252 |
| **CIMD - Ethnocultural Composition Q1 (least deprived) (REF)** | 0.0000 | . | . | . | . | . |
| **CIMD -Situational Vulnerability Q2** | 0.0175 | 0.0127 | 1.3800 | 0.1665 | -0.0073 | 0.0424 |
| **CIMD - Situational Vulnerability Q3** | 0.0067 | 0.0146 | 0.4600 | 0.6477 | -0.0220 | 0.0354 |
| **CIMD -Situational Vulnerability Q4** | 0.0230 | 0.0141 | 1.6300 | 0.1027 | -0.0046 | 0.0506 |
| **CIMD -Situational Vulnerability Q5 (most deprived)** | -0.0102 | 0.0156 | -0.6600 | 0.5109 | -0.0408 | 0.0203 |
| **CIMD - Situational Vulnerability Q1 (least deprived) (REF)** | 0.0000 | . | . | . | . | . |
| **Social Assistance (any in past 5 years)** | 0.0771 | 0.0101 | 7.6100 | <.0001 | 0.0573 | 0.0970 |
| **Social Assistance (none in past 5 years) (REF)** | 0.0000 | . | . | . | . | . |
| **Program of Study - French Immersion** | -0.0377 | 0.0090 | -4.1800 | <.0001 | -0.0554 | -0.0200 |
| **Program of Study - Other** | 0.2621 | 0.1271 | 2.0600 | 0.0392 | 0.0130 | 0.5113 |
| **Program of Study - French** | -0.0013 | 0.0386 | -0.0300 | 0.9738 | -0.0769 | 0.0744 |
| **Program of Study - English (REF)** | 0.0000 | . | . | . | . | . |
| **Household composition – Adults (age 22+) – No adults in household** | 0.1049 | 0.0320 | 3.2700 | 0.0011 | 0.0421 | 0.1678 |
| **Household composition – Adults (age 22+) – One adult in household** | 0.0352 | 0.0089 | 3.9500 | <.0001 | 0.0178 | 0.0527 |
| **Household composition – Adults (age 22+) – More than one adult in household (REF)** | 0.0000 | . | . | . | . | . |
| **Household composition – Children (age 21 or under) – Student is only child in household** | 0.0176 | 0.0085 | 2.0800 | 0.0380 | 0.0010 | 0.0342 |
| **Household composition – Children (age 21 or under) – Other children in household (REF)** | 0.0000 | . | . | . | . | . |
| **Recent immigrant** | -0.0614 | 0.0600 | -1.0200 | 0.3066 | -0.1791 | 0.0563 |
| **Not a recent immigrant (REF)** | 0.0000 | . | . | . | . | . |

**Supplementary Table S6h. GLM regression estimates – Proportion of language courses failed (score < 60%) for grades 9-12 (AY 2017 – 2020) (Untreated group as reference)**

| **Parameter** | **Estimate** | **Standard**  **Error** | **t Value** | **Pr > \|t\|** | **95% Confidence Limits** | |
| --- | --- | --- | --- | --- | --- | --- |
| **Intercept** | 0.2319 | 0.0476 | 4.8700 | <.0001 | 0.1386 | 0.3252 |
| **Treated ADHD** | -0.0232 | 0.0063 | -3.6900 | 0.0002 | -0.0356 | -0.0109 |
| **Untreated ADHD (REF)** | 0.0000 | . | . | . | . | . |
| **Age** | -0.0120 | 0.0022 | -5.4000 | <.0001 | -0.0163 | -0.0076 |
| **Male** | 0.0313 | 0.0057 | 5.4400 | <.0001 | 0.0200 | 0.0425 |
| **Female (REF)** | 0.0000 | . | . | . | . | . |
| **Household income quintile Q2** | 0.0014 | 0.0098 | 0.1400 | 0.8848 | -0.0178 | 0.0207 |
| **Household income quintile Q3** | -0.0024 | 0.0109 | -0.2200 | 0.8265 | -0.0238 | 0.0190 |
| **Household income quintile Q4** | -0.0205 | 0.0117 | -1.7500 | 0.0805 | -0.0434 | 0.0025 |
| **Household income quintile Q5 (highest income)** | -0.0189 | 0.0128 | -1.4800 | 0.1383 | -0.0440 | 0.0061 |
| **Household income quintile Q1 (lowest income) (REF)** | 0.0000 | . | . | . | . | . |
| **NB Health Zone 2** | -0.0032 | 0.0074 | -0.4300 | 0.6641 | -0.0178 | 0.0113 |
| **NB Health Zone 3** | -0.0192 | 0.0075 | -2.5800 | 0.0100 | -0.0339 | -0.0046 |
| **NB Health Zone 4** | -0.0288 | 0.0180 | -1.6000 | 0.1096 | -0.0640 | 0.0065 |
| **NB Health Zone 5** | 0.0080 | 0.0167 | 0.4800 | 0.6325 | -0.0247 | 0.0406 |
| **NB Health Zone 6** | -0.0168 | 0.0138 | -1.2200 | 0.2236 | -0.0438 | 0.0102 |
| **NB Health Zone 7** | 0.0247 | 0.0134 | 1.8500 | 0.0643 | -0.0015 | 0.0509 |
| **NB Health Zone 1 (REF)** | 0.0000 | . | . | . | . | . |
| **Comorbid conditions - Mood & anxiety disorders (yes)** | 0.0274 | 0.0089 | 3.0900 | 0.0020 | 0.0100 | 0.0448 |
| **Comorbid conditions - Mood & anxiety disorders (no) (REF)** | 0.0000 | . | . | . | . | . |
| **Comorbid conditions – One or more of: asthma, diabetes, epilepsy, schizophrenia (yes)** | -0.0220 | 0.0257 | -0.8600 | 0.3917 | -0.0724 | 0.0284 |
| **Comorbid conditions – One or more of: asthma, diabetes, epilepsy, schizophrenia (no) (REF)** | 0.0000 | . | . | . | . | . |
| **Select medications (one or more)** | 0.0222 | 0.0077 | 2.8700 | 0.0041 | 0.0070 | 0.0373 |
| **Select medications (none) (REF)** | 0.0000 | . | . | . | . | . |
| **School District - Anglophone** | 0.0152 | 0.0289 | 0.5200 | 0.6000 | -0.0415 | 0.0718 |
| **School District – Francophone (REF)** | 0.0000 | . | . | . | . | . |
| **CIMD - Residential Instability Q2** | 0.0010 | 0.0080 | 0.1300 | 0.8971 | -0.0147 | 0.0168 |
| **CIMD - Residential Instability Q3** | -0.0154 | 0.0086 | -1.7800 | 0.0744 | -0.0323 | 0.0015 |
| **CIMD - Residential Instability Q4** | 0.0022 | 0.0097 | 0.2200 | 0.8234 | -0.0169 | 0.0212 |
| **CIMD – Residential Instability Q5 (most deprived)** | 0.0147 | 0.0128 | 1.1500 | 0.2514 | -0.0104 | 0.0398 |
| **CIMD - Residential Instability Q1 (least deprived) (REF)** | 0.0000 | . | . | . | . | . |
| **CIMD - Economic Dependency Q2** | 0.0174 | 0.0099 | 1.7500 | 0.0797 | -0.0021 | 0.0368 |
| **CIMD - Economic Dependency Q3** | 0.0204 | 0.0100 | 2.0500 | 0.0407 | 0.0009 | 0.0399 |
| **CIMD - Economic Dependency Q4** | 0.0188 | 0.0102 | 1.8300 | 0.0669 | -0.0013 | 0.0389 |
| **CIMD - Economic Dependency Q5 (most deprived)** | 0.0005 | 0.0105 | 0.0500 | 0.9597 | -0.0201 | 0.0211 |
| **CIMD - Economic Dependency Q1 (least deprived) (REF)** | 0.0000 | . | . | . | . | . |
| **CIMD - Ethnocultural Composition Q2** | 0.0154 | 0.0061 | 2.5100 | 0.0122 | 0.0033 | 0.0274 |
| **CIMD - Ethnocultural Composition Q3** | 0.0155 | 0.0088 | 1.7500 | 0.0801 | -0.0019 | 0.0328 |
| **CIMD - Ethnocultural Composition Q4** | 0.0202 | 0.0133 | 1.5200 | 0.1294 | -0.0059 | 0.0462 |
| **CIMD - Ethnocultural Composition Q5 (most deprived)** | -0.0064 | 0.0192 | -0.3400 | 0.7369 | -0.0440 | 0.0311 |
| **CIMD - Ethnocultural Composition Q1 (least deprived) (REF)** | 0.0000 | . | . | . | . | . |
| **CIMD -Situational Vulnerability Q2** | 0.0064 | 0.0094 | 0.6800 | 0.4934 | -0.0120 | 0.0248 |
| **CIMD - Situational Vulnerability Q3** | 0.0146 | 0.0107 | 1.3700 | 0.1721 | -0.0064 | 0.0356 |
| **CIMD -Situational Vulnerability Q4** | 0.0354 | 0.0103 | 3.4400 | 0.0006 | 0.0152 | 0.0556 |
| **CIMD -Situational Vulnerability Q5 (most deprived)** | 0.0276 | 0.0112 | 2.4700 | 0.0137 | 0.0057 | 0.0495 |
| **CIMD - Situational Vulnerability Q1 (least deprived) (REF)** | 0.0000 | . | . | . | . | . |
| **Social Assistance (any in past 5 years)** | 0.0707 | 0.0073 | 9.6300 | <.0001 | 0.0563 | 0.0851 |
| **Social Assistance (none in past 5 years) (REF)** | 0.0000 | . | . | . | . | . |
| **Program of Study - French Immersion** | -0.0111 | 0.0069 | -1.6000 | 0.1087 | -0.0246 | 0.0025 |
| **Program of Study - Other** | 0.1087 | 0.0419 | 2.6000 | 0.0094 | 0.0267 | 0.1908 |
| **Program of Study - French** | -0.0007 | 0.0281 | -0.0300 | 0.9791 | -0.0559 | 0.0544 |
| **Program of Study - English (REF)** | 0.0000 | . | . | . | . | . |
| **Household composition – Adults (age 22+) – No adults in household** | 0.0191 | 0.0196 | 0.9800 | 0.3291 | -0.0193 | 0.0575 |
| **Household composition – Adults (age 22+) – One adult in household** | 0.0319 | 0.0064 | 5.0000 | <.0001 | 0.0194 | 0.0444 |
| **Household composition – Adults (age 22+) – More than one adult in household (REF)** | 0.0000 | . | . | . | . | . |
| **Household composition – Children (age 21 or under) – Student is only child in household** | 0.0020 | 0.0060 | 0.3200 | 0.7461 | -0.0099 | 0.0138 |
| **Household composition – Children (age 21 or under) – Other children in household (REF)** | 0.0000 | . | . | . | . | . |
| **Recent immigrant** | -0.0729 | 0.0453 | -1.6100 | 0.1072 | -0.1616 | 0.0158 |
| **Not a recent immigrant (REF)** | 0.0000 | . | . | . | . | . |
