# Supplementary material for: Academic Outcomes in Primary and Secondary School Students Prescribed Long-Acting Stimulants for ADHD Management
Source: J Atten Disord. 2025 Oct 7;30(4):493–505. doi: 10.1177/10870547251378169 (PMC12953683; doi:10.1177/10870547251378169)
Supplement: sj-docx-7-jad-10.1177_10870547251378169 – Supplemental material for Academic Outcomes in Primary and Secondary School Students Prescribed Long-Acting Stimulants for ADHD Management [file sj-docx-7-jad-10.1177_10870547251378169.docx]

**Supplementary Table S7a. GLM regression estimates – Provincial assessment exams overall percentile rank for grades K-8 (AY 2017-2019)**

| **Parameter** | **Estimate** | **Standard**  **Error** | **t Value** | **Pr > \|t\|** | **95% Confidence Limits** | |
| --- | --- | --- | --- | --- | --- | --- |
| **Intercept** | 23.0360 | 6.1454 | 3.7500 | 0.0002 | 10.9905 | 35.0814 |
| **Treated ADHD** | -14.0176 | 0.7029 | -19.9400 | <.0001 | -15.3953 | -12.6399 |
| **Untreated ADHD** | -13.3508 | 0.6369 | -20.9600 | <.0001 | -14.5992 | -12.1024 |
| **No ADHD (REF)** | 0.0000 | . | . | . | . | . |
| **Age** | 0.0684 | 0.1008 | 0.6800 | 0.4972 | -0.1291 | 0.2659 |
| **Male** | -0.7497 | 0.4072 | -1.8400 | 0.0657 | -1.5479 | 0.0485 |
| **Female (REF)** | 0.0000 | . | . | . | . | . |
| **Household income quintile Q2** | 0.4084 | 0.7823 | 0.5200 | 0.6017 | -1.1250 | 1.9418 |
| **Household income quintile Q3** | 0.1434 | 0.8386 | 0.1700 | 0.8642 | -1.5004 | 1.7872 |
| **Household income quintile Q4** | 1.8691 | 0.8997 | 2.0800 | 0.0378 | 0.1056 | 3.6325 |
| **Household income quintile Q5 (highest income)** | 1.7051 | 0.9738 | 1.7500 | 0.0800 | -0.2036 | 3.6138 |
| **Household income quintile Q1 (lowest income) (REF)** | 0.0000 | . | . | . | . | . |
| **NB Health Zone 2** | 4.6872 | 0.6389 | 7.3400 | <.0001 | 3.4350 | 5.9395 |
| **NB Health Zone 3** | 2.4386 | 0.6247 | 3.9000 | <.0001 | 1.2142 | 3.6630 |
| **NB Health Zone 4** | 3.0728 | 0.8265 | 3.7200 | 0.0002 | 1.4528 | 4.6929 |
| **NB Health Zone 5** | 6.8589 | 1.2058 | 5.6900 | <.0001 | 4.4956 | 9.2223 |
| **NB Health Zone 6** | 6.3706 | 0.7908 | 8.0600 | <.0001 | 4.8205 | 7.9207 |
| **NB Health Zone 7** | -1.1430 | 1.0064 | -1.1400 | 0.2561 | -3.1157 | 0.8297 |
| **NB Health Zone 1 (REF)** | 0.0000 | . | . | . | . | . |
| **Comorbid conditions - Mood & anxiety disorders (yes)** | -1.7315 | 1.2673 | -1.3700 | 0.1718 | -4.2155 | 0.7524 |
| **Comorbid conditions - Mood & anxiety disorders (no) (REF)** | 0.0000 | . | . | . | . | . |
| **Comorbid conditions – One or more of: asthma, diabetes, epilepsy, schizophrenia (yes)** | 0.9028 | 2.1923 | 0.4100 | 0.6805 | -3.3942 | 5.1998 |
| **Comorbid conditions – One or more of: asthma, diabetes, epilepsy, schizophrenia (no) (REF)** | 0.0000 | . | . | . | . | . |
| **Select medications (one or more)** | -1.7377 | 1.2868 | -1.3500 | 0.1769 | -4.2601 | 0.7846 |
| **Select medications (none) (REF)** | 0.0000 | . | . | . | . | . |
| **School District - Anglophone** | 29.7099 | 5.8298 | 5.1000 | <.0001 | 18.2830 | 41.1368 |
| **School District – Francophone (REF)** | 0.0000 | . | . | . | . | . |
| **CIMD - Residential Instability Q2** | 0.2599 | 0.5930 | 0.4400 | 0.6612 | -0.9024 | 1.4221 |
| **CIMD - Residential Instability Q3** | 0.5332 | 0.6321 | 0.8400 | 0.3990 | -0.7058 | 1.7721 |
| **CIMD - Residential Instability Q4** | 1.0463 | 0.7455 | 1.4000 | 0.1605 | -0.4149 | 2.5075 |
| **CIMD – Residential Instability Q5 (most deprived)** | 1.8583 | 1.0330 | 1.8000 | 0.0721 | -0.1666 | 3.8831 |
| **CIMD - Residential Instability Q1 (least deprived) (REF)** | 0.0000 | . | . | . | . | . |
| **CIMD - Economic Dependency Q2** | -1.8349 | 0.7682 | -2.3900 | 0.0169 | -3.3408 | -0.3291 |
| **CIMD - Economic Dependency Q3** | -0.5380 | 0.7777 | -0.6900 | 0.4891 | -2.0624 | 0.9864 |
| **CIMD - Economic Dependency Q4** | -1.3100 | 0.8043 | -1.6300 | 0.1034 | -2.8864 | 0.2664 |
| **CIMD - Economic Dependency Q5 (most deprived)** | -2.3840 | 0.8229 | -2.9000 | 0.0038 | -3.9969 | -0.7710 |
| **CIMD - Economic Dependency Q1 (least deprived) (REF)** | 0.0000 | . | . | . | . | . |
| **CIMD - Ethnocultural Composition Q2** | -0.7379 | 0.4621 | -1.6000 | 0.1103 | -1.6437 | 0.1678 |
| **CIMD - Ethnocultural Composition Q3** | 0.4166 | 0.6668 | 0.6200 | 0.5321 | -0.8903 | 1.7236 |
| **CIMD - Ethnocultural Composition Q4** | 1.9864 | 1.0031 | 1.9800 | 0.0477 | 0.0201 | 3.9526 |
| **CIMD - Ethnocultural Composition Q5 (most deprived)** | -4.6941 | 1.6225 | -2.8900 | 0.0038 | -7.8743 | -1.5138 |
| **CIMD - Ethnocultural Composition Q1 (least deprived) (REF)** | 0.0000 | . | . | . | . | . |
| **CIMD -Situational Vulnerability Q2** | -2.8105 | 0.7528 | -3.7300 | 0.0002 | -4.2861 | -1.3350 |
| **CIMD - Situational Vulnerability Q3** | -3.5817 | 0.8195 | -4.3700 | <.0001 | -5.1880 | -1.9754 |
| **CIMD -Situational Vulnerability Q4** | -4.2720 | 0.8063 | -5.3000 | <.0001 | -5.8525 | -2.6916 |
| **CIMD -Situational Vulnerability Q5 (most deprived)** | -6.6296 | 0.8762 | -7.5700 | <.0001 | -8.3470 | -4.9122 |
| **CIMD - Situational Vulnerability Q1 (least deprived) (REF)** | 0.0000 | . | . | . | . | . |
| **Social Assistance (any in past 5 years)** | -8.9715 | 0.6196 | -14.4800 | <.0001 | -10.1860 | -7.7569 |
| **Social Assistance (none in past 5 years) (REF)** | 0.0000 | . | . | . | . | . |
| **Program of Study - French Immersion/Other** | 5.3847 | 0.5754 | 9.3600 | <.0001 | 4.2568 | 6.5125 |
| **Program of Study - French** | 32.6677 | 5.8350 | 5.6000 | <.0001 | 21.2305 | 44.1049 |
| **Program of Study - English (REF)** | 0.0000 | . | . | . | . | . |
| **Household composition – Adults (age 22+) – No adults in household** | -7.3738 | 3.6557 | -2.0200 | 0.0437 | -14.5393 | -0.2083 |
| **Household composition – Adults (age 22+) – One adult in household** | -3.5839 | 0.4854 | -7.3800 | <.0001 | -4.5353 | -2.6325 |
| **Household composition – Adults (age 22+) – More than one adult in household (REF)** | 0.0000 | . | . | . | . | . |
| **Household composition – Children (age 21 or under) – Student is only child in household** | -1.6094 | 0.5308 | -3.0300 | 0.0024 | -2.6499 | -0.5689 |
| **Household composition – Children (age 21 or under) – Other children in household (REF)** | 0.0000 | . | . | . | . | . |
| **Recent immigrant** | -4.7095 | 1.3759 | -3.4200 | 0.0006 | -7.4064 | -2.0126 |
| **Not a recent immigrant (REF)** | 0.0000 | . | . | . | . | . |

**Supplementary Table S7b. GLM regression estimates - Provincial assessment exams percentile rank for STEM subjects for grades K-8 (AY 2017-2019)**

| **Parameter** | **Estimate** | **Standard**  **Error** | **t Value** | **Pr > \|t\|** | **95% Confidence Limits** | |
| --- | --- | --- | --- | --- | --- | --- |
| **Intercept** | 53.4102 | 11.3390 | 4.7100 | <.0001 | 31.1840 | 75.6365 |
| **Treated ADHD** | -8.6382 | 0.8997 | -9.6000 | <.0001 | -10.4018 | -6.8746 |
| **Untreated ADHD** | -9.4770 | 0.7076 | -13.3900 | <.0001 | -10.8640 | -8.0899 |
| **No ADHD (REF)** | 0.0000 | . | . | . | . | . |
| **Age** | -2.8592 | 0.4846 | -5.9000 | <.0001 | -3.8090 | -1.9093 |
| **Male** | 2.3306 | 0.5090 | 4.5800 | <.0001 | 1.3328 | 3.3284 |
| **Female (REF)** | 0.0000 | . | . | . | . | . |
| **Household income quintile Q2** | 2.2869 | 0.9497 | 2.4100 | 0.0161 | 0.4253 | 4.1485 |
| **Household income quintile Q3** | 0.6252 | 1.0248 | 0.6100 | 0.5418 | -1.3836 | 2.6340 |
| **Household income quintile Q4** | 4.8487 | 1.0940 | 4.4300 | <.0001 | 2.7043 | 6.9931 |
| **Household income quintile Q5 (highest income)** | 5.7809 | 1.1856 | 4.8800 | <.0001 | 3.4569 | 8.1050 |
| **Household income quintile Q1 (lowest income) (REF)** | 0.0000 | . | . | . | . | . |
| **NB Health Zone 2** | 3.1070 | 0.6938 | 4.4800 | <.0001 | 1.7471 | 4.4669 |
| **NB Health Zone 3** | 4.8773 | 0.7032 | 6.9400 | <.0001 | 3.4988 | 6.2557 |
| **NB Health Zone 4** | -1.6951 | 1.4586 | -1.1600 | 0.2452 | -4.5542 | 1.1640 |
| **NB Health Zone 5** | -2.7094 | 1.7469 | -1.5500 | 0.1209 | -6.1337 | 0.7148 |
| **NB Health Zone 6** | -0.1216 | 1.2564 | -0.1000 | 0.9229 | -2.5844 | 2.3411 |
| **NB Health Zone 7** | 5.4310 | 1.1763 | 4.6200 | <.0001 | 3.1253 | 7.7367 |
| **NB Health Zone 1 (REF)** | 0.0000 | . | . | . | . | . |
| **Comorbid conditions - Mood & anxiety disorders (yes)** | -3.2063 | 0.8724 | -3.6800 | 0.0002 | -4.9163 | -1.4963 |
| **Comorbid conditions - Mood & anxiety disorders (no) (REF)** | 0.0000 | . | . | . | . | . |
| **Comorbid conditions – One or more of: asthma, diabetes, epilepsy, schizophrenia (yes)** | -2.6404 | 2.9252 | -0.9000 | 0.3667 | -8.3743 | 3.0935 |
| **Comorbid conditions – One or more of: asthma, diabetes, epilepsy, schizophrenia (no) (REF)** | 0.0000 | . | . | . | . | . |
| **Select medications (one or more)** | -4.6324 | 1.3620 | -3.4000 | 0.0007 | -7.3021 | -1.9626 |
| **Select medications (none) (REF)** | 0.0000 | . | . | . | . | . |
| **School District - Anglophone** | 30.5551 | 8.6817 | 3.5200 | 0.0004 | 13.5376 | 47.5726 |
| **School District – Francophone (REF)** | 0.0000 | . | . | . | . | . |
| **CIMD - Residential Instability Q2** | -0.0739 | 0.7179 | -0.1000 | 0.9180 | -1.4812 | 1.3333 |
| **CIMD - Residential Instability Q3** | -0.4708 | 0.7859 | -0.6000 | 0.5492 | -2.0113 | 1.0698 |
| **CIMD - Residential Instability Q4** | 2.6571 | 0.9049 | 2.9400 | 0.0033 | 0.8833 | 4.4308 |
| **CIMD – Residential Instability Q5 (most deprived)** | 5.3475 | 1.2726 | 4.2000 | <.0001 | 2.8529 | 7.8420 |
| **CIMD - Residential Instability Q1 (least deprived) (REF)** | 0.0000 | . | . | . | . | . |
| **CIMD - Economic Dependency Q2** | 1.2277 | 0.9212 | 1.3300 | 0.1826 | -0.5780 | 3.0333 |
| **CIMD - Economic Dependency Q3** | 4.0441 | 0.9256 | 4.3700 | <.0001 | 2.2298 | 5.8585 |
| **CIMD - Economic Dependency Q4** | 2.3974 | 0.9608 | 2.5000 | 0.0126 | 0.5141 | 4.2807 |
| **CIMD - Economic Dependency Q5 (most deprived)** | 2.9098 | 0.9830 | 2.9600 | 0.0031 | 0.9831 | 4.8366 |
| **CIMD - Economic Dependency Q1 (least deprived) (REF)** | 0.0000 | . | . | . | . | . |
| **CIMD - Ethnocultural Composition Q2** | -0.4119 | 0.5683 | -0.7200 | 0.4686 | -1.5259 | 0.7021 |
| **CIMD - Ethnocultural Composition Q3** | 0.0618 | 0.8123 | 0.0800 | 0.9394 | -1.5304 | 1.6540 |
| **CIMD - Ethnocultural Composition Q4** | 4.6489 | 1.2297 | 3.7800 | 0.0002 | 2.2384 | 7.0593 |
| **CIMD - Ethnocultural Composition Q5 (most deprived)** | 4.5774 | 2.0267 | 2.2600 | 0.0239 | 0.6047 | 8.5501 |
| **CIMD - Ethnocultural Composition Q1 (least deprived) (REF)** | 0.0000 | . | . | . | . | . |
| **CIMD -Situational Vulnerability Q2** | -2.8804 | 0.8582 | -3.3600 | 0.0008 | -4.5627 | -1.1981 |
| **CIMD - Situational Vulnerability Q3** | -2.7216 | 0.9753 | -2.7900 | 0.0053 | -4.6333 | -0.8099 |
| **CIMD -Situational Vulnerability Q4** | -2.5093 | 0.9509 | -2.6400 | 0.0083 | -4.3732 | -0.6454 |
| **CIMD -Situational Vulnerability Q5 (most deprived)** | -4.4697 | 1.0402 | -4.3000 | <.0001 | -6.5086 | -2.4307 |
| **CIMD - Situational Vulnerability Q1 (least deprived) (REF)** | 0.0000 | . | . | . | . | . |
| **Social Assistance (any in past 5 years)** | -7.0336 | 0.8472 | -8.3000 | <.0001 | -8.6941 | -5.3730 |
| **Social Assistance (none in past 5 years) (REF)** | 0.0000 | . | . | . | . | . |
| **Program of Study - French Immersion/Other** | 13.0181 | 0.5826 | 22.3400 | <.0001 | 11.8760 | 14.1601 |
| **Program of Study - French** | 42.9876 | 8.7311 | 4.9200 | <.0001 | 25.8732 | 60.1019 |
| **Program of Study - English (REF)** | 0.0000 | . | . | . | . | . |
| **Household composition – Adults (age 22+) – No adults in household** | -0.3652 | 7.3722 | -0.0500 | 0.9605 | -14.8158 | 14.0854 |
| **Household composition – Adults (age 22+) – One adult in household** | -2.6638 | 0.6589 | -4.0400 | <.0001 | -3.9553 | -1.3723 |
| **Household composition – Adults (age 22+) – More than one adult in household (REF)** | 0.0000 | . | . | . | . | . |
| **Household composition – Children (age 21 or under) – Student is only child in household** | -3.1607 | 0.5954 | -5.3100 | <.0001 | -4.3278 | -1.9935 |
| **Household composition – Children (age 21 or under) – Other children in household (REF)** | 0.0000 | . | . | . | . | . |
| **Recent immigrant** | -1.9902 | 1.7348 | -1.1500 | 0.2513 | -5.3906 | 1.4102 |
| **Not a recent immigrant (REF)** | 0.0000 | **.** | **.** | **.** | **.** | **.** |

**Supplementary Table S7c. GLM regression estimates - Provincial assessment exams percentile rank for math for grades K-8 (AY 2017-2019)**

| **Parameter** | **Estimate** | **Standard**  **Error** | **t Value** | **Pr > \|t\|** | **95% Confidence Limits** | |
| --- | --- | --- | --- | --- | --- | --- |
| **Intercept** | 19.5569 | 6.6874 | 2.9200 | 0.0035 | 6.4489 | 32.6648 |
| **Treated ADHD** | -14.7890 | 0.7862 | -18.8100 | <.0001 | -16.3300 | -13.2479 |
| **Untreated ADHD** | -13.6511 | 0.7144 | -19.1100 | <.0001 | -15.0515 | -12.2507 |
| **No ADHD (REF)** | 0.0000 | . | . | . | . | . |
| **Age** | 0.1314 | 0.1069 | 1.2300 | 0.2192 | -0.0782 | 0.3409 |
| **Male** | 2.2328 | 0.4252 | 5.2500 | <.0001 | 1.3993 | 3.0663 |
| **Female (REF)** | 0.0000 | . | . | . | . | . |
| **Household income quintile Q2** | 0.6375 | 0.8225 | 0.7800 | 0.4383 | -0.9747 | 2.2497 |
| **Household income quintile Q3** | -0.0521 | 0.8791 | -0.0600 | 0.9527 | -1.7753 | 1.6710 |
| **Household income quintile Q4** | 1.8231 | 0.9433 | 1.9300 | 0.0533 | -0.0259 | 3.6722 |
| **Household income quintile Q5 (highest income)** | 1.9386 | 1.0195 | 1.9000 | 0.0573 | -0.0598 | 3.9370 |
| **Household income quintile Q1 (lowest income) (REF)** | 0.0000 | . | . | . | . | . |
| **NB Health Zone 2** | 7.6220 | 0.6720 | 11.3400 | <.0001 | 6.3047 | 8.9393 |
| **NB Health Zone 3** | 3.6078 | 0.6540 | 5.5200 | <.0001 | 2.3259 | 4.8896 |
| **NB Health Zone 4** | 4.8097 | 0.8602 | 5.5900 | <.0001 | 3.1236 | 6.4958 |
| **NB Health Zone 5** | 7.8320 | 1.2561 | 6.2400 | <.0001 | 5.3698 | 10.2941 |
| **NB Health Zone 6** | 8.3098 | 0.8283 | 10.0300 | <.0001 | 6.6862 | 9.9333 |
| **NB Health Zone 7** | 1.9284 | 1.0505 | 1.8400 | 0.0664 | -0.1307 | 3.9874 |
| **NB Health Zone 1 (REF)** | 0.0000 | . | . | . | . | . |
| **Comorbid conditions - Mood & anxiety disorders (yes)** | -3.0294 | 1.3727 | -2.2100 | 0.0273 | -5.7201 | -0.3387 |
| **Comorbid conditions - Mood & anxiety disorders (no) (REF)** | 0.0000 | . | . | . | . | . |
| **Comorbid conditions – One or more of: asthma, diabetes, epilepsy, schizophrenia (yes)** | 0.7869 | 2.3647 | 0.3300 | 0.7393 | -3.8482 | 5.4219 |
| **Comorbid conditions – One or more of: asthma, diabetes, epilepsy, schizophrenia (no) (REF)** | 0.0000 | . | . | . | . | . |
| **Select medications (one or more)** | -2.0759 | 1.4894 | -1.3900 | 0.1634 | -4.9953 | 0.8435 |
| **Select medications (none) (REF)** | 0.0000 | . | . | . | . | . |
| **School District - Anglophone** | 29.5440 | 6.3768 | 4.6300 | <.0001 | 17.0449 | 42.0431 |
| **School District – Francophone (REF)** | 0.0000 | . | . | . | . | . |
| **CIMD - Residential Instability Q2** | -0.3655 | 0.6173 | -0.5900 | 0.5538 | -1.5755 | 0.8445 |
| **CIMD - Residential Instability Q3** | -0.4133 | 0.6593 | -0.6300 | 0.5307 | -1.7056 | 0.8790 |
| **CIMD - Residential Instability Q4** | 0.4912 | 0.7789 | 0.6300 | 0.5283 | -1.0356 | 2.0179 |
| **CIMD – Residential Instability Q5 (most deprived)** | 0.8222 | 1.0892 | 0.7500 | 0.4504 | -1.3128 | 2.9571 |
| **CIMD - Residential Instability Q1 (least deprived) (REF)** | 0.0000 | . | . | . | . | . |
| **CIMD - Economic Dependency Q2** | -1.4830 | 0.8013 | -1.8500 | 0.0642 | -3.0537 | 0.0877 |
| **CIMD - Economic Dependency Q3** | -0.5266 | 0.8094 | -0.6500 | 0.5153 | -2.1131 | 1.0600 |
| **CIMD - Economic Dependency Q4** | -1.3453 | 0.8392 | -1.6000 | 0.1089 | -2.9903 | 0.2997 |
| **CIMD - Economic Dependency Q5 (most deprived)** | -2.7096 | 0.8573 | -3.1600 | 0.0016 | -4.3901 | -1.0292 |
| **CIMD - Economic Dependency Q1 (least deprived) (REF)** | 0.0000 | . | . | . | . | . |
| **CIMD - Ethnocultural Composition Q2** | -0.4364 | 0.4835 | -0.9000 | 0.3668 | -1.3841 | 0.5114 |
| **CIMD - Ethnocultural Composition Q3** | 1.0513 | 0.6961 | 1.5100 | 0.1310 | -0.3131 | 2.4157 |
| **CIMD - Ethnocultural Composition Q4** | 1.0495 | 1.0537 | 1.0000 | 0.3193 | -1.0158 | 3.1148 |
| **CIMD - Ethnocultural Composition Q5 (most deprived)** | -3.7638 | 1.7343 | -2.1700 | 0.0300 | -7.1631 | -0.3644 |
| **CIMD - Ethnocultural Composition Q1 (least deprived) (REF)** | 0.0000 | . | . | . | . | . |
| **CIMD -Situational Vulnerability Q2** | -3.1067 | 0.7831 | -3.9700 | <.0001 | -4.6416 | -1.5717 |
| **CIMD - Situational Vulnerability Q3** | -3.0855 | 0.8536 | -3.6100 | 0.0003 | -4.7585 | -1.4124 |
| **CIMD -Situational Vulnerability Q4** | -3.7855 | 0.8401 | -4.5100 | <.0001 | -5.4321 | -2.1389 |
| **CIMD -Situational Vulnerability Q5 (most deprived)** | -5.8784 | 0.9141 | -6.4300 | <.0001 | -7.6702 | -4.0866 |
| **CIMD - Situational Vulnerability Q1 (least deprived) (REF)** | 0.0000 | . | . | . | . | . |
| **Social Assistance (any in past 5 years)** | -9.0888 | 0.6632 | -13.7000 | <.0001 | -10.3887 | -7.7889 |
| **Social Assistance (none in past 5 years) (REF)** | 0.0000 | . | . | . | . | . |
| **Program of Study - French Immersion/Other** | 4.7267 | 0.5984 | 7.9000 | <.0001 | 3.5537 | 5.8996 |
| **Program of Study - French** | 33.2544 | 6.3841 | 5.2100 | <.0001 | 20.7410 | 45.7679 |
| **Program of Study - English (REF)** | 0.0000 | . | . | . | . | . |
| **Household composition – Adults (age 22+) – No adults in household** | -6.9107 | 4.0532 | -1.7100 | 0.0882 | -14.8554 | 1.0339 |
| **Household composition – Adults (age 22+) – One adult in household** | -3.3241 | 0.5102 | -6.5200 | <.0001 | -4.3241 | -2.3241 |
| **Household composition – Adults (age 22+) – More than one adult in household (REF)** | 0.0000 | . | . | . | . | . |
| **Household composition – Children (age 21 or under) – Student is only child in household** | -2.4280 | 0.5584 | -4.3500 | <.0001 | -3.5225 | -1.3336 |
| **Household composition – Children (age 21 or under) – Other children in household (REF)** | 0.0000 | . | . | . | . | . |
| **Recent immigrant** | -0.7087 | 1.4171 | -0.5000 | 0.6170 | -3.4863 | 2.0690 |
| **Not a recent immigrant (REF)** | 0.0000 | . | . | . | . | . |

**Supplementary Table S7d. GLM regression estimates - Provincial assessment exams percentile rank for language for grades K-8 (AY 2017-2019)**

| **Parameter** | **Estimate** | **Standard**  **Error** | **t Value** | **Pr > \|t\|** | **95% Confidence Limits** | |
| --- | --- | --- | --- | --- | --- | --- |
| **Intercept** | 79.0590 | 28.1310 | 2.8100 | 0.0050 | 23.9186 | 134.1993 |
| **Treated ADHD** | -8.8087 | 0.9739 | -9.0400 | <.0001 | -10.7177 | -6.8997 |
| **Untreated ADHD** | -8.3170 | 0.7914 | -10.5100 | <.0001 | -9.8682 | -6.7657 |
| **No ADHD (REF)** | 0.0000 | . | . | . | . | . |
| **Age** | 0.4294 | 0.1891 | 2.2700 | 0.0232 | 0.0588 | 0.8000 |
| **Male** | -5.0950 | 0.4759 | -10.7100 | <.0001 | -6.0279 | -4.1621 |
| **Female (REF)** | 0.0000 | . | . | . | . | . |
| **Household income quintile Q2** | -0.0525 | 0.9091 | -0.0600 | 0.9540 | -1.8344 | 1.7294 |
| **Household income quintile Q3** | 0.7959 | 1.0105 | 0.7900 | 0.4309 | -1.1848 | 2.7766 |
| **Household income quintile Q4** | 1.8973 | 1.0843 | 1.7500 | 0.0802 | -0.2281 | 4.0227 |
| **Household income quintile Q5 (highest income)** | 1.1607 | 1.1756 | 0.9900 | 0.3235 | -1.1437 | 3.4650 |
| **Household income quintile Q1 (lowest income) (REF)** | 0.0000 | . | . | . | . | . |
| **NB Health Zone 2** | 4.3266 | 0.6409 | 6.7500 | <.0001 | 3.0703 | 5.5829 |
| **NB Health Zone 3** | 3.3637 | 0.6474 | 5.2000 | <.0001 | 2.0946 | 4.6328 |
| **NB Health Zone 4** | 2.4990 | 2.2368 | 1.1200 | 0.2639 | -1.8855 | 6.8835 |
| **NB Health Zone 5** | 6.5366 | 1.9830 | 3.3000 | 0.0010 | 2.6498 | 10.4235 |
| **NB Health Zone 6** | 4.5069 | 1.6329 | 2.7600 | 0.0058 | 1.3061 | 7.7076 |
| **NB Health Zone 7** | -0.1554 | 1.1519 | -0.1300 | 0.8927 | -2.4133 | 2.1025 |
| **NB Health Zone 1 (REF)** | 0.0000 | . | . | . | . | . |
| **Comorbid conditions - Mood & anxiety disorders (yes)** | 0.6655 | 1.7012 | 0.3900 | 0.6957 | -2.6691 | 4.0001 |
| **Comorbid conditions - Mood & anxiety disorders (no) (REF)** | 0.0000 | . | . | . | . | . |
| **Comorbid conditions – One or more of: asthma, diabetes, epilepsy, schizophrenia (yes)** | 4.9722 | 2.4855 | 2.0000 | 0.0455 | 0.1003 | 9.8440 |
| **Comorbid conditions – One or more of: asthma, diabetes, epilepsy, schizophrenia (no) (REF)** | 0.0000 | . | . | . | . | . |
| **Select medications (one or more)** | -2.8109 | 1.6340 | -1.7200 | 0.0854 | -6.0138 | 0.3921 |
| **Select medications (none) (REF)** | 0.0000 | . | . | . | . | . |
| **School District - Anglophone** | -32.3651 | 27.9515 | -1.1600 | 0.2469 | -87.1537 | 22.4236 |
| **School District – Francophone (REF)** | 0.0000 | . | . | . | . | . |
| **CIMD - Residential Instability Q2** | 1.0131 | 0.7001 | 1.4500 | 0.1479 | -0.3593 | 2.3854 |
| **CIMD - Residential Instability Q3** | 1.0248 | 0.7698 | 1.3300 | 0.1831 | -0.4842 | 2.5338 |
| **CIMD - Residential Instability Q4** | -0.4079 | 0.8762 | -0.4700 | 0.6415 | -2.1254 | 1.3095 |
| **CIMD – Residential Instability Q5 (most deprived)** | 1.3127 | 1.1533 | 1.1400 | 0.2551 | -0.9480 | 3.5734 |
| **CIMD - Residential Instability Q1 (least deprived) (REF)** | 0.0000 | . | . | . | . | . |
| **CIMD - Economic Dependency Q2** | -1.3209 | 0.8514 | -1.5500 | 0.1208 | -2.9898 | 0.3480 |
| **CIMD - Economic Dependency Q3** | -1.3543 | 0.8565 | -1.5800 | 0.1138 | -3.0331 | 0.3245 |
| **CIMD - Economic Dependency Q4** | -1.4486 | 0.8879 | -1.6300 | 0.1028 | -3.1890 | 0.2918 |
| **CIMD - Economic Dependency Q5 (most deprived)** | -0.9160 | 0.9218 | -0.9900 | 0.3204 | -2.7228 | 0.8908 |
| **CIMD - Economic Dependency Q1 (least deprived) (REF)** | 0.0000 | . | . | . | . | . |
| **CIMD - Ethnocultural Composition Q2** | -0.3393 | 0.5521 | -0.6100 | 0.5388 | -1.4215 | 0.7428 |
| **CIMD - Ethnocultural Composition Q3** | 0.4467 | 0.7783 | 0.5700 | 0.5661 | -1.0790 | 1.9723 |
| **CIMD - Ethnocultural Composition Q4** | 3.6785 | 1.1089 | 3.3200 | 0.0009 | 1.5049 | 5.8522 |
| **CIMD - Ethnocultural Composition Q5 (most deprived)** | -5.2157 | 1.6254 | -3.2100 | 0.0013 | -8.4017 | -2.0298 |
| **CIMD - Ethnocultural Composition Q1 (least deprived) (REF)** | 0.0000 | . | . | . | . | . |
| **CIMD -Situational Vulnerability Q2** | -0.7850 | 0.8021 | -0.9800 | 0.3277 | -2.3572 | 0.7872 |
| **CIMD - Situational Vulnerability Q3** | -1.0058 | 0.9223 | -1.0900 | 0.2755 | -2.8137 | 0.8021 |
| **CIMD -Situational Vulnerability Q4** | -2.1799 | 0.8997 | -2.4200 | 0.0154 | -3.9435 | -0.4164 |
| **CIMD -Situational Vulnerability Q5 (most deprived)** | -4.0162 | 1.0021 | -4.0100 | <.0001 | -5.9805 | -2.0519 |
| **CIMD - Situational Vulnerability Q1 (least deprived) (REF)** | 0.0000 | . | . | . | . | . |
| **Social Assistance (any in past 5 years)** | -5.8331 | 0.6740 | -8.6500 | <.0001 | -7.1542 | -4.5119 |
| **Social Assistance (none in past 5 years) (REF)** | 0.0000 | . | . | . | . | . |
| **Program of Study - French Immersion/Other** | 5.3602 | 0.5228 | 10.2500 | <.0001 | 4.3354 | 6.3850 |
| **Program of Study - French** | -32.5747 | 28.0008 | -1.1600 | 0.2447 | -87.4599 | 22.3106 |
| **Program of Study - English (REF)** | 0.0000 | . | . | . | . | . |
| **Household composition – Adults (age 22+) – No adults in household** | -6.6048 | 4.1988 | -1.5700 | 0.1157 | -14.8350 | 1.6254 |
| **Household composition – Adults (age 22+) – One adult in household** | -2.5857 | 0.5939 | -4.3500 | <.0001 | -3.7498 | -1.4216 |
| **Household composition – Adults (age 22+) – More than one adult in household (REF)** | 0.0000 | . | . | . | . | . |
| **Household composition – Children (age 21 or under) – Student is only child in household** | 0.7635 | 0.6512 | 1.1700 | 0.2410 | -0.5129 | 2.0399 |
| **Household composition – Children (age 21 or under) – Other children in household (REF)** | 0.0000 | . | . | . | . | . |
| **Recent immigrant** | -2.7449 | 1.3957 | -1.9700 | 0.0492 | -5.4807 | -0.0091 |
| **Not a recent immigrant (REF)** | 0.0000 | . | . | . | . | . |

**Supplementary Table S7e. GLM regression estimates - Provincial assessment exams overall percentile rank for grades 9-11 (AY 2017-2019)**

| **Parameter** | **Estimate** | **Standard**  **Error** | **t Value** | **Pr > \|t\|** | **95% Confidence Limits** | |
| --- | --- | --- | --- | --- | --- | --- |
| **Intercept** | 42.1191 | 10.7724 | 3.9100 | <.0001 | 21.0041 | 63.2341 |
| **Treated ADHD** | -8.7767 | 0.8589 | -10.2200 | <.0001 | -10.4602 | -7.0932 |
| **Untreated ADHD** | -9.5647 | 0.6508 | -14.7000 | <.0001 | -10.8403 | -8.2892 |
| **No ADHD (REF)** | 0.0000 | . | . | . | . | . |
| **Age** | -1.5841 | 0.3484 | -4.5500 | <.0001 | -2.2670 | -0.9013 |
| **Male** | -1.9053 | 0.4354 | -4.3800 | <.0001 | -2.7588 | -1.0518 |
| **Female (REF)** | 0.0000 | . | . | . | . | . |
| **Household income quintile Q2** | 1.9179 | 0.8238 | 2.3300 | 0.0199 | 0.3032 | 3.5327 |
| **Household income quintile Q3** | 1.6385 | 0.8949 | 1.8300 | 0.0671 | -0.1156 | 3.3925 |
| **Household income quintile Q4** | 3.2380 | 0.9596 | 3.3700 | 0.0007 | 1.3571 | 5.1188 |
| **Household income quintile Q5 (highest income)** | 4.2746 | 1.0455 | 4.0900 | <.0001 | 2.2253 | 6.3239 |
| **Household income quintile Q1 (lowest income) (REF)** | 0.0000 | . | . | . | . | . |
| **NB Health Zone 2** | 3.9156 | 0.5998 | 6.5300 | <.0001 | 2.7399 | 5.0913 |
| **NB Health Zone 3** | 5.0494 | 0.5978 | 8.4500 | <.0001 | 3.8777 | 6.2210 |
| **NB Health Zone 4** | 1.5682 | 1.3707 | 1.1400 | 0.2526 | -1.1184 | 4.2549 |
| **NB Health Zone 5** | 1.7799 | 1.5430 | 1.1500 | 0.2487 | -1.2446 | 4.8044 |
| **NB Health Zone 6** | 6.8997 | 1.1538 | 5.9800 | <.0001 | 4.6382 | 9.1612 |
| **NB Health Zone 7** | 4.0032 | 1.0240 | 3.9100 | <.0001 | 1.9961 | 6.0103 |
| **NB Health Zone 1 (REF)** | 0.0000 | . | . | . | . | . |
| **Comorbid conditions - Mood & anxiety disorders (yes)** | -0.6766 | 0.8653 | -0.7800 | 0.4342 | -2.3727 | 1.0194 |
| **Comorbid conditions - Mood & anxiety disorders (no) (REF)** | 0.0000 | . | . | . | . | . |
| **Comorbid conditions – One or more of: asthma, diabetes, epilepsy, schizophrenia (yes)** | 3.9702 | 2.6117 | 1.5200 | 0.1285 | -1.1490 | 9.0895 |
| **Comorbid conditions – One or more of: asthma, diabetes, epilepsy, schizophrenia (no) (REF)** | 0.0000 | . | . | . | . | . |
| **Select medications (one or more)** | -3.5133 | 1.2504 | -2.8100 | 0.0050 | -5.9641 | -1.0624 |
| **Select medications (none) (REF)** | 0.0000 | . | . | . | . | . |
| **School District - Anglophone** | 26.2666 | 9.1708 | 2.8600 | 0.0042 | 8.2909 | 44.2423 |
| **School District – Francophone (REF)** | 0.0000 | . | . | . | . | . |
| **CIMD - Residential Instability Q2** | -0.7173 | 0.6291 | -1.1400 | 0.2542 | -1.9503 | 0.5157 |
| **CIMD - Residential Instability Q3** | -0.4501 | 0.6882 | -0.6500 | 0.5131 | -1.7991 | 0.8989 |
| **CIMD - Residential Instability Q4** | 1.7371 | 0.7911 | 2.2000 | 0.0281 | 0.1865 | 3.2877 |
| **CIMD – Residential Instability Q5 (most deprived)** | 3.9928 | 1.0813 | 3.6900 | 0.0002 | 1.8733 | 6.1124 |
| **CIMD - Residential Instability Q1 (least deprived) (REF)** | 0.0000 | . | . | . | . | . |
| **CIMD - Economic Dependency Q2** | 0.0086 | 0.7831 | 0.0100 | 0.9912 | -1.5263 | 1.5436 |
| **CIMD - Economic Dependency Q3** | 0.9755 | 0.7810 | 1.2500 | 0.2117 | -0.5554 | 2.5064 |
| **CIMD - Economic Dependency Q4** | -0.1661 | 0.8158 | -0.2000 | 0.8387 | -1.7651 | 1.4329 |
| **CIMD - Economic Dependency Q5 (most deprived)** | 1.3246 | 0.8389 | 1.5800 | 0.1144 | -0.3197 | 2.9689 |
| **CIMD - Economic Dependency Q1 (least deprived) (REF)** | 0.0000 | . | . | . | . | . |
| **CIMD - Ethnocultural Composition Q2** | -0.6873 | 0.4931 | -1.3900 | 0.1634 | -1.6539 | 0.2794 |
| **CIMD - Ethnocultural Composition Q3** | 0.8188 | 0.7043 | 1.1600 | 0.2450 | -0.5617 | 2.1992 |
| **CIMD - Ethnocultural Composition Q4** | 3.6732 | 1.0526 | 3.4900 | 0.0005 | 1.6100 | 5.7364 |
| **CIMD - Ethnocultural Composition Q5 (most deprived)** | 1.5930 | 1.6331 | 0.9800 | 0.3294 | -1.6081 | 4.7941 |
| **CIMD - Ethnocultural Composition Q1 (least deprived) (REF)** | 0.0000 | . | . | . | . | . |
| **CIMD -Situational Vulnerability Q2** | -3.4745 | 0.7324 | -4.7400 | <.0001 | -4.9102 | -2.0389 |
| **CIMD - Situational Vulnerability Q3** | -3.9284 | 0.8478 | -4.6300 | <.0001 | -5.5902 | -2.2666 |
| **CIMD -Situational Vulnerability Q4** | -3.9565 | 0.8218 | -4.8100 | <.0001 | -5.5674 | -2.3456 |
| **CIMD -Situational Vulnerability Q5 (most deprived)** | -5.6419 | 0.9063 | -6.2200 | <.0001 | -7.4184 | -3.8654 |
| **CIMD - Situational Vulnerability Q1 (least deprived) (REF)** | 0.0000 | . | . | . | . | . |
| **Social Assistance (any in past 5 years)** | -9.7376 | 0.7053 | -13.8100 | <.0001 | -11.1201 | -8.3550 |
| **Social Assistance (none in past 5 years) (REF)** | 0.0000 | . | . | . | . | . |
| **Program of Study - French Immersion/Other** | 12.8649 | 0.4925 | 26.1200 | <.0001 | 11.8996 | 13.8302 |
| **Program of Study - French** | 33.6394 | 9.1836 | 3.6600 | 0.0003 | 15.6386 | 51.6402 |
| **Program of Study - English (REF)** | 0.0000 | . | . | . | . | . |
| **Household composition – Adults (age 22+) – No adults in household** | -13.5725 | 5.4051 | -2.5100 | 0.0120 | -24.1671 | -2.9779 |
| **Household composition – Adults (age 22+) – One adult in household** | -2.9412 | 0.5601 | -5.2500 | <.0001 | -4.0390 | -1.8434 |
| **Household composition – Adults (age 22+) – More than one adult in household (REF)** | 0.0000 | . | . | . | . | . |
| **Household composition – Children (age 21 or under) – Student is only child in household** | 0.1066 | 0.5336 | 0.2000 | 0.8416 | -0.9393 | 1.1526 |
| **Household composition – Children (age 21 or under) – Other children in household (REF)** | 0.0000 | . | . | . | . | . |
| **Recent immigrant** | -7.6099 | 1.3376 | -5.6900 | <.0001 | -10.2318 | -4.9881 |
| **Not a recent immigrant (REF)** | 0.0000 | . | . | . | . | . |

**Supplementary Table S7f. GLM regression estimates - Provincial assessment exams percentile rank for STEM subjects for grades 9-11 (AY 2017-2019)**

| **Parameter** | **Estimate** | **Standard**  **Error** | **t Value** | **Pr > \|t\|** | **95% Confidence Limits** | |
| --- | --- | --- | --- | --- | --- | --- |
| **Intercept** | 53.4102 | 11.3390 | 4.7100 | <.0001 | 31.1840 | 75.6365 |
| **Treated ADHD** | -8.6382 | 0.8997 | -9.6000 | <.0001 | -10.4018 | -6.8746 |
| **Untreated ADHD** | -9.4770 | 0.7076 | -13.3900 | <.0001 | -10.8640 | -8.0899 |
| **No ADHD (REF)** | 0.0000 | . | . | . | . | . |
| **Age** | -2.8592 | 0.4846 | -5.9000 | <.0001 | -3.8090 | -1.9093 |
| **Male** | 2.3306 | 0.5090 | 4.5800 | <.0001 | 1.3328 | 3.3284 |
| **Female (REF)** | 0.0000 | . | . | . | . | . |
| **Household income quintile Q2** | 2.2869 | 0.9497 | 2.4100 | 0.0161 | 0.4253 | 4.1485 |
| **Household income quintile Q3** | 0.6252 | 1.0248 | 0.6100 | 0.5418 | -1.3836 | 2.6340 |
| **Household income quintile Q4** | 4.8487 | 1.0940 | 4.4300 | <.0001 | 2.7043 | 6.9931 |
| **Household income quintile Q5 (highest income)** | 5.7809 | 1.1856 | 4.8800 | <.0001 | 3.4569 | 8.1050 |
| **Household income quintile Q1 (lowest income) (REF)** | 0.0000 | . | . | . | . | . |
| **NB Health Zone 2** | 3.1070 | 0.6938 | 4.4800 | <.0001 | 1.7471 | 4.4669 |
| **NB Health Zone 3** | 4.8773 | 0.7032 | 6.9400 | <.0001 | 3.4988 | 6.2557 |
| **NB Health Zone 4** | -1.6951 | 1.4586 | -1.1600 | 0.2452 | -4.5542 | 1.1640 |
| **NB Health Zone 5** | -2.7094 | 1.7469 | -1.5500 | 0.1209 | -6.1337 | 0.7148 |
| **NB Health Zone 6** | -0.1216 | 1.2564 | -0.1000 | 0.9229 | -2.5844 | 2.3411 |
| **NB Health Zone 7** | 5.4310 | 1.1763 | 4.6200 | <.0001 | 3.1253 | 7.7367 |
| **NB Health Zone 1 (REF)** | 0.0000 | . | . | . | . | . |
| **Comorbid conditions - Mood & anxiety disorders (yes)** | -3.2063 | 0.8724 | -3.6800 | 0.0002 | -4.9163 | -1.4963 |
| **Comorbid conditions - Mood & anxiety disorders (no) (REF)** | 0.0000 | . | . | . | . | . |
| **Comorbid conditions – One or more of: asthma, diabetes, epilepsy, schizophrenia (yes)** | -2.6404 | 2.9252 | -0.9000 | 0.3667 | -8.3743 | 3.0935 |
| **Comorbid conditions – One or more of: asthma, diabetes, epilepsy, schizophrenia (no) (REF)** | 0.0000 | . | . | . | . | . |
| **Select medications (one or more)** | -4.6324 | 1.3620 | -3.4000 | 0.0007 | -7.3021 | -1.9626 |
| **Select medications (none) (REF)** | 0.0000 | . | . | . | . | . |
| **School District - Anglophone** | 30.5551 | 8.6817 | 3.5200 | 0.0004 | 13.5376 | 47.5726 |
| **School District – Francophone (REF)** | 0.0000 | . | . | . | . | . |
| **CIMD - Residential Instability Q2** | -0.0739 | 0.7179 | -0.1000 | 0.9180 | -1.4812 | 1.3333 |
| **CIMD - Residential Instability Q3** | -0.4708 | 0.7859 | -0.6000 | 0.5492 | -2.0113 | 1.0698 |
| **CIMD - Residential Instability Q4** | 2.6571 | 0.9049 | 2.9400 | 0.0033 | 0.8833 | 4.4308 |
| **CIMD – Residential Instability Q5 (most deprived)** | 5.3475 | 1.2726 | 4.2000 | <.0001 | 2.8529 | 7.8420 |
| **CIMD - Residential Instability Q1 (least deprived) (REF)** | 0.0000 | . | . | . | . | . |
| **CIMD - Economic Dependency Q2** | 1.2277 | 0.9212 | 1.3300 | 0.1826 | -0.5780 | 3.0333 |
| **CIMD - Economic Dependency Q3** | 4.0441 | 0.9256 | 4.3700 | <.0001 | 2.2298 | 5.8585 |
| **CIMD - Economic Dependency Q4** | 2.3974 | 0.9608 | 2.5000 | 0.0126 | 0.5141 | 4.2807 |
| **CIMD - Economic Dependency Q5 (most deprived)** | 2.9098 | 0.9830 | 2.9600 | 0.0031 | 0.9831 | 4.8366 |
| **CIMD - Economic Dependency Q1 (least deprived) (REF)** | 0.0000 | . | . | . | . | . |
| **CIMD - Ethnocultural Composition Q2** | -0.4119 | 0.5683 | -0.7200 | 0.4686 | -1.5259 | 0.7021 |
| **CIMD - Ethnocultural Composition Q3** | 0.0618 | 0.8123 | 0.0800 | 0.9394 | -1.5304 | 1.6540 |
| **CIMD - Ethnocultural Composition Q4** | 4.6489 | 1.2297 | 3.7800 | 0.0002 | 2.2384 | 7.0593 |
| **CIMD - Ethnocultural Composition Q5 (most deprived)** | 4.5774 | 2.0267 | 2.2600 | 0.0239 | 0.6047 | 8.5501 |
| **CIMD - Ethnocultural Composition Q1 (least deprived) (REF)** | 0.0000 | . | . | . | . | . |
| **CIMD -Situational Vulnerability Q2** | -2.8804 | 0.8582 | -3.3600 | 0.0008 | -4.5627 | -1.1981 |
| **CIMD - Situational Vulnerability Q3** | -2.7216 | 0.9753 | -2.7900 | 0.0053 | -4.6333 | -0.8099 |
| **CIMD -Situational Vulnerability Q4** | -2.5093 | 0.9509 | -2.6400 | 0.0083 | -4.3732 | -0.6454 |
| **CIMD -Situational Vulnerability Q5 (most deprived)** | -4.4697 | 1.0402 | -4.3000 | <.0001 | -6.5086 | -2.4307 |
| **CIMD - Situational Vulnerability Q1 (least deprived) (REF)** | 0.0000 | . | . | . | . | . |
| **Social Assistance (any in past 5 years)** | -7.0336 | 0.8472 | -8.3000 | <.0001 | -8.6941 | -5.3730 |
| **Social Assistance (none in past 5 years) (REF)** | 0.0000 | . | . | . | . | . |
| **Program of Study - French Immersion/Other** | 13.0181 | 0.5826 | 22.3400 | <.0001 | 11.8760 | 14.1601 |
| **Program of Study - French** | 42.9876 | 8.7311 | 4.9200 | <.0001 | 25.8732 | 60.1019 |
| **Program of Study - English (REF)** | 0.0000 | . | . | . | . | . |
| **Household composition – Adults (age 22+) – No adults in household** | -0.3652 | 7.3722 | -0.0500 | 0.9605 | -14.8158 | 14.0854 |
| **Household composition – Adults (age 22+) – One adult in household** | -2.6638 | 0.6589 | -4.0400 | <.0001 | -3.9553 | -1.3723 |
| **Household composition – Adults (age 22+) – More than one adult in household (REF)** | 0.0000 | . | . | . | . | . |
| **Household composition – Children (age 21 or under) – Student is only child in household** | -3.1607 | 0.5954 | -5.3100 | <.0001 | -4.3278 | -1.9935 |
| **Household composition – Children (age 21 or under) – Other children in household (REF)** | 0.0000 | . | . | . | . | . |
| **Recent immigrant** | -1.9902 | 1.7348 | -1.1500 | 0.2513 | -5.3906 | 1.4102 |
| **Not a recent immigrant (REF)** | 0.0000 | . | . | . | . | . |

**Supplementary Table S7g. GLM regression estimates - Provincial assessment exams percentile rank for math for grades 9-11 (AY 2017-2019)**

| **Parameter** | **Estimate** | **Standard**  **Error** | **t Value** | **Pr > \|t\|** | **95% Confidence Limits** | |
| --- | --- | --- | --- | --- | --- | --- |
| **Intercept** | 55.7058 | 12.7564 | 4.3700 | <.0001 | 30.6996 | 80.7121 |
| **Treated ADHD** | -9.2039 | 1.1585 | -7.9400 | <.0001 | -11.4748 | -6.9330 |
| **Untreated ADHD** | -11.5046 | 0.9325 | -12.3400 | <.0001 | -13.3326 | -9.6766 |
| **No ADHD (REF)** | 0.0000 | . | . | . | . | . |
| **Age** | -2.7978 | 0.6221 | -4.5000 | <.0001 | -4.0173 | -1.5784 |
| **Male** | 2.5034 | 0.6638 | 3.7700 | 0.0002 | 1.2022 | 3.8046 |
| **Female (REF)** | 0.0000 | . | . | . | . | . |
| **Household income quintile Q2** | 1.9418 | 1.2409 | 1.5600 | 0.1177 | -0.4907 | 4.3742 |
| **Household income quintile Q3** | 0.5578 | 1.3275 | 0.4200 | 0.6744 | -2.0446 | 3.1601 |
| **Household income quintile Q4** | 4.3625 | 1.4156 | 3.0800 | 0.0021 | 1.5875 | 7.1375 |
| **Household income quintile Q5 (highest income)** | 5.9161 | 1.5315 | 3.8600 | 0.0001 | 2.9140 | 8.9182 |
| **Household income quintile Q1 (lowest income) (REF)** | 0.0000 | . | . | . | . | . |
| **NB Health Zone 2** | 4.1068 | 0.9432 | 4.3500 | <.0001 | 2.2578 | 5.9558 |
| **NB Health Zone 3** | 7.4105 | 0.9449 | 7.8400 | <.0001 | 5.5581 | 9.2628 |
| **NB Health Zone 4** | -0.6331 | 1.5745 | -0.4000 | 0.6876 | -3.7196 | 2.4533 |
| **NB Health Zone 5** | -0.7362 | 2.1141 | -0.3500 | 0.7277 | -4.8805 | 3.4080 |
| **NB Health Zone 6** | -0.1642 | 1.4284 | -0.1100 | 0.9085 | -2.9643 | 2.6359 |
| **NB Health Zone 7** | 5.4742 | 1.5536 | 3.5200 | 0.0004 | 2.4287 | 8.5198 |
| **NB Health Zone 1 (REF)** | 0.0000 | . | . | . | . | . |
| **Comorbid conditions - Mood & anxiety disorders (yes)** | -3.4187 | 1.1491 | -2.9800 | 0.0029 | -5.6713 | -1.1662 |
| **Comorbid conditions - Mood & anxiety disorders (no) (REF)** | 0.0000 | . | . | . | . | . |
| **Comorbid conditions – One or more of: asthma, diabetes, epilepsy, schizophrenia (yes)** | 0.0826 | 3.7426 | 0.0200 | 0.9824 | -7.2540 | 7.4193 |
| **Comorbid conditions – One or more of: asthma, diabetes, epilepsy, schizophrenia (no) (REF)** | 0.0000 | . | . | . | . | . |
| **Select medications (one or more)** | -3.9032 | 1.7951 | -2.1700 | 0.0297 | -7.4222 | -0.3842 |
| **Select medications (none) (REF)** | 0.0000 | . | . | . | . | . |
| **School District - Anglophone** | 27.6690 | 8.6589 | 3.2000 | 0.0014 | 10.6951 | 44.6429 |
| **School District – Francophone (REF)** | 0.0000 | . | . | . | . | . |
| **CIMD - Residential Instability Q2** | -1.0152 | 0.9385 | -1.0800 | 0.2794 | -2.8549 | 0.8245 |
| **CIMD - Residential Instability Q3** | -1.4506 | 1.0198 | -1.4200 | 0.1549 | -3.4496 | 0.5484 |
| **CIMD - Residential Instability Q4** | 2.4526 | 1.1835 | 2.0700 | 0.0383 | 0.1325 | 4.7727 |
| **CIMD – Residential Instability Q5 (most deprived)** | 5.9654 | 1.6774 | 3.5600 | 0.0004 | 2.6773 | 9.2536 |
| **CIMD - Residential Instability Q1 (least deprived) (REF)** | 0.0000 | . | . | . | . | . |
| **CIMD - Economic Dependency Q2** | 0.9526 | 1.2188 | 0.7800 | 0.4345 | -1.4366 | 3.3418 |
| **CIMD - Economic Dependency Q3** | 3.6945 | 1.2247 | 3.0200 | 0.0026 | 1.2937 | 6.0953 |
| **CIMD - Economic Dependency Q4** | 2.2044 | 1.2707 | 1.7300 | 0.0828 | -0.2866 | 4.6954 |
| **CIMD - Economic Dependency Q5 (most deprived)** | 2.5858 | 1.2966 | 1.9900 | 0.0461 | 0.0442 | 5.1274 |
| **CIMD - Economic Dependency Q1 (least deprived) (REF)** | 0.0000 | . | . | . | . | . |
| **CIMD - Ethnocultural Composition Q2** | 0.0097 | 0.7380 | 0.0100 | 0.9895 | -1.4370 | 1.4565 |
| **CIMD - Ethnocultural Composition Q3** | -0.6000 | 1.0616 | -0.5700 | 0.5720 | -2.6810 | 1.4810 |
| **CIMD - Ethnocultural Composition Q4** | 5.1849 | 1.6361 | 3.1700 | 0.0015 | 1.9777 | 8.3921 |
| **CIMD - Ethnocultural Composition Q5 (most deprived)** | 4.5385 | 2.7066 | 1.6800 | 0.0936 | -0.7671 | 9.8442 |
| **CIMD - Ethnocultural Composition Q1 (least deprived) (REF)** | 0.0000 | . | . | . | . | . |
| **CIMD -Situational Vulnerability Q2** | -2.9620 | 1.1456 | -2.5900 | 0.0097 | -5.2079 | -0.7162 |
| **CIMD - Situational Vulnerability Q3** | -2.9058 | 1.2880 | -2.2600 | 0.0241 | -5.4306 | -0.3811 |
| **CIMD -Situational Vulnerability Q4** | -3.0208 | 1.2555 | -2.4100 | 0.0161 | -5.4818 | -0.5597 |
| **CIMD -Situational Vulnerability Q5 (most deprived)** | -5.3010 | 1.3593 | -3.9000 | <.0001 | -7.9656 | -2.6364 |
| **CIMD - Situational Vulnerability Q1 (least deprived) (REF)** | 0.0000 | . | . | . | . | . |
| **Social Assistance (any in past 5 years)** | -7.7319 | 1.1224 | -6.8900 | <.0001 | -9.9322 | -5.5316 |
| **Social Assistance (none in past 5 years) (REF)** | 0.0000 | . | . | . | . | . |
| **Program of Study - French Immersion/Other** | 12.7487 | 0.8086 | 15.7700 | <.0001 | 11.1635 | 14.3339 |
| **Program of Study - French** | 41.0144 | 8.7307 | 4.7000 | <.0001 | 23.8997 | 58.1290 |
| **Program of Study - English (REF)** | 0.0000 | . | . | . | . | . |
| **Household composition – Adults (age 22+) – No adults in household** | -6.0843 | 8.7025 | -0.7000 | 0.4845 | -23.1437 | 10.9752 |
| **Household composition – Adults (age 22+) – One adult in household** | -2.4994 | 0.8472 | -2.9500 | 0.0032 | -4.1601 | -0.8387 |
| **Household composition – Adults (age 22+) – More than one adult in household (REF)** | 0.0000 | . | . | . | . | . |
| **Household composition – Children (age 21 or under) – Student is only child in household** | -4.1901 | 0.7716 | -5.4300 | <.0001 | -5.7026 | -2.6775 |
| **Household composition – Children (age 21 or under) – Other children in household (REF)** | 0.0000 | . | . | . | . | . |
| **Recent immigrant** | -3.2976 | 2.2956 | -1.4400 | 0.1509 | -7.7977 | 1.2024 |
| **Not a recent immigrant (REF)** | 0.0000 | . | . | . | . | . |

**Supplementary Table S7h. GLM regression estimates - Provincial assessment exams percentile rank for language for grades 9-11 (AY 2017-2019)**

| **Parameter** | **Estimate** | **Standard**  **Error** | **t Value** | **Pr > \|t\|** | **95% Confidence Limits** | |
| --- | --- | --- | --- | --- | --- | --- |
| **Intercept** | 49.7441 | 13.4296 | 3.7000 | 0.0002 | 23.4209 | 76.0673 |
| **Treated ADHD** | -8.4705 | 0.8199 | -10.3300 | <.0001 | -10.0776 | -6.8634 |
| **Untreated ADHD** | -8.9389 | 0.6113 | -14.6200 | <.0001 | -10.1372 | -7.7406 |
| **No ADHD (REF)** | 0.0000 | . | . | . | . | . |
| **Age** | -0.5919 | 0.3180 | -1.8600 | 0.0628 | -1.2153 | 0.0315 |
| **Male** | -4.6526 | 0.3980 | -11.6900 | <.0001 | -5.4327 | -3.8725 |
| **Female (REF)** | 0.0000 | . | . | . | . | . |
| **Household income quintile Q2** | 1.1544 | 0.7555 | 1.5300 | 0.1265 | -0.3264 | 2.6352 |
| **Household income quintile Q3** | 0.8922 | 0.8208 | 1.0900 | 0.2770 | -0.7166 | 2.5011 |
| **Household income quintile Q4** | 2.1891 | 0.8800 | 2.4900 | 0.0129 | 0.4642 | 3.9140 |
| **Household income quintile Q5 (highest income)** | 3.3783 | 0.9568 | 3.5300 | 0.0004 | 1.5028 | 5.2538 |
| **Household income quintile Q1 (lowest income) (REF)** | 0.0000 | . | . | . | . | . |
| **NB Health Zone 2** | 3.2088 | 0.5461 | 5.8800 | <.0001 | 2.1385 | 4.2791 |
| **NB Health Zone 3** | 4.0126 | 0.5435 | 7.3800 | <.0001 | 2.9473 | 5.0780 |
| **NB Health Zone 4** | -0.0276 | 1.3448 | -0.0200 | 0.9837 | -2.6635 | 2.6084 |
| **NB Health Zone 5** | 2.7130 | 1.4292 | 1.9000 | 0.0577 | -0.0883 | 5.5142 |
| **NB Health Zone 6** | 7.0578 | 1.1101 | 6.3600 | <.0001 | 4.8819 | 9.2336 |
| **NB Health Zone 7** | 3.3256 | 0.9279 | 3.5800 | 0.0003 | 1.5068 | 5.1444 |
| **NB Health Zone 1 (REF)** | 0.0000 | . | . | . | . | . |
| **Comorbid conditions - Mood & anxiety disorders (yes)** | -0.2192 | 0.8059 | -0.2700 | 0.7857 | -1.7988 | 1.3605 |
| **Comorbid conditions - Mood & anxiety disorders (no) (REF)** | 0.0000 | . | . | . | . | . |
| **Comorbid conditions – One or more of: asthma, diabetes, epilepsy, schizophrenia (yes)** | 4.2498 | 2.5098 | 1.6900 | 0.0904 | -0.6696 | 9.1691 |
| **Comorbid conditions – One or more of: asthma, diabetes, epilepsy, schizophrenia (no) (REF)** | 0.0000 | . | . | . | . | . |
| **Select medications (one or more)** | -3.0791 | 1.1842 | -2.6000 | 0.0093 | -5.4003 | -0.7579 |
| **Select medications (none) (REF)** | 0.0000 | . | . | . | . | . |
| **School District - Anglophone** | 7.2494 | 12.2379 | 0.5900 | 0.5536 | -16.7378 | 31.2367 |
| **School District – Francophone (REF)** | 0.0000 | . | . | . | . | . |
| **CIMD - Residential Instability Q2** | -0.5848 | 0.5736 | -1.0200 | 0.3079 | -1.7092 | 0.5395 |
| **CIMD - Residential Instability Q3** | -0.3296 | 0.6287 | -0.5200 | 0.6001 | -1.5618 | 0.9027 |
| **CIMD - Residential Instability Q4** | 1.5574 | 0.7175 | 2.1700 | 0.0300 | 0.1510 | 2.9638 |
| **CIMD – Residential Instability Q5 (most deprived)** | 2.9821 | 0.9891 | 3.0100 | 0.0026 | 1.0434 | 4.9208 |
| **CIMD - Residential Instability Q1 (least deprived) (REF)** | 0.0000 | . | . | . | . | . |
| **CIMD - Economic Dependency Q2** | 0.2817 | 0.7152 | 0.3900 | 0.6937 | -1.1202 | 1.6835 |
| **CIMD - Economic Dependency Q3** | 1.0656 | 0.7150 | 1.4900 | 0.1361 | -0.3358 | 2.4670 |
| **CIMD - Economic Dependency Q4** | 0.3314 | 0.7472 | 0.4400 | 0.6573 | -1.1331 | 1.7960 |
| **CIMD - Economic Dependency Q5 (most deprived)** | 1.8121 | 0.7660 | 2.3700 | 0.0180 | 0.3107 | 3.3135 |
| **CIMD - Economic Dependency Q1 (least deprived) (REF)** | 0.0000 | . | . | . | . | . |
| **CIMD - Ethnocultural Composition Q2** | -0.6384 | 0.4513 | -1.4100 | 0.1573 | -1.5230 | 0.2463 |
| **CIMD - Ethnocultural Composition Q3** | 1.0414 | 0.6433 | 1.6200 | 0.1055 | -0.2196 | 2.3024 |
| **CIMD - Ethnocultural Composition Q4** | 3.3155 | 0.9572 | 3.4600 | 0.0005 | 1.4393 | 5.1918 |
| **CIMD - Ethnocultural Composition Q5 (most deprived)** | 0.3647 | 1.5038 | 0.2400 | 0.8084 | -2.5829 | 3.3124 |
| **CIMD - Ethnocultural Composition Q1 (least deprived) (REF)** | 0.0000 | . | . | . | . | . |
| **CIMD -Situational Vulnerability Q2** | -2.6894 | 0.6650 | -4.0400 | <.0001 | -3.9929 | -1.3860 |
| **CIMD - Situational Vulnerability Q3** | -3.5612 | 0.7732 | -4.6100 | <.0001 | -5.0766 | -2.0457 |
| **CIMD -Situational Vulnerability Q4** | -3.6725 | 0.7512 | -4.8900 | <.0001 | -5.1449 | -2.2001 |
| **CIMD -Situational Vulnerability Q5 (most deprived)** | -5.4053 | 0.8301 | -6.5100 | <.0001 | -7.0323 | -3.7783 |
| **CIMD - Situational Vulnerability Q1 (least deprived) (REF)** | 0.0000 | . | . | . | . | . |
| **Social Assistance (any in past 5 years)** | -8.8978 | 0.6506 | -13.6800 | <.0001 | -10.1730 | -7.6225 |
| **Social Assistance (none in past 5 years) (REF)** | 0.0000 | . | . | . | . | . |
| **Program of Study - French Immersion/Other** | 10.5349 | 0.4444 | 23.7100 | <.0001 | 9.6639 | 11.4059 |
| **Program of Study - French** | 10.8474 | 12.2299 | 0.8900 | 0.3751 | -13.1242 | 34.8190 |
| **Program of Study - English (REF)** | 0.0000 | . | . | . | . | . |
| **Household composition – Adults (age 22+) – No adults in household** | -14.1762 | 5.7069 | -2.4800 | 0.0130 | -25.3621 | -2.9902 |
| **Household composition – Adults (age 22+) – One adult in household** | -2.6196 | 0.5175 | -5.0600 | <.0001 | -3.6339 | -1.6053 |
| **Household composition – Adults (age 22+) – More than one adult in household (REF)** | 0.0000 | . | . | . | . | . |
| **Household composition – Children (age 21 or under) – Student is only child in household** | 0.7883 | 0.4877 | 1.6200 | 0.1060 | -0.1675 | 1.7442 |
| **Household composition – Children (age 21 or under) – Other children in household (REF)** | 0.0000 | . | . | . | . | . |
| **Recent immigrant** | -7.9003 | 1.2952 | -6.1000 | <.0001 | -10.4390 | -5.3617 |
| **Not a recent immigrant (REF)** | 0.0000 | . | . | . | . | . |
