# Supplementary material for: Academic Outcomes in Primary and Secondary School Students Prescribed Long-Acting Stimulants for ADHD Management
Source: J Atten Disord. 2025 Oct 7;30(4):493–505. doi: 10.1177/10870547251378169 (PMC12953683; doi:10.1177/10870547251378169)
Supplement: sj-docx-8-jad-10.1177_10870547251378169 – Supplemental material for Academic Outcomes in Primary and Secondary School Students Prescribed Long-Acting Stimulants for ADHD Management [file sj-docx-8-jad-10.1177_10870547251378169.docx]

**Supplementary Table S8a. GLM regression estimates – Provincial assessment exams overall percentile rank for grades K-8 (AY 2017-2019) (Untreated group as reference)**

| **Parameter** | **Estimate** | **Standard**  **Error** | **t Value** | **Pr > \|t\|** | **95% Confidence Limits** | |
| --- | --- | --- | --- | --- | --- | --- |
| **Intercept** | 6.3531 | 6.2837 | 1.0100 | 0.3120 | -5.9646 | 18.6708 |
| **Treated ADHD** | 0.2792 | 0.6436 | 0.4300 | 0.6644 | -0.9825 | 1.5410 |
| **Untreated ADHD (REF)** | 0.0000 | . | . | . | . | . |
| **Age** | -0.1440 | 0.1124 | -1.2800 | 0.2001 | -0.3643 | 0.0763 |
| **Male** | 2.5103 | 0.6710 | 3.7400 | 0.0002 | 1.1950 | 3.8255 |
| **Female (REF)** | 0.0000 | . | . | . | . | . |
| **Household income quintile Q2** | -0.3042 | 1.1411 | -0.2700 | 0.7898 | -2.5411 | 1.9326 |
| **Household income quintile Q3** | -1.0392 | 1.2408 | -0.8400 | 0.4023 | -3.4715 | 1.3931 |
| **Household income quintile Q4** | 0.2408 | 1.3242 | 0.1800 | 0.8557 | -2.3551 | 2.8367 |
| **Household income quintile Q5 (highest income)** | -0.2035 | 1.4626 | -0.1400 | 0.8894 | -3.0705 | 2.6636 |
| **Household income quintile Q1 (lowest income) (REF)** | 0.0000 | . | . | . | . | . |
| **NB Health Zone 2** | 3.7343 | 0.9612 | 3.8800 | 0.0001 | 1.8500 | 5.6186 |
| **NB Health Zone 3** | 3.9193 | 0.9551 | 4.1000 | <.0001 | 2.0471 | 5.7916 |
| **NB Health Zone 4** | 1.1117 | 1.4248 | 0.7800 | 0.4353 | -1.6813 | 3.9046 |
| **NB Health Zone 5** | 2.8521 | 1.8314 | 1.5600 | 0.1194 | -0.7380 | 6.4422 |
| **NB Health Zone 6** | 2.9400 | 1.2158 | 2.4200 | 0.0156 | 0.5566 | 5.3233 |
| **NB Health Zone 7** | -0.5105 | 1.6215 | -0.3100 | 0.7529 | -3.6891 | 2.6681 |
| **NB Health Zone 1 (REF)** | 0.0000 | . | . | . | . | . |
| **Comorbid conditions - Mood & anxiety disorders (yes)** | 0.6052 | 1.1616 | 0.5200 | 0.6024 | -1.6719 | 2.8824 |
| **Comorbid conditions - Mood & anxiety disorders (no) (REF)** | 0.0000 | . | . | . | . | . |
| **Comorbid conditions – One or more of: asthma, diabetes, epilepsy, schizophrenia (yes)** | 2.3920 | 3.3137 | 0.7200 | 0.4704 | -4.1037 | 8.8878 |
| **Comorbid conditions – One or more of: asthma, diabetes, epilepsy, schizophrenia (no) (REF)** | 0.0000 | . | . | . | . | . |
| **Select medications (one or more)** | -1.8926 | 1.0318 | -1.8300 | 0.0667 | -3.9152 | 0.1300 |
| **Select medications (none) (REF)** | 0.0000 | . | . | . | . | . |
| **School District - Anglophone** | 32.7093 | 5.6965 | 5.7400 | <.0001 | 21.5425 | 43.8760 |
| **School District – Francophone (REF)** | 0.0000 | . | . | . | . | . |
| **CIMD - Residential Instability Q2** | -0.1995 | 0.9483 | -0.2100 | 0.8334 | -2.0584 | 1.6595 |
| **CIMD - Residential Instability Q3** | -0.0418 | 0.9932 | -0.0400 | 0.9664 | -1.9888 | 1.9052 |
| **CIMD - Residential Instability Q4** | 0.4274 | 1.1314 | 0.3800 | 0.7056 | -1.7905 | 2.6453 |
| **CIMD – Residential Instability Q5 (most deprived)** | 1.8807 | 1.5147 | 1.2400 | 0.2144 | -1.0885 | 4.8499 |
| **CIMD - Residential Instability Q1 (least deprived) (REF)** | 0.0000 | . | . | . | . | . |
| **CIMD - Economic Dependency Q2** | -1.4878 | 1.2125 | -1.2300 | 0.2198 | -3.8646 | 0.8890 |
| **CIMD - Economic Dependency Q3** | 1.3915 | 1.2154 | 1.1400 | 0.2523 | -0.9911 | 3.7741 |
| **CIMD - Economic Dependency Q4** | -0.9730 | 1.2504 | -0.7800 | 0.4365 | -3.4242 | 1.4782 |
| **CIMD - Economic Dependency Q5 (most deprived)** | -0.2419 | 1.2853 | -0.1900 | 0.8507 | -2.7615 | 2.2776 |
| **CIMD - Economic Dependency Q1 (least deprived) (REF)** | 0.0000 | . | . | . | . | . |
| **CIMD - Ethnocultural Composition Q2** | -0.4236 | 0.7139 | -0.5900 | 0.5529 | -1.8232 | 0.9759 |
| **CIMD - Ethnocultural Composition Q3** | 0.2319 | 1.0335 | 0.2200 | 0.8225 | -1.7941 | 2.2579 |
| **CIMD - Ethnocultural Composition Q4** | 1.2183 | 1.5676 | 0.7800 | 0.4371 | -1.8546 | 4.2912 |
| **CIMD - Ethnocultural Composition Q5 (most deprived)** | -1.0175 | 2.4240 | -0.4200 | 0.6747 | -5.7692 | 3.7342 |
| **CIMD - Ethnocultural Composition Q1 (least deprived) (REF)** | 0.0000 | . | . | . | . | . |
| **CIMD -Situational Vulnerability Q2** | -2.9825 | 1.1832 | -2.5200 | 0.0117 | -5.3019 | -0.6631 |
| **CIMD - Situational Vulnerability Q3** | -5.9202 | 1.3105 | -4.5200 | <.0001 | -8.4892 | -3.3512 |
| **CIMD -Situational Vulnerability Q4** | -5.9338 | 1.2755 | -4.6500 | <.0001 | -8.4341 | -3.4335 |
| **CIMD -Situational Vulnerability Q5 (most deprived)** | -6.6400 | 1.3595 | -4.8800 | <.0001 | -9.3049 | -3.9750 |
| **CIMD - Situational Vulnerability Q1 (least deprived) (REF)** | 0.0000 | . | . | . | . | . |
| **Social Assistance (any in past 5 years)** | -6.3290 | 0.8410 | -7.5300 | <.0001 | -7.9777 | -4.6804 |
| **Social Assistance (none in past 5 years) (REF)** | 0.0000 | . | . | . | . | . |
| **Program of Study - French Immersion/Other** | 12.6739 | 0.9675 | 13.1000 | <.0001 | 10.7773 | 14.5705 |
| **Program of Study - French** | 35.6966 | 5.7087 | 6.2500 | <.0001 | 24.5059 | 46.8872 |
| **Program of Study - English (REF)** | 0.0000 | . | . | . | . | . |
| **Household composition – Adults (age 22+) – No adults in household** | -12.7785 | 4.6084 | -2.7700 | 0.0056 | -21.8121 | -3.7448 |
| **Household composition – Adults (age 22+) – One adult in household** | -2.4157 | 0.7084 | -3.4100 | 0.0007 | -3.8044 | -1.0269 |
| **Household composition – Adults (age 22+) – More than one adult in household (REF)** | 0.0000 | . | . | . | . | . |
| **Household composition – Children (age 21 or under) – Student is only child in household** | -0.7529 | 0.7447 | -1.0100 | 0.3120 | -2.2127 | 0.7068 |
| **Household composition – Children (age 21 or under) – Other children in household (REF)** | 0.0000 | . | . | . | . | . |
| **Recent immigrant** | 1.2530 | 6.1739 | 0.2000 | 0.8392 | -10.8495 | 13.3554 |
| **Not a recent immigrant (REF)** | 0.0000 | . | . | . | . | . |

**Supplementary Table S8b. GLM regression estimates - Provincial assessment exams percentile rank for STEM subjects for grades K-8 (AY 2017-2019) (Untreated group as reference)**

| **Parameter** | **Estimate** | **Standard**  **Error** | **t Value** | **Pr > \|t\|** | **95% Confidence Limits** | |
| --- | --- | --- | --- | --- | --- | --- |
| **Intercept** | 0.5343 | 6.7844 | 0.0800 | 0.9372 | -12.7660 | 13.8346 |
| **Treated ADHD** | 0.9785 | 0.7769 | 1.2600 | 0.2079 | -0.5446 | 2.5016 |
| **Untreated ADHD (REF)** | 0.0000 | . | . | . | . | . |
| **Age** | -0.0031 | 0.1475 | -0.0200 | 0.9832 | -0.2922 | 0.2860 |
| **Male** | 5.2013 | 0.8240 | 6.3100 | <.0001 | 3.5859 | 6.8168 |
| **Female (REF)** | 0.0000 | . | . | . | . | . |
| **Household income quintile Q2** | -0.5806 | 1.4052 | -0.4100 | 0.6795 | -3.3353 | 2.1742 |
| **Household income quintile Q3** | -0.0606 | 1.5067 | -0.0400 | 0.9679 | -3.0143 | 2.8932 |
| **Household income quintile Q4** | -0.6108 | 1.6069 | -0.3800 | 0.7039 | -3.7609 | 2.5394 |
| **Household income quintile Q5 (highest income)** | 1.3667 | 1.7599 | 0.7800 | 0.4374 | -2.0834 | 4.8169 |
| **Household income quintile Q1 (lowest income) (REF)** | 0.0000 | . | . | . | . | . |
| **NB Health Zone 2** | 3.5951 | 1.2390 | 2.9000 | 0.0037 | 1.1661 | 6.0241 |
| **NB Health Zone 3** | 4.5684 | 1.2136 | 3.7600 | 0.0002 | 2.1892 | 6.9476 |
| **NB Health Zone 4** | 3.3420 | 1.6130 | 2.0700 | 0.0383 | 0.1798 | 6.5043 |
| **NB Health Zone 5** | 0.4017 | 2.1607 | 0.1900 | 0.8525 | -3.8341 | 4.6375 |
| **NB Health Zone 6** | 2.7705 | 1.3975 | 1.9800 | 0.0475 | 0.0309 | 5.5101 |
| **NB Health Zone 7** | 0.6422 | 1.9823 | 0.3200 | 0.7460 | -3.2439 | 4.5284 |
| **NB Health Zone 1 (REF)** | 0.0000 | . | . | . | . | . |
| **Comorbid conditions - Mood & anxiety disorders (yes)** | -0.7602 | 1.4140 | -0.5400 | 0.5909 | -3.5322 | 2.0118 |
| **Comorbid conditions - Mood & anxiety disorders (no) (REF)** | 0.0000 | . | . | . | . | . |
| **Comorbid conditions – One or more of: asthma, diabetes, epilepsy, schizophrenia (yes)** | -1.7659 | 4.1745 | -0.4200 | 0.6723 | -9.9497 | 6.4179 |
| **Comorbid conditions – One or more of: asthma, diabetes, epilepsy, schizophrenia (no) (REF)** | 0.0000 | . | . | . | . | . |
| **Select medications (one or more)** | -2.2524 | 1.2916 | -1.7400 | 0.0812 | -4.7844 | 0.2797 |
| **Select medications (none) (REF)** | 0.0000 | . | . | . | . | . |
| **School District - Anglophone** | 33.6193 | 5.8987 | 5.7000 | <.0001 | 22.0553 | 45.1833 |
| **School District – Francophone (REF)** | 0.0000 | . | . | . | . | . |
| **CIMD - Residential Instability Q2** | -1.0781 | 1.1437 | -0.9400 | 0.3459 | -3.3202 | 1.1639 |
| **CIMD - Residential Instability Q3** | -1.7338 | 1.1930 | -1.4500 | 0.1462 | -4.0726 | 0.6051 |
| **CIMD - Residential Instability Q4** | 0.3319 | 1.3744 | 0.2400 | 0.8092 | -2.3626 | 3.0263 |
| **CIMD – Residential Instability Q5 (most deprived)** | 1.9639 | 1.9065 | 1.0300 | 0.3030 | -1.7736 | 5.7013 |
| **CIMD - Residential Instability Q1 (least deprived) (REF)** | 0.0000 | . | . | . | . | . |
| **CIMD - Economic Dependency Q2** | 0.3264 | 1.5085 | 0.2200 | 0.8287 | -2.6309 | 3.2836 |
| **CIMD - Economic Dependency Q3** | 2.8447 | 1.5164 | 1.8800 | 0.0607 | -0.1282 | 5.8176 |
| **CIMD - Economic Dependency Q4** | 0.1378 | 1.5497 | 0.0900 | 0.9291 | -2.9002 | 3.1758 |
| **CIMD - Economic Dependency Q5 (most deprived)** | -0.3454 | 1.5894 | -0.2200 | 0.8280 | -3.4612 | 2.7705 |
| **CIMD - Economic Dependency Q1 (least deprived) (REF)** | 0.0000 | . | . | . | . | . |
| **CIMD - Ethnocultural Composition Q2** | -0.4496 | 0.8627 | -0.5200 | 0.6023 | -2.1408 | 1.2417 |
| **CIMD - Ethnocultural Composition Q3** | -0.8686 | 1.2682 | -0.6800 | 0.4934 | -3.3547 | 1.6176 |
| **CIMD - Ethnocultural Composition Q4** | 1.2900 | 2.0138 | 0.6400 | 0.5218 | -2.6578 | 5.2378 |
| **CIMD - Ethnocultural Composition Q5 (most deprived)** | 2.5227 | 3.3772 | 0.7500 | 0.4551 | -4.0981 | 9.1435 |
| **CIMD - Ethnocultural Composition Q1 (least deprived) (REF)** | 0.0000 | . | . | . | . | . |
| **CIMD -Situational Vulnerability Q2** | -1.6171 | 1.4708 | -1.1000 | 0.2716 | -4.5005 | 1.2663 |
| **CIMD - Situational Vulnerability Q3** | -3.4891 | 1.6117 | -2.1600 | 0.0304 | -6.6486 | -0.3295 |
| **CIMD -Situational Vulnerability Q4** | -4.3733 | 1.5718 | -2.7800 | 0.0054 | -7.4547 | -1.2920 |
| **CIMD -Situational Vulnerability Q5 (most deprived)** | -4.9476 | 1.6609 | -2.9800 | 0.0029 | -8.2037 | -1.6915 |
| **CIMD - Situational Vulnerability Q1 (least deprived) (REF)** | 0.0000 | . | . | . | . | . |
| **Social Assistance (any in past 5 years)** | -5.9950 | 1.0664 | -5.6200 | <.0001 | -8.0856 | -3.9044 |
| **Social Assistance (none in past 5 years) (REF)** | 0.0000 | . | . | . | . | . |
| **Program of Study - French Immersion/Other** | 10.9341 | 1.2175 | 8.9800 | <.0001 | 8.5474 | 13.3209 |
| **Program of Study - French** | 36.2954 | 5.9219 | 6.1300 | <.0001 | 24.6859 | 47.9049 |
| **Program of Study - English (REF)** | 0.0000 | . | . | . | . | . |
| **Household composition – Adults (age 22+) – No adults in household** | -13.3841 | 6.2658 | -2.1400 | 0.0327 | -25.6677 | -1.1005 |
| **Household composition – Adults (age 22+) – One adult in household** | -1.6246 | 0.8585 | -1.8900 | 0.0585 | -3.3077 | 0.0585 |
| **Household composition – Adults (age 22+) – More than one adult in household (REF)** | 0.0000 | . | . | . | . | . |
| **Household composition – Children (age 21 or under) – Student is only child in household** | -3.4845 | 0.9075 | -3.8400 | 0.0001 | -5.2636 | -1.7054 |
| **Household composition – Children (age 21 or under) – Other children in household (REF)** | 0.0000 | . | . | . | . | . |
| **Recent immigrant** | 13.1851 | 8.1665 | 1.6100 | 0.1065 | -2.8248 | 29.1949 |
| **Not a recent immigrant (REF)** | 0.0000 | . | . | . | . | . |

**Supplementary Table S8c. GLM regression estimates - Provincial assessment exams percentile rank for math for grades K-8 (AY 2017-2019) (Untreated group as reference)**

| **Parameter** | **Estimate** | **Standard**  **Error** | **t Value** | **Pr > \|t\|** | **95% Confidence Limits** | |
| --- | --- | --- | --- | --- | --- | --- |
| **Intercept** | 12.9459 | 8.5009 | 1.5200 | 0.1279 | -3.7217 | 29.6135 |
| **Treated ADHD** | -0.5283 | 0.9663 | -0.5500 | 0.5846 | -2.4229 | 1.3663 |
| **Untreated ADHD (REF)** | 0.0000 | . | . | . | . | . |
| **Age** | -0.2392 | 0.2351 | -1.0200 | 0.3091 | -0.7001 | 0.2218 |
| **Male** | 5.5781 | 1.0351 | 5.3900 | <.0001 | 3.5486 | 7.6076 |
| **Female (REF)** | 0.0000 | . | . | . | . | . |
| **Household income quintile Q2** | -1.3188 | 1.7608 | -0.7500 | 0.4539 | -4.7712 | 2.1336 |
| **Household income quintile Q3** | -0.9825 | 1.8792 | -0.5200 | 0.6011 | -4.6670 | 2.7020 |
| **Household income quintile Q4** | -2.7875 | 2.0144 | -1.3800 | 0.1665 | -6.7370 | 1.1620 |
| **Household income quintile Q5 (highest income)** | -1.5346 | 2.2079 | -0.7000 | 0.4871 | -5.8636 | 2.7943 |
| **Household income quintile Q1 (lowest income) (REF)** | 0.0000 | . | . | . | . | . |
| **NB Health Zone 2** | 3.7624 | 1.7181 | 2.1900 | 0.0286 | 0.3938 | 7.1310 |
| **NB Health Zone 3** | 1.0276 | 1.6005 | 0.6400 | 0.5209 | -2.1105 | 4.1657 |
| **NB Health Zone 4** | 2.8794 | 1.8653 | 1.5400 | 0.1228 | -0.7778 | 6.5366 |
| **NB Health Zone 5** | 3.3656 | 2.6900 | 1.2500 | 0.2110 | -1.9087 | 8.6399 |
| **NB Health Zone 6** | 4.7138 | 1.6635 | 2.8300 | 0.0046 | 1.4523 | 7.9753 |
| **NB Health Zone 7** | -2.1131 | 2.5711 | -0.8200 | 0.4112 | -7.1543 | 2.9281 |
| **NB Health Zone 1 (REF)** | 0.0000 | . | . | . | . | . |
| **Comorbid conditions - Mood & anxiety disorders (yes)** | -0.0065 | 2.1312 | 0.0000 | 0.9976 | -4.1850 | 4.1721 |
| **Comorbid conditions - Mood & anxiety disorders (no) (REF)** | 0.0000 | . | . | . | . | . |
| **Comorbid conditions – One or more of: asthma, diabetes, epilepsy, schizophrenia (yes)** | -1.7273 | 5.6595 | -0.3100 | 0.7602 | -12.8239 | 9.3692 |
| **Comorbid conditions – One or more of: asthma, diabetes, epilepsy, schizophrenia (no) (REF)** | 0.0000 | . | . | . | . | . |
| **Select medications (one or more)** | -1.0202 | 1.5878 | -0.6400 | 0.5206 | -4.1333 | 2.0930 |
| **Select medications (none) (REF)** | 0.0000 | . | . | . | . | . |
| **School District - Anglophone** | 31.0906 | 7.2105 | 4.3100 | <.0001 | 16.9531 | 45.2281 |
| **School District – Francophone (REF)** | 0.0000 | . | . | . | . | . |
| **CIMD - Residential Instability Q2** | -1.1476 | 1.4594 | -0.7900 | 0.4317 | -4.0091 | 1.7139 |
| **CIMD - Residential Instability Q3** | -1.4446 | 1.4980 | -0.9600 | 0.3350 | -4.3817 | 1.4926 |
| **CIMD - Residential Instability Q4** | -0.3427 | 1.7311 | -0.2000 | 0.8431 | -3.7367 | 3.0514 |
| **CIMD – Residential Instability Q5 (most deprived)** | -2.0544 | 2.3764 | -0.8600 | 0.3874 | -6.7137 | 2.6050 |
| **CIMD - Residential Instability Q1 (least deprived) (REF)** | 0.0000 | . | . | . | . | . |
| **CIMD - Economic Dependency Q2** | -2.5081 | 1.9007 | -1.3200 | 0.1871 | -6.2348 | 1.2186 |
| **CIMD - Economic Dependency Q3** | -0.6420 | 1.9146 | -0.3400 | 0.7374 | -4.3959 | 3.1119 |
| **CIMD - Economic Dependency Q4** | -3.5227 | 1.9504 | -1.8100 | 0.0710 | -7.3468 | 0.3014 |
| **CIMD - Economic Dependency Q5 (most deprived)** | -5.2900 | 2.0120 | -2.6300 | 0.0086 | -9.2348 | -1.3451 |
| **CIMD - Economic Dependency Q1 (least deprived) (REF)** | 0.0000 | . | . | . | . | . |
| **CIMD - Ethnocultural Composition Q2** | -0.6315 | 1.0851 | -0.5800 | 0.5606 | -2.7591 | 1.4960 |
| **CIMD - Ethnocultural Composition Q3** | 0.3840 | 1.5966 | 0.2400 | 0.8100 | -2.7464 | 3.5143 |
| **CIMD - Ethnocultural Composition Q4** | -0.9044 | 2.4935 | -0.3600 | 0.7169 | -5.7934 | 3.9847 |
| **CIMD - Ethnocultural Composition Q5 (most deprived)** | 2.3129 | 4.1605 | 0.5600 | 0.5783 | -5.8444 | 10.4703 |
| **CIMD - Ethnocultural Composition Q1 (least deprived) (REF)** | 0.0000 | . | . | . | . | . |
| **CIMD -Situational Vulnerability Q2** | -0.9376 | 1.8825 | -0.5000 | 0.6185 | -4.6286 | 2.7534 |
| **CIMD - Situational Vulnerability Q3** | -3.4063 | 2.0446 | -1.6700 | 0.0958 | -7.4150 | 0.6025 |
| **CIMD -Situational Vulnerability Q4** | -3.5378 | 2.0030 | -1.7700 | 0.0775 | -7.4651 | 0.3896 |
| **CIMD -Situational Vulnerability Q5 (most deprived)** | -4.0724 | 2.1227 | -1.9200 | 0.0551 | -8.2343 | 0.0895 |
| **CIMD - Situational Vulnerability Q1 (least deprived) (REF)** | 0.0000 | . | . | . | . | . |
| **Social Assistance (any in past 5 years)** | -7.1777 | 1.2973 | -5.5300 | <.0001 | -9.7214 | -4.6340 |
| **Social Assistance (none in past 5 years) (REF)** | 0.0000 | . | . | . | . | . |
| **Program of Study - French Immersion/Other** | 8.5172 | 1.6902 | 5.0400 | <.0001 | 5.2033 | 11.8312 |
| **Program of Study - French** | 31.4390 | 7.2442 | 4.3400 | <.0001 | 17.2353 | 45.6426 |
| **Program of Study - English (REF)** | 0.0000 | . | . | . | . | . |
| **Household composition – Adults (age 22+) – No adults in household** | -13.1886 | 7.1251 | -1.8500 | 0.0643 | -27.1587 | 0.7816 |
| **Household composition – Adults (age 22+) – One adult in household** | -3.0024 | 1.0530 | -2.8500 | 0.0044 | -5.0669 | -0.9379 |
| **Household composition – Adults (age 22+) – More than one adult in household (REF)** | 0.0000 | . | . | . | . | . |
| **Household composition – Children (age 21 or under) – Student is only child in household** | -2.8749 | 1.1712 | -2.4500 | 0.0142 | -5.1713 | -0.5785 |
| **Household composition – Children (age 21 or under) – Other children in household (REF)** | 0.0000 | . | . | . | . | . |
| **Recent immigrant** | 16.9361 | 10.2538 | 1.6500 | 0.0987 | -3.1683 | 37.0405 |
| **Not a recent immigrant (REF)** | 0.0000 | . | . | . | . | . |

**Supplementary Table S8d. GLM regression estimates - Provincial assessment exams percentile rank for language for grades K-8 (AY 2017-2019) (Untreated group as reference)**

| **Parameter** | **Estimate** | **Standard**  **Error** | **t Value** | **Pr > \|t\|** | **95% Confidence Limits** | |
| --- | --- | --- | --- | --- | --- | --- |
| **Intercept** | 52.4637 | 16.0925 | 3.2600 | 0.0011 | 20.9164 | 84.0110 |
| **Treated ADHD** | -0.1212 | 0.7602 | -0.1600 | 0.8733 | -1.6115 | 1.3690 |
| **Untreated ADHD (REF)** | 0.0000 | . | . | . | . | . |
| **Age** | -0.0985 | 0.1306 | -0.7500 | 0.4511 | -0.3546 | 0.1577 |
| **Male** | -0.0220 | 0.7726 | -0.0300 | 0.9773 | -1.5366 | 1.4926 |
| **Female (REF)** | 0.0000 | . | . | . | . | . |
| **Household income quintile Q2** | 0.5612 | 1.3167 | 0.4300 | 0.6699 | -2.0200 | 3.1425 |
| **Household income quintile Q3** | -0.6814 | 1.4700 | -0.4600 | 0.6430 | -3.5632 | 2.2003 |
| **Household income quintile Q4** | 1.6916 | 1.5596 | 1.0800 | 0.2781 | -1.3658 | 4.7490 |
| **Household income quintile Q5 (highest income)** | 0.1077 | 1.7362 | 0.0600 | 0.9505 | -3.2958 | 3.5112 |
| **Household income quintile Q1 (lowest income) (REF)** | 0.0000 | . | . | . | . | . |
| **NB Health Zone 2** | 4.0610 | 1.0042 | 4.0400 | <.0001 | 2.0923 | 6.0296 |
| **NB Health Zone 3** | 4.4175 | 1.0314 | 4.2800 | <.0001 | 2.3956 | 6.4395 |
| **NB Health Zone 4** | -4.2863 | 2.3854 | -1.8000 | 0.0724 | -8.9625 | 0.3900 |
| **NB Health Zone 5** | 3.3750 | 2.2583 | 1.4900 | 0.1351 | -1.0521 | 7.8020 |
| **NB Health Zone 6** | 4.0783 | 1.8138 | 2.2500 | 0.0246 | 0.5226 | 7.6341 |
| **NB Health Zone 7** | 1.9243 | 1.8514 | 1.0400 | 0.2987 | -1.7050 | 5.5537 |
| **NB Health Zone 1 (REF)** | 0.0000 | . | . | . | . | . |
| **Comorbid conditions - Mood & anxiety disorders (yes)** | 0.8861 | 1.3194 | 0.6700 | 0.5019 | -1.7004 | 3.4726 |
| **Comorbid conditions - Mood & anxiety disorders (no) (REF)** | 0.0000 | . | . | . | . | . |
| **Comorbid conditions – One or more of: asthma, diabetes, epilepsy, schizophrenia (yes)** | 0.4037 | 3.8038 | 0.1100 | 0.9155 | -7.0531 | 7.8605 |
| **Comorbid conditions – One or more of: asthma, diabetes, epilepsy, schizophrenia (no) (REF)** | 0.0000 | . | . | . | . | . |
| **Select medications (one or more)** | -1.8131 | 1.2071 | -1.5000 | 0.1332 | -4.1795 | 0.5534 |
| **Select medications (none) (REF)** | 0.0000 | . | . | . | . | . |
| **School District - Anglophone** | -14.1124 | 15.8223 | -0.8900 | 0.3725 | -45.1300 | 16.9052 |
| **School District – Francophone (REF)** | 0.0000 | . | . | . | . | . |
| **CIMD - Residential Instability Q2** | 0.3269 | 1.0987 | 0.3000 | 0.7661 | -1.8270 | 2.4807 |
| **CIMD - Residential Instability Q3** | 1.6709 | 1.1719 | 1.4300 | 0.1540 | -0.6265 | 3.9684 |
| **CIMD - Residential Instability Q4** | 1.0565 | 1.2966 | 0.8100 | 0.4152 | -1.4854 | 3.5984 |
| **CIMD – Residential Instability Q5 (most deprived)** | 2.6261 | 1.6943 | 1.5500 | 0.1212 | -0.6954 | 5.9476 |
| **CIMD - Residential Instability Q1 (least deprived) (REF)** | 0.0000 | . | . | . | . | . |
| **CIMD - Economic Dependency Q2** | -0.0556 | 1.3628 | -0.0400 | 0.9674 | -2.7273 | 2.6160 |
| **CIMD - Economic Dependency Q3** | 1.7046 | 1.3686 | 1.2500 | 0.2130 | -0.9783 | 4.3875 |
| **CIMD - Economic Dependency Q4** | -0.7391 | 1.4134 | -0.5200 | 0.6010 | -3.5100 | 2.0317 |
| **CIMD - Economic Dependency Q5 (most deprived)** | 2.8676 | 1.4454 | 1.9800 | 0.0473 | 0.0341 | 5.7011 |
| **CIMD - Economic Dependency Q1 (least deprived) (REF)** | 0.0000 | . | . | . | . | . |
| **CIMD - Ethnocultural Composition Q2** | -0.1364 | 0.8358 | -0.1600 | 0.8703 | -1.7749 | 1.5021 |
| **CIMD - Ethnocultural Composition Q3** | -0.3481 | 1.1921 | -0.2900 | 0.7703 | -2.6850 | 1.9889 |
| **CIMD - Ethnocultural Composition Q4** | 0.7260 | 1.7524 | 0.4100 | 0.6787 | -2.7094 | 4.1614 |
| **CIMD - Ethnocultural Composition Q5 (most deprived)** | -2.6318 | 2.5514 | -1.0300 | 0.3024 | -7.6334 | 2.3699 |
| **CIMD - Ethnocultural Composition Q1 (least deprived) (REF)** | 0.0000 | . | . | . | . | . |
| **CIMD -Situational Vulnerability Q2** | -4.0235 | 1.3036 | -3.0900 | 0.0020 | -6.5791 | -1.4680 |
| **CIMD - Situational Vulnerability Q3** | -7.5930 | 1.4834 | -5.1200 | <.0001 | -10.5011 | -4.6849 |
| **CIMD -Situational Vulnerability Q4** | -6.9541 | 1.4389 | -4.8300 | <.0001 | -9.7750 | -4.1332 |
| **CIMD -Situational Vulnerability Q5 (most deprived)** | -7.0227 | 1.5527 | -4.5200 | <.0001 | -10.0665 | -3.9789 |
| **CIMD - Situational Vulnerability Q1 (least deprived) (REF)** | 0.0000 | . | . | . | . | . |
| **Social Assistance (any in past 5 years)** | -4.2537 | 0.9489 | -4.4800 | <.0001 | -6.1138 | -2.3935 |
| **Social Assistance (none in past 5 years) (REF)** | 0.0000 | . | . | . | . | . |
| **Program of Study - French Immersion/Other** | 12.3072 | 0.9424 | 13.0600 | <.0001 | 10.4598 | 14.1547 |
| **Program of Study - French** | -8.8828 | 15.8170 | -0.5600 | 0.5744 | -39.8900 | 22.1243 |
| **Program of Study - English (REF)** | 0.0000 | . | . | . | . | . |
| **Household composition – Adults (age 22+) – No adults in household** | -13.9537 | 5.6638 | -2.4600 | 0.0138 | -25.0569 | -2.8506 |
| **Household composition – Adults (age 22+) – One adult in household** | -2.1195 | 0.8417 | -2.5200 | 0.0118 | -3.7696 | -0.4694 |
| **Household composition – Adults (age 22+) – More than one adult in household (REF)** | 0.0000 | . | . | . | . | . |
| **Household composition – Children (age 21 or under) – Student is only child in household** | 1.1343 | 0.8653 | 1.3100 | 0.1900 | -0.5620 | 2.8306 |
| **Household composition – Children (age 21 or under) – Other children in household (REF)** | 0.0000 | . | . | . | . | . |
| **Recent immigrant** | 8.0503 | 5.8592 | 1.3700 | 0.1695 | -3.4360 | 19.5365 |
| **Not a recent immigrant (REF)** | 0.0000 | . | . | . | . | . |

**Supplementary Table S8e. GLM regression estimates - Provincial assessment exams overall percentile rank for grades 9-11 (AY 2017-2019) (Untreated group as reference)**

| **Parameter** | **Estimate** | **Standard**  **Error** | **t Value** | **Pr > \|t\|** | **95% Confidence Limits** | |
| --- | --- | --- | --- | --- | --- | --- |
| **Intercept** | 6.3531 | 6.2837 | 1.0100 | 0.3120 | -5.9646 | 18.6708 |
| **Treated ADHD** | 0.2792 | 0.6436 | 0.4300 | 0.6644 | -0.9825 | 1.5410 |
| **Untreated ADHD (REF)** | 0.0000 | . | . | . | . | . |
| **Age** | -0.1440 | 0.1124 | -1.2800 | 0.2001 | -0.3643 | 0.0763 |
| **Male** | 2.5103 | 0.6710 | 3.7400 | 0.0002 | 1.1950 | 3.8255 |
| **Female (REF)** | 0.0000 | . | . | . | . | . |
| **Household income quintile Q2** | -0.3042 | 1.1411 | -0.2700 | 0.7898 | -2.5411 | 1.9326 |
| **Household income quintile Q3** | -1.0392 | 1.2408 | -0.8400 | 0.4023 | -3.4715 | 1.3931 |
| **Household income quintile Q4** | 0.2408 | 1.3242 | 0.1800 | 0.8557 | -2.3551 | 2.8367 |
| **Household income quintile Q5 (highest income)** | -0.2035 | 1.4626 | -0.1400 | 0.8894 | -3.0705 | 2.6636 |
| **Household income quintile Q1 (lowest income) (REF)** | 0.0000 | . | . | . | . | . |
| **NB Health Zone 2** | 3.7343 | 0.9612 | 3.8800 | 0.0001 | 1.8500 | 5.6186 |
| **NB Health Zone 3** | 3.9193 | 0.9551 | 4.1000 | <.0001 | 2.0471 | 5.7916 |
| **NB Health Zone 4** | 1.1117 | 1.4248 | 0.7800 | 0.4353 | -1.6813 | 3.9046 |
| **NB Health Zone 5** | 2.8521 | 1.8314 | 1.5600 | 0.1194 | -0.7380 | 6.4422 |
| **NB Health Zone 6** | 2.9400 | 1.2158 | 2.4200 | 0.0156 | 0.5566 | 5.3233 |
| **NB Health Zone 7** | -0.5105 | 1.6215 | -0.3100 | 0.7529 | -3.6891 | 2.6681 |
| **NB Health Zone 1 (REF)** | 0.0000 | . | . | . | . | . |
| **Comorbid conditions - Mood & anxiety disorders (yes)** | 0.6052 | 1.1616 | 0.5200 | 0.6024 | -1.6719 | 2.8824 |
| **Comorbid conditions - Mood & anxiety disorders (no) (REF)** | 0.0000 | . | . | . | . | . |
| **Comorbid conditions – One or more of: asthma, diabetes, epilepsy, schizophrenia (yes)** | 2.3920 | 3.3137 | 0.7200 | 0.4704 | -4.1037 | 8.8878 |
| **Comorbid conditions – One or more of: asthma, diabetes, epilepsy, schizophrenia (no) (REF)** | 0.0000 | . | . | . | . | . |
| **Select medications (one or more)** | -1.8926 | 1.0318 | -1.8300 | 0.0667 | -3.9152 | 0.1300 |
| **Select medications (none) (REF)** | 0.0000 | . | . | . | . | . |
| **School District - Anglophone** | 32.7093 | 5.6965 | 5.7400 | <.0001 | 21.5425 | 43.8760 |
| **School District – Francophone (REF)** | 0.0000 | . | . | . | . | . |
| **CIMD - Residential Instability Q2** | -0.1995 | 0.9483 | -0.2100 | 0.8334 | -2.0584 | 1.6595 |
| **CIMD - Residential Instability Q3** | -0.0418 | 0.9932 | -0.0400 | 0.9664 | -1.9888 | 1.9052 |
| **CIMD - Residential Instability Q4** | 0.4274 | 1.1314 | 0.3800 | 0.7056 | -1.7905 | 2.6453 |
| **CIMD – Residential Instability Q5 (most deprived)** | 1.8807 | 1.5147 | 1.2400 | 0.2144 | -1.0885 | 4.8499 |
| **CIMD - Residential Instability Q1 (least deprived) (REF)** | 0.0000 | . | . | . | . | . |
| **CIMD - Economic Dependency Q2** | -1.4878 | 1.2125 | -1.2300 | 0.2198 | -3.8646 | 0.8890 |
| **CIMD - Economic Dependency Q3** | 1.3915 | 1.2154 | 1.1400 | 0.2523 | -0.9911 | 3.7741 |
| **CIMD - Economic Dependency Q4** | -0.9730 | 1.2504 | -0.7800 | 0.4365 | -3.4242 | 1.4782 |
| **CIMD - Economic Dependency Q5 (most deprived)** | -0.2419 | 1.2853 | -0.1900 | 0.8507 | -2.7615 | 2.2776 |
| **CIMD - Economic Dependency Q1 (least deprived) (REF)** | 0.0000 | . | . | . | . | . |
| **CIMD - Ethnocultural Composition Q2** | -0.4236 | 0.7139 | -0.5900 | 0.5529 | -1.8232 | 0.9759 |
| **CIMD - Ethnocultural Composition Q3** | 0.2319 | 1.0335 | 0.2200 | 0.8225 | -1.7941 | 2.2579 |
| **CIMD - Ethnocultural Composition Q4** | 1.2183 | 1.5676 | 0.7800 | 0.4371 | -1.8546 | 4.2912 |
| **CIMD - Ethnocultural Composition Q5 (most deprived)** | -1.0175 | 2.4240 | -0.4200 | 0.6747 | -5.7692 | 3.7342 |
| **CIMD - Ethnocultural Composition Q1 (least deprived) (REF)** | 0.0000 | . | . | . | . | . |
| **CIMD -Situational Vulnerability Q2** | -2.9825 | 1.1832 | -2.5200 | 0.0117 | -5.3019 | -0.6631 |
| **CIMD - Situational Vulnerability Q3** | -5.9202 | 1.3105 | -4.5200 | <.0001 | -8.4892 | -3.3512 |
| **CIMD -Situational Vulnerability Q4** | -5.9338 | 1.2755 | -4.6500 | <.0001 | -8.4341 | -3.4335 |
| **CIMD -Situational Vulnerability Q5 (most deprived)** | -6.6400 | 1.3595 | -4.8800 | <.0001 | -9.3049 | -3.9750 |
| **CIMD - Situational Vulnerability Q1 (least deprived) (REF)** | 0.0000 | . | . | . | . | . |
| **Social Assistance (any in past 5 years)** | -6.3290 | 0.8410 | -7.5300 | <.0001 | -7.9777 | -4.6804 |
| **Social Assistance (none in past 5 years) (REF)** | 0.0000 | . | . | . | . | . |
| **Program of Study - French Immersion/Other** | 12.6739 | 0.9675 | 13.1000 | <.0001 | 10.7773 | 14.5705 |
| **Program of Study - French** | 35.6966 | 5.7087 | 6.2500 | <.0001 | 24.5059 | 46.8872 |
| **Program of Study - English (REF)** | 0.0000 | . | . | . | . | . |
| **Household composition – Adults (age 22+) – No adults in household** | -12.7785 | 4.6084 | -2.7700 | 0.0056 | -21.8121 | -3.7448 |
| **Household composition – Adults (age 22+) – One adult in household** | -2.4157 | 0.7084 | -3.4100 | 0.0007 | -3.8044 | -1.0269 |
| **Household composition – Adults (age 22+) – More than one adult in household (REF)** | 0.0000 | . | . | . | . | . |
| **Household composition – Children (age 21 or under) – Student is only child in household** | -0.7529 | 0.7447 | -1.0100 | 0.3120 | -2.2127 | 0.7068 |
| **Household composition – Children (age 21 or under) – Other children in household (REF)** | 0.0000 | . | . | . | . | . |
| **Recent immigrant** | 1.2530 | 6.1739 | 0.2000 | 0.8392 | -10.8495 | 13.3554 |
| **Not a recent immigrant (REF)** | 0.0000 | . | . | . | . | . |

**Supplementary Table S8f. GLM regression estimates - Provincial assessment exams percentile rank for STEM subjects for grades 9-11 (AY 2017-2019) (Untreated group as reference)**

| **Parameter** | **Estimate** | **Standard**  **Error** | **t Value** | **Pr > \|t\|** | **95% Confidence Limits** | |
| --- | --- | --- | --- | --- | --- | --- |
| **Intercept** | 0.5343 | 6.7844 | 0.0800 | 0.9372 | -12.7660 | 13.8346 |
| **Treated ADHD** | 0.9785 | 0.7769 | 1.2600 | 0.2079 | -0.5446 | 2.5016 |
| **Untreated ADHD (REF)** | 0.0000 | . | . | . | . | . |
| **Age** | -0.0031 | 0.1475 | -0.0200 | 0.9832 | -0.2922 | 0.2860 |
| **Male** | 5.2013 | 0.8240 | 6.3100 | <.0001 | 3.5859 | 6.8168 |
| **Female (REF)** | 0.0000 | . | . | . | . | . |
| **Household income quintile Q2** | -0.5806 | 1.4052 | -0.4100 | 0.6795 | -3.3353 | 2.1742 |
| **Household income quintile Q3** | -0.0606 | 1.5067 | -0.0400 | 0.9679 | -3.0143 | 2.8932 |
| **Household income quintile Q4** | -0.6108 | 1.6069 | -0.3800 | 0.7039 | -3.7609 | 2.5394 |
| **Household income quintile Q5 (highest income)** | 1.3667 | 1.7599 | 0.7800 | 0.4374 | -2.0834 | 4.8169 |
| **Household income quintile Q1 (lowest income) (REF)** | 0.0000 | . | . | . | . | . |
| **NB Health Zone 2** | 3.5951 | 1.2390 | 2.9000 | 0.0037 | 1.1661 | 6.0241 |
| **NB Health Zone 3** | 4.5684 | 1.2136 | 3.7600 | 0.0002 | 2.1892 | 6.9476 |
| **NB Health Zone 4** | 3.3420 | 1.6130 | 2.0700 | 0.0383 | 0.1798 | 6.5043 |
| **NB Health Zone 5** | 0.4017 | 2.1607 | 0.1900 | 0.8525 | -3.8341 | 4.6375 |
| **NB Health Zone 6** | 2.7705 | 1.3975 | 1.9800 | 0.0475 | 0.0309 | 5.5101 |
| **NB Health Zone 7** | 0.6422 | 1.9823 | 0.3200 | 0.7460 | -3.2439 | 4.5284 |
| **NB Health Zone 1 (REF)** | 0.0000 | . | . | . | . | . |
| **Comorbid conditions - Mood & anxiety disorders (yes)** | -0.7602 | 1.4140 | -0.5400 | 0.5909 | -3.5322 | 2.0118 |
| **Comorbid conditions - Mood & anxiety disorders (no) (REF)** | 0.0000 | . | . | . | . | . |
| **Comorbid conditions – One or more of: asthma, diabetes, epilepsy, schizophrenia (yes)** | -1.7659 | 4.1745 | -0.4200 | 0.6723 | -9.9497 | 6.4179 |
| **Comorbid conditions – One or more of: asthma, diabetes, epilepsy, schizophrenia (no) (REF)** | 0.0000 | . | . | . | . | . |
| **Select medications (one or more)** | -2.2524 | 1.2916 | -1.7400 | 0.0812 | -4.7844 | 0.2797 |
| **Select medications (none) (REF)** | 0.0000 | . | . | . | . | . |
| **School District - Anglophone** | 33.6193 | 5.8987 | 5.7000 | <.0001 | 22.0553 | 45.1833 |
| **School District – Francophone (REF)** | 0.0000 | . | . | . | . | . |
| **CIMD - Residential Instability Q2** | -1.0781 | 1.1437 | -0.9400 | 0.3459 | -3.3202 | 1.1639 |
| **CIMD - Residential Instability Q3** | -1.7338 | 1.1930 | -1.4500 | 0.1462 | -4.0726 | 0.6051 |
| **CIMD - Residential Instability Q4** | 0.3319 | 1.3744 | 0.2400 | 0.8092 | -2.3626 | 3.0263 |
| **CIMD – Residential Instability Q5 (most deprived)** | 1.9639 | 1.9065 | 1.0300 | 0.3030 | -1.7736 | 5.7013 |
| **CIMD - Residential Instability Q1 (least deprived) (REF)** | 0.0000 | . | . | . | . | . |
| **CIMD - Economic Dependency Q2** | 0.3264 | 1.5085 | 0.2200 | 0.8287 | -2.6309 | 3.2836 |
| **CIMD - Economic Dependency Q3** | 2.8447 | 1.5164 | 1.8800 | 0.0607 | -0.1282 | 5.8176 |
| **CIMD - Economic Dependency Q4** | 0.1378 | 1.5497 | 0.0900 | 0.9291 | -2.9002 | 3.1758 |
| **CIMD - Economic Dependency Q5 (most deprived)** | -0.3454 | 1.5894 | -0.2200 | 0.8280 | -3.4612 | 2.7705 |
| **CIMD - Economic Dependency Q1 (least deprived) (REF)** | 0.0000 | . | . | . | . | . |
| **CIMD - Ethnocultural Composition Q2** | -0.4496 | 0.8627 | -0.5200 | 0.6023 | -2.1408 | 1.2417 |
| **CIMD - Ethnocultural Composition Q3** | -0.8686 | 1.2682 | -0.6800 | 0.4934 | -3.3547 | 1.6176 |
| **CIMD - Ethnocultural Composition Q4** | 1.2900 | 2.0138 | 0.6400 | 0.5218 | -2.6578 | 5.2378 |
| **CIMD - Ethnocultural Composition Q5 (most deprived)** | 2.5227 | 3.3772 | 0.7500 | 0.4551 | -4.0981 | 9.1435 |
| **CIMD - Ethnocultural Composition Q1 (least deprived) (REF)** | 0.0000 | . | . | . | . | . |
| **CIMD -Situational Vulnerability Q2** | -1.6171 | 1.4708 | -1.1000 | 0.2716 | -4.5005 | 1.2663 |
| **CIMD - Situational Vulnerability Q3** | -3.4891 | 1.6117 | -2.1600 | 0.0304 | -6.6486 | -0.3295 |
| **CIMD -Situational Vulnerability Q4** | -4.3733 | 1.5718 | -2.7800 | 0.0054 | -7.4547 | -1.2920 |
| **CIMD -Situational Vulnerability Q5 (most deprived)** | -4.9476 | 1.6609 | -2.9800 | 0.0029 | -8.2037 | -1.6915 |
| **CIMD - Situational Vulnerability Q1 (least deprived) (REF)** | 0.0000 | . | . | . | . | . |
| **Social Assistance (any in past 5 years)** | -5.9950 | 1.0664 | -5.6200 | <.0001 | -8.0856 | -3.9044 |
| **Social Assistance (none in past 5 years) (REF)** | 0.0000 | . | . | . | . | . |
| **Program of Study - French Immersion/Other** | 10.9341 | 1.2175 | 8.9800 | <.0001 | 8.5474 | 13.3209 |
| **Program of Study - French** | 36.2954 | 5.9219 | 6.1300 | <.0001 | 24.6859 | 47.9049 |
| **Program of Study - English (REF)** | 0.0000 | . | . | . | . | . |
| **Household composition – Adults (age 22+) – No adults in household** | -13.3841 | 6.2658 | -2.1400 | 0.0327 | -25.6677 | -1.1005 |
| **Household composition – Adults (age 22+) – One adult in household** | -1.6246 | 0.8585 | -1.8900 | 0.0585 | -3.3077 | 0.0585 |
| **Household composition – Adults (age 22+) – More than one adult in household (REF)** | 0.0000 | . | . | . | . | . |
| **Household composition – Children (age 21 or under) – Student is only child in household** | -3.4845 | 0.9075 | -3.8400 | 0.0001 | -5.2636 | -1.7054 |
| **Household composition – Children (age 21 or under) – Other children in household (REF)** | 0.0000 | . | . | . | . | . |
| **Recent immigrant** | 13.1851 | 8.1665 | 1.6100 | 0.1065 | -2.8248 | 29.1949 |
| **Not a recent immigrant (REF)** | 0.0000 | . | . | . | . | . |

**Supplementary Table S8g. GLM regression estimates - Provincial assessment exams percentile rank for math for grades 9-11 (AY 2017-2019) (Untreated group as reference)**

| **Parameter** | **Estimate** | **Standard**  **Error** | **t Value** | **Pr > \|t\|** | **95% Confidence Limits** | |
| --- | --- | --- | --- | --- | --- | --- |
| **Intercept** | 26.6707 | 21.0007 | 1.2700 | 0.2043 | -14.5183 | 67.8597 |
| **Treated ADHD** | 3.4145 | 1.3413 | 2.5500 | 0.0110 | 0.7838 | 6.0452 |
| **Untreated ADHD (REF)** | 0.0000 | . | . | . | . | . |
| **Age** | -2.2540 | 0.9690 | -2.3300 | 0.0201 | -4.1545 | -0.3535 |
| **Male** | 4.1285 | 1.4104 | 2.9300 | 0.0035 | 1.3622 | 6.8948 |
| **Female (REF)** | 0.0000 | . | . | . | . | . |
| **Household income quintile Q2** | 1.3656 | 2.4227 | 0.5600 | 0.5731 | -3.3862 | 6.1173 |
| **Household income quintile Q3** | 1.3153 | 2.5984 | 0.5100 | 0.6128 | -3.7810 | 6.4116 |
| **Household income quintile Q4** | 3.6474 | 2.7517 | 1.3300 | 0.1852 | -1.7495 | 9.0444 |
| **Household income quintile Q5 (highest income)** | 7.2504 | 3.0043 | 2.4100 | 0.0159 | 1.3579 | 13.1428 |
| **Household income quintile Q1 (lowest income) (REF)** | 0.0000 | . | . | . | . | . |
| **NB Health Zone 2** | 4.5499 | 1.8558 | 2.4500 | 0.0143 | 0.9100 | 8.1897 |
| **NB Health Zone 3** | 10.0842 | 1.9277 | 5.2300 | <.0001 | 6.3034 | 13.8650 |
| **NB Health Zone 4** | 11.1261 | 3.4891 | 3.1900 | 0.0015 | 4.2829 | 17.9693 |
| **NB Health Zone 5** | -3.9010 | 3.7144 | -1.0500 | 0.2938 | -11.1862 | 3.3841 |
| **NB Health Zone 6** | -1.3050 | 2.7603 | -0.4700 | 0.6364 | -6.7188 | 4.1089 |
| **NB Health Zone 7** | 6.9373 | 3.2362 | 2.1400 | 0.0322 | 0.5901 | 13.2846 |
| **NB Health Zone 1 (REF)** | 0.0000 | . | . | . | . | . |
| **Comorbid conditions - Mood & anxiety disorders (yes)** | -2.3385 | 1.9262 | -1.2100 | 0.2249 | -6.1164 | 1.4394 |
| **Comorbid conditions - Mood & anxiety disorders (no) (REF)** | 0.0000 | . | . | . | . | . |
| **Comorbid conditions – One or more of: asthma, diabetes, epilepsy, schizophrenia (yes)** | -1.6779 | 6.3444 | -0.2600 | 0.7915 | -14.1214 | 10.7656 |
| **Comorbid conditions – One or more of: asthma, diabetes, epilepsy, schizophrenia (no) (REF)** | 0.0000 | . | . | . | . | . |
| **Select medications (one or more)** | -6.1620 | 2.3157 | -2.6600 | 0.0079 | -10.7038 | -1.6202 |
| **Select medications (none) (REF)** | 0.0000 | . | . | . | . | . |
| **School District - Anglophone** | 30.2589 | 13.5430 | 2.2300 | 0.0256 | 3.6966 | 56.8211 |
| **School District – Francophone (REF)** | 0.0000 | . | . | . | . | . |
| **CIMD - Residential Instability Q2** | -0.9818 | 1.8876 | -0.5200 | 0.6030 | -4.6841 | 2.7204 |
| **CIMD - Residential Instability Q3** | -3.4015 | 2.0375 | -1.6700 | 0.0952 | -7.3977 | 0.5946 |
| **CIMD - Residential Instability Q4** | 2.0678 | 2.3302 | 0.8900 | 0.3750 | -2.5025 | 6.6380 |
| **CIMD – Residential Instability Q5 (most deprived)** | 8.9526 | 3.3041 | 2.7100 | 0.0068 | 2.4722 | 15.4330 |
| **CIMD - Residential Instability Q1 (least deprived) (REF)** | 0.0000 | . | . | . | . | . |
| **CIMD - Economic Dependency Q2** | 5.9644 | 2.5322 | 2.3600 | 0.0186 | 0.9979 | 10.9309 |
| **CIMD - Economic Dependency Q3** | 8.6956 | 2.5268 | 3.4400 | 0.0006 | 3.7396 | 13.6515 |
| **CIMD - Economic Dependency Q4** | 6.4114 | 2.6039 | 2.4600 | 0.0139 | 1.3042 | 11.5185 |
| **CIMD - Economic Dependency Q5 (most deprived)** | 8.4000 | 2.6478 | 3.1700 | 0.0015 | 3.2068 | 13.5932 |
| **CIMD - Economic Dependency Q1 (least deprived) (REF)** | 0.0000 | . | . | . | . | . |
| **CIMD - Ethnocultural Composition Q2** | 0.4439 | 1.4678 | 0.3000 | 0.7623 | -2.4348 | 3.3227 |
| **CIMD - Ethnocultural Composition Q3** | -2.0745 | 2.1294 | -0.9700 | 0.3301 | -6.2509 | 2.1019 |
| **CIMD - Ethnocultural Composition Q4** | 6.0850 | 3.4640 | 1.7600 | 0.0792 | -0.7089 | 12.8790 |
| **CIMD - Ethnocultural Composition Q5 (most deprived)** | 1.5373 | 5.9381 | 0.2600 | 0.7958 | -10.1092 | 13.1838 |
| **CIMD - Ethnocultural Composition Q1 (least deprived) (REF)** | 0.0000 | . | . | . | . | . |
| **CIMD -Situational Vulnerability Q2** | -2.5154 | 2.3872 | -1.0500 | 0.2922 | -7.1975 | 2.1666 |
| **CIMD - Situational Vulnerability Q3** | -3.2991 | 2.6638 | -1.2400 | 0.2157 | -8.5237 | 1.9255 |
| **CIMD -Situational Vulnerability Q4** | -6.3023 | 2.5667 | -2.4600 | 0.0142 | -11.3364 | -1.2682 |
| **CIMD -Situational Vulnerability Q5 (most deprived)** | -6.9108 | 2.7047 | -2.5600 | 0.0107 | -12.2156 | -1.6060 |
| **CIMD - Situational Vulnerability Q1 (least deprived) (REF)** | 0.0000 | . | . | . | . | . |
| **Social Assistance (any in past 5 years)** | -2.7379 | 1.9621 | -1.4000 | 0.1631 | -6.5862 | 1.1105 |
| **Social Assistance (none in past 5 years) (REF)** | 0.0000 | . | . | . | . | . |
| **Program of Study - French Immersion/Other** | 13.0659 | 1.7845 | 7.3200 | <.0001 | 9.5660 | 16.5658 |
| **Program of Study - French** | 39.9269 | 13.5723 | 2.9400 | 0.0033 | 13.3071 | 66.5466 |
| **Program of Study - English (REF)** | 0.0000 | . | . | . | . | . |
| **Household composition – Adults (age 22+) – No adults in household** | -14.3820 | 15.5539 | -0.9200 | 0.3553 | -44.8881 | 16.1241 |
| **Household composition – Adults (age 22+) – One adult in household** | 1.6538 | 1.5337 | 1.0800 | 0.2810 | -1.3543 | 4.6620 |
| **Household composition – Adults (age 22+) – More than one adult in household (REF)** | 0.0000 | . | . | . | . | . |
| **Household composition – Children (age 21 or under) – Student is only child in household** | -4.0231 | 1.4683 | -2.7400 | 0.0062 | -6.9028 | -1.1434 |
| **Household composition – Children (age 21 or under) – Other children in household (REF)** | 0.0000 | . | . | . | . | . |
| **Recent immigrant** | 9.5896 | 13.5561 | 0.7100 | 0.4794 | -16.9982 | 36.1774 |
| **Not a recent immigrant (REF)** | 0.0000 | . | . | . | . | . |

**Supplementary Table S8h. GLM regression estimates - Provincial assessment exams percentile rank for language for grades 9-11 (AY 2017-2019) (Untreated group as reference)**

| **Parameter** | **Estimate** | **Standard**  **Error** | **t Value** | **Pr > \|t\|** | **95% Confidence Limits** | |
| --- | --- | --- | --- | --- | --- | --- |
| **Intercept** | 52.4637 | 16.0925 | 3.2600 | 0.0011 | 20.9164 | 84.0110 |
| **Treated ADHD** | -0.1212 | 0.7602 | -0.1600 | 0.8733 | -1.6115 | 1.3690 |
| **Untreated ADHD (REF)** | 0.0000 | . | . | . | . | . |
| **Age** | -0.0985 | 0.1306 | -0.7500 | 0.4511 | -0.3546 | 0.1577 |
| **Male** | -0.0220 | 0.7726 | -0.0300 | 0.9773 | -1.5366 | 1.4926 |
| **Female (REF)** | 0.0000 | . | . | . | . | . |
| **Household income quintile Q2** | 0.5612 | 1.3167 | 0.4300 | 0.6699 | -2.0200 | 3.1425 |
| **Household income quintile Q3** | -0.6814 | 1.4700 | -0.4600 | 0.6430 | -3.5632 | 2.2003 |
| **Household income quintile Q4** | 1.6916 | 1.5596 | 1.0800 | 0.2781 | -1.3658 | 4.7490 |
| **Household income quintile Q5 (highest income)** | 0.1077 | 1.7362 | 0.0600 | 0.9505 | -3.2958 | 3.5112 |
| **Household income quintile Q1 (lowest income) (REF)** | 0.0000 | . | . | . | . | . |
| **NB Health Zone 2** | 4.0610 | 1.0042 | 4.0400 | <.0001 | 2.0923 | 6.0296 |
| **NB Health Zone 3** | 4.4175 | 1.0314 | 4.2800 | <.0001 | 2.3956 | 6.4395 |
| **NB Health Zone 4** | -4.2863 | 2.3854 | -1.8000 | 0.0724 | -8.9625 | 0.3900 |
| **NB Health Zone 5** | 3.3750 | 2.2583 | 1.4900 | 0.1351 | -1.0521 | 7.8020 |
| **NB Health Zone 6** | 4.0783 | 1.8138 | 2.2500 | 0.0246 | 0.5226 | 7.6341 |
| **NB Health Zone 7** | 1.9243 | 1.8514 | 1.0400 | 0.2987 | -1.7050 | 5.5537 |
| **NB Health Zone 1 (REF)** | 0.0000 | . | . | . | . | . |
| **Comorbid conditions - Mood & anxiety disorders (yes)** | 0.8861 | 1.3194 | 0.6700 | 0.5019 | -1.7004 | 3.4726 |
| **Comorbid conditions - Mood & anxiety disorders (no) (REF)** | 0.0000 | . | . | . | . | . |
| **Comorbid conditions – One or more of: asthma, diabetes, epilepsy, schizophrenia (yes)** | 0.4037 | 3.8038 | 0.1100 | 0.9155 | -7.0531 | 7.8605 |
| **Comorbid conditions – One or more of: asthma, diabetes, epilepsy, schizophrenia (no) (REF)** | 0.0000 | . | . | . | . | . |
| **Select medications (one or more)** | -1.8131 | 1.2071 | -1.5000 | 0.1332 | -4.1795 | 0.5534 |
| **Select medications (none) (REF)** | 0.0000 | . | . | . | . | . |
| **School District - Anglophone** | -14.1124 | 15.8223 | -0.8900 | 0.3725 | -45.1300 | 16.9052 |
| **School District – Francophone (REF)** | 0.0000 | . | . | . | . | . |
| **CIMD - Residential Instability Q2** | 0.3269 | 1.0987 | 0.3000 | 0.7661 | -1.8270 | 2.4807 |
| **CIMD - Residential Instability Q3** | 1.6709 | 1.1719 | 1.4300 | 0.1540 | -0.6265 | 3.9684 |
| **CIMD - Residential Instability Q4** | 1.0565 | 1.2966 | 0.8100 | 0.4152 | -1.4854 | 3.5984 |
| **CIMD – Residential Instability Q5 (most deprived)** | 2.6261 | 1.6943 | 1.5500 | 0.1212 | -0.6954 | 5.9476 |
| **CIMD - Residential Instability Q1 (least deprived) (REF)** | 0.0000 | . | . | . | . | . |
| **CIMD - Economic Dependency Q2** | -0.0556 | 1.3628 | -0.0400 | 0.9674 | -2.7273 | 2.6160 |
| **CIMD - Economic Dependency Q3** | 1.7046 | 1.3686 | 1.2500 | 0.2130 | -0.9783 | 4.3875 |
| **CIMD - Economic Dependency Q4** | -0.7391 | 1.4134 | -0.5200 | 0.6010 | -3.5100 | 2.0317 |
| **CIMD - Economic Dependency Q5 (most deprived)** | 2.8676 | 1.4454 | 1.9800 | 0.0473 | 0.0341 | 5.7011 |
| **CIMD - Economic Dependency Q1 (least deprived) (REF)** | 0.0000 | . | . | . | . | . |
| **CIMD - Ethnocultural Composition Q2** | -0.1364 | 0.8358 | -0.1600 | 0.8703 | -1.7749 | 1.5021 |
| **CIMD - Ethnocultural Composition Q3** | -0.3481 | 1.1921 | -0.2900 | 0.7703 | -2.6850 | 1.9889 |
| **CIMD - Ethnocultural Composition Q4** | 0.7260 | 1.7524 | 0.4100 | 0.6787 | -2.7094 | 4.1614 |
| **CIMD - Ethnocultural Composition Q5 (most deprived)** | -2.6318 | 2.5514 | -1.0300 | 0.3024 | -7.6334 | 2.3699 |
| **CIMD - Ethnocultural Composition Q1 (least deprived) (REF)** | 0.0000 | . | . | . | . | . |
| **CIMD -Situational Vulnerability Q2** | -4.0235 | 1.3036 | -3.0900 | 0.0020 | -6.5791 | -1.4680 |
| **CIMD - Situational Vulnerability Q3** | -7.5930 | 1.4834 | -5.1200 | <.0001 | -10.5011 | -4.6849 |
| **CIMD -Situational Vulnerability Q4** | -6.9541 | 1.4389 | -4.8300 | <.0001 | -9.7750 | -4.1332 |
| **CIMD -Situational Vulnerability Q5 (most deprived)** | -7.0227 | 1.5527 | -4.5200 | <.0001 | -10.0665 | -3.9789 |
| **CIMD - Situational Vulnerability Q1 (least deprived) (REF)** | 0.0000 | . | . | . | . | . |
| **Social Assistance (any in past 5 years)** | -4.2537 | 0.9489 | -4.4800 | <.0001 | -6.1138 | -2.3935 |
| **Social Assistance (none in past 5 years) (REF)** | 0.0000 | . | . | . | . | . |
| **Program of Study - French Immersion/Other** | 12.3072 | 0.9424 | 13.0600 | <.0001 | 10.4598 | 14.1547 |
| **Program of Study - French** | -8.8828 | 15.8170 | -0.5600 | 0.5744 | -39.8900 | 22.1243 |
| **Program of Study - English (REF)** | 0.0000 | . | . | . | . | . |
| **Household composition – Adults (age 22+) – No adults in household** | -13.9537 | 5.6638 | -2.4600 | 0.0138 | -25.0569 | -2.8506 |
| **Household composition – Adults (age 22+) – One adult in household** | -2.1195 | 0.8417 | -2.5200 | 0.0118 | -3.7696 | -0.4694 |
| **Household composition – Adults (age 22+) – More than one adult in household (REF)** | 0.0000 | . | . | . | . | . |
| **Household composition – Children (age 21 or under) – Student is only child in household** | 1.1343 | 0.8653 | 1.3100 | 0.1900 | -0.5620 | 2.8306 |
| **Household composition – Children (age 21 or under) – Other children in household (REF)** | 0.0000 | . | . | . | . | . |
| **Recent immigrant** | 8.0503 | 5.8592 | 1.3700 | 0.1695 | -3.4360 | 19.5365 |
| **Not a recent immigrant (REF)** | 0.0000 | . | . | . | . | . |
