# Supplementary material for: Academic Outcomes in Primary and Secondary School Students Prescribed Long-Acting Stimulants for ADHD Management
Source: J Atten Disord. 2025 Oct 7;30(4):493–505. doi: 10.1177/10870547251378169 (PMC12953683; doi:10.1177/10870547251378169)
Supplement: sj-docx-9-jad-10.1177_10870547251378169 – Supplemental material for Academic Outcomes in Primary and Secondary School Students Prescribed Long-Acting Stimulants for ADHD Management [file sj-docx-9-jad-10.1177_10870547251378169.docx]

**Supplementary Table S9. Logistic regression estimates - Likelihood of not graduating from high school on first attempt (AY 2017-2019)**

| **Odds Ratio Estimates** | | | |
| --- | --- | --- | --- |
| **Effect** | **Point Estimate** | **95% Wald**  **Confidence Limits** | |
| **Treated ADHD vs No ADHD** | 1.39 | 1.02 | 1.894 |
| **Untreated ADHD vs No ADHD** | 2.222 | 1.871 | 2.64 |
| **Age** | 1.106 | 1.061 | 1.152 |
| **Male vs Female** | 1.125 | 0.98 | 1.293 |
| **Household income quintile Q2 vs Q1 (lowest income)** | 0.887 | 0.706 | 1.114 |
| **Household income quintile Q3 vs Q1 (lowest income)** | 0.926 | 0.713 | 1.204 |
| **Household income quintile Q4 vs Q1 (lowest income)** | 0.927 | 0.699 | 1.23 |
| **Household income quintile Q5 (highest income) vs Q1 (lowest income)** | 0.888 | 0.648 | 1.216 |
| **NB Health Zone 2 vs Zone 1** | 0.816 | 0.673 | 0.99 |
| **NB Health Zone 3 vs Zone 1** | 0.885 | 0.729 | 1.074 |
| **NB Health Zone 4 vs Zone 1** | 0.822 | 0.541 | 1.249 |
| **NB Health Zone 5 vs Zone 1** | 0.742 | 0.47 | 1.172 |
| **NB Health Zone 6 vs Zone 1** | 0.94 | 0.684 | 1.29 |
| **NB Health Zone 7 vs Zone 1** | 1.124 | 0.84 | 1.504 |
| **Comorbid conditions – Mood & anxiety disorders - yes vs no** | 1.575 | 1.139 | 2.178 |
| **Comorbid conditions – One or more of: asthma, diabetes, epilepsy, schizophrenia - yes vs no** | 0.889 | 0.502 | 1.573 |
| **Select medications - yes vs no** | 3.91 | 3.064 | 4.988 |
| **School District - Anglophone vs Francophone** | 0.653 | 0.402 | 1.062 |
| **CIMD - Residential Instability Q2 vs Q1 (least deprived)** | 0.984 | 0.795 | 1.217 |
| **CIMD - Residential Instability Q3 vs Q1 (least deprived)** | 1.13 | 0.903 | 1.414 |
| **CIMD - Residential Instability Q4 vs Q1 (least deprived)** | 1.118 | 0.866 | 1.443 |
| **CIMD - Residential Instability Q5 (most deprived) vs Q1 (least deprived)** | 1.507 | 1.103 | 2.06 |
| **CIMD - Economic Dependency Q2 vs Q1 (least deprived)** | 0.914 | 0.697 | 1.198 |
| **CIMD - Economic Dependency Q3 vs Q1 (least deprived)** | 0.881 | 0.674 | 1.151 |
| **CIMD - Economic Dependency Q4 vs Q1 (least deprived)** | 0.868 | 0.661 | 1.14 |
| **CIMD - Economic Dependency Q5 (most deprived) vs Q1 (least deprived)** | 0.821 | 0.623 | 1.083 |
| **CIMD - Ethnocultural Composition Q2 vs Q1 (least deprived)** | 1.154 | 0.987 | 1.348 |
| **CIMD - Ethnocultural Composition Q3 vs Q1 (least deprived)** | 1.013 | 0.8 | 1.281 |
| **CIMD - Ethnocultural Composition Q4 vs Q1 (least deprived)** | 0.902 | 0.64 | 1.27 |
| **CIMD - Ethnocultural Composition Q5 (most deprived) vs Q1 (least deprived)** | 1.067 | 0.705 | 1.615 |
| **CIMD - Situational Vulnerability Q2 vs Q1 (least deprived)** | 1.111 | 0.852 | 1.45 |
| **CIMD - Situational Vulnerability Q3 vs Q1 (least deprived)** | 1.178 | 0.881 | 1.575 |
| **CIMD - Situational Vulnerability Q4 vs Q1 (least deprived)** | 1.37 | 1.038 | 1.807 |
| **CIMD - Situational Vulnerability Q5 (most deprived) vs Q1 (least deprived)** | 1.353 | 1.006 | 1.82 |
| **Social Assistance – any received in past 5 years - yes vs no** | 2.708 | 2.285 | 3.21 |
| **Program of Study - French Immersion/Other vs English** | 0.245 | 0.19 | 0.316 |
| **Program of Study - French vs English** | 0.159 | 0.095 | 0.265 |
| **Household composition – Adults (age 22+) – No adults in household vs More than one adult in household** | 4.777 | 3.817 | 5.979 |
| **Household composition – Adults (age 22+) – One adult in household vs More than one adult in household** | 1.442 | 1.214 | 1.714 |
| **Household composition - Children (age 21 or under) – Student is only child in household vs Other children in household** | 1.148 | 0.989 | 1.332 |
| **Recent immigrant vs Not a recent immigrant** | 0.648 | 0.434 | 0.968 |
